# Full‐Length 16S and 18S rRNA Long‐Read Sequencing Reveals Gut Microbiome Diversity in the European Brown Hare ( *Lepus europaeus* )

**DOI:** 10.1111/1758-2229.70358

**Published:** 2026-05-25

**Authors:** Zbigniew Bełkot, Mateusz G. Adamski, Zuzanna J. Strzałkowska, Ewa D. Domańska, Daria Kłosińska, Grzegorz Kunstman, Dawid Skoczek, Joanna Pławińska‐Czarnak

**Affiliations:** ^1^ Department of Food Hygiene of Animal Origin, Faculty of Veterinary Medicine University of Life Sciences in Lublin Lublin Poland; ^2^ SPARK‐TECH Ltd Krakow Poland; ^3^ Department of Preclinical Sciences, Institute of Veterinary Medicine Warsaw University of Life Sciences Warsaw Poland; ^4^ Division of Histology and Embryology, Department of Morphological Sciences, Institute of Veterinary Medicine Warsaw University of Life Sciences Warsaw Poland; ^5^ Malopolska Centre of Biotechnology Jagiellonian University Krakow Poland

**Keywords:** 16S/18S rRNA, brown hare, gut microbiome, *Lepus europaeus*, one health, third‐generation sequencing, wildlife ecology

## Abstract

The European brown hare ( *Lepus europaeus* ) is a declining wildlife species of ecological and epidemiological importance, yet its intestinal microbiome remains poorly characterized. Here, Oxford Nanopore long‐read sequencing was used to analyse full‐length 16S and 18S rRNA genes from pooled large‐intestine contents of 30 healthy hares divided into three groups. Comparative taxonomic assignment at 95% and 80% sequence identity thresholds revealed striking differences in diversity estimates, with the lower threshold uncovering up to ten‐fold more taxa. Across all samples, 40 phyla, 360 families, 1027 genera, and 3373 species were identified, including 30 taxa not previously reported in lagomorphs. These included *Monoglobus pectinilyticus*, *Ruminococcus champanellensis* , *Odoribacter splanchnicus* , *Butyricimonas virosa* , and *Akkermansia muciniphila* , associated with pectin degradation, cellulose hydrolysis, butyrate production, mucin degradation, bile acid transformation, and nitrogen recycling. Several taxa relevant to both animal and human health were also detected, supporting hares as sentinels of environmental microbiota within a One Health framework. These findings show that analytical parameter selection strongly shapes microbiome interpretation and provide the most comprehensive gut microbiome profile of the European brown hare to date. The study expands lagomorph microbial ecology and highlights long‐read sequencing as a valuable tool for wildlife microbiome surveillance in undercharacterized host species globally.

## Graphical abstract

Full‐length 16S and 18S rRNA Oxford Nanopore sequencing of large intestine contents from 30 healthy European brown hares revealed broad gut microbiome diversity. An 80% identity threshold detected substantially greater taxonomic richness than 95%, emphasizing the value of long‐read sequencing in wildlife microbiome research.

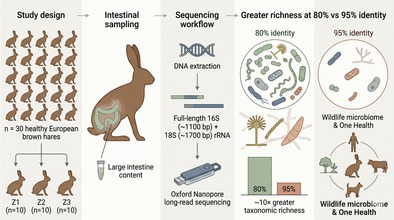

## 1 Introduction

The brown hare ( *Lepus europaeus* Pall.) is a small game species that is very popular in Poland. However, its population has significantly decreased over the last 50 years. In the 1970s, the population was estimated to be over 3 million individuals. Since then, this number has declined fivefold, and the hunting acquisition of this species has also drastically decreased. The rapid decline in the hare population is attributed to a variety of factors (Demirbaş 2015; Nasiadka and Dziedzic 2014). The decline in the hare population can be attributed to several common factors. Firstly, the intensification of agriculture has led to the loss of essential habitats. Urbanization and the expansion of the road network also contribute to this issue. Additionally, pollution affecting soil, water, and air, along with ongoing climate change, negatively impacts reproductive processes in hares. Another critical factor in the decline is the significant increase in predation, particularly by foxes. This rise in fox numbers is linked to successful rabies vaccination programs. The presence of invasive species such as raccoon dogs and raccoons, as well as birds of prey and corvids, further affects hare populations. Moreover, poaching and hunting activities pose additional threats, along with the influence of synanthropic predators, especially outdoor domestic cats (Wierzbowska et al. 2016; Panek 2018; Farkas et al. 2016; Sliwinski et al. 2019; Fitzner et al. 2022). A significant factor that influences the hare population is the spread of infectious and parasitic diseases. These diseases can lead to a decline in their overall health and reproductive capacity, and they are often a direct cause of mortality (Posautz et al. 2015; Diakou et al. 2014; Kornaś et al. 2014; Panayotova‐Pencheva 2022) The brown hare is a typical herbivorous animal. It rarely drinks water; instead, it meets its hydration needs through the plants it consumes. In spring and summer, it primarily feeds on above‐ground parts of plants. In autumn, its diet may include roots and other plant foods sourced from underground. During the day, brown hares gnaw on twigs from trees and shrubs, and they also enjoy the young shoots found in streams. Their favourite foods include young seedlings of fruit crops as well as a variety of agricultural crops (Okarma and Tomek 2024). Hares reach sexual maturity at approximately 8 months of age. A typical litter consists of 2–5 young; however, in areas influenced by human activity and agriculture, the actual reproductive capacity is often limited to 1 or 2 young. This limitation is primarily due to high levels of indicator metals that affect the reproduction of these animals, along with other factors impacting reproductive success. Studies indicate that out of 10 young born to a single female in a year (across four litters), only one typically survives to adulthood (Wajdzik et al. 2017). In Poland, the hunting season for hares lasts for 2 months, from November 1 to December 31. Hunting can only occur during organized collective hunts, which require the participation of at least six hunters. The carcasses of hunted hares are highly valuable for their culinary uses, as their meat is considered a prized food source.

The brown hare ( *Lepus europaeus* ) plays a key role in forest and agricultural ecosystems, acting as a vital link in the trophic cascades and a potential reservoir of pathogens. Its gut microbiome is a dynamic ecosystem of microorganisms that play fundamental roles in metabolic processes, nutrient digestion, and ecological interactions.

Studies of the intestinal microbiome of the brown hare reveal a dominance of bacteria from the phyla *Firmicutes* and *Bacteroidetes*. These bacteria are essential for digesting dietary fibre and for the host's energy metabolism (Stalder et al. 2019). The high abundance of these bacteria indicates that the hare has adapted to a diet rich in plant‐based components, including difficult‐to‐digest polysaccharides. Additionally, the presence of bacteria from the genus *Ruminococcus* suggests that the hare is capable of efficiently fermenting cellulose, which may enhance its ability to extract energy from the plant material it consumes (Stalder et al. 2019).

A comparative analysis of the gut microbiome in the brown hare and the European rabbit ( *Oryctolagus cuniculus* ) reveals significant differences in their microbiota composition (Shanmuganandam et al. 2020). Hares have been observed to possess a greater diversity of microorganisms that are involved in carbohydrate metabolism, indicating their capability to utilize a wider range of food resources. These differences may hold ecological significance regarding competition for food niches and could impact the population dynamics of both species in ecosystems where they coexist.

Additional studies indicate an essential role for enzymes such as urease in metabolic processes occurring in the large intestine of the hare (Miśta et al. 2018). The high activity of this enzyme may be related to nitrogen recycling, which allows for more efficient use of nutrients and affects the nitrogen balance in ecosystems. This may be crucial for the trophic structure and functioning of habitats in which the hare occurs.

The hare microbiome may also play a role in the transmission of pathogens with zoonotic potential. Studies show the presence of bacteria from the genera *Escherichia, Listeria, Clostridium* and *Salmonella*, which may be etiological factors of infections in other animals and humans (unpublished own studies). The research on the digestive microbiome of the brown hare ( *Lepus europaeus* ) is currently limited, and the existing scientific literature in this field remains insufficient.

Next‐generation sequencing (NGS) is widely used to study the microbiomes of humans and animals (Combrink et al. 2023; Forcina et al. 2022). This technology allows for high‐throughput parallel sequencing of short DNA fragments. Key methodologies involved in NGS include the fragmentation of DNA into short reads (typically 100–300 base pairs for Illumina and up to 600 base pairs for Ion Torrent, with some specialized applications reaching up to 800 base pairs), polymerase chain reaction (PCR) amplification to increase the amount of genetic material (which can introduce amplification biases), and sequencing by synthesis, where fluorescent signals are detected during the incorporation of nucleotides. NGS is extensively applied in microbiome research due to its high sequencing depth, cost efficiency per sample, and high accuracy in short‐read analysis. However, its primary limitation lies in the short read length, which poses challenges in the de novo assembly of complex bacterial genomes and the precise differentiation of closely related microbial taxa. This limitation typically restricts the sequencing of the 16S rRNA gene to partial coverage of 3–4 variable regions at most, depending on the platform and protocol used. Such read length constraints often make it difficult to distinguish between closely related bacterial species, although still allowing for accurate genus‐level identification. While this approach has been the cornerstone of microbiome studies for years, the inability to consistently resolve species‐level differences has been a persistent challenge for researchers seeking fine‐scale taxonomic resolution (Weinroth et al. 2022). In this study, our primary aim was to obtain a population‐level profile of the hare gut microbiome; therefore, we applied a pooling strategy to emphasize community‐wide microbial signatures rather than individual‐specific variation.

Third‐generation sequencing (TGS) technologies represent a paradigm shift by enabling long‐read sequencing without length limitations for DNA fragments. This advancement allows for the sequencing of entire 16S rRNA genes (approximately 1500 base pairs), providing significantly improved taxonomic resolution. Currently, two major platforms dominate the TGS landscape. Pacific Biosciences (PacBio) employs Single Molecule Real‐Time (SMRT) sequencing, which utilizes zero‐mode waveguides to detect fluorescent signals from single DNA molecules during synthesis, allowing for real‐time observation of nucleotide incorporation without amplification bias. In contrast, Oxford Nanopore Technologies uses a fundamentally different approach based on ion flow through nanopores. This platform integrates electronic chips with sequencing proteins, where neural networks interpret nucleotides based on voltage differences as DNA molecules pass through the nanopores—a substantial departure from conventional sequencing methodologies that rely on optical detection systems (Bayega et al. 2018).

The recent introduction of Oxford Nanopore's Q14 chemistry and improved flow cell designs has revolutionized the field, with quality and accuracy scores now comparable to other sequencing platforms (Q scores above 30). This technological advancement has effectively eliminated the previous accuracy concerns associated with nanopore sequencing, making it a viable option for high‐resolution microbiome studies.

This article presents how the use of third‐generation sequencing can significantly expand the range of microorganisms detected in the microbiome based on sequencing the full‐length 16S and 18S rRNA gene amplicons from the large intestine of the brown hare. By leveraging the capabilities of long‐read sequencing, we demonstrate enhanced taxonomic resolution that provides deeper insights into microbial community structure and function than previously possible with short‐read technologies.

## 2 Results

### 2.1 Comparative Analysis of Taxonomic Diversity at Different Sequence Identity Thresholds

Key biodiversity metrics calculated for hare rectal microbiome samples at two different sequence identity thresholds (95% and 80%) were presented in Table 1. The indices include Shannon diversity (H), which measures both richness and evenness; Species Richness, representing the total number of unique species identified; Simpson diversity (1‐D), which emphasizes species dominance; Pielou's evenness (J), indicating how evenly species are distributed; and Chao1, an estimator of true species richness accounting for undetected species.

**TABLE 1:** Comparison of biodiversity indices between 95% and 80% sequence identity thresholds across three samples (Z1, Z2, and Z3).

Index	Z1 (95%)	Z1 (80%)	Z2 (95%)	Z2 (80%)	Z3 (95%)	Z3 (80%)
Shannon H	3.21	4.66	3.14	5.09	4.18	4.89
Species richness	168.00	2182.00	212.00	2405.00	328.00	3051.00
Simpson 1‐D	0.91	0.94	0.87	0.96	0.97	0.94
Pielou J	0.63	0.61	0.59	0.65	0.72	0.61
Chao1	265.53	4450.33	332.44	4353.67	541.49	5984.93

**FIGURE 1:**
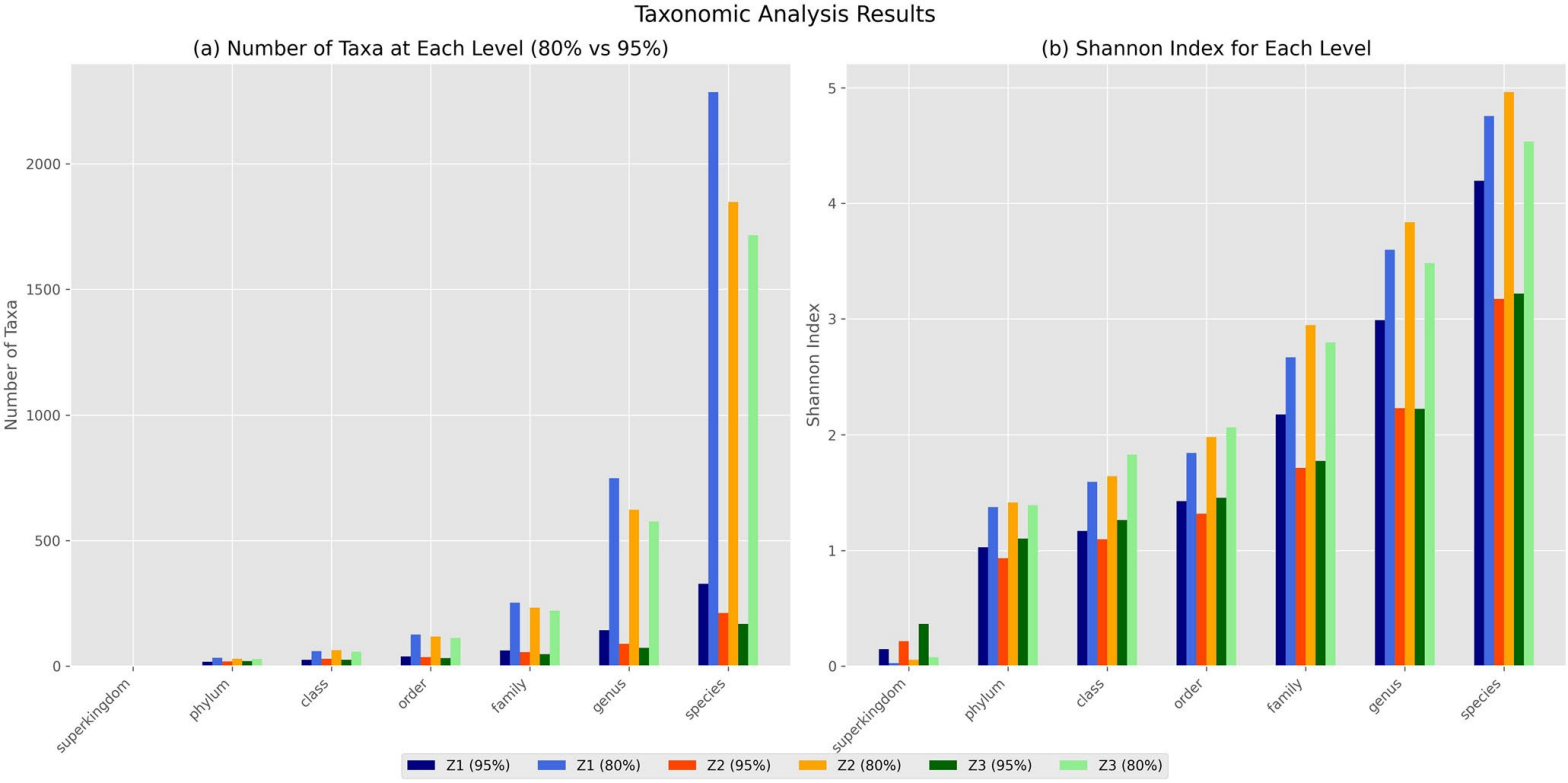
Taxonomic diversity (a) and Shannon diversity index (b) comparison between 95% and 80% sequence identity thresholds across different taxonomic levels for samples Z1, Z2, and Z3. Data are presented as counts of taxa and Shannon index values calculated from pooled sample profiles (Z1–Z3).

This analysis is visually supported by Figure 1, where panel (b) illustrates the substantial differences in Shannon diversity indices across taxonomic levels, consistently showing higher values for the 80% threshold samples.

As shown in Figure 1A, the 80% threshold leads to a significant increase in taxonomic richness compared to the 95% threshold, particularly at the genus and species levels, where the differences are most evident. While the 95% threshold offers greater confidence in taxonomic assignments, the 80% threshold markedly broadens the detectable diversity, revealing a wider array of microorganisms that might otherwise remain undetected with stricter matching criteria. A detailed comparison of taxonomic assignments across seven levels, from Superkingdom to Species, is presented in Table 2. Importantly, both thresholds were applied to the same set of quality‐filtered reads; the difference in “Assigned sequences” reflects the number of reads whose best BLAST hit meets each respective threshold, not a difference in the input data. This table includes the total number of taxa identified, the number of assigned sequences, the percentage of assigned sequences, the taxa shared between thresholds, and the taxa uniquely identified at the 80% threshold. This systematic comparison demonstrates how the stringency of sequence matching impacts the depth and breadth of taxonomic classification.

**TABLE 2:** Comprehensive taxonomic composition analysis across all hierarchical levels at 95% and 80% sequence identity thresholds for three microbiome samples (Z1, Z2, and Z3).

Taxonomic Level	Parameter	Z1 (95%)	Z1 (80%)	Z2 (95%)	Z2 (80%)	Z3 (95%)	Z3 (80%)
Superkingdom	Number of taxa	2	2	2	2	2	2
	Assigned sequences	2471	33,573	4105	41,881	4595	58,314
	% of assigned sequences	100.00	99.99	100.00	99.99	100.00	97.53
	Shared taxa between thresholds	2	—	2	—	2	—
	Unique taxa for 80%	—	0	—	0	—	0
Phylum	Number of taxa	19	28	18	29	17	33
	Assigned sequences	2457	31,204	4077	39,278	4413	55,625
	% of assigned sequences	99.43	92.94	99.32	93.77	96.04	93.03
	Shared taxa between thresholds	19	—	18	—	17	—
	Unique taxa for 80%	—	9	—	11	—	16
Class	Number of taxa	25	57	29	63	25	59
	Assigned sequences	2414	29,913	4005	36,711	4215	52,694
	% of assigned sequences	97.69	89.09	97.56	87.64	91.73	88.13
	Shared taxa between thresholds	25	—	29	—	25	—
	Unique taxa for 80%	—	32	—	34	—	34
Order	Number of taxa	31	112	35	117	38	126
	Assigned sequences	2423	29,851	4019	36,668	4217	52,348
	% of assigned sequences	98.06	88.91	97.90	87.54	91.77	87.55
	Shared taxa between thresholds	31	—	35	—	38	—
	Unique taxa for 80%	—	81	—	82	—	88
Family	Number of taxa	47	220	55	232	62	252
	Assigned sequences	2408	28,531	3929	34,213	4056	49,325
	% of assigned sequences	97.45	84.98	95.71	81.68	88.27	82.49
	Shared taxa between thresholds	47	—	55	—	62	—
	Unique taxa for 80%	—	173	—	177	—	190
Genus	Number of taxa	73	577	88	623	143	749
	Assigned sequences	2403	27,583	3867	33,057	3879	47,573
	% of assigned sequences	97.25	82.15	94.20	78.92	84.42	79.56
	Shared taxa between thresholds	73	—	88	—	143	—
	Unique taxa for 80%	—	504	—	535	—	606
Species	Number of taxa	168	1715	212	1848	328	2285
	Assigned sequences	2471	33,573	4105	41,881	4595	58,314
	% of assigned sequences	100.00	99.99	100.00	99.99	100.00	97.53
	Shared taxa between thresholds	168	—	212	—	328	—
	Unique taxa for 80%	—	1547	—	1636	—	1957

**FIGURE 2:**
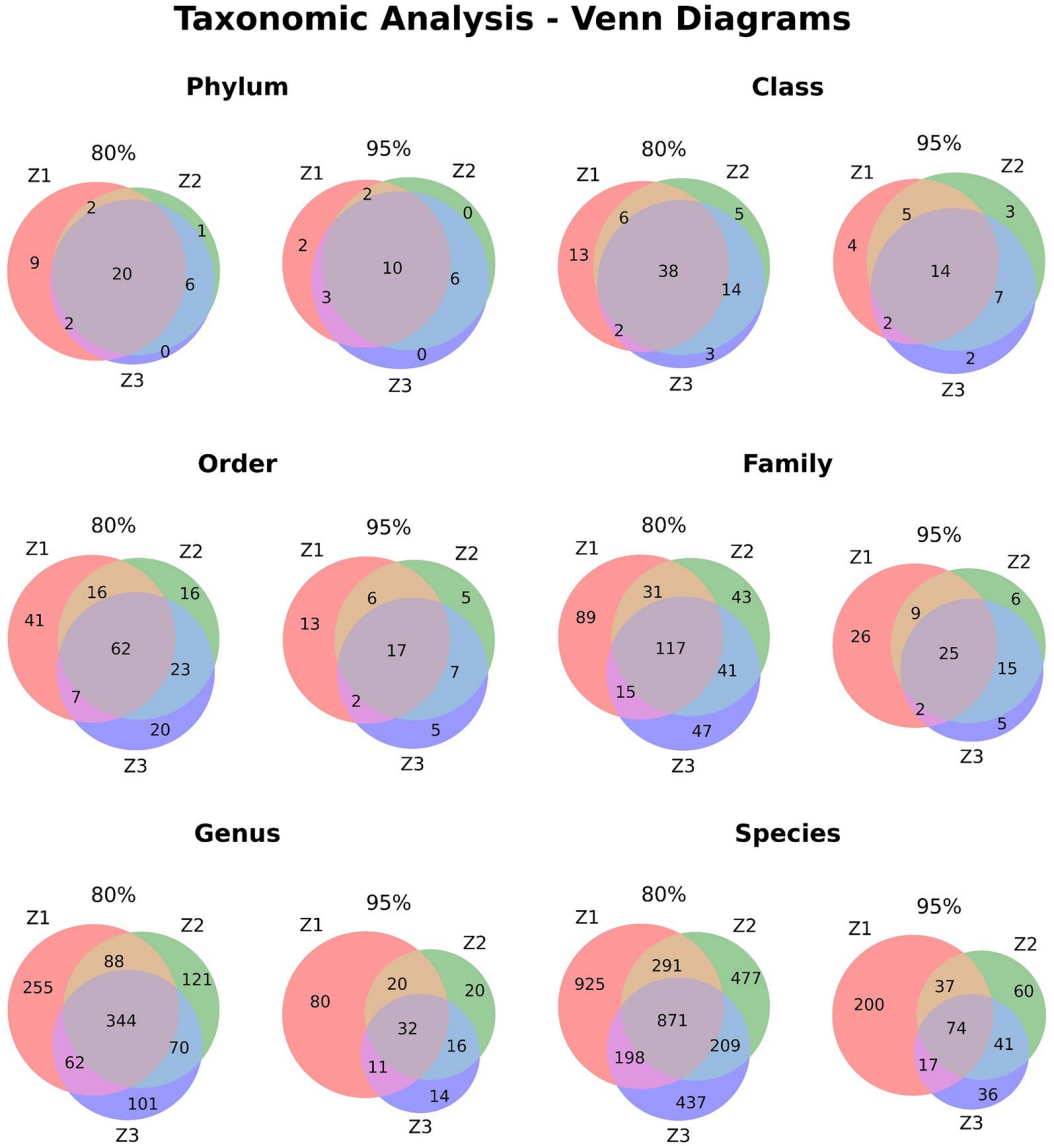
Venn diagram comparison of taxonomic overlap between samples Z1, Z2, and Z3 at 80% and 95% sequence identity thresholds across taxonomic levels.

A quantitative comparison of taxonomic overlap between samples Z1, Z2, and Z3 was presented in the table within Figure 2, at both 80% and 95% sequence identity thresholds. The table reports key metrics for each taxonomic level, including the total number of unique taxa across all samples, the taxa common to all three samples, and the individual taxonomic counts for each sample. This comparison is visually represented in Figure 2, which uses Venn diagrams to illustrate the distribution and overlap of taxa between the samples at each taxonomic level.

The comparative analysis of taxonomic overlap reveals significant differences in microbial community composition between the 80% and 95% sequence identity thresholds. At the 80% threshold, a substantially greater number of taxa were identified across all taxonomic levels compared to the 95% threshold. The most notable differences were observed at the species level (3408 unique taxa at 80% vs. 465 unique taxa at 95%) and the genus level (1041 unique taxa at 80% vs. 193 unique taxa at 95%). Additionally, the number of taxa common to all three samples was consistently higher at the 80% threshold, indicating that a broader core microbiome was detected using less stringent matching criteria (Table 3).

**TABLE 3:** Summary table quantifying the taxonomic distribution across samples at both thresholds, including total unique taxa, taxa common to all samples, and individual sample counts for each taxonomic level.

Taxonomic level	Identity threshold	Total unique taxa	Common in all samples	Z1 count	Z2 count	Z3 count
Phylum	80%	40	20	33	29	28
	95%	23	10	17	18	19
Class	80%	81	38	59	63	57
	95%	37	14	25	29	25
Order	80%	185	62	126	117	112
	95%	55	17	38	35	31
Family	80%	383	117	252	232	220
	95%	88	25	62	55	47
Genus	80%	1041	344	749	623	577
	95%	193	32	143	88	73
Species	80%	3408	871	2285	1848	1715
	95%	465	74	328	212	168

*Note:* Total Unique Taxa: The total number of unique taxa across all three samples. Common in all samples: The number of taxa found in all three samples. Z1, Z2, Z3 Count: The number of unique taxa in each sample.

Sample Z1 consistently exhibited the highest taxonomic richness at both thresholds across most taxonomic levels, particularly at the species level, where it identified 2285 taxa at an 80% sequence identity threshold compared to 328 taxa at a 95% sequence identity threshold. The Venn diagrams clearly illustrate that, while the 95% threshold results in fewer unique taxa per sample, the 80% threshold presents a more complex picture of both shared and sample specific microbial diversity.

This analysis demonstrates that sequence matching stringency significantly impacts the detected taxonomic composition and the apparent relationships between samples. While the 95% threshold provides higher confidence in taxonomic assignments, it may underestimate the true diversity and commonality between samples, potentially obscuring important ecological patterns in microbial communities.

The figure displays comparative Venn diagrams for each taxonomic level, ranging from Phylum to Species. On the left, the diagrams represent an 80% identity threshold, while the right side shows a 95% identity threshold. Each circle corresponds to a sample (Z1, Z2, or Z3), with numbers indicating the counts of unique and shared taxa between the samples. The taxonomic composition of microbial communities in samples Z1, Z2, and Z3 is presented in the table, which includes unique sequence identifiers, taxonomic classifications, and abundance metrics for each identified taxon. Only taxa with a minimum of 10 sequences (Count ≥ 10) are included in this table (Table S1).

To evaluate the impact of the sequence identity threshold on detecting variability between samples, we conducted Kruskal‐Wallis tests to compare samples Z1, Z2, and Z3 at each taxonomic level, using both 80% and 95% thresholds (see Table 4). This non‐parametric statistical method allowed us to determine whether the taxonomic differences observed among the samples were statistically significant (*p* < 0.05).

**TABLE 4:** Statistical comparison of taxonomic composition between samples Z1, Z2, and Z3 at different sequence identity thresholds.

Identity threshold	Taxonomic level	Kruskal‐Wallis (H)	*p*	Significant
80%	Phylum	0.2585	0.8788	No
80%	Class	0.6067	0.7383	No
80%	Order	0.0450	0.9778	No
80%	Family	0.3128	0.8552	No
80%	Genus	11.6245	0.0030	Yes
80%	Species	29.9248	3.2 × 10^−7^	Yes
95%	Phylum	0.8734	0.6462	No
95%	Class	0.1351	0.9347	No
95%	Order	0.3459	0.8412	No
95%	Family	2.5374	0.2812	No
95%	Genus	37.9491	5.7 × 10^−9^	Yes
95%	Species	63.8668	1.4 × 10^−14^	Yes

*Note:* The Kruskal‐Wallis test was performed to determine statistically significant differences between samples at each taxonomic level. A *p* < 0.05 indicates significant differences.

At the 80% threshold, significant differences between samples were observed at two taxonomic levels: Genus (*H* = 11.6245, *p* = 0.0030) and Species (*H* = 29.9248, *p* = 3.2 × 10^−7^). In contrast, at the 95% threshold, significant differences were also detected at the same two taxonomic levels, with particularly strong differences at the Genus (*H* = 37.9491, *p* = 5.7 × 10^−9^) and Species (*H* = 63.8668, *p* = 1.4 × 10^−14^) levels. No significant differences were found at the Phylum, Class, Order, or Family levels at either threshold. These findings indicate that, regardless of the threshold applied, statistically significant differences between samples consistently emerged at the Genus and Species levels, suggesting a reproducible and threshold‐independent pattern of taxonomic divergence confined to the lower levels of classification.

### 2.2 Taxonomic Composition Analysis Across Classification Levels

#### 2.2.1 Taxonomic Composition at Different Sequence Identity Thresholds

The analysis of taxonomic composition across all major taxonomic levels (Phylum, Class, Order, Family, Genus, and Species) at both 80% and 95% sequence identity thresholds revealed significant differences in community structure and diversity.

Importantly, the 80% threshold appears to mask certain taxa that become visible at the 95% threshold while simultaneously overrepresenting others. This differential detection significantly impacts the characterization of the microbiome. For example, Ascomycota (4.3%) is completely absent in the 80% analysis (likely falling below the 1% reporting threshold), while Spirochaetota (25.2%) is prominent at 80% but absent at 95%. These substantial differences demonstrate how threshold selection can fundamentally alter our understanding of microbial community composition.

At the Phylum level, Bacteroidota shows a dramatic difference between thresholds, accounting for only 25.9% at 80% but dominating at 59.6% at the 95% threshold. This represents a 33.7% difference, emphasizing how threshold selection can significantly shift the perceived dominance patterns of major phyla. Conversely, Bacillota and Actinomycetota both show reduced representation at higher thresholds (39.7% vs. 31.7% and 6.6% vs. 1.5%, respectively).

The Class‐level analysis revealed even more pronounced threshold‐dependent variations. Bacteroidia shows a 34.7% difference between thresholds (26.7% at 80% vs. 61.4% at 95%). Three classes present at > 1% in the 80% threshold were undetectable at 95%: Spirochaetia (26.5%), Erysipelotrichia (3.9%), and Negativicutes (1.0%). Conversely, Saccharomycetes (3.1%) was uniquely detected at the 95% threshold. Such substantial shifts in class representation demonstrate how threshold choice can profoundly influence our understanding of microbial community structure.

At the Order level, similar patterns emerged with Bacteroidales showing a 34.6% difference between thresholds. Four orders present at > 1% in the 80% threshold were undetectable at 95%, including Spirochaetales (26.6%), while three orders including Lactobacillales (4.1%) and Monoglobales (3.8%) were uniquely detected at 95%.

Family‐level composition revealed the most dramatic differences, with Bacteroidaceae showing a 40.3% difference between thresholds (11.2% at 80% vs. 51.5% at 95%). Remarkably, 10 families detected at > 1% in the 80% threshold were absent at 95%, including Spirochaetaceae (26.4%) and Odoribacteraceae (6.7%), while 5 families including Monoglobaceae (3.9%) and Streptococcaceae (3.4%) were uniquely detected at 95%.

Genus‐level analysis showed that *Spirochaeta*, the predominant genus at 80% (26.3%), was completely absent at 95%. Meanwhile, Phocaeicola and Bacteroides showed dramatically higher abundances at 95% (26.0% vs. 3.6% and 26.7% vs. 7.4%, respectively). Nine genera present at > 1% in the 80% threshold were undetectable at 95%, while five genera were uniquely detected at the 95% threshold.

At the Species level, which provides the most detailed taxonomic resolution, *Spirochaeta* sp. canine oral taxon 379 dominated at 80% (21.3%) but was completely absent at 95%. Conversely, *Phocaeicola vulgatus* (16.5%) and *Bacteroides uniformis* (15.9%) dominated at 95% but showed minimal presence at 80% (1.6% and 1.8%, respectively). A total of 9 species present at > 1% in the 80% threshold were undetectable at 95%, while 11 species including *Phocaeicola dorei* (4.2%) and *Monoglobus pectinilyticus* (3.7%) were uniquely detected at 95%.

These findings demonstrate that threshold selection substantially impacts microbial community characterization, with the 95% threshold generally revealing a more diverse community structure with different dominant taxa compared to the 80% threshold. The fact that many taxa can be completely missed or significantly under/overrepresented depending on threshold choice highlights the importance of careful parameter selection in microbiome studies, as these differences can lead to fundamentally different biological interpretations of the same sample.

#### 2.2.2 Comparative Analysis of Taxa Richness Between Sequence Identity Thresholds

**FIGURE 3:**
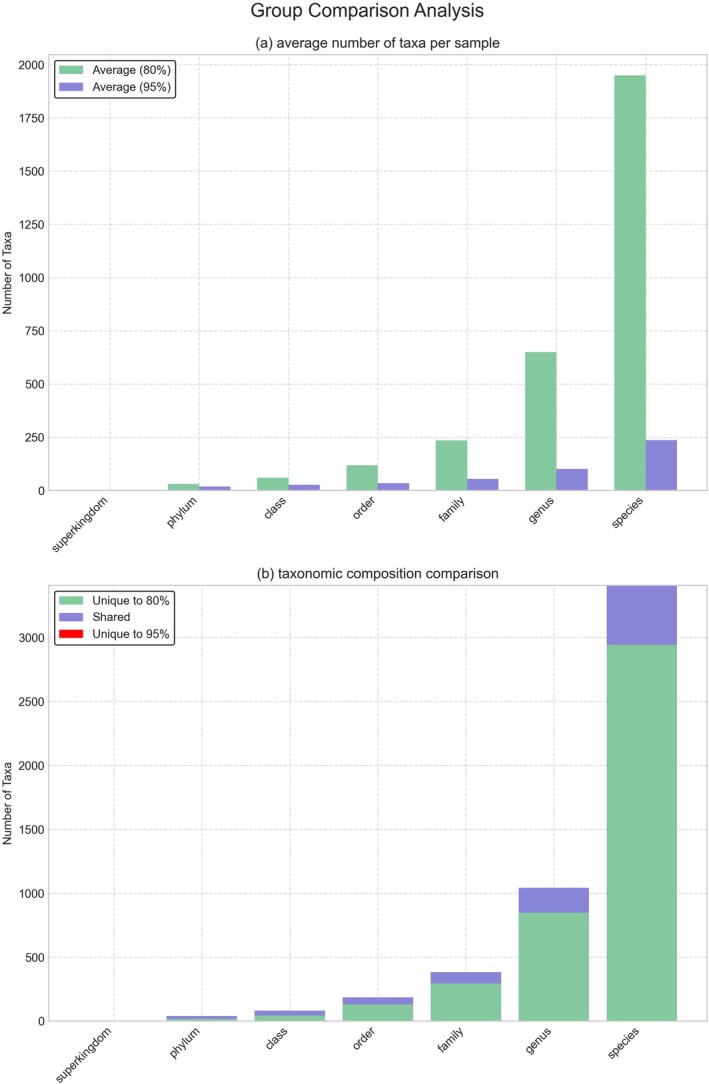
Group comparison analysis of taxa detected at 80% and 95% sequence identity thresholds across taxonomic levels. (a) Average number of taxa per sample at each taxonomic level. Green bars represent the 80% threshold, and purple bars represent the 95% threshold. (b) Taxonomic composition comparison showing the distribution of taxa across different taxonomic levels. Each bar is divided into three components: Taxa unique to the 80% threshold (green), taxa shared between both thresholds (purple), and taxa unique to the 95% threshold (red). (The supplementary data for this figure can be found in Table S2).

To further investigate the impact of sequence identity thresholds on microbial community characterization, we compared the number and distribution of taxa detected at 80% and 95% thresholds across all taxonomic levels from superkingdom to species (Figure 3). This analysis revealed striking differences in taxonomic richness and composition between the two thresholds.

As illustrated in Figure 3a, the average number of taxa per sample was consistently higher at the 80% threshold across all taxonomic levels. This difference became more pronounced as the taxonomic levels became more specific. At the species level, samples analysed with the 80% threshold contained an average of about 2000 taxa per sample, whereas those processed at the 95% threshold had only around 200 taxa per sample. This represents nearly a 10‐fold difference in detected diversity.

The comparison of taxonomic composition (Figure 3b) offers additional insights into the relationship between taxa identified at both thresholds. While there was a core set of taxa shared between the two thresholds across all taxonomic levels, the 80% threshold revealed a significantly larger number of unique taxa that were not detected at the 95% threshold. This distinction was particularly pronounced at the species level, where approximately 3000 taxa were uniquely identified at the 80% threshold, compared to only around 400 unique taxa at the 95% threshold.

Interestingly, the number of shared taxa remained relatively stable across most taxonomic levels, suggesting that the 95% threshold primarily captures a core subset of the microbial community that is also detected at the 80% threshold. The comparatively small number of taxa unique to the 95% threshold indicates that higher stringency rarely results in novel taxonomic assignments not captured by lower stringency analysis.

**FIGURE 4:**
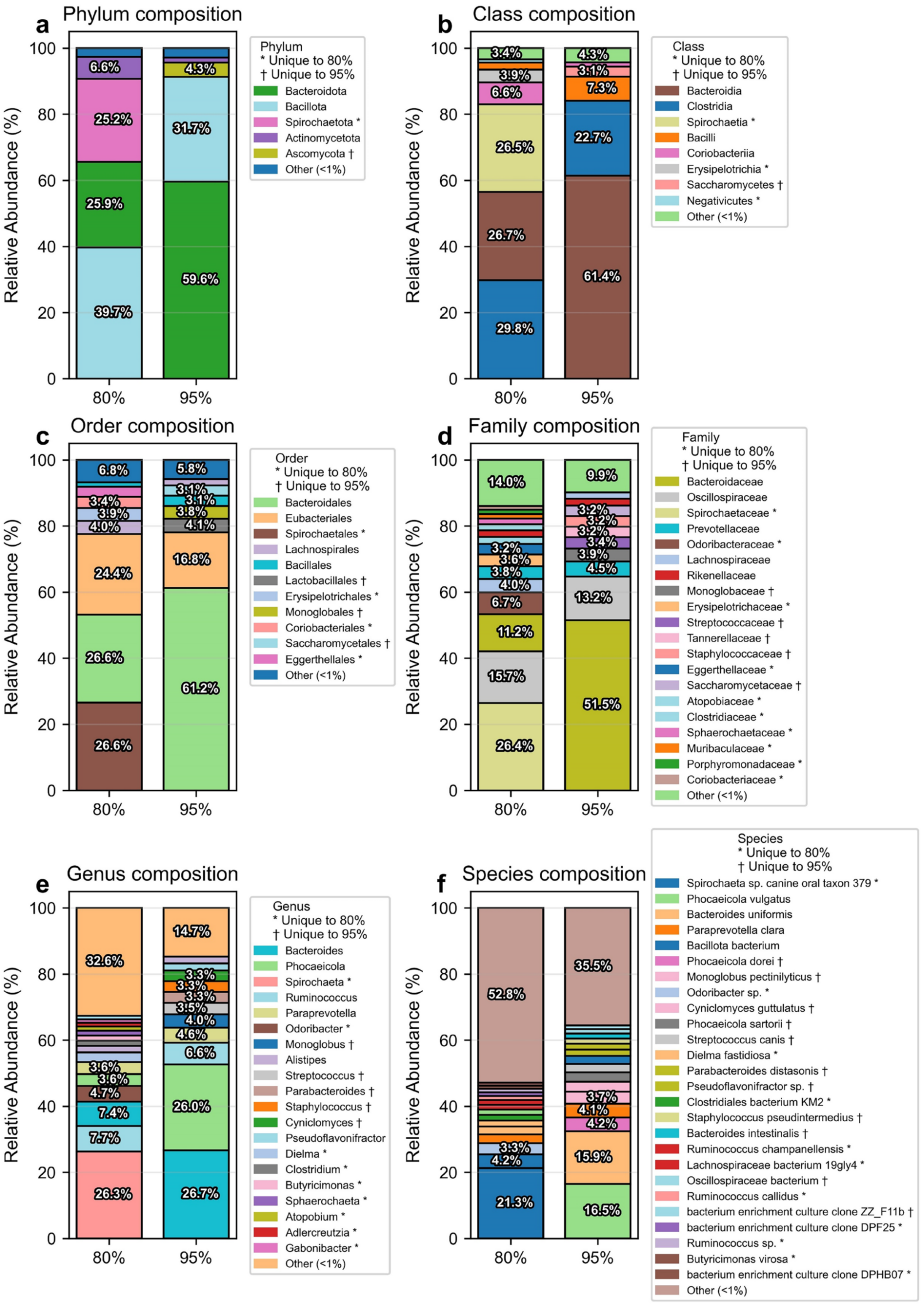
Taxonomic composition analysis at 80% and 95% sequence identity thresholds across six taxonomic levels. Symbols * and † indicate taxa unique to 80% threshold and 95% threshold, respectively. (a) Family composition showing the relative abundance (%) of dominant families at both 80% threshold (left) and 95% threshold (right). (b) Class composition illustrating the distribution of bacterial classes at both sequence identity thresholds. (c) Order composition comparing the relative abundances of bacterial orders between 80% and 95% thresholds. (d) Genus composition displaying the distribution of bacterial genera with different sequence matching stringencies. (e) Phylum composition showing the major bacterial phyla detected at both sequence identity thresholds. (f) Species composition comparing the distribution of bacterial species between 80% and 95% thresholds. Each panel presents paired stacked bar charts with 80% threshold data (left) and 95% threshold data (right). Taxa contributing less than 1% to overall community composition are grouped as “Other (< 1%)”. NCBI Taxonomy IDs is provided for species in parentheses. Colour coding is consistent within each taxonomic level to facilitate comparison between threshold values.

These findings complement our previous taxonomic composition and statistical analyses, demonstrating that while the 95% threshold provides higher confidence in taxonomic assignments and better discriminatory power for detecting inter‐sample differences, it does so at the cost of significantly reduced taxonomic breadth, potentially missing thousands of taxa that are detected at lower stringency thresholds. It should be noted that taxa reported exclusively at the 80% threshold are labelled by their closest database reference and may represent novel or uncharacterized lineages rather than the named species itself. In addition to richness‐based comparisons, the relative abundance profiles of dominant taxa across phylum, class, order, family, genus, and species levels are presented in Figure 4.

#### 2.2.3 Comparison With Previously Published Microbiome Studies

**FIGURE 5:**
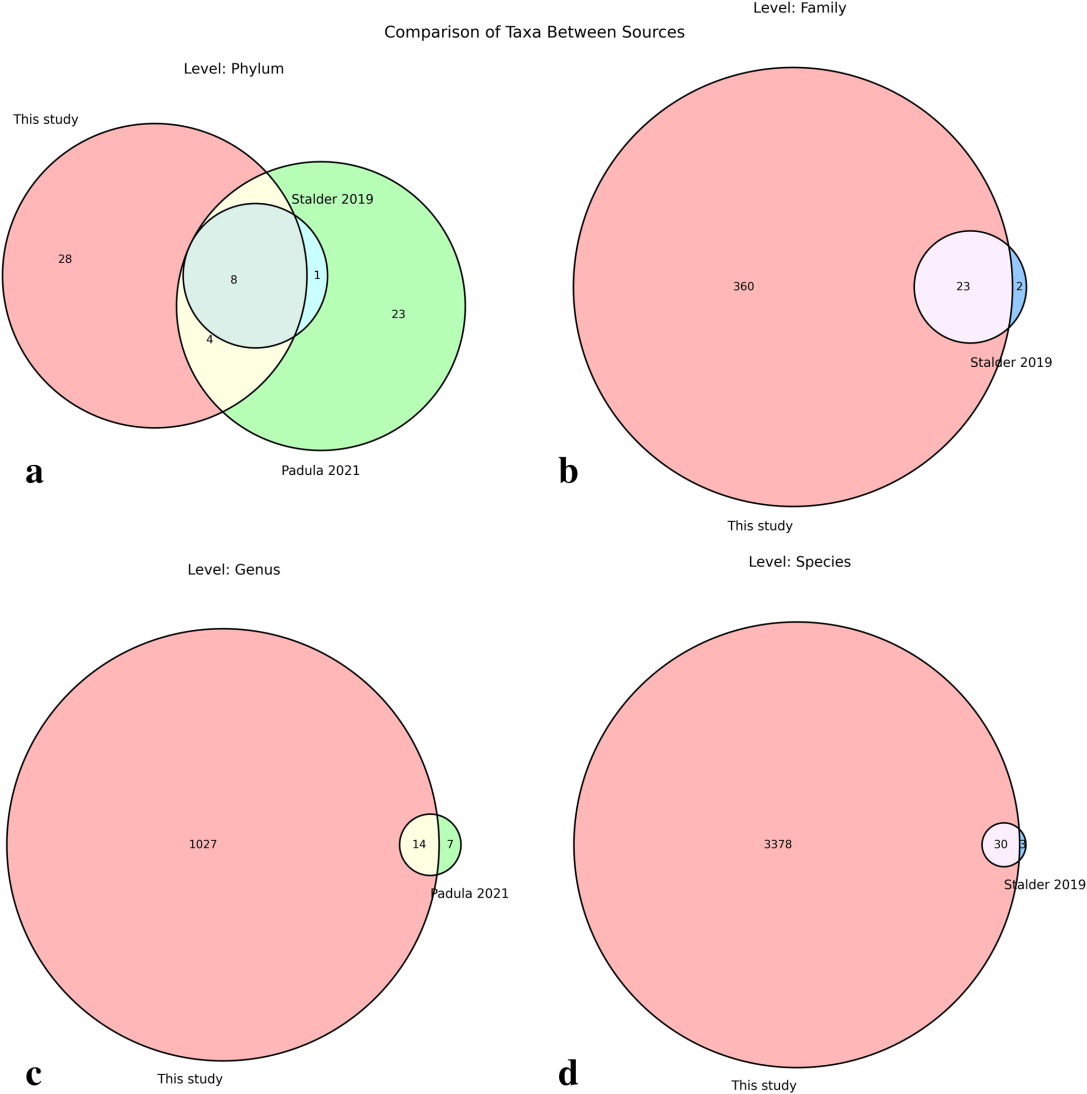
Comparison of taxonomic composition across different studies of similar microbiomes. Venn diagrams comparing the taxonomic overlap at four levels: (a) Phylum—comparison between this study (red), Stalder et al. (2019) (blue), and Padula et al. (2021) (green); (b) Family—comparison between this study and Stalder et al. (2019); (c) Genus—comparison between this study and Padula et al. (2021); (d) Species—comparison between this study and Stalder et al. (2019). Numbers represent unique taxa counts.

To evaluate the comprehensiveness of our taxonomic characterization approach, we compared the taxa identified in our study with those reported in two previous microbiome studies: a Stalder et al. (2019) publication in Scientific Reports and a Padula et al. (2021) publication in Biology (Figure 5). This comparative analysis was performed across four taxonomic levels (Phylum, Family, Genus, and Species) to assess the extent of taxonomic overlap and the identification of taxa. It should be noted that this comparison is subject to important limitations: the studies differ substantially in sequencing platform (Illumina short‐read vs. Oxford Nanopore long‐read in our study), target gene region (partial vs. near‐full‐length 16S rRNA), bioinformatic pipeline, reference database, geographic origin of the sampled hares, and dietary context. Consequently, the observed differences in taxonomic richness reflect a combination of true biological variation and methodological factors, and should not be attributed solely to biological differences in microbiome composition.

At the phylum level (Figure 5A), our analysis identified 40 phyla, of which 28 (70%) were unique to our study. We shared 8 phyla with the Stalder et al. (2019) study (Stalder et al. (2019)) and 12 phyla with the Padula et al. (2021) study (Padula et al. (2021)), while 1 phyla was exclusively shared between the two reference studies.

The differences became more pronounced at higher taxonomic resolutions. At the family level (Figure 5B), our analysis identified 360 unique families compared to only one unique family in Stalder et al. (2019) study.

The genus‐level comparison (Figure 5C) revealed even more striking differences, with our study identifying 1027 unique genera with 7 unique to Biology study.

At the species level (Figure 5D), the divergence was most dramatic, with our analysis identifying 3373 unique species. The Stalder et al. (2019) study identified only one unique species.

The missing taxa report (Table 5) identified 37 taxa across all taxonomic levels that were reported in previous studies but not detected in our analysis. Of these, 21 taxa (56.8%) lacked defined entries in the NCBI taxonomy database. These undefined taxa, described using non‐standard nomenclature, include 12 phyla from the Padula et al. (2021) publication and 1 phyla from publication Stalder et al. 2019, 1 family from publication Stalder et al. (2019) and 6 genera from Padula et al. (2021).

**TABLE 5:** Taxa with NCBI database entries identified in previous publications but absent from the current dataset.

Lp	Level	Source	NCBI name	Published name	NCBI tax ID
1	Phylum	Padula et al. (2021)	Armatimonadota	Armatimonadetes	67,819
2	Phylum	Padula et al. (2021)	Chlamydiota	Chlamydiae	204,428
3	Phylum	Padula et al. (2021)	Campylobacterota	Epsilonbacteraeota	29,547
4	Phylum	Padula et al. (2021)	Methanobacteriota	Euryarchaeota	28,890
5	Phylum	Padula et al. (2021)	Gemmatimonadota	Gemmatimonadetes	142,182
6	Phylum	Padula et al. (2021)	Kiritimatiellota	Kiritimatiellaeota	134,625
7	Phylum	Padula et al. (2021)	Nanobdellota	Nanoarchaeota	192,989
8	Phylum	Padula et al. (2021)	Candidatus Omnitrophota	Omnitrophicaeota	67,812
9	Phylum	Padula et al. (2021)	Planctomycetota	Planctomycetes	203,682
10	Phylum	Padula et al. (2021)	Nitrososphaerota	Thaumarchaeota	651,137
11	Phylum	Padula et al. (2021)	Verrucomicrobiota	Verrucomicrobia	74,201
12	Family	Stalder et al. (2019)	Brucellaceae	Brucellaceae	118,882
13	Genus	Padula et al. (2021)	Candidatus Saccharimonas	Candidatus Saccharimonas	1,331,051
14	Species	Stalder et al. (2019)	*Sphingobacterium wenxiniae*	*Sphingobacterium wenxiniae*	683,125
15	Species	Stalder et al. (2019)	*Selenomonas dianae*	*Selenomonas dianae*	135,079
16	Species	Stalder et al. (2019)	Kyrpidia tusciae	Kyrpidia tusciae	33,943

*Note:* The table shows representative taxa with valid NCBI taxonomy entries that were reported in previous studies but not detected in our analysis. The full report identified 40 missing taxa, of which 19 (47.5%) lacked defined entries in the NCBI taxonomy database. The list of taxa found in reference sources but missing in the current study is provided in Table S3.

Beyond the dominant phyla *Firmicutes* and *Bacteroidota*, our data revealed several taxa not previously reported in the brown hare gut. *Monoglobus pectinilyticus* is a specialist degrader of pectin, a major component of plant cell walls, and its presence is consistent with the herbivorous diet of hares. *Phocaeicola dorei* and *Phocaeicola vulgatus* are saccharolytic anaerobes with a broad capacity for polysaccharide utilization; they may contribute to short‐chain fatty acid (SCFA) production, supporting intestinal epithelial health. Members of the genus *Odoribacter* are butyrate producers, potentially beneficial in maintaining gut homeostasis, while *Alistipes* spp. are associated with the degradation of complex dietary glycans but can also act as opportunists under dysbiotic conditions. Detection of *Staphylococcus pseudintermedius* and *Streptococcus canis* at low abundance is noteworthy, as both are opportunistic pathogens typically associated with companion animals, suggesting possible environmental spillover.

To enhance the detailed taxonomic composition provided in the Supporting Information, we selected 30 representative taxa that are particularly significant due to their novelty in the microbiome of lagomorphs, their ecological roles, or their potential relevance to One Health. These microorganisms exemplify the functional diversity of the brown hare gut microbiome, including specialized fibre degraders, butyrate producers, and opportunistic pathogens with zoonotic potential. Their presence in hares offers new insights into the adaptation of lagomorph gut ecosystems and emphasizes potential pathways of microbial exchange between wild, farm animals and humans.

A summary of these taxa is presented in Table 6.

**TABLE 6:** Unique taxa identified in the brown hare gut microbiome and their ecological functions relevant to one health.

Taxon	Ecological/functional role	One health relevance	Significance in lagomorphs	References
*Monoglobus pectinilyticus*	Specialist degrader of pectins and complex plant polysaccharides; produces short‐chain fatty acids (SCFAs), mainly acetate, modulating epithelial barrier integrity and energy metabolism.	Humans: involved in colonic fermentation of dietary fibre. Livestock (cattle, sheep, goats): pectin degradation central to rumen fermentation.	First record in lagomorphs; indicates adaptation of hares to high‐fibre wild diets and microbial specialization in the caecum.	(Kim et al. 2019; Kim et al. 2023)
*Phocaeicola vulgatus*	Prominent carbohydrate degrader; produces SCFAs (acetate, propionate); lipopolysaccharide (LPS) signalling modulates immunity.	Common in humans and dogs; implicated in inflammatory bowel disease (IBD); also present in pigs.	Detection in hares suggests environmental sharing of taxa between wildlife, livestock, and humans; potential influence on hare gut immunity.	(Wexler 2007; Jin et al. 2024)
*Phocaeicola dorei*	Carbohydrate and bile acid metabolism; produces propionate regulating hepatic gluconeogenesis.	Humans: linked to cardiovascular risk modulation. Detected in rodents and companion animals.	Presence in hares expands host range; may act as a reservoir of bacteria implicated in chronic human disease.	(Jin et al. 2024)
*Bacteroides uniformis*	Produces SCFAs (acetate, propionate); supports epithelial health and immune balance; some strains with probiotic potential.	Humans: proposed for metabolic syndrome therapy. Dogs and pigs: detected in gut communities.	Presence in hares indicates beneficial taxon supporting fibre digestion; may enhance population resilience.	(Gómez del Pulgar et al. 2020)
*Odoribacter splanchnicus*	Butyrate‐producing anaerobe; immunoregulatory effects; also produces hydrogen sulfide (H_2_S).	Humans: protective role in colorectal cancer. Swine and rodents: present in gut ecosystems.	First report in lagomorphs; suggests role in colon health via butyrate production.	(Hiippala et al. 2020)
*Butyricimonas virosa*	Anaerobic fermenter producing butyrate; butyrate fuels colonocytes, strengthens tight junctions, and downregulates inflammation.	Humans: commensal, occasionally bloodstream infections. Dogs and poultry: contributes to SCFA pools.	First detection in hares indicates expansion of butyrate‐producing guild, buffering seasonal fibre shifts.	(Sakamoto et al. 2009)
*Alistipes* spp.	Amino acid‐ and bile acid‐associated fermenters; produce succinate and propionate, short‐chain fatty acids (SCFAs) with context‐dependent roles in host immunity. May exert both protective and dysbiotic effects, influencing intestinal and systemic inflammation.	Humans: enriched in subsets of inflammatory bowel disease (IBD) and neuropsychiatric disorders; also part of healthy microbiota. Companion animals (cats, dogs): recurrent members of carnivore gut microbiota, with abundance influenced by diet and stress.	Detection in hares adds clinically important genus; may influence immune tone under wild diets.	(Parker et al. 2020; Dziarski et al. 2016)
*Paraprevotella clara*	Saccharolytic anaerobe capable of degrading hemicellulose and plant‐derived oligosaccharides; produces acetate and succinate that serve as substrates for secondary fermenters (e.g., propionate producers). Enhances metabolic cross‐feeding in the large intestine.	Humans and primates (e.g., macaques): enriched in high‐fibre diets and associated with efficient carbohydrate metabolism. Ruminants and other herbivores: phylogenetically allied pathways support plant‐fibre utilization.	First documentation in hares; expands saccharolytic diversity beyond canonical *Bacteroides* and *Prevotella*, reinforcing adaptability to heterogeneous wild plant glycans.	(Morotomi et al. 2009)
*Prevotella copri complex*	Prominent degrader of plant polysaccharides; produces succinate (a key intermediate for propionate formation), with clade‐level functional heterogeneity affecting host lipid and glucose metabolism; strain‐level differences can tip outcomes toward either eubiosis or low‐grade inflammation in the **caecum/colon**.	**Humans:** diet‐dependent, clade‐specific associations with metabolic health and inflammation; enriched on high‐fibre diets. **Ruminants/equids/swine:** typically enriched on forage‐based diets, shaping short‐chain fatty acid (SCFA) pools.	Presence in hares mirrors other herbivores and underscores diet‐driven assembly; strain/clade profiling is warranted to predict metabolic and immunological effects in lagomorph **caecum**.	(Tett et al. 2019)
*Ruminococcus champanellensis*	Strictly anaerobic **cellulolytic** bacterium assembling multi‐enzyme **cellulosomes** that initiate hydrolysis of crystalline cellulose; releases oligo−/monosaccharides fueling secondary fermenters and generates acetate/formate and H_2_, supporting syntrophy with methanogens in the **caecum**.	**Humans:** contributes to degradation of resistant fibres, expanding fermentative niches and SCFA production. **Herbivores (ruminants, rabbits, rodents):** pivotal for lignocellulose utilization and energy harvest from coarse forages.	In hares, indicates an efficient fibrolytic hub adapted to lignified wild plants; supports caecal fermentation networks and cross‐feeding that stabilize energy extraction.	(Chassard et al. 2012; Ze et al. 2012)
*Ruminococcus* spp. (sensu lato)	Anaerobic cellulolytic/hemicellulolytic guild that initiates degradation of crystalline cellulose, hemicelluloses and resistant starch in the **caecum and colon**; produces short‐chain fatty acids (SCFAs: acetate, butyrate) and H_2_, supporting syntrophy with methanogens; cellulosome‐like multi‐enzyme systems drive primary fibre hydrolysis and cross‐feeding to secondary fermenters.	**Humans:** *R. bromii* is a keystone degrader of resistant starch shaping SCFA pools and metabolic responses. **Livestock/Equids/Swine/Rabbits:** pivotal for fibre conversion efficiency, ruminal/caecal health and productivity; abundance is strongly diet‐responsive.	In hares, confirms classical fibrolytic hubs enabling efficient energy harvest from lignified wild forages; supports stable caecal fermentation networks under seasonal diet shifts.	(Chen et al. 2024; Rangarajan et al. 2022)
*Blautia wexlerae*	Produces acetate and butyrate; strengthens epithelial barrier; contributes to anti‐inflammatory balance.	**Humans:** inverse associations with obesity/type‐2 diabetes; oral administration improved metabolic and barrier markers in intervention/animal studies. **Companion animals/livestock:** prevalent commensal linked to intestinal health and eubiosis.	First detection in hares suggests the presence of beneficial butyrogenic Firmicutes that may stabilize barrier function and immune tone during energy‐limited, high‐fibre feeding.	(Hosomi et al. 2022; Liu, Mao, et al. 2021)
*Faecalibacterium prausnitzii*	One of the most abundant **butyrate** producers in the mammalian gut; converts complex polysaccharide‐derived substrates via cross‐feeding into butyrate that fuels colonocytes, tightens epithelial junctions, induces **IL‐10** and suppresses **NF‐κB** signalling; secretes **microbial anti‐inflammatory molecules (MAM)**; stabilizes luminal pH and oxygen tension in the **colon**.	**Humans:** consistently depleted in inflammatory bowel disease (IBD) and other chronic inflammatory states; higher abundance associates with eubiosis and improved metabolic markers. **Livestock/rodents:** abundance correlates with intestinal health and barrier integrity; experimental supplementation improves mucosal healing.	Detection in hares indicates conservation of the **SCFA–mucosa–immune** axis in lagomorphs; candidate biomarker of large‐intestine (colon/caecum) health and resilience under high‐fibre, seasonal diets.	(Martín et al. 2023; Sokol et al. 2008)
*Roseburia intestinalis*	Motile, strictly anaerobic **butyrate** producer; degrades resistant dietary fibres and reinforces the **mucus layer** and epithelial **tight junctions**; promotes immune tolerance and displays anti‐inflammatory effects; **extracellular vesicles (EVs)** from *R. intestinalis* mitigate colitis and restore barrier function in vivo; contributes to SCFA pools in the **colon** and **caecum**.	**Humans/rodents:** reduced in type 2 diabetes and IBD; increases with fibre/inulin‐rich diets; EVs alleviate colitis and modulate microbiota‐host crosstalk. **Herbivores (horses, rabbits):** important contributor to SCFA production in the large intestine; diet‐responsive across hindgut fermenters.	Presence in hares expands the butyrogenic guild and suggests a role in maintaining barrier integrity and immunological homeostasis during fluctuations in botanical fibre quality characteristic of wild foraging.	(Han et al. 2024; Tamanai‐Shacoori et al. 2017)
*Akkermansia muciniphila*	Obligate anaerobe specialized in **mucin** degradation in the colonic mucus layer; produces **short‐chain fatty acids (SCFAs)** (acetate, propionate) that fuel colonocytes and promote **mucus turnover**; modulates host immunity (e.g., dampening **NF‐κB** activation) and metabolic signalling (e.g., **GLP‐1**, **bile acid** receptors FXR/TGR5); supports barrier integrity via tight‐junction reinforcement in the **colon/caecum**.	**Humans:** consistently reduced in obesity, type 2 diabetes and inflammatory bowel disease; administration (including pasteurized preparations) improves metabolic and barrier markers in clinical/animal studies. **Companion animals (dogs/cats):** higher abundance associates with leanness and better gut barrier metrics; **livestock:** emerging evidence for roles in barrier and metabolic homeostasis.	First detection in brown hares indicates a conserved **mucin‐degrading niche** within lagomorphs, suggesting contributions to mucus‐layer renewal and immunometabolic homeostasis during seasonal diet shifts.	(Depommier et al. 2019; Khalili et al. 2024)
*Phascolarctobacterium succinatutens*	Specialist **succinate‐utilizing** anaerobe that rapidly converts succinate (a major product of primary saccharolytic bacteria) into **propionate**; thereby shapes SCFA pools, lowers luminal succinate (linked to dysbiosis/inflammation when excessive), and stabilizes redox balance in the **caecum/colon**; weak/absent growth on sugars—metabolic niche coupling to upstream fibre degraders.	**Humans:** abundance tracks “**succinotype**” microbiome states and associates with favourable lipid/glucose profiles in diet‐intervention cohorts. **Swine/rodents:** higher levels correlate with improved metabolic resilience and lower adiposity; presence reported in healthy canine gut as part of SCFA networks.	Detection in hares indicates an operative **succinate→propionate** axis, enhancing energy recovery from plant fermentation and buffering succinate accumulation that could impair epithelial function.	(Anthamatten et al. 2024; Wu et al. 2011)
*Desulfovibrio piger*	Sulfate‐reducing bacterium (SRB); reduces sulfate to **hydrogen sulfide (H**_**2**_ **S)**, a metabolite influencing redox balance and mucosal health; moderate H_2_S contributes to microbial cross‐feeding, but excess damages epithelial mitochondria and barrier integrity in the **colon**.	**Humans:** enriched in ulcerative colitis and irritable bowel syndrome; H_2_S implicated in pro‐inflammatory conditions. **Rodents/pigs:** increased abundance correlates with colitis‐like pathology.	First detection in hares highlights active **sulfur cycling** in the caecum; suggests vulnerability to redox imbalance under high‐sulfate or protein‐rich forage.	(Singh et al. 2023; Wegmann et al. 2017)
*Methanobrevibacter smithii*	Dominant **archaeal methanogen**; consumes H_2_ and CO_2_ to produce methane, maintaining low H_2_ partial pressure and improving fermentation efficiency in the **caecum and colon**.	**Humans:** major gut methanogen; higher levels correlate with constipation and altered transit times. **Ruminants:** central player in enteric methane emissions.	First detection in hares underscores syntrophic links between fibrolytic bacteria and methanogens, improving energy harvest but potentially contributing to methane release in wild lagomorphs.	(Malat et al. 2024; Moissl‐Eichinger et al. 2018; Borrel et al. 2020)
*Gordonibacter urolithinfaciens*	Specialist degrader of **ellagic acid** into **urolithins**, metabolites with antioxidant, anti‐inflammatory, and estrogenic activity; urolithins regulate gut‐liver axis and mitochondrial health.	**Humans:** linked to cardiometabolic benefits (e.g., reduced risk of atherosclerosis). **Rodents:** supplementation improves barrier and metabolic outcomes.	First detection in hares suggests processing of ellagitannins from wild plants; indicates a role in bioactive polyphenol metabolism in lagomorphs.	(Selma et al. 2014)
*Gabonibacter* sp.	Anaerobic member of the family Porphyromonadaceae associated with the gut microbiota; metabolic characterization of the genus remains limited.	**Humans:** members of the genus have been isolated from faecal samples and are considered part of the rare gut‐associated bacterial diversity.	Detection of a Gabonibacter‐assigned taxon in hares expands current knowledge of poorly characterized Porphyromonadaceae lineages in wildlife gut ecosystems.	(Mourembou et al. 2016)
*Sphaerochaeta pleomorpha*	Free‐living anaerobe with unusual cell morphology; ferments carbohydrates to acetate and ethanol; genome encodes atypical spirochaete metabolic pathways.	**Humans/rodents:** rare in gut but occasionally recovered in low abundance. **Environmental sources:** identified in sediments and anaerobic digesters.	Highly abundant in hare gut microbiota, Sphaerochaeta spp. contribute to carbohydrate fermentation in the hindgut. Their presence indicates a reservoir of uncultured, non‐pathogenic Spirochaetes potentially supporting dietary adaptation and energy recovery from fibrous plant material.	(Ritalahti et al. 2012; Stalder et al. 2019)
*Spirochaeta* spp. (non‐pathogenic)	Obligate anaerobes utilizing polysaccharides (xylans, cellulose derivatives) and producing acetate, ethanol, and H_2_; contribute to fibrolytic consortia in the **caecum** of herbivores.	**Herbivores (ruminants, horses):** key contributors to plant fibre turnover in large‐intestine fermentation. **Humans:** rare, non‐pathogenic spirochaetes occasionally detected in stool.	In European brown hares, Spirochaetes—dominated by Sphaerochaetaceae—are significantly enriched compared with rabbits, with prevalent OTUs related to Sphaerochaeta spp. (e.g., *S. globosa* , *S. pleomorpha* ). The low 16S rRNA identity to cultured references suggests hare‐adapted, uncultured lineages rather than pathogens, underscoring the ecological uniqueness of lagomorph gut communities.	(Caro‐Quintero et al. 2012)
*Petroclostridium xylanilyticum*	Strictly anaerobic Firmicute specialized in degrading **xylan** (a hemicellulose) into oligosaccharides, acetate, and butyrate; initiates breakdown of plant cell‐wall polysaccharides, fueling cross‐feeding chains in the **caecum**.	**Herbivores:** isolated from rumen and equine caecum; enhances fibre utilization and SCFA production. **Humans:** low‐abundance member of gut consortia, potentially linked to dietary fibre metabolism.	First detection in hares highlights their capacity to exploit xylan‐rich forages typical of grasses and shrubs.	(Chassard et al. 2010; Zhang et al. 2018)
*Harryflintia acetispora*	Anaerobic fermenter; produces acetate and spores; phylogenetically related to Lachnospiraceae.	Humans: rarely detected; ecological role unclear.	First‐time in hares; suggests unexplored acetate producers in wildlife.	(Petzoldt et al. 2016)
*Candidatus Stoquefichus/Oscillospira‐related lineage*	Uncultured gut‐associated lineage related to Oscillospira/Oscillospiraceae, inferred from metagenomic studies to participate in fermentative metabolism and short‐chain fatty acid production.	**Humans:** identified in metagenomes, linked to butyrate supply and metabolic health. **Ruminants/rodents:** detected across herbivores, indicating conserved role in fibre fermentation.	Detection in hares expands representation of uncultured butyrate producers; underscores cryptic but functionally relevant members of lagomorph gut consortia.	(Gophna et al. 2017)
*Streptococcus canis*	Gram‐positive coccus, opportunistic pathogen; commensal of the skin, oral cavity, and intestine of carnivores. Produces virulence factors such as **M‐like protein (SCM)**, streptolysins, and adhesins. Capable of causing bacteremia, necrotizing fasciitis, and streptococcal toxic shock‐like syndrome.	**Dogs and cats:** frequent cause of dermatitis, otitis, septicemia, and neonatal infections. **Humans:** zoonotic infections via bites, scratches, or close contact; cases include sepsis and endocarditis. **Livestock:** sporadic infections in cattle and swine.	First detection in hares underscores their potential as reservoirs of opportunistic zoonotic *Streptococcus*. May reflect spillover from carnivores in overlapping habitats.	(Galpérine et al. 2007; Cucco et al. 2025)
*Staphylococcus pseudintermedius*	Coagulase‐positive **staphylococcus**; colonizes skin and mucosa of dogs and cats. Produces toxins (enterotoxins, leukotoxins, exfoliative toxins) and biofilm. Multidrug resistance (notably **methicillin‐resistant *S. pseudintermedius* , MRSP**) is a growing concern.	**Dogs:** major cause of pyoderma, otitis externa, and postoperative wound infections. **Cats:** less frequent but relevant. **Humans:** zoonotic infections reported in veterinarians, pet owners; include wound infections and bacteremia. **Livestock:** rare but occasionally isolated.	Detection in hares indicates possible environmental acquisition and adds a wildlife reservoir dimension to the epidemiology of MRSP and related clones	(Somayaji et al. 2016; Morais et al. 2023)
Odoribacteraceae (family)	Family of anaerobic Bacteroidota; includes genera (*Odoribacter*, *Gabonibacter*, *Butyricimonas*). Members produce **butyrate and other SCFAs**, modulate bile acid metabolism, and contribute to anti‐inflammatory networks in the **colon/caecum**.	**Humans:** depletion linked with inflammatory bowel disease and metabolic syndrome; butyrate‐producing potential supports barrier integrity. **Livestock (swine, ruminants):** associated with feed efficiency and resilience to enteric dysbiosis. **Companion animals:** detected in dog and cat gut consortia.	Expansion of *Odoribacteraceae* in hares demonstrates a diverse butyrogenic guild, previously underestimated in lagomorph gut ecology.	(Vacca et al. 2020; García‐López et al. 2019)
Monoglobaceae (family)	Family comprising specialist degraders of **pectins** and other complex plant cell‐wall polysaccharides (e.g., genus *Monoglobus*). Members encode extensive **carbohydrate‐active enzymes (CAZymes)** for pectin deconstruction (polygalacturonases, pectate lyases) and produce **short‐chain fatty acids (SCFAs)**—predominantly **acetate**—that fuel colonocytes and support cross‐feeding to butyrate producers in the **caecum/colon**.	**Humans:** family‐level enrichment on pectin‐rich diets; acetate output linked to improved mucosal energy balance and barrier function. **Herbivores/livestock:** functional analogues enhance utilization of fruit/forage pectins, contributing to feed efficiency and stable SCFA pools.	Detection in hares documents a **pectinolytic guild** adapted to wild plant substrates, supporting energy harvest from soft tissues of forbs/shrubs and expanding the known host range of pectin‐degrading consortia in lagomorphs.	(Kim et al. 2017; Kim et al. 2019)
Prevotellaceae (family)	Dominant **saccharolytic** family in plant‐rich diets; degrades **starches, xylans, hemicelluloses** and host/dietary glycans to **succinate, acetate, propionate**. Exhibits trophic specialization (primary saccharolysis, succinate overflow) that feeds **propionate‐producing** taxa and shapes SCFA pools in the **caecum and colon**; marked **strain/clade heterogeneity** in immunometabolic outputs.	**Humans:** enrichment on high‐fibre, non‐westernized diets; in some contexts linked to low‐grade inflammation, in others to metabolic benefits—effects are **clade‐specific**. **Ruminants/equids/swine:** typically abundant under forage‐based feeding, associated with fibre conversion efficiency and nitrogen salvage.	n hares, robust Prevotellaceae signal indicates **diet‐driven assembly** optimized for heterogeneous wild plant glycans; supports succinate‐centred cross‐feeding that enhances propionate formation and energy extraction in lagomorph large intestine.	(Chen et al. 2021; Rojas et al. 2021).

Abbreviations: AMR, antimicrobial resistance; H_2_S, hydrogen sulfide; IBD, inflammatory bowel disease; OMV, outer membrane vesicle; SCFA, short‐chain fatty acids.

## 3 Discussion

Next‐generation sequencing (NGS) has become a cornerstone technology in microbiome research due to its high sequencing depth, cost efficiency per sample, and high accuracy in short‐read analysis. However, its primary limitation lies in the short read length, which typically ranges from 100 to 300 base pairs for Illumina and up to 600 base pairs for Ion Torrent platforms. This constraint poses significant challenges in the de novo assembly of complex bacterial genomes and the precise differentiation of closely related microbial taxa. While NGS has been widely applied to study the microbiomes of both humans and animals, the short‐read nature of the technology often restricts 16S rRNA gene sequencing to partial coverage of only 3–4 variable regions at most. This limitation frequently results in difficulties distinguishing between closely related bacterial species, typically allowing only for accurate genus‐level identification rather than the species‐level resolution often required for comprehensive microbiome characterization.

Third‐generation sequencing (TGS) technologies, represented by PacBio Single Molecule Real‐Time (SMRT) sequencing and Oxford Nanopore Technology (ONT), address several fundamental limitations of NGS. The most significant advantage of TGS for microbiome analysis is its ability to generate long reads that can span the entire length of marker genes such as 16S rRNA (approximately 1500 base pairs) and 18S rRNA. This capability provides significantly improved taxonomic resolution compared to the partial gene coverage achieved with NGS technologies. While both NGS and TGS approaches for amplicon sequencing utilize PCR amplification, the long‐read capability of TGS enables complete coverage of taxonomically informative regions in a single read, eliminating the need for assembly of shorter fragments and reducing the ambiguity in taxonomic assignments. Additionally, the real‐time data acquisition capability, particularly in ONT‐based platforms, accelerates sequencing and downstream bioinformatics analyses, making it increasingly suitable for field applications and rapid microbiome assessments.

For microbiome analysis, the targeted sequencing of marker genes that enable species identification, particularly 16S and 18S rRNA genes, represents the most efficient approach. These conserved genes contain hypervariable regions that permit taxonomic classification while maintaining sufficient conservation for universal primer binding. Typically, only a small fraction of a bacterial genome, often estimated at less than 1%, consists of species‐specific sequences suitable for accurate taxonomic identification. This means that whole‐genome shotgun sequencing would require approximately 100 times more sequencing depth to achieve the same taxonomic resolution as targeted amplicon sequencing. In practical terms, this translates to 5 h of targeted sequencing versus several days for whole‐genome approaches, making amplicon sequencing significantly more cost‐effective and time‐efficient for taxonomic profiling.

While 16S and 18S rRNA genes remain the gold standard for bacterial and eukaryotic identification respectively, other marker genes are occasionally employed for specific taxonomic groups. These include *rpoB* (RNA polymerase beta subunit) for enhanced resolution in some bacterial genera (Ogier et al. 2019), *gyrB* (DNA gyrase subunit B) for better discrimination of closely related species (Liu, Pei, et al. 2021), and ITS (Internal Transcribed Spacer) regions for fungal identification. Despite widespread usage, 16S rRNA sequencing often lacks sufficient resolution for species and strain‐level identification (Johnson et al. 2019).

A significant limitation of amplicon‐based approaches is their dependence on universal primers. Primer bias can lead to selective amplification of certain microbial groups while failing to capture others, resulting in skewed community representations. This challenge is particularly pronounced when studying diverse and less characterized microbiomes such as those found in wildlife species like the brown hare. To overcome this limitation, in our study, we utilized proprietary primers developed by SPARK‐TECH that were specifically designed to target a broad range of over 120,000 prokaryotic and eukaryotic species. These primers are the subject of a patent application and represent a significant advancement in reducing amplification bias in microbiome studies of non‐model organisms. For 16S rRNA, the primers amplify a near‐full‐length region producing amplicons of approximately 1100 bp; for 18S rRNA, the target region yields amplicons of approximately 1700 bp. While the primer sequences themselves are proprietary, full reproducibility of the results presented here is ensured through the commercially available SPARKbiom sequencing and analysis service offered by SPARK‐TECH Sp. z o.o. (Kraków, Poland; https://spark‐tech‐lab.com). The service provides end‐to‐end long‐read 16S/18S rRNA amplicon sequencing using the same primer sets, library preparation protocols, and bioinformatic pipeline described in this study, enabling independent researchers to replicate or extend this work by submitting samples directly to the platform. This commercial availability satisfies the reproducibility requirement while protecting the underlying intellectual property, an approach analogous to other proprietary sequencing kits widely used in the field.

Our analysis of the brown hare microbiome reveals important considerations regarding sequence identity thresholds in taxonomic assignment. While human microbiome studies typically employ high similarity thresholds (≥ 95%) for taxonomic classification, our findings demonstrate that such stringent criteria may be suboptimal for wildlife microbiome analysis. At the 95% threshold, the number of sequences assigned was significantly lower compared to the 80% threshold. Our data showed that at the 80% threshold, the number of assigned sequences increased dramatically—by approximately 12.6‐fold for sample Z1, 10.2‐fold for sample Z2, and 12.7‐fold for sample Z3 compared to the 95% threshold. This difference is particularly important when considering how the BLAST algorithm functions in taxonomic assignment, where alignment scores and percentage identity are used to determine the closest match in reference databases.

The improved classification rate at the 80% threshold likely reflects the substantial diversity of microbial species in wildlife and the underrepresentation of these organisms in current reference databases. It is important to note that at the 80% threshold, the taxonomic label assigned to a given read represents the closest available reference sequence in the database, not a confirmed species identification. In the BLAST+ workflow, each query read is assigned to its single best‐matching reference; when this best hit shares only 80%–94.9% identity, the read most likely originates from an organism that is related to, but taxonomically distinct from, the named reference species. Such reads may represent novel strains, species variants adapted to the hare gut environment, or even entirely new taxa that have not been previously characterized. Therefore, species names reported at the 80% threshold should be interpreted as “nearest characterized relative” rather than definitive species assignments. This distinction is particularly relevant for wildlife microbiome studies, where reference database coverage is inherently limited, and underscores the potential of such studies as fertile ground for the discovery of novel microbial diversity. An important consideration regarding our analytical approach is the composition of the reference database. We deliberately excluded NCBI records annotated as “uncultured,” “unidentified,” or “unknown” (and additionally “environmental” and “sample” for 18S sequences) in order to obtain taxonomic assignments linked to formally described organisms. While this strategy ensures that each taxonomic label corresponds to a characterized reference, it may introduce a systematic bias in wildlife microbiome studies: for under‐studied host species such as the brown hare, the closest matching sequences in public databases may reside among uncultured or environmental entries that were excluded from our reference set. Consequently, some reads that failed to meet the percent identity threshold against our curated database might have achieved higher similarity scores against uncultured reference sequences, potentially altering the proportion of classified reads and the taxonomic labels assigned. This limitation should be considered when interpreting both the number of unassigned reads and the taxonomic identities reported, particularly at the 80% threshold. Future studies could benefit from parallel analyses using both curated and comprehensive (including uncultured) reference databases to quantify the impact of this filtering step.

Our results emphasize that achieving genus and species‐level resolution is critical for understanding microbiome composition and function in wildlife. The significant differences observed between 80% and 95% thresholds at these taxonomic levels (504‐606 unique genera and 1547‐1957 unique species identified only at the 80% threshold) demonstrate that overly stringent matching criteria may obscure substantial portions of the microbial community. This is particularly evident in the detection of important taxa such as Spirochaetota (25.2% abundance at 80% threshold but completely absent at 95%) and Ascomycota (4.3% abundance at 95% but absent at 80%), highlighting how threshold selection can fundamentally alter our perception of community composition.

Our data analysis did not identify any reads assigned to *Salmonella* spp. or *Listeria* spp. in the taxonomic alignments (see Tables S1 and S2). In contrast, *Escherichia coli* was detected incidentally at very low levels (e.g., Z1: approximately 0.04% of sequences; Table S1). This finding aligns with *E. coli* 's classification as a complex taxon that includes numerous non‐pathogenic commensal lineages; the material was obtained from healthy hares.

*Clostridium sensu lato* was present at a low proportion at the genus level (around 1.6% at the 80% threshold), represented by species that are not typical mammalian pathogens, such as *Clostridium islandicum*. Notably, no classic enteric pathogens were found among the dominant taxa (Tables S1 and S2).

Additionally, in the 95% alignment, we observed low but measurable shares of zoonotic opportunistic pathogens, including *Staphylococcus pseudintermedius* (approximately 1.5%) and *Streptococcus canis* (around 2.5%). These findings may suggest environmental exposure to the microbiota of farm and companion animals or cross‐species transmission; however, these results should be interpreted with caution.

The comprehensive nature of our approach is further validated by our comparative analysis with previous studies of the hare microbiome. Our findings encompass nearly all previously reported taxa while identifying thousands of additional organisms not detected in earlier investigations. Specifically, we identified 40 phyla (with 28 unique to our study), 360 unique families, 1027 unique genera, and 3373 unique species not reported in previous publications. This substantial expansion of the known hare microbiome composition directly results from the combination of third‐generation sequencing technology and optimized taxonomic assignment parameters.

The advantages of TGS make it particularly valuable for studying the microbiome of wildlife species like the brown hare. The ability to fully sequence 16S and 18S rRNA genes provides unprecedented taxonomic resolution, enabling the identification of rare or novel microbial strains that might play crucial roles in host health or disease. This comprehensive approach to taxonomic classification significantly enhances our understanding of microbial diversity in wildlife species.

The choice of sequence identity threshold should not be treated as a purely technical decision made during data processing, but rather as a fundamental research design consideration with substantial consequences for biological interpretation. While a more permissive threshold is tempting due to its capacity to uncover a far greater number of taxa—potentially revealing novel or poorly characterized lineages—its application requires careful a priori justification. When the research objective is hypothesis‐driven and focused on well‐characterized microbial communities, a stringent threshold offers greater taxonomic confidence and reduces the risk of misassignment. In contrast, when the goal is exploratory—particularly in wildlife species with underrepresented gut microbiomes in reference databases—a relaxed threshold may serve as a legitimate post hoc discovery tool, provided that results are interpreted as tentative taxonomic annotations rather than definitive species identifications. Ideally, the threshold strategy should be defined before data collection, aligned with the biological question, the expected diversity of the host's microbiome, and the coverage of available reference databases. Parallel analysis at multiple thresholds, as applied in the present study, can serve as a useful framework for distinguishing a conserved core microbiome from a broader, putatively novel microbial landscape. A limitation of the present study is that pooling prevents conclusions on inter‐individual differences. However, pooling can also be considered an advantage, as it emphasizes the population‐level microbial signature of the European brown hare. This approach reduces noise introduced by transient or host‐specific microorganisms and allows detection of taxa representative of the species' gut ecosystem. Such a perspective is particularly valuable in wildlife research, where ecological and epidemiological roles (e.g., as reservoirs of pathogens or indicators of environmental microbial diversity) are more relevant than the characterization of single individuals. Future studies should combine both strategies—pooling for population‐level inference and individual sampling for assessing host‐specific variability.

In summary, our study represents the most comprehensive characterization of the brown hare microbiome to date, made possible by the application of third‐generation sequencing technology and optimized analytical approaches. Our findings not only encompass previously identified taxa but also substantially expand our understanding of the microbial diversity associated with this important wildlife species. Furthermore, our methodological approach provides a valuable framework for future wildlife microbiome studies, emphasizing the importance of appropriate sequence identity thresholds and comprehensive taxonomic analysis based on full‐length 16S and 18S rRNA gene sequencing.

## 4 Materials and Methods

In Poland, according to the Regulation of the Minister of the Environment regarding hunting seasons for game animals, the hunting season for brown hares is from 1 November to 31 December (Regulation of the Minister of the Environment of 16 2005).

### 4.1 Animals, Samples, and Localisation

The hares chosen for the study were selected during a 2‐day collective hunt organized in two hunting districts in the western part of the Lublin Upland. During the 2024 hare hunting season, a total of 30 healthy brown hares ( *Lepus europaeus* Pall.) were hunted, including both males and females. After conducting autopsies, it was determined that all the carcasses were healthy and showed no anatomopathological changes.

The study material consisted of large intestine contents. A total of 30 hares were randomly divided into three groups (Z1, Z2, Z3), each comprising 10 individuals. Intestinal contents from 30 European brown hares were collected and subsequently combined into three pooled samples (Z1–Z3). Pooling was deliberately applied to obtain sufficient DNA yields for high‐throughput long‐read sequencing and to reduce stochastic variation associated with low‐biomass samples. Moreover, by merging individuals, each pool reflected the microbiota composition at the population level rather than being biased by host‐specific variation. This strategy has been successfully applied in previous wildlife microbiome studies where the objective was to characterize community‐wide microbial diversity rather than individual profiles. From each composite sample (Z1, Z2, and Z3), a 2.5 g sample was collected and stored at −20°C for further analysis.

### 4.2 Sequencing, Data Calculation, and Statistical Analysis

The protocol for isolation, amplification, and sequencing was performed as previously published with modifications (Węsierska et al. 2025).

Prior to sequencing, the samples were thawed at a controlled temperature. DNA was then isolated using a silica column‐based kit (QIAamp PowerFecal Pro DNA Kit, QIAGEN), according to the manufacturer's instructions. Following DNA isolation, polymerase chain reaction (PCR) was performed to amplify the 16S rRNA gene and 18S rRNA gene regions of the bacterial and eukaryotic DNA. This amplification was carried out using proprietary SPARKbiom primers (SPARK‐TECH). After PCR amplification, the samples were prepared for sequencing according to the Oxford Nanopore Kit 14 chemistry library preparation protocol. Amplification was performed under the following conditions: the first step was initial denaturation at 95°C for 1 min; the second step included 30 cycles (denaturation at 95°C for 20s, annealing at 55°C for 30s, and extension at 65°C for 2 min). The last step was a final extension at 65°C for 5 min. Nanopore sequencing was then carried out using the MinION device (Oxford Nanopore). Upon completion of the sequencing, the raw data was processed using the MinKNOW software (Oxford Nanopore Technologies) for real‐time base calling and quality assessment. A minimum quality score threshold of Q15 was applied during base calling; reads below this threshold were discarded. Of the reads passing the Q15 filter, approximately 20%–30% remained unclassified by MinKNOW's internal barcoding algorithm and were excluded from downstream analysis. The remaining classified reads were further filtered by alignment length during the BLAST+ step, with matches shorter than 500 nucleotides excluded from taxonomic assignment. No additional length trimming, subsampling, or chimera‐filtering steps were applied, as the use of full‐length amplicon primers and the Kit 14 chemistry (which provides per‐read accuracy of > Q20) were considered sufficient to minimize chimeric and truncated sequences.

Taxonomic classification of the sequences was carried out using a database of 1.5 million reference sequences from NCBI (querry 16:16S [Title] NOT uncultured [All Fields] NOT unidentified [All Fields] NOT unknown[All Fields] AND (800:40,000 [Sequence Length]), querry18: 18S[Title] NOT (uncultured [All Fields] OR unidentified [All Fields] OR unknown[All Fields] OR environmental [All Fields] OR sample[All Fields]) AND (500:5000 [Sequence Length])). BLAST+ (version ncbi‐blast‐2.16.0) was employed for taxonomic assignment. All quality‐filtered reads (Q score ≥ 15; length ≥ 500 nt) were aligned against the reference database in a single BLAST run. From this single set of alignments, two taxonomic profiles were derived by applying minimum percent identity thresholds of 95% and 80%, respectively. Consequently, the 80% dataset represents a superset that includes all reads classified at 95%, plus additional reads whose best BLAST hit falls between 80% and 94.9% identity. This means that the higher number of assigned sequences at the 80% threshold (e.g., 33,573 vs. 2471 for sample Z1 at the superkingdom level) does not stem from a different input read set, but from the inclusion of reads whose closest database match falls below 95% identity. Such reads likely represent taxa that are divergent from available reference sequences. Matches with alignment lengths below 500 nucleotides were excluded from both analyses. For each sample, two taxonomic profiles were obtained at the 95% and 80% thresholds. The pooled sample contains taxa from all samples with a summed count for each species, with the average based on the total count.

Based on two publications, Stalder et al. (2019) and Padula et al. (2021), taxonomic groups present in the hare intestine were identified at the phylum, family, genus, and species levels. Taxonomic classification was mapped according to the current NCBI taxonomy. In instances where specific taxa could not be assigned, they were designated as “unclassified,” followed by the original taxonomic designation reported in the respective publication.

For the paired sample analysis, a systematic comparison of taxonomic results was conducted at sequence identity thresholds of 95% and 80% using multiple diversity indices. Biodiversity metrics were calculated for each sample pair, including the Shannon diversity index (H′), Species Richness, Simpson diversity index (1‐D), Pielou's evenness index (J), and the Chao1 species richness estimator.

For the comparative analysis of taxonomic diversity, the number of unique taxa and the Shannon diversity index were calculated for each taxonomic level, ranging from Superkingdom to Species, across all samples at both sequence identity thresholds. Data processing and analysis were conducted using Python, employing the pandas and NumPy libraries. Visualization was performed using Plotly to generate comparative bar charts illustrating taxonomic richness and Shannon diversity indices, facilitating a direct comparison between datasets at the 80% and 95% sequence identity thresholds.

Following the analysis of taxonomic diversity, Venn diagram visualization was applied to examine relationships between samples across sequence identity thresholds. Using the matplotlib‐venn library in Python, interactive side‐by‐side diagrams were generated for each taxonomic level, from Phylum to Species, to illustrate shared and unique taxa among samples (Z1, Z2, Z3) at both 80% and 95% sequence identity thresholds. This analysis was supplemented with a comprehensive summary table presenting the total number of unique taxa, taxa common to all samples, and taxon counts specific to individual samples at each taxonomic level.

For the assessment of taxonomic composition, relative abundance was aggregated and visualized using Python with the pandas and matplotlib libraries. Stacked bar charts were generated to display the top 15 taxa at each taxonomic level, while less abundant taxa (< 1%) were grouped into an “Other” category. NCBI Taxonomy IDs were incorporated to facilitate parallel comparisons between sequence identity thresholds.

To assess differences between classifications at the two sequence identity thresholds, a comparative group analysis was conducted by calculating three key metrics: the average number of taxa identified per sample at each threshold, the number of taxa unique to each threshold, and the number of taxa shared between both thresholds. Visualizations included grouped bar charts representing average taxa counts and stacked charts illustrating the composition breakdown across taxonomic levels.

Statistical evaluation was conducted using a non‐parametric approach, applying Kruskal‐Wallis H‐tests to assess significant differences in taxonomic composition between samples at both sequence identity thresholds. Statistical significance was defined as *p* < 0.05. The results were compiled into tables presenting H‐statistic values, *p*‐values, and significance determinations for each comparison.

For comparative analysis with published literature, Venn diagram visualization was utilized to compare the 80% threshold dataset with taxonomic compositions reported in two reference publications (Stalder et al. 2019; Padula et al. 2021). The analysis focused on four taxonomic levels (Phylum, Family, Genus, Species) and incorporated both NCBI‐standardized nomenclature and the original taxonomic names from the referenced studies. Diagrams illustrating taxonomic overlap were generated, and reports were compiled to document taxa identified in previous studies but absent from the analyzed dataset.

### 4.3 Statistical Analysis

All analyses were performed on pooled sample profiles (Z1–Z3) generated from the same set of quality‐filtered reads. During pre‐processing, reads with a base‐calling quality score below Q15 were discarded, unclassified reads were excluded, and only BLAST alignments of at least 500 nt were retained. No additional normalization or rarefaction was applied. Taxonomic composition was summarized as counts and relative abundances, and alpha diversity was reported using Shannon diversity (H), Simpson diversity (1–D), Pielou's evenness (J), species richness, and Chao1. For each comparison, *n* = 3 pooled samples per threshold (Z1, Z2, Z3). Differences in taxonomic composition among pooled samples were evaluated separately for each taxonomic level using the Kruskal–Wallis H‐test, with a significance threshold of *p* < 0.05. Statistical analyses were conducted in Python using pandas, NumPy, and SciPy, and figures were generated using matplotlib, Plotly, and matplotlib‐venn.

## Author Contributions

**Zbigniew Bełkot:** conceptualization, investigation, methodology, data curation, resources, writing – original draft, writing – review and editing, funding acquisition. **Daria Kłosińska:** investigation, writing – original draft, writing – review and editing, methodology. **Ewa D. Domańska:** investigation, writing – original draft, writing – review and editing, resources, data curation. **Zuzanna J. Strzałkowska:** investigation, writing – original draft, writing – review and editing, methodology, resources, data curation. **Mateusz G. Adamski:** conceptualization, methodology, software, writing – original draft, writing – review and editing, visualization, formal analysis, validation. **Grzegorz Kunstman:** software, formal analysis, writing – review and editing, writing – original draft. **Joanna Pławińska‐Czarnak:** conceptualization, investigation, writing – original draft, writing – review and editing, methodology, visualization, formal analysis, supervision, project administration, resources. **Dawid Skoczek:** writing – original draft, writing – review and editing, software, formal analysis.

## Funding

The authors have nothing to report.

## Ethics Statement

The study complied with Directive 2010/63/EU and the Act of the Polish Parliament dated 15 January 2015 on the protection of animals used for scientific purposes (Journal of Laws of the Republic of Poland 2015, item 266). The brown hares were not killed for the purposes of the study. The hunt took place in accordance with Polish hunting law (Act of the Polish Parliament dated 13 October 1995, item 713, the Hunting law, Chapter 3, Art. 8 Hunt and the Regulation of the Minister of the Environment of 16 March 2005 on the determination of hunting periods for game animals [Journal of Laws 2023, item 99]) during the 2024 hunting season.

## Consent

The authors have nothing to report.

## Conflicts of Interest

The authors declare no conflicts of interest.

## Supporting information


**Table S1:** Detailed taxonomic composition, sequence identifiers, and abundance metrics for microbial communities across brown hare intestinal samples (Z1, Z2, and Z3). The dataset includes absolute sequence counts and relative abundances for each identified taxon, filtered to include only those with a minimum sequence count threshold of 10 (Count ≥ 10).

**Table S2:** Quantitative group comparison analysis of detected microbial taxa across hierarchical classification levels at 80% and 95% sequence identity thresholds. This dataset represents the underlying values for Figure 3, detailing the average number of taxa identified per sample, taxa unique to each stringency threshold, and the core set of shared taxa.

**Table S3:** Comprehensive inventory of microbial taxa reported in previous brown hare (Lepus europaeus) gut microbiome reference studies (Stalder et al. 2019 and Padula et al. 2021) that were absent from the current dataset. Lineages are mapped according to current NCBI taxonomy standards, highlighting both valid entries and those lacking defined database nomenclature.

## Data Availability

The data that support the findings of this study are openly available in [NCBI] at https://www.ncbi.nlm.nih.gov/sra/PRJNA1238910, reference number PRJNA1238910SUB15187482.
