# Supplementary material for: Full‐Length 16S and 18S rRNA Long‐Read Sequencing Reveals Gut Microbiome Diversity in the European Brown Hare ( Lepus europaeus )
Source: Environ Microbiol Rep. 2026 May 25;18(3):e70358. doi: 10.1111/1758-2229.70358 (PMC13239162; doi:10.1111/1758-2229.70358)
Supplement: Supplementary file 1 — Table S1: Detailed taxonomic composition, sequence identifiers, and abundance metrics for microbial communities across brown hare intestinal samples (Z1, Z2, and Z3). The dataset includes absolute sequence counts and relative abundances for each identified taxon, filtered to include only those with a minimum sequence count threshold of 10 (Count ≥ 10). [file EMI4-18-e70358-s001.docx]

**Supplementary Table 1**

Taxonomic composition of microbial communities in samples Z1, Z2, and Z3. The table includes unique sequence identifiers, taxonomic classification, and abundance metrics for each identified taxon. Only taxa with at least 10 sequences (Count ≥ 10) are included in this table.

Column descriptions:

- Sample: Sample identifier (Z1, Z2, or Z3)
- ID: Unique identifier for each sequence within the dataset
- Count: Number of reads assigned to the taxon (minimum 10)
- %: Percentage of reads within the sample
- NCBI Tax ID: NCBI Taxonomy database identifier
- Sequence ID: Unique sequence identifier
- Tax Name: Taxonomic name as assigned in the NCBI database
- Superkingdom: Highest taxonomic rank (Domain)
- Phylum: Phylum classification
- Class: Class classification
- Order: Order classification
- Family: Family classification
- Genus: Genus classification
- Species: Species classification

| **Sample** | **ID** | **Count** | **%** | **NCBI Tax ID** | **Sequence ID** | **Tax Name** | **Superkingdom** | **Phylum** | **Class** | **Order** | **Family** | **Genus** | **Species** |
| --- | --- | --- | --- | --- | --- | --- | --- | --- | --- | --- | --- | --- | --- |
| Z1 | 1 | 14384 | 24.06 | 1151583 | NCBI-3-16S@JN713550.1 | Spirochaeta sp. canine oral taxon 379 | Bacteria | Spirochaetota | Spirochaetia | Spirochaetales | Spirochaetaceae | Spirochaeta | Spirochaeta sp. canine oral taxon 379 |
| Z1 | 2 | 2445 | 4.09 | 1879010 | NCBI-3-16S@MW741711.1 | Bacillota bacterium | Bacteria | Bacillota |  |  |  |  | Bacillota bacterium |
| Z1 | 3 | 1877 | 3.14 | 454154 | NCBI-3-16S@OK510284.1 | Paraprevotella clara | Bacteria | Bacteroidota | Bacteroidia | Bacteroidales | Prevotellaceae | Paraprevotella | Paraprevotella clara |
| Z1 | 4 | 1545 | 2.58 | 1965233 | NCBI-3-16S@MK287741.1 | Odoribacter sp. | Bacteria | Bacteroidota | Bacteroidia | Bacteroidales | Odoribacteraceae | Odoribacter | Odoribacter sp. |
| Z1 | 5 | 1479 | 2.47 | -1 | NOT_FOUND | --- |  |  |  |  |  |  |  |
| Z1 | 6 | 1124 | 1.88 | 40519 | NCBI-3-16S@X85100.1 | Ruminococcus callidus | Bacteria | Bacillota | Clostridia | Eubacteriales | Oscillospiraceae | Ruminococcus | Ruminococcus callidus |
| Z1 | 7 | 1003 | 1.68 | 214819 | NCBI-3-16S@AF550610.1 | Lachnospiraceae bacterium 19gly4 | Bacteria | Bacillota | Clostridia | Lachnospirales | Lachnospiraceae |  | Lachnospiraceae bacterium 19gly4 |
| Z1 | 8 | 968 | 1.62 | 213810 | NCBI-3-16S@NR_102884.1 | Ruminococcus champanellensis 18P13 = JCM 17042 | Bacteria | Bacillota | Clostridia | Eubacteriales | Oscillospiraceae | Ruminococcus | Ruminococcus champanellensis |
| Z1 | 9 | 897 | 1.50 | 862466 | NCBI-3-16S@HM231145.1 | Clostridiales bacterium KM2 | Bacteria | Bacillota | Clostridia | Eubacteriales |  |  | Clostridiales bacterium KM2 |
| Z1 | 10 | 720 | 1.20 | 820 | NCBI-3-16S@OR946131.1 | Bacteroides uniformis | Bacteria | Bacteroidota | Bacteroidia | Bacteroidales | Bacteroidaceae | Bacteroides | Bacteroides uniformis |
| Z1 | 11 | 647 | 1.08 | 41978 | NCBI-3-16S@MK431733.1 | Ruminococcus sp. | Bacteria | Bacillota | Clostridia | Eubacteriales | Oscillospiraceae | Ruminococcus | Ruminococcus sp. |
| Z1 | 12 | 638 | 1.07 | 2606638 | NCBI-3-16S@NR_180814.1 | Bullifex porci | Bacteria | Spirochaetota | Spirochaetia | Spirochaetales | Spirochaetaceae | Bullifex | Bullifex porci |
| Z1 | 13 | 637 | 1.07 | 357276 | NCBI-3-16S@OR125614.1 | Phocaeicola dorei | Bacteria | Bacteroidota | Bacteroidia | Bacteroidales | Bacteroidaceae | Phocaeicola | Phocaeicola dorei |
| Z1 | 14 | 465 | 0.78 | 393755 | NCBI-3-16S@DQ676999.1 | iron-reducing enrichment clone Cl-A7 | Bacteria |  |  |  |  |  | iron-reducing enrichment clone Cl-A7 |
| Z1 | 15 | 439 | 0.73 | 1917882 | NCBI-3-16S@NR_179582.1 | Gabonibacter timonensis | Bacteria | Bacteroidota | Bacteroidia | Bacteroidales | Porphyromonadaceae | Gabonibacter | Gabonibacter timonensis |
| Z1 | 16 | 405 | 0.68 | 1945594 | NCBI-3-16S@MW682303.1 | Sporobacter sp. | Bacteria | Bacillota | Clostridia | Eubacteriales | Oscillospiraceae | Sporobacter | Sporobacter sp. |
| Z1 | 17 | 404 | 0.68 | 666483 | NCBI-3-16S@GQ377128.1 | bacterium enrichment culture clone DPF25 | Bacteria |  |  |  |  |  | bacterium enrichment culture clone DPF25 |
| Z1 | 18 | 395 | 0.66 | 2779355 | NCBI-3-16S@NR_180617.1 | Ructibacterium gallinarum | Bacteria | Bacillota | Clostridia | Eubacteriales | Oscillospiraceae | Ructibacterium | Ructibacterium gallinarum |
| Z1 | 19 | 375 | 0.63 | 1792311 | NCBI-3-16S@NR_178871.1 | Petroclostridium xylanilyticum | Bacteria | Bacillota | Clostridia | Eubacteriales | Oscillospiraceae | Petroclostridium | Petroclostridium xylanilyticum |
| Z1 | 20 | 373 | 0.62 | 1131707 | NCBI-3-16S@NR_102964.1 | Sphaerochaeta pleomorpha | Bacteria | Spirochaetota | Spirochaetia | Spirochaetales | Sphaerochaetaceae | Sphaerochaeta | Sphaerochaeta pleomorpha |
| Z1 | 21 | 371 | 0.62 | 1980281 | NCBI-3-16S@MN081672.1 | Pseudoflavonifractor sp. | Bacteria | Bacillota | Clostridia | Eubacteriales | Oscillospiraceae | Pseudoflavonifractor | Pseudoflavonifractor sp. |
| Z1 | 22 | 342 | 0.57 | 1981510 | NCBI-3-16S@MK615117.1 | Monoglobus pectinilyticus | Bacteria | Bacillota | Clostridia | Monoglobales | Monoglobaceae | Monoglobus | Monoglobus pectinilyticus |
| Z1 | 23 | 324 | 0.54 | 28118 | NCBI-3-16S@NR_113075.1 | Odoribacter splanchnicus | Bacteria | Bacteroidota | Bacteroidia | Bacteroidales | Odoribacteraceae | Odoribacter | Odoribacter splanchnicus |
| Z1 | 24 | 319 | 0.53 | 821 | NCBI-3-16S@MT902980.1 | Phocaeicola vulgatus | Bacteria | Bacteroidota | Bacteroidia | Bacteroidales | Bacteroidaceae | Phocaeicola | Phocaeicola vulgatus |
| Z1 | 25 | 315 | 0.53 | 544645 | NCBI-3-16S@MT902988.1 | Butyricimonas virosa | Bacteria | Bacteroidota | Bacteroidia | Bacteroidales | Odoribacteraceae | Butyricimonas | Butyricimonas virosa |
| Z1 | 26 | 313 | 0.52 | 1920505 | NCBI-3-16S@OQ146993.1 | Pleomorphochaeta sp. | Bacteria | Spirochaetota | Spirochaetia | Spirochaetales | Sphaerochaetaceae | Pleomorphochaeta | Pleomorphochaeta sp. |
| Z1 | 27 | 313 | 0.52 | 519019 | NCBI-3-16S@EU592966.1 | Atopobium sp. F0209 | Bacteria | Actinomycetota | Coriobacteriia | Coriobacteriales | Atopobiaceae | Atopobium | Atopobium sp. F0209 |
| Z1 | 28 | 309 | 0.52 | 28117 | NCBI-3-16S@NR_113152.1 | Alistipes putredinis | Bacteria | Bacteroidota | Bacteroidia | Bacteroidales | Rikenellaceae | Alistipes | Alistipes putredinis |
| Z1 | 29 | 300 | 0.50 | 2026735 | NCBI-3-16S@OQ808222.1 | Deltaproteobacteria bacterium | Bacteria | Myxococcota | Myxococcia |  |  |  | Deltaproteobacteria bacterium |
| Z1 | 30 | 300 | 0.50 | 2364796 | NCBI-3-16S@LS999998.1 | Ruminococcus sp. Marseille-P6503 | Bacteria | Bacillota | Clostridia | Eubacteriales | Oscillospiraceae | Ruminococcus | Ruminococcus sp. Marseille-P6503 |
| Z1 | 31 | 277 | 0.46 | 1796646 | NCBI-3-16S@OK626632.1 | Muribaculum intestinale | Bacteria | Bacteroidota | Bacteroidia | Bacteroidales | Muribaculaceae | Muribaculum | Muribaculum intestinale |
| Z1 | 32 | 267 | 0.45 | 329854 | NCBI-3-16S@NR_041307.1 | Bacteroides intestinalis | Bacteria | Bacteroidota | Bacteroidia | Bacteroidales | Bacteroidaceae | Bacteroides | Bacteroides intestinalis |
| Z1 | 33 | 262 | 0.44 | 2763676 | NCBI-3-16S@MT905150.1 | Qingrenia yutianensis | Bacteria | Bacillota | Clostridia | Eubacteriales | Oscillospiraceae | Qingrenia | Qingrenia yutianensis |
| Z1 | 34 | 261 | 0.44 | 502558 | NCBI-3-16S@AB379693.1 | Eggerthella sp. YY7918 | Bacteria | Actinomycetota | Coriobacteriia | Eggerthellales | Eggerthellaceae | Eggerthella | Eggerthella sp. YY7918 |
| Z1 | 35 | 254 | 0.42 | 1796610 | NCBI-3-16S@OM658549.1 | Adlercreutzia muris | Bacteria | Actinomycetota | Coriobacteriia | Eggerthellales | Eggerthellaceae | Adlercreutzia | Adlercreutzia muris |
| Z1 | 36 | 252 | 0.42 | 2725562 | NCBI-3-16S@NR_181379.1 | Caecibacteroides pullorum | Bacteria | Bacteroidota | Bacteroidia | Bacteroidales | Bacteroidaceae | Caecibacteroides | Caecibacteroides pullorum |
| Z1 | 37 | 246 | 0.41 | 55507 | NCBI-3-16S@Y09434.1 | Schwartzia succinivorans | Bacteria | Bacillota | Negativicutes | Selenomonadales | Selenomonadaceae | Schwartzia | Schwartzia succinivorans |
| Z1 | 38 | 244 | 0.41 | 411317 | NCBI-3-16S@EF088328.1 | Clostridium islandicum | Bacteria | Bacillota | Clostridia | Eubacteriales | Clostridiaceae | Clostridium | Clostridium islandicum |
| Z1 | 39 | 241 | 0.40 | 697329 | NCBI-3-16S@NR_115230.1 | Ruminococcus albus 7 = DSM 20455 | Bacteria | Bacillota | Clostridia | Eubacteriales | Oscillospiraceae | Ruminococcus | Ruminococcus albus |
| Z1 | 40 | 236 | 0.39 | 2485925 | NCBI-3-16S@MN913813.1 | Oscillospiraceae bacterium | Bacteria | Bacillota | Clostridia | Eubacteriales | Oscillospiraceae |  | Oscillospiraceae bacterium |
| Z1 | 41 | 226 | 0.38 | 1264 | NCBI-3-16S@X85098.1 | Ruminococcus albus | Bacteria | Bacillota | Clostridia | Eubacteriales | Oscillospiraceae | Ruminococcus | Ruminococcus albus |
| Z1 | 42 | 211 | 0.35 | 1330740 | NCBI-3-16S@KC853480.1 | bacterium enrichment culture clone aHCH2_E12 | Bacteria |  |  |  |  |  | bacterium enrichment culture clone aHCH2_E12 |
| Z1 | 43 | 205 | 0.34 | 2606638 | NCBI-3-16S@NR_180814.1 | Bullifex porci | Bacteria | Spirochaetota | Spirochaetia | Spirochaetales | Spirochaetaceae | Bullifex | Bullifex porci |
| Z1 | 44 | 192 | 0.32 | 394503 | NCBI-3-16S@OK626620.1 | Ruminiclostridium cellulolyticum H10 | Bacteria | Bacillota | Clostridia | Eubacteriales | Oscillospiraceae | Ruminiclostridium | Ruminiclostridium cellulolyticum |
| Z1 | 45 | 192 | 0.32 | 1265 | NCBI-3-16S@AF030449.1 | Ruminococcus flavefaciens | Bacteria | Bacillota | Clostridia | Eubacteriales | Oscillospiraceae | Ruminococcus | Ruminococcus flavefaciens |
| Z1 | 46 | 190 | 0.32 | 2872177 | NCBI-3-16S@OM658611.1 | Odoribacteraceae bacterium | Bacteria | Bacteroidota | Bacteroidia | Bacteroidales | Odoribacteraceae |  | Odoribacteraceae bacterium |
| Z1 | 47 | 190 | 0.32 | 29523 | NCBI-3-16S@AM117579.1 | Bacteroides sp. | Bacteria | Bacteroidota | Bacteroidia | Bacteroidales | Bacteroidaceae | Bacteroides | Bacteroides sp. |
| Z1 | 48 | 185 | 0.31 | 2986072 | NCBI-3-16S@OX352005.1 | Candidatus Minimicrobia sp. IHU4 | Bacteria | Candidatus Saccharibacteria |  |  |  | Candidatus Minimicrobia | Candidatus Minimicrobia sp. IHU4 |
| Z1 | 49 | 185 | 0.31 | 1965293 | NCBI-3-16S@MN081721.1 | Intestinimonas sp. | Bacteria | Bacillota | Clostridia | Eubacteriales |  | Intestinimonas | Intestinimonas sp. |
| Z1 | 50 | 183 | 0.31 | 84026 | NCBI-3-16S@MT903092.1 | [Clostridium] methylpentosum | Bacteria | Bacillota | Clostridia | Eubacteriales | Oscillospiraceae |  | [Clostridium] methylpentosum |
| Z1 | 51 | 182 | 0.30 | 1309939 | NCBI-3-16S@KC441622.1 | bacterium enrichment culture clone ZZ_F11b | Bacteria |  |  |  |  |  | bacterium enrichment culture clone ZZ_F11b |
| Z1 | 52 | 181 | 0.30 | 1172158 | NCBI-3-16S@JQ735967.1 | bacterium enrichment culture clone AK10 | Bacteria |  |  |  |  |  | bacterium enrichment culture clone AK10 |
| Z1 | 53 | 178 | 0.30 | 1411144 | NCBI-3-16S@AB861982.1 | Bacteroides caecigallinarum | Bacteria | Bacteroidota | Bacteroidia | Bacteroidales | Bacteroidaceae | Bacteroides | Bacteroides caecigallinarum |
| Z1 | 54 | 171 | 0.29 | 1188760 | NCBI-3-16S@JQ670725.1 | bacterium enrichment culture clone KWE55-24 | Bacteria |  |  |  |  |  | bacterium enrichment culture clone KWE55-24 |
| Z1 | 55 | 170 | 0.28 | 258132 | NCBI-3-16S@AY466715.1 | Clostridiales bacterium NS5-2 | Bacteria | Bacillota | Clostridia | Eubacteriales |  |  | Clostridiales bacterium NS5-2 |
| Z1 | 56 | 169 | 0.28 | 2164149 | NCBI-3-16S@NR_169458.1 | Tepidibaculum saccharolyticum | Bacteria | Bacillota | Clostridia | Eubacteriales | Oscillospiraceae | Tepidibaculum | Tepidibaculum saccharolyticum |
| Z1 | 57 | 166 | 0.28 | 1131707 | NCBI-3-16S@NR_114609.1 | Sphaerochaeta pleomorpha | Bacteria | Spirochaetota | Spirochaetia | Spirochaetales | Sphaerochaetaceae | Sphaerochaeta | Sphaerochaeta pleomorpha |
| Z1 | 58 | 154 | 0.26 | 1852363 | NCBI-3-16S@LT576387.1 | Clostridiales bacterium Marseille-P2846 | Bacteria | Bacillota | Clostridia | Eubacteriales | Beduinellaceae | Beduinella | Beduinella massiliensis |
| Z1 | 59 | 152 | 0.25 | 1872387 | NCBI-3-16S@MK287689.1 | Adlercreutzia sp. | Bacteria | Actinomycetota | Coriobacteriia | Eggerthellales | Eggerthellaceae | Adlercreutzia | Adlercreutzia sp. |
| Z1 | 60 | 149 | 0.25 | 823 | NCBI-3-16S@AB640686.1 | Parabacteroides distasonis | Bacteria | Bacteroidota | Bacteroidia | Bacteroidales | Tannerellaceae | Parabacteroides | Parabacteroides distasonis |
| Z1 | 61 | 147 | 0.25 | 1870988 | NCBI-3-16S@NR_147370.1 | Pseudoflavonifractor phocaeensis | Bacteria | Bacillota | Clostridia | Eubacteriales | Oscillospiraceae | Pseudoflavonifractor | Pseudoflavonifractor phocaeensis |
| Z1 | 62 | 140 | 0.23 | 2751153 | NCBI-3-16S@NR_173687.1 | Coprobacter secundus subsp. similis | Bacteria | Bacteroidota | Bacteroidia | Bacteroidales | Barnesiellaceae | Coprobacter | Coprobacter secundus |
| Z1 | 63 | 140 | 0.23 | 1702285 | NCBI-3-16S@NR_179426.1 | Intestinimonas gabonensis | Bacteria | Bacillota | Clostridia | Eubacteriales |  | Intestinimonas | Intestinimonas gabonensis |
| Z1 | 64 | 139 | 0.23 | 1161942 | NCBI-3-16S@MH282446.1 | Ruminococcus champanellensis | Bacteria | Bacillota | Clostridia | Eubacteriales | Oscillospiraceae | Ruminococcus | Ruminococcus champanellensis |
| Z1 | 65 | 135 | 0.23 | 1776381 | NCBI-3-16S@NR_146815.1 | Olegusella massiliensis | Bacteria | Actinomycetota | Coriobacteriia | Coriobacteriales | Coriobacteriaceae | Olegusella | Olegusella massiliensis |
| Z1 | 66 | 132 | 0.22 | 399361 | NCBI-3-16S@DQ833401.1 | Sphaerochaeta sp. RCcp2 | Bacteria | Spirochaetota | Spirochaetia | Spirochaetales | Sphaerochaetaceae | Sphaerochaeta | Sphaerochaeta sp. RCcp2 |
| Z1 | 67 | 124 | 0.21 | 1972561 | NCBI-3-16S@OM368626.1 | Eggerthellaceae bacterium | Bacteria | Actinomycetota | Coriobacteriia | Eggerthellales | Eggerthellaceae |  | Eggerthellaceae bacterium |
| Z1 | 68 | 124 | 0.21 | 2939460 | NCBI-3-16S@OP389241.1 | Parvivirga hydrogeniphila | Bacteria | Actinomycetota | Coriobacteriia | Anaerosomatales | Anaerosomataceae | Parvivirga | Parvivirga hydrogeniphila |
| Z1 | 69 | 123 | 0.21 | 1650661 | NCBI-3-16S@NR_179410.1 | Clostridium phoceensis | Bacteria | Bacillota | Clostridia | Eubacteriales | Clostridiaceae | Clostridium | Clostridium phoceensis |
| Z1 | 70 | 118 | 0.20 | 1872444 | NCBI-3-16S@OK271574.1 | Alistipes sp. | Bacteria | Bacteroidota | Bacteroidia | Bacteroidales | Rikenellaceae | Alistipes | Alistipes sp. |
| Z1 | 71 | 116 | 0.19 | 1945593 | NCBI-3-16S@MK287649.1 | Oscillibacter sp. | Bacteria | Bacillota | Clostridia | Eubacteriales | Oscillospiraceae | Oscillibacter | Oscillibacter sp. |
| Z1 | 72 | 116 | 0.19 | 1870986 | NCBI-3-16S@NR_147375.1 | Colidextribacter massiliensis | Bacteria | Bacillota | Clostridia | Eubacteriales |  | Colidextribacter | Colidextribacter massiliensis |
| Z1 | 73 | 114 | 0.19 | 1917883 | NCBI-3-16S@NR_179583.1 | Bacteroides togonis | Bacteria | Bacteroidota | Bacteroidia | Bacteroidales | Bacteroidaceae | Bacteroides | Bacteroides togonis |
| Z1 | 74 | 113 | 0.19 | 1870985 | NCBI-3-16S@NR_179544.1 | Arabiibacter massiliensis | Bacteria | Actinomycetota | Coriobacteriia | Eggerthellales | Eggerthellaceae | Arabiibacter | Arabiibacter massiliensis |
| Z1 | 75 | 112 | 0.19 | 747645 | NCBI-3-16S@NR_117374.1 | Parvibacter caecicola | Bacteria | Actinomycetota | Coriobacteriia | Coriobacteriales | Coriobacteriaceae | Parvibacter | Parvibacter caecicola |
| Z1 | 76 | 111 | 0.19 | 1151617 | NCBI-3-16S@JN713189.1 | Clostridiales bacterium canine oral taxon 027 | Bacteria | Bacillota | Clostridia | Eubacteriales |  |  | Clostridiales bacterium canine oral taxon 027 |
| Z1 | 77 | 108 | 0.18 | 76124 | NCBI-3-16S@U13037.1 | [Eubacterium] minutum | Bacteria | Bacillota | Clostridia | Eubacteriales | Eubacteriales Family XIII. Incertae Sedis |  | [Eubacterium] minutum |
| Z1 | 78 | 106 | 0.18 | 2086579 | NCBI-3-16S@LT985388.1 | Bacteroides sp. Marseille-P3684 | Bacteria | Bacteroidota | Bacteroidia | Bacteroidales | Bacteroidaceae | Bacteroides | Bacteroides sp. Marseille-P3684 |
| Z1 | 79 | 105 | 0.18 | 1628085 | NCBI-3-16S@NR_151982.1 | Agathobaculum butyriciproducens | Bacteria | Bacillota | Clostridia | Eubacteriales | Butyricicoccaceae | Agathobaculum | Agathobaculum butyriciproducens |
| Z1 | 80 | 104 | 0.17 | 1972642 | NCBI-3-16S@MW599793.1 | Sphaerochaeta sp. | Bacteria | Spirochaetota | Spirochaetia | Spirochaetales | Sphaerochaetaceae | Sphaerochaeta | Sphaerochaeta sp. |
| Z1 | 81 | 104 | 0.17 | 1903262 | NCBI-3-16S@NR_179564.1 | Bacteroides ndongoniae | Bacteria | Bacteroidota | Bacteroidia | Bacteroidales | Bacteroidaceae | Bacteroides | Bacteroides ndongoniae |
| Z1 | 82 | 102 | 0.17 | 3032870 | NCBI-3-16S@MK929052.1 | Lepagella muris | Bacteria | Bacteroidota | Bacteroidia | Bacteroidales | Muribaculaceae | Lepagella | Lepagella muris |
| Z1 | 83 | 102 | 0.17 | 2094145 | NCBI-3-16S@NR_179663.1 | Atopobium massiliense | Bacteria | Actinomycetota | Coriobacteriia | Coriobacteriales | Atopobiaceae | Atopobium | Atopobium massiliense |
| Z1 | 84 | 102 | 0.17 | 1929886 | NCBI-3-16S@MK287687.1 | Eggerthella sp. | Bacteria | Actinomycetota | Coriobacteriia | Eggerthellales | Eggerthellaceae | Eggerthella | Eggerthella sp. |
| Z1 | 85 | 101 | 0.17 | 1034346 | NCBI-3-16S@NR_125593.1 | Dielma fastidiosa | Bacteria | Bacillota | Erysipelotrichia | Erysipelotrichales | Erysipelotrichaceae | Dielma | Dielma fastidiosa |
| Z1 | 86 | 100 | 0.17 | 2211183 | NCBI-3-16S@NR_180148.1 | Evtepia gabavorous | Bacteria | Bacillota | Clostridia | Eubacteriales |  | Evtepia | Evtepia gabavorous |
| Z1 | 87 | 99 | 0.17 | 1159221 | NCBI-3-16S@AB702935.1 | Clostridiales bacterium CIEAF 013 | Bacteria | Bacillota | Clostridia | Eubacteriales |  |  | Clostridiales bacterium CIEAF 013 |
| Z1 | 88 | 94 | 0.16 | 1297424 | NCBI-3-16S@NR_125464.1 | Anaerobacterium chartisolvens | Bacteria | Bacillota | Clostridia | Eubacteriales | Oscillospiraceae | Anaerobacterium | Anaerobacterium chartisolvens |
| Z1 | 89 | 93 | 0.16 | 1155414 | NCBI-3-16S@JN688037.1 | bacterium enrichment culture clone M235 | Bacteria |  |  |  |  |  | bacterium enrichment culture clone M235 |
| Z1 | 90 | 92 | 0.15 | 1155412 | NCBI-3-16S@JN688035.1 | bacterium enrichment culture clone M153 | Bacteria |  |  |  |  |  | bacterium enrichment culture clone M153 |
| Z1 | 91 | 92 | 0.15 | 1869337 | NCBI-3-16S@ON076560.1 | Parabacteroides sp. | Bacteria | Bacteroidota | Bacteroidia | Bacteroidales | Tannerellaceae | Parabacteroides | Parabacteroides sp. |
| Z1 | 92 | 91 | 0.15 | 1872092 | NCBI-3-16S@MZ310614.1 | Acetivibrio sp. | Bacteria | Bacillota | Clostridia | Eubacteriales | Oscillospiraceae | Acetivibrio | Acetivibrio sp. |
| Z1 | 93 | 90 | 0.15 | 1159223 | NCBI-3-16S@AB702937.1 | Clostridiales bacterium CIEAF 021 | Bacteria | Bacillota | Clostridia | Eubacteriales |  |  | Clostridiales bacterium CIEAF 021 |
| Z1 | 94 | 90 | 0.15 | 2049025 | NCBI-3-16S@MK287660.1 | Flavonifractor sp. | Bacteria | Bacillota | Clostridia | Eubacteriales | Oscillospiraceae | Flavonifractor | Flavonifractor sp. |
| Z1 | 95 | 88 | 0.15 | 2485926 | NCBI-3-16S@OM658553.1 | Atopobiaceae bacterium | Bacteria | Actinomycetota | Coriobacteriia | Coriobacteriales | Atopobiaceae |  | Atopobiaceae bacterium |
| Z1 | 96 | 88 | 0.15 | 671267 | NCBI-3-16S@NR_113195.1 | Phocaeicola sartorii | Bacteria | Bacteroidota | Bacteroidia | Bacteroidales | Bacteroidaceae | Phocaeicola | Phocaeicola sartorii |
| Z1 | 97 | 88 | 0.15 | 298183 | NCBI-3-16S@AY756145.2 | anaerobic bacterium Glu3 | Bacteria | Bacillota | Clostridia | Eubacteriales |  |  | anaerobic bacterium Glu3 |
| Z1 | 98 | 87 | 0.15 | 2585118 | NCBI-3-16S@ON705225.1 | Alistipes communis | Bacteria | Bacteroidota | Bacteroidia | Bacteroidales | Rikenellaceae | Alistipes | Alistipes communis |
| Z1 | 99 | 86 | 0.14 | 569483 | NCBI-3-16S@FJ269072.1 | iron-reducing bacterium enrichment culture clone HN109 | Bacteria |  |  |  |  |  | iron-reducing bacterium enrichment culture clone HN109 |
| Z1 | 100 | 86 | 0.14 | 2202144 | NCBI-3-16S@ON560939.1 | Spirochaetota bacterium | Bacteria | Spirochaetota |  |  |  |  | Spirochaetota bacterium |
| Z1 | 101 | 85 | 0.14 | 454155 | NCBI-3-16S@NR_113078.1 | Paraprevotella xylaniphila | Bacteria | Bacteroidota | Bacteroidia | Bacteroidales | Prevotellaceae | Paraprevotella | Paraprevotella xylaniphila |
| Z1 | 102 | 83 | 0.14 | 2584469 | NCBI-3-16S@NR_180580.1 | Olsenella lakotia | Bacteria | Actinomycetota | Coriobacteriia | Coriobacteriales | Atopobiaceae | Olsenella | Olsenella lakotia |
| Z1 | 103 | 83 | 0.14 | 1352374 | NCBI-3-16S@KF156793.1 | Ruminococcus sp. YE78 | Bacteria | Bacillota | Clostridia | Eubacteriales | Oscillospiraceae | Ruminococcus | Ruminococcus sp. YE78 |
| Z1 | 104 | 82 | 0.14 | 1642647 | NCBI-3-16S@NR_148807.1 | Proteiniphilum saccharofermentans | Bacteria | Bacteroidota | Bacteroidia | Bacteroidales | Dysgonomonadaceae | Proteiniphilum | Proteiniphilum saccharofermentans |
| Z1 | 105 | 81 | 0.14 | 1954376 | NCBI-3-16S@MZ310618.1 | Caproiciproducens sp. | Bacteria | Bacillota | Clostridia | Eubacteriales | Acutalibacteraceae | Caproiciproducens | Caproiciproducens sp. |
| Z1 | 106 | 79 | 0.13 | 2030927 | NCBI-3-16S@MK929084.1 | Bacteroidales bacterium | Bacteria | Bacteroidota | Bacteroidia | Bacteroidales |  |  | Bacteroidales bacterium |
| Z1 | 107 | 76 | 0.13 | 1515 | NCBI-3-16S@NR_113157.1 | Acetivibrio thermocellus | Bacteria | Bacillota | Clostridia | Eubacteriales | Oscillospiraceae | Acetivibrio | Acetivibrio thermocellus |
| Z1 | 108 | 74 | 0.12 | 2779352 | NCBI-3-16S@NR_180619.1 | Pseudoflavonifractor gallinarum | Bacteria | Bacillota | Clostridia | Eubacteriales | Oscillospiraceae | Pseudoflavonifractor | Pseudoflavonifractor gallinarum |
| Z1 | 109 | 74 | 0.12 | 742727 | NCBI-3-16S@NR_113070.1 | Bacteroides oleiciplenus YIT 12058 | Bacteria | Bacteroidota | Bacteroidia | Bacteroidales | Bacteroidaceae | Bacteroides | Bacteroides oleiciplenus |
| Z1 | 110 | 74 | 0.12 | 1197717 | NCBI-3-16S@MN537495.1 | Cloacibacillus porcorum | Bacteria | Synergistota | Synergistia | Synergistales | Synergistaceae | Cloacibacillus | Cloacibacillus porcorum |
| Z1 | 111 | 74 | 0.12 | 1841865 | NCBI-3-16S@NR_144747.1 | Mediterranea massiliensis | Bacteria | Bacteroidota | Bacteroidia | Bacteroidales | Bacteroidaceae | Mediterranea | Mediterranea massiliensis |
| Z1 | 112 | 72 | 0.12 | 2606626 | NCBI-3-16S@NR_180830.1 | Sodaliphilus pleomorphus | Bacteria | Bacteroidota | Bacteroidia | Bacteroidales | Muribaculaceae | Sodaliphilus | Sodaliphilus pleomorphus |
| Z1 | 113 | 72 | 0.12 | 915171 | NCBI-3-16S@HQ452853.1 | Clostridiales bacterium 30-4c | Bacteria | Bacillota | Clostridia | Eubacteriales |  |  | Clostridiales bacterium 30-4c |
| Z1 | 114 | 71 | 0.12 | 1898206 | NCBI-3-16S@OQ150036.1 | Spirochaetaceae bacterium | Bacteria | Spirochaetota | Spirochaetia | Spirochaetales | Spirochaetaceae |  | Spirochaetaceae bacterium |
| Z1 | 115 | 70 | 0.12 | 246199 | NCBI-3-16S@AY445594.1 | Ruminococcus albus 8 | Bacteria | Bacillota | Clostridia | Eubacteriales | Oscillospiraceae | Ruminococcus | Ruminococcus albus |
| Z1 | 116 | 70 | 0.12 | 1588753 | NCBI-3-16S@KP192306.1 | Coriobacteriales bacterium DNF00809 | Bacteria | Actinomycetota | Coriobacteriia | Coriobacteriales |  |  | Coriobacteriales bacterium DNF00809 |
| Z1 | 117 | 69 | 0.12 | 5082 | NCBI-3-18S@KP190122.1 | Penicillium roqueforti | Eukaryota | Ascomycota | Eurotiomycetes | Eurotiales | Aspergillaceae | Penicillium | Penicillium roqueforti |
| Z1 | 118 | 69 | 0.12 | 2894156 | NCBI-3-16S@NR_184626.1 | Leptogranulimonas caecicola | Bacteria | Actinomycetota | Coriobacteriia | Coriobacteriales | Kribbibacteriaceae | Leptogranulimonas | Leptogranulimonas caecicola |
| Z1 | 119 | 69 | 0.12 | 2779354 | NCBI-3-16S@NR_180622.1 | Gemmiger gallinarum | Bacteria | Bacillota | Clostridia | Eubacteriales |  | Gemmiger | Gemmiger gallinarum |
| Z1 | 120 | 67 | 0.11 | 1898203 | NCBI-3-16S@MH699349.1 | Lachnospiraceae bacterium | Bacteria | Bacillota | Clostridia | Lachnospirales | Lachnospiraceae |  | Lachnospiraceae bacterium |
| Z1 | 121 | 67 | 0.11 | 1151583 | NCBI-3-16S@JN713550.1 | Spirochaeta sp. canine oral taxon 379 | Bacteria | Spirochaetota | Spirochaetia | Spirochaetales | Spirochaetaceae | Spirochaeta | Spirochaeta sp. canine oral taxon 379 |
| Z1 | 122 | 67 | 0.11 | 2897707 | NCBI-3-16S@NR_144748.1 | Merdimmobilis hominis | Bacteria | Bacillota | Clostridia | Eubacteriales | Oscillospiraceae | Merdimmobilis | Merdimmobilis hominis |
| Z1 | 123 | 64 | 0.11 | 142586 | NCBI-3-16S@OK272455.1 | Eubacterium sp. | Bacteria | Bacillota | Clostridia | Eubacteriales | Eubacteriaceae | Eubacterium | Eubacterium sp. |
| Z1 | 124 | 63 | 0.11 | 1236512 | NCBI-3-16S@NR_113072.1 | Bacteroides rodentium JCM 16496 | Bacteria | Bacteroidota | Bacteroidia | Bacteroidales | Bacteroidaceae | Bacteroides | Bacteroides rodentium |
| Z1 | 125 | 62 | 0.10 | 2049021 | NCBI-3-16S@ON831581.1 | Butyricicoccus sp. | Bacteria | Bacillota | Clostridia | Eubacteriales | Butyricicoccaceae | Butyricicoccus | Butyricicoccus sp. |
| Z1 | 126 | 62 | 0.10 | 1647718 | NCBI-3-16S@KR232873.1 | Propionibacterium sp. S342 | Bacteria | Actinomycetota | Actinomycetes | Propionibacteriales | Propionibacteriaceae | Propionibacterium | Propionibacterium sp. S342 |
| Z1 | 127 | 61 | 0.10 | 762968 | NCBI-3-16S@NR_041626.1 | Paraprevotella clara YIT 11840 | Bacteria | Bacteroidota | Bacteroidia | Bacteroidales | Prevotellaceae | Paraprevotella | Paraprevotella clara |
| Z1 | 128 | 60 | 0.10 | 1871015 | NCBI-3-16S@LT598575.1 | Pseudoflavonifractor sp. Marseille-P3106 | Bacteria | Bacillota | Clostridia | Eubacteriales | Oscillospiraceae | Pseudoflavonifractor | Pseudoflavonifractor sp. Marseille-P3106 |
| Z1 | 129 | 60 | 0.10 | 2763659 | NCBI-3-16S@NR_181390.1 | Fumia xinanensis | Bacteria | Bacillota | Clostridia | Eubacteriales | Oscillospiraceae | Fumia | Fumia xinanensis |
| Z1 | 130 | 59 | 0.10 | 292800 | NCBI-3-16S@MN055958.1 | Flavonifractor plautii | Bacteria | Bacillota | Clostridia | Eubacteriales | Oscillospiraceae | Flavonifractor | Flavonifractor plautii |
| Z1 | 131 | 59 | 0.10 | 1155415 | NCBI-3-16S@JN688038.1 | bacterium enrichment culture clone M244 | Bacteria |  |  |  |  |  | bacterium enrichment culture clone M244 |
| Z1 | 132 | 59 | 0.10 | 1577240 | NCBI-3-16S@KM462152.1 | Bacteroides sp. feline oral taxon 308 | Bacteria | Bacteroidota | Bacteroidia | Bacteroidales | Bacteroidaceae | Bacteroides | Bacteroides sp. feline oral taxon 308 |
| Z1 | 133 | 58 | 0.10 | 1926877 | NCBI-3-16S@MG551268.2 | Proteiniphilum sp. | Bacteria | Bacteroidota | Bacteroidia | Bacteroidales | Dysgonomonadaceae | Proteiniphilum | Proteiniphilum sp. |
| Z1 | 134 | 58 | 0.10 | 1156936 | NCBI-3-16S@JQ404436.1 | Clostridium sp. WSC-9-7 | Bacteria | Bacillota | Clostridia | Eubacteriales | Clostridiaceae | Clostridium | Clostridium sp. WSC-9-7 |
| Z1 | 135 | 57 | 0.10 | 545496 | NCBI-3-16S@EU815223.1 | Ruminococcus sp. NML 00-0124 | Bacteria | Bacillota | Clostridia | Eubacteriales | Oscillospiraceae | Ruminococcus | Ruminococcus sp. NML 00-0124 |
| Z1 | 136 | 57 | 0.10 | 1841866 | NCBI-3-16S@NR_179526.1 | Oscillibacter massiliensis | Bacteria | Bacillota | Clostridia | Eubacteriales | Oscillospiraceae | Oscillibacter | Oscillibacter massiliensis |
| Z1 | 137 | 57 | 0.10 | 1968902 | NCBI-3-16S@OK067651.1 | Gordonibacter sp. | Bacteria | Actinomycetota | Coriobacteriia | Eggerthellales | Eggerthellaceae | Gordonibacter | Gordonibacter sp. |
| Z1 | 138 | 56 | 0.09 | 2070686 | NCBI-3-16S@NR_179114.1 | Enteroscipio rubneri | Bacteria | Actinomycetota | Coriobacteriia | Eggerthellales | Eggerthellaceae | Enteroscipio | Enteroscipio rubneri |
| Z1 | 139 | 56 | 0.09 | 666493 | NCBI-3-16S@GQ377117.1 | bacterium enrichment culture clone DPHB07 | Bacteria |  |  |  |  |  | bacterium enrichment culture clone DPHB07 |
| Z1 | 140 | 55 | 0.09 | 1556 | NCBI-3-16S@NR_117601.1 | Gottschalkia acidurici | Bacteria | Bacillota | Tissierellia | Tissierellales | Gottschalkiaceae | Gottschalkia | Gottschalkia acidurici |
| Z1 | 141 | 55 | 0.09 | 100176 | NCBI-3-16S@NR_025025.1 | Papillibacter cinnamivorans | Bacteria | Bacillota | Clostridia | Eubacteriales | Oscillospiraceae | Papillibacter | Papillibacter cinnamivorans |
| Z1 | 142 | 54 | 0.09 | 1987501 | NCBI-3-16S@NR_179626.1 | Massiliimalia timonensis | Bacteria | Bacillota | Clostridia | Eubacteriales | Oscillospiraceae | Massiliimalia | Massiliimalia timonensis |
| Z1 | 143 | 54 | 0.09 | 246787 | NCBI-3-16S@OR945891.1 | Bacteroides cellulosilyticus | Bacteria | Bacteroidota | Bacteroidia | Bacteroidales | Bacteroidaceae | Bacteroides | Bacteroides cellulosilyticus |
| Z1 | 144 | 53 | 0.09 | 411467 | NCBI-3-16S@NR_025670.1 | Pseudoflavonifractor capillosus ATCC 29799 | Bacteria | Bacillota | Clostridia | Eubacteriales | Oscillospiraceae | Pseudoflavonifractor | Pseudoflavonifractor capillosus |
| Z1 | 145 | 53 | 0.09 | 2763662 | NCBI-3-16S@NR_181382.1 | Yeguia hominis | Bacteria | Bacillota | Clostridia | Eubacteriales | Yeguiaceae | Yeguia | Yeguia hominis |
| Z1 | 146 | 53 | 0.09 | 349096 | NCBI-3-16S@NR_043658.1 | Pectinatus haikarae | Bacteria | Bacillota | Negativicutes | Selenomonadales | Selenomonadaceae | Pectinatus | Pectinatus haikarae |
| Z1 | 147 | 53 | 0.09 | 712955 | NCBI-3-16S@HM099644.1 | Clostridiales bacterium oral taxon F32 | Bacteria | Bacillota | Clostridia | Eubacteriales |  |  | Clostridiales bacterium oral taxon F32 |
| Z1 | 148 | 52 | 0.09 | 2163169 | NCBI-3-16S@MN081684.1 | Kineothrix sp. | Bacteria | Bacillota | Clostridia | Lachnospirales | Lachnospiraceae | Kineothrix | Kineothrix sp. |
| Z1 | 149 | 52 | 0.09 | 1533824 | NCBI-3-16S@KM277367.1 | Clostridium sp. AUH-JLC108 | Bacteria | Bacillota | Clostridia | Eubacteriales | Clostridiaceae | Clostridium | Clostridium sp. AUH-JLC108 |
| Z1 | 150 | 52 | 0.09 | 328812 | NCBI-3-16S@MT902993.1 | Parabacteroides goldsteinii | Bacteria | Bacteroidota | Bacteroidia | Bacteroidales | Tannerellaceae | Parabacteroides | Parabacteroides goldsteinii |
| Z1 | 151 | 51 | 0.09 | 2585119 | NCBI-3-16S@NR_179296.1 | Alistipes dispar | Bacteria | Bacteroidota | Bacteroidia | Bacteroidales | Rikenellaceae | Alistipes | Alistipes dispar |
| Z1 | 152 | 50 | 0.08 | 2866587 | NCBI-3-16S@OU557123.1 | Paraeggerthella sp. Marseille-Q4926 | Bacteria | Actinomycetota | Coriobacteriia | Eggerthellales | Eggerthellaceae | Paraeggerthella | Paraeggerthella sp. Marseille-Q4926 |
| Z1 | 153 | 50 | 0.08 | 1969738 | NCBI-3-16S@MH463786.1 | Butyricimonas sp. | Bacteria | Bacteroidota | Bacteroidia | Bacteroidales | Odoribacteraceae | Butyricimonas | Butyricimonas sp. |
| Z1 | 154 | 50 | 0.08 | 1411150 | NCBI-3-16S@HG531807.1 | Sphaerochaeta sp. DSM 26296 | Bacteria | Spirochaetota | Spirochaetia | Spirochaetales | Sphaerochaetaceae | Sphaerochaeta | Sphaerochaeta sp. DSM 26296 |
| Z1 | 155 | 49 | 0.08 | 1796613 | NCBI-3-16S@OK626630.1 | Bacteroides caecimuris | Bacteria | Bacteroidota | Bacteroidia | Bacteroidales | Bacteroidaceae | Bacteroides | Bacteroides caecimuris |
| Z1 | 156 | 48 | 0.08 | 2981770 | NCBI-3-16S@OK510325.1 | Gallintestinimicrobium propionicum | Bacteria | Bacillota | Clostridia | Lachnospirales | Lachnospiraceae | Gallintestinimicrobium | Gallintestinimicrobium propionicum |
| Z1 | 157 | 48 | 0.08 | 320502 | NCBI-3-16S@OK626616.1 | Acetivibrio alkalicellulosi | Bacteria | Bacillota | Clostridia | Eubacteriales | Oscillospiraceae | Acetivibrio | Acetivibrio alkalicellulosi |
| Z1 | 158 | 47 | 0.08 | 1841857 | NCBI-3-16S@NR_144745.1 | Culturomica massiliensis | Bacteria | Bacteroidota | Bacteroidia | Bacteroidales | Odoribacteraceae | Culturomica | Culturomica massiliensis |
| Z1 | 159 | 46 | 0.08 | 2763654 | NCBI-3-16S@NR_181383.1 | Luoshenia tenuis | Bacteria | Bacillota | Clostridia | Christensenellales | Christensenellaceae | Luoshenia | Luoshenia tenuis |
| Z1 | 160 | 46 | 0.08 | 213810 | NCBI-3-16S@NR_102884.1 | Ruminococcus champanellensis 18P13 = JCM 17042 | Bacteria | Bacillota | Clostridia | Eubacteriales | Oscillospiraceae | Ruminococcus | Ruminococcus champanellensis |
| Z1 | 161 | 46 | 0.08 | 1501392 | NCBI-3-16S@NR_173687.1 | Coprobacter secundus | Bacteria | Bacteroidota | Bacteroidia | Bacteroidales | Barnesiellaceae | Coprobacter | Coprobacter secundus |
| Z1 | 162 | 44 | 0.07 | 393757 | NCBI-3-16S@DQ677001.1 | iron-reducing enrichment clone Cl-A9 | Bacteria |  |  |  |  |  | iron-reducing enrichment clone Cl-A9 |
| Z1 | 163 | 43 | 0.07 | 915176 | NCBI-3-16S@HQ452851.1 | Clostridiales bacterium 53-4c | Bacteria | Bacillota | Clostridia | Eubacteriales |  |  | Clostridiales bacterium 53-4c |
| Z1 | 164 | 43 | 0.07 | 3047475 | NCBI-3-16S@OQ998951.1 | Gordonibacter sp. KGMB12511 | Bacteria | Actinomycetota | Coriobacteriia | Eggerthellales | Eggerthellaceae | Gordonibacter | Gordonibacter faecis |
| Z1 | 165 | 43 | 0.07 | 40518 | NCBI-3-16S@X85099.1 | Ruminococcus bromii | Bacteria | Bacillota | Clostridia | Eubacteriales | Oscillospiraceae | Ruminococcus | Ruminococcus bromii |
| Z1 | 166 | 42 | 0.07 | 1907658 | NCBI-3-16S@NR_179567.1 | Bacteroides ilei | Bacteria | Bacteroidota | Bacteroidia | Bacteroidales | Bacteroidaceae | Bacteroides | Bacteroides ilei |
| Z1 | 167 | 42 | 0.07 | 2707299 | NCBI-3-16S@NR_179369.1 | Adlercreutzia hattorii | Bacteria | Actinomycetota | Coriobacteriia | Eggerthellales | Eggerthellaceae | Adlercreutzia | Adlercreutzia hattorii |
| Z1 | 168 | 40 | 0.07 | 1159222 | NCBI-3-16S@AB702936.1 | Clostridiales bacterium CIEAF 019 | Bacteria | Bacillota | Clostridia | Eubacteriales |  |  | Clostridiales bacterium CIEAF 019 |
| Z1 | 169 | 40 | 0.07 | 871324 | NCBI-3-16S@MW397775.1 | Bacteroides stercorirosoris | Bacteria | Bacteroidota | Bacteroidia | Bacteroidales | Bacteroidaceae | Bacteroides | Bacteroides stercorirosoris |
| Z1 | 170 | 39 | 0.07 | 3046383 | NCBI-3-16S@ON706274.1 | Aristaeella lactis | Bacteria | Bacillota | Clostridia | Eubacteriales | Aristaeellaceae | Aristaeella | Aristaeella lactis |
| Z1 | 171 | 39 | 0.07 | 351091 | NCBI-3-16S@MH282444.1 | Oscillibacter valericigenes | Bacteria | Bacillota | Clostridia | Eubacteriales | Oscillospiraceae | Oscillibacter | Oscillibacter valericigenes |
| Z1 | 172 | 39 | 0.07 | 2011094 | NCBI-3-16S@MN055926.1 | Coriobacteriaceae bacterium | Bacteria | Actinomycetota | Coriobacteriia | Coriobacteriales | Coriobacteriaceae |  | Coriobacteriaceae bacterium |
| Z1 | 173 | 39 | 0.07 | 641768 | NCBI-3-16S@FJ889653.1 | Ruminococcus sp. ZS2-15 | Bacteria | Bacillota | Clostridia | Eubacteriales | Oscillospiraceae | Ruminococcus | Ruminococcus sp. ZS2-15 |
| Z1 | 174 | 38 | 0.06 | 1240100 | NCBI-3-16S@AB752501.1 | Coriobacteriaceae bacterium SNR48-44 | Bacteria | Actinomycetota | Coriobacteriia | Coriobacteriales | Coriobacteriaceae |  | Coriobacteriaceae bacterium SNR48-44 |
| Z1 | 175 | 38 | 0.06 | 28113 | NCBI-3-16S@JN713478.1 | Bacteroides heparinolyticus | Bacteria | Bacteroidota | Bacteroidia | Bacteroidales | Bacteroidaceae | Bacteroides | Bacteroides heparinolyticus |
| Z1 | 176 | 38 | 0.06 | 2046249 | NCBI-3-16S@MT312829.1 | Centipeda sp. (in: firmicutes) | Bacteria | Bacillota | Negativicutes | Selenomonadales | Selenomonadaceae | Centipeda | Centipeda sp. (in: firmicutes) |
| Z1 | 177 | 38 | 0.06 | 915170 | NCBI-3-16S@HQ452852.1 | Clostridiales bacterium 24-4c | Bacteria | Bacillota | Clostridia | Eubacteriales |  |  | Clostridiales bacterium 24-4c |
| Z1 | 178 | 38 | 0.06 | 537405 | NCBI-3-16S@EU728724.1 | Lactobacillales bacterium DJF_B280 | Bacteria | Bacillota | Bacilli | Lactobacillales |  |  | Lactobacillales bacterium DJF_B280 |
| Z1 | 179 | 38 | 0.06 | 399354 | NCBI-3-16S@DQ833395.1 | Sphaerochaeta sp. 'Grapes TMA14' | Bacteria | Spirochaetota | Spirochaetia | Spirochaetales | Sphaerochaetaceae | Sphaerochaeta | Sphaerochaeta sp. 'Grapes TMA14' |
| Z1 | 180 | 38 | 0.06 | 1647716 | NCBI-3-16S@KR232852.1 | Porphyromonadaceae bacterium S190 | Bacteria | Bacteroidota | Bacteroidia | Bacteroidales | Porphyromonadaceae |  | Porphyromonadaceae bacterium S190 |
| Z1 | 181 | 38 | 0.06 | 1462571 | NCBI-3-16S@NR_148822.1 | Bacteroides gallinaceum | Bacteria | Bacteroidota | Bacteroidia | Bacteroidales | Bacteroidaceae | Bacteroides | Bacteroides gallinaceum |
| Z1 | 182 | 37 | 0.06 | 2834112 | NCBI-3-16S@NR_181731.1 | Bacteroides propionicigenes | Bacteria | Bacteroidota | Bacteroidia | Bacteroidales | Bacteroidaceae | Bacteroides | Bacteroides propionicigenes |
| Z1 | 183 | 37 | 0.06 | 2981726 | NCBI-3-16S@OK510342.1 | Hominimerdicola aceti | Bacteria | Bacillota | Clostridia | Eubacteriales | Oscillospiraceae | Hominimerdicola | Hominimerdicola aceti |
| Z1 | 184 | 37 | 0.06 | 28111 | NCBI-3-16S@MT902967.1 | Bacteroides eggerthii | Bacteria | Bacteroidota | Bacteroidia | Bacteroidales | Bacteroidaceae | Bacteroides | Bacteroides eggerthii |
| Z1 | 185 | 37 | 0.06 | 1030127 | NCBI-3-16S@JF813174.1 | Bacteroides sp. dnLKV2 | Bacteria | Bacteroidota | Bacteroidia | Bacteroidales | Bacteroidaceae | Bacteroides | Bacteroides sp. dnLKV2 |
| Z1 | 186 | 36 | 0.06 | 376804 | NCBI-3-16S@NR_041446.1 | Phocaeicola barnesiae | Bacteria | Bacteroidota | Bacteroidia | Bacteroidales | Bacteroidaceae | Phocaeicola | Phocaeicola barnesiae |
| Z1 | 187 | 36 | 0.06 | 377137 | NCBI-3-16S@AM236337.1 | spirochete endosymbiont of a lucinid bivalve | Bacteria | Spirochaetota | Spirochaetia | Spirochaetales |  |  | spirochete endosymbiont of a lucinid bivalve |
| Z1 | 188 | 36 | 0.06 | 747602 | NCBI-3-16S@AB551425.1 | Clostridium sp. TG60-81 | Bacteria | Bacillota | Clostridia | Eubacteriales | Clostridiaceae | Clostridium | Clostridium sp. TG60-81 |
| Z1 | 189 | 35 | 0.06 | 889071 | NCBI-3-16S@HQ222293.1 | Clostridium sp. enrichment culture clone VanCtr97 | Bacteria | Bacillota | Clostridia | Eubacteriales | Clostridiaceae | Clostridium | Clostridium sp. enrichment culture clone VanCtr97 |
| Z1 | 190 | 35 | 0.06 | 1720203 | NCBI-3-16S@NR_169358.1 | Butyricimonas phoceensis | Bacteria | Bacteroidota | Bacteroidia | Bacteroidales | Odoribacteraceae | Butyricimonas | Butyricimonas phoceensis |
| Z1 | 191 | 35 | 0.06 | 1288121 | NCBI-3-16S@NR_118219.1 | Alistipes senegalensis | Bacteria | Bacteroidota | Bacteroidia | Bacteroidales | Rikenellaceae | Alistipes | Alistipes senegalensis |
| Z1 | 192 | 34 | 0.06 | 569511 | NCBI-3-16S@FJ269048.1 | iron-reducing bacterium enrichment culture clone HN7 | Bacteria |  |  |  |  |  | iron-reducing bacterium enrichment culture clone HN7 |
| Z1 | 193 | 33 | 0.06 | 1093223 | NCBI-3-16S@JN613166.1 | Anaerovibrio sp. 656 | Bacteria | Bacillota | Negativicutes | Selenomonadales | Selenomonadaceae | Anaerovibrio | Anaerovibrio sp. 656 |
| Z1 | 194 | 33 | 0.06 | 78930 | NCBI-3-16S@AJ009450.1 | chimeric sequence SJA-7 | Bacteria |  |  |  |  |  | chimeric sequence SJA-7 |
| Z1 | 195 | 33 | 0.06 | 46506 | NCBI-3-16S@OR554138.1 | Bacteroides stercoris | Bacteria | Bacteroidota | Bacteroidia | Bacteroidales | Bacteroidaceae | Bacteroides | Bacteroides stercoris |
| Z1 | 196 | 33 | 0.06 | 1159215 | NCBI-3-16S@AB702928.1 | Clostridiales bacterium CIEAF 020 | Bacteria | Bacillota | Clostridia | Eubacteriales |  |  | Clostridiales bacterium CIEAF 020 |
| Z1 | 197 | 33 | 0.06 | 2966552 | NCBI-3-16S@ON921083.1 | Thermocaproicibacter melissae | Bacteria | Bacillota | Clostridia | Eubacteriales | Oscillospiraceae | Thermocaproicibacter | Thermocaproicibacter melissae |
| Z1 | 198 | 32 | 0.05 | 2763053 | NCBI-3-16S@MT905206.1 | Lawsonibacter hominis | Bacteria | Bacillota | Clostridia | Eubacteriales | Oscillospiraceae | Lawsonibacter | Lawsonibacter hominis |
| Z1 | 199 | 32 | 0.05 | 1297617 | NCBI-3-16S@MZ310684.1 | Intestinimonas butyriciproducens | Bacteria | Bacillota | Clostridia | Eubacteriales |  | Intestinimonas | Intestinimonas butyriciproducens |
| Z1 | 200 | 32 | 0.05 | 1898111 | NCBI-3-16S@MZ262878.1 | Cryomorphaceae bacterium | Bacteria | Bacteroidota | Flavobacteriia | Flavobacteriales | Cryomorphaceae |  | Cryomorphaceae bacterium |
| Z1 | 201 | 32 | 0.05 | 2763660 | NCBI-3-16S@NR_181384.1 | Feifania hominis | Bacteria | Bacillota | Clostridia | Eubacteriales | Feifaniaceae | Feifania | Feifania hominis |
| Z1 | 202 | 31 | 0.05 | 39490 | NCBI-3-16S@KT221541.1 | Eubacterium ramulus | Bacteria | Bacillota | Clostridia | Eubacteriales | Eubacteriaceae | Eubacterium | Eubacterium ramulus |
| Z1 | 203 | 31 | 0.05 | 1335613 | NCBI-3-16S@MK544835.1 | Gordonibacter urolithinfaciens | Bacteria | Actinomycetota | Coriobacteriia | Eggerthellales | Eggerthellaceae | Gordonibacter | Gordonibacter urolithinfaciens |
| Z1 | 204 | 30 | 0.05 | 1033732 | NCBI-3-16S@NR_118219.1 | Alistipes senegalensis JC50 | Bacteria | Bacteroidota | Bacteroidia | Bacteroidales | Rikenellaceae | Alistipes | Alistipes senegalensis |
| Z1 | 205 | 30 | 0.05 | 1347151 | NCBI-3-16S@KF030226.1 | Petrimonas sp. canine oral taxon 434 | Bacteria | Bacteroidota | Bacteroidia | Bacteroidales | Dysgonomonadaceae | Petrimonas | Petrimonas sp. canine oral taxon 434 |
| Z1 | 206 | 30 | 0.05 | 1972642 | NCBI-3-16S@MG696667.1 | Sphaerochaeta sp. | Bacteria | Spirochaetota | Spirochaetia | Spirochaetales | Sphaerochaetaceae | Sphaerochaeta | Sphaerochaeta sp. |
| Z1 | 207 | 30 | 0.05 | 1918636 | NCBI-3-16S@MN081649.1 | Acutalibacter sp. | Bacteria | Bacillota | Clostridia | Eubacteriales | Acutalibacteraceae | Acutalibacter | Acutalibacter sp. |
| Z1 | 208 | 30 | 0.05 | 692055 | NCBI-3-16S@FJ808609.1 | Clostridium sp. 6-16 | Bacteria | Bacillota | Clostridia | Eubacteriales | Clostridiaceae | Clostridium | Clostridium sp. 6-16 |
| Z1 | 209 | 29 | 0.05 | 1977263 | NCBI-3-16S@MK287690.1 | Enterorhabdus sp. | Bacteria | Actinomycetota | Coriobacteriia | Eggerthellales | Eggerthellaceae | Enterorhabdus | Enterorhabdus sp. |
| Z1 | 210 | 29 | 0.05 | 2763656 | NCBI-3-16S@NR_181387.1 | Guopingia tenuis | Bacteria | Bacillota | Clostridia | Christensenellales | Christensenellaceae | Guopingia | Guopingia tenuis |
| Z1 | 211 | 29 | 0.05 | 371600 | NCBI-3-16S@AM230649.1 | Bacteroides sp. XB44A | Bacteria | Bacteroidota | Bacteroidia | Bacteroidales | Bacteroidaceae | Bacteroides | Bacteroides sp. XB44A |
| Z1 | 212 | 28 | 0.05 | 1211813 | NCBI-3-16S@NR_144706.1 | Alistipes ihumii AP11 | Bacteria | Bacteroidota | Bacteroidia | Bacteroidales | Rikenellaceae | Alistipes | Alistipes ihumii |
| Z1 | 213 | 28 | 0.05 | 2763052 | NCBI-3-16S@MT905207.1 | Lawsonibacter faecis | Bacteria | Bacillota | Clostridia | Eubacteriales | Oscillospiraceae | Lawsonibacter | Lawsonibacter faecis |
| Z1 | 214 | 28 | 0.05 | 720554 | NCBI-3-16S@NR_102987.1 | Acetivibrio clariflavus DSM 19732 | Bacteria | Bacillota | Clostridia | Eubacteriales | Oscillospiraceae | Acetivibrio | Acetivibrio clariflavus |
| Z1 | 215 | 28 | 0.05 | 1898207 | NCBI-3-16S@ON714589.1 | Clostridiales bacterium | Bacteria | Bacillota | Clostridia | Eubacteriales |  |  | Clostridiales bacterium |
| Z1 | 216 | 27 | 0.05 | 31899 | NCBI-3-16S@L09180.1 | Caldicellulosiruptor bescii | Bacteria | Bacillota |  | Caldicellulosiruptorales | Caldicellulosiruptoraceae | Caldicellulosiruptor | Caldicellulosiruptor bescii |
| Z1 | 217 | 27 | 0.05 | 630799 | NCBI-3-16S@FJ799136.1 | bacterium enrichment culture clone EtOH-23 | Bacteria |  |  |  |  |  | bacterium enrichment culture clone EtOH-23 |
| Z1 | 218 | 27 | 0.05 | 1907662 | NCBI-3-16S@NR_148574.1 | Raoultibacter timonensis | Bacteria | Actinomycetota | Coriobacteriia | Eggerthellales | Eggerthellaceae | Raoultibacter | Raoultibacter timonensis |
| Z1 | 219 | 27 | 0.05 | 1965323 | NCBI-3-16S@MZ496396.1 | Robertkochia sp. | Bacteria | Bacteroidota | Flavobacteriia | Flavobacteriales | Flavobacteriaceae | Robertkochia | Robertkochia sp. |
| Z1 | 220 | 27 | 0.05 | 626929 | NCBI-3-16S@NR_113065.1 | Bacteroides clarus | Bacteria | Bacteroidota | Bacteroidia | Bacteroidales | Bacteroidaceae | Bacteroides | Bacteroides clarus |
| Z1 | 221 | 27 | 0.05 | 626930 | NCBI-3-16S@MW558146.1 | Bacteroides fluxus | Bacteria | Bacteroidota | Bacteroidia | Bacteroidales | Bacteroidaceae | Bacteroides | Bacteroides fluxus |
| Z1 | 222 | 27 | 0.05 | 328813 | NCBI-3-16S@OR945844.1 | Alistipes onderdonkii | Bacteria | Bacteroidota | Bacteroidia | Bacteroidales | Rikenellaceae | Alistipes | Alistipes onderdonkii |
| Z1 | 223 | 26 | 0.04 | 853 | NCBI-3-16S@OL587616.1 | Faecalibacterium prausnitzii | Bacteria | Bacillota | Clostridia | Eubacteriales | Oscillospiraceae | Faecalibacterium | Faecalibacterium prausnitzii |
| Z1 | 224 | 26 | 0.04 | 562 | NCBI-3-16S@KP276745.1 | Escherichia coli | Bacteria | Pseudomonadota | Gammaproteobacteria | Enterobacterales | Enterobacteriaceae | Escherichia | Escherichia coli |
| Z1 | 225 | 26 | 0.04 | 2086583 | NCBI-3-16S@NR_179661.1 | Pseudoruminococcus massiliensis | Bacteria | Bacillota | Clostridia | Eubacteriales | Acutalibacteraceae | Pseudoruminococcus | Pseudoruminococcus massiliensis |
| Z1 | 226 | 25 | 0.04 | 569500 | NCBI-3-16S@FJ269045.1 | iron-reducing bacterium enrichment culture clone HN3 | Bacteria |  |  |  |  |  | iron-reducing bacterium enrichment culture clone HN3 |
| Z1 | 227 | 25 | 0.04 | 537365 | NCBI-3-16S@EU728720.1 | Bacteroidaceae bacterium DJF_B220 | Bacteria | Bacteroidota | Bacteroidia | Bacteroidales | Bacteroidaceae |  | Bacteroidaceae bacterium DJF_B220 |
| Z1 | 228 | 25 | 0.04 | 1141812 | NCBI-3-16S@JQ599692.1 | Clostridium sp. enrichment culture clone 06-1235251-89 | Bacteria | Bacillota | Clostridia | Eubacteriales | Clostridiaceae | Clostridium | Clostridium sp. enrichment culture clone 06-1235251-89 |
| Z1 | 229 | 25 | 0.04 | 35830 | NCBI-3-16S@JQ820024.1 | Acetivibrio cellulolyticus | Bacteria | Bacillota | Clostridia | Eubacteriales | Oscillospiraceae | Acetivibrio | Acetivibrio cellulolyticus |
| Z1 | 230 | 25 | 0.04 | 1852385 | NCBI-3-16S@NR_173693.1 | Olsenella phocaeensis | Bacteria | Actinomycetota | Coriobacteriia | Coriobacteriales | Atopobiaceae | Olsenella | Olsenella phocaeensis |
| Z1 | 231 | 25 | 0.04 | 2576756 | NCBI-3-16S@NR_174324.1 | Caproicibacter fermentans | Bacteria | Bacillota | Clostridia | Eubacteriales | Acutalibacteraceae | Caproicibacter | Caproicibacter fermentans |
| Z1 | 232 | 24 | 0.04 | 1796620 | NCBI-3-16S@NR_144605.1 | Acutalibacter muris | Bacteria | Bacillota | Clostridia | Eubacteriales | Acutalibacteraceae | Acutalibacter | Acutalibacter muris |
| Z1 | 233 | 24 | 0.04 | 1030136 | NCBI-3-16S@JF813177.1 | Parabacteroides sp. dnLKV8 | Bacteria | Bacteroidota | Bacteroidia | Bacteroidales | Tannerellaceae | Parabacteroides | Parabacteroides sp. dnLKV8 |
| Z1 | 234 | 24 | 0.04 | 569452 | NCBI-3-16S@FJ269086.1 | iron-reducing bacterium enrichment culture clone HN-HFO10 | Bacteria |  |  |  |  |  | iron-reducing bacterium enrichment culture clone HN-HFO10 |
| Z1 | 235 | 24 | 0.04 | 1547597 | NCBI-3-16S@NR_178727.1 | Sanguibacteroides justesenii | Bacteria | Bacteroidota | Bacteroidia | Bacteroidales | Porphyromonadaceae | Sanguibacteroides | Sanguibacteroides justesenii |
| Z1 | 236 | 24 | 0.04 | 28112 | NCBI-3-16S@X73962.1 | Tannerella forsythia | Bacteria | Bacteroidota | Bacteroidia | Bacteroidales | Tannerellaceae | Tannerella | Tannerella forsythia |
| Z1 | 237 | 24 | 0.04 | 1151542 | NCBI-3-16S@JN713451.1 | Prevotella sp. canine oral taxon 284 | Bacteria | Bacteroidota | Bacteroidia | Bacteroidales | Prevotellaceae | Prevotella | Prevotella sp. canine oral taxon 284 |
| Z1 | 238 | 23 | 0.04 | 471189 | NCBI-3-16S@OM760840.1 | Gordonibacter pamelaeae | Bacteria | Actinomycetota | Coriobacteriia | Eggerthellales | Eggerthellaceae | Gordonibacter | Gordonibacter pamelaeae |
| Z1 | 239 | 23 | 0.04 | 1003345 | NCBI-3-16S@AB622816.1 | Clostridium sp. Culture-23 | Bacteria | Bacillota | Clostridia | Eubacteriales | Clostridiaceae | Clostridium | Clostridium sp. Culture-23 |
| Z1 | 240 | 23 | 0.04 | 2053611 | NCBI-3-16S@MW714943.1 | Selenomonas sp. | Bacteria | Bacillota | Negativicutes | Selenomonadales | Selenomonadaceae | Selenomonas | Selenomonas sp. |
| Z1 | 241 | 23 | 0.04 | 1577241 | NCBI-3-16S@KM461992.1 | Bacteroidia bacterium feline oral taxon 115 | Bacteria | Bacteroidota | Bacteroidia |  |  |  | Bacteroidia bacterium feline oral taxon 115 |
| Z1 | 242 | 22 | 0.04 | 885470 | NCBI-3-16S@FR691448.1 | Pedobacter sp. R-36962 | Bacteria | Bacteroidota | Sphingobacteriia | Sphingobacteriales | Sphingobacteriaceae | Pedobacter | Pedobacter sp. R-36962 |
| Z1 | 243 | 22 | 0.04 | 2764589 | NCBI-3-16S@MT905201.1 | Oscillibacter sp. BX15 | Bacteria | Bacillota | Clostridia | Eubacteriales | Oscillospiraceae | Oscillibacter | Oscillibacter sp. BX15 |
| Z1 | 244 | 22 | 0.04 | 1515 | NCBI-3-16S@FN555230.1 | Acetivibrio thermocellus | Bacteria | Bacillota | Clostridia | Eubacteriales | Oscillospiraceae | Acetivibrio | Acetivibrio thermocellus |
| Z1 | 245 | 22 | 0.04 | 326368 | NCBI-3-16S@DQ003622.1 | Prevotella genomosp. P8 oral clone MB3_P13 | Bacteria | Bacteroidota | Bacteroidia | Bacteroidales | Prevotellaceae | Prevotella | Prevotella genomosp. P8 oral clone MB3_P13 |
| Z1 | 246 | 22 | 0.04 | 2986072 | NCBI-3-16S@OX352005.1 | Candidatus Minimicrobia sp. IHU4 | Bacteria | Candidatus Saccharibacteria |  |  |  | Candidatus Minimicrobia | Candidatus Minimicrobia sp. IHU4 |
| Z1 | 247 | 22 | 0.04 | 2137881 | NCBI-3-16S@MK287767.1 | Coprobacillus sp. | Bacteria | Bacillota | Erysipelotrichia | Erysipelotrichales | Coprobacillaceae | Coprobacillus | Coprobacillus sp. |
| Z1 | 248 | 22 | 0.04 | 2799560 | NCBI-3-16S@LR861113.1 | Ruminococcaceae bacterium BL-4 | Bacteria | Bacillota | Clostridia | Eubacteriales | Oscillospiraceae |  | Ruminococcaceae bacterium BL-4 |
| Z1 | 249 | 22 | 0.04 | 1852371 | NCBI-3-16S@NR_144751.1 | Raoultibacter massiliensis | Bacteria | Actinomycetota | Coriobacteriia | Eggerthellales | Eggerthellaceae | Raoultibacter | Raoultibacter massiliensis |
| Z1 | 250 | 21 | 0.04 | 2049037 | NCBI-3-16S@OM658616.1 | Parasutterella sp. | Bacteria | Pseudomonadota | Betaproteobacteria | Burkholderiales | Sutterellaceae | Parasutterella | Parasutterella sp. |
| Z1 | 251 | 21 | 0.04 | 537292 | NCBI-3-16S@EU728708.1 | Parabacteroides sp. DJF_B086 | Bacteria | Bacteroidota | Bacteroidia | Bacteroidales | Tannerellaceae | Parabacteroides | Parabacteroides sp. DJF_B086 |
| Z1 | 252 | 21 | 0.04 | 2039241 | NCBI-3-16S@MK287718.1 | Anaerotignum sp. | Bacteria | Bacillota | Clostridia | Lachnospirales | Anaerotignaceae | Anaerotignum | Anaerotignum sp. |
| Z1 | 253 | 21 | 0.04 | 2049040 | NCBI-3-16S@MK287624.1 | Roseburia sp. | Bacteria | Bacillota | Clostridia | Lachnospirales | Lachnospiraceae | Roseburia | Roseburia sp. |
| Z1 | 254 | 21 | 0.04 | 1898207 | NCBI-3-16S@MK170164.1 | Clostridiales bacterium | Bacteria | Bacillota | Clostridia | Eubacteriales |  |  | Clostridiales bacterium |
| Z1 | 255 | 21 | 0.04 | 675999 | NCBI-3-16S@GQ503875.1 | bacterium enrichment culture clone Ecwsrb038 | Bacteria |  |  |  |  |  | bacterium enrichment culture clone Ecwsrb038 |
| Z1 | 256 | 21 | 0.04 | 2763658 | NCBI-3-16S@NR_181388.1 | Ligaoa zhengdingensis | Bacteria | Bacillota | Clostridia | Eubacteriales | Oscillospiraceae | Ligaoa | Ligaoa zhengdingensis |
| Z1 | 257 | 21 | 0.04 | 569465 | NCBI-3-16S@FJ269084.1 | iron-reducing bacterium enrichment culture clone HN-HFO4 | Bacteria |  |  |  |  |  | iron-reducing bacterium enrichment culture clone HN-HFO4 |
| Z1 | 258 | 21 | 0.04 | 1414721 | NCBI-3-16S@OP727734.1 | Clostridium jeddahense | Bacteria | Bacillota | Clostridia | Eubacteriales | Oscillospiraceae | Faecalispora | Faecalispora jeddahensis |
| Z1 | 259 | 21 | 0.04 | 1898207 | NCBI-3-16S@ON714588.1 | Clostridiales bacterium | Bacteria | Bacillota | Clostridia | Eubacteriales |  |  | Clostridiales bacterium |
| Z1 | 260 | 21 | 0.04 | 2850323 | NCBI-3-16S@NR_181407.1 | Diplocloster agilis | Bacteria | Bacillota | Clostridia | Lachnospirales | Lachnospiraceae | Diplocloster | Diplocloster agilis |
| Z1 | 261 | 20 | 0.03 | 394751 | NCBI-3-16S@OR056046.1 | Pseudoalteromonas arctica | Bacteria | Pseudomonadota | Gammaproteobacteria | Alteromonadales | Pseudoalteromonadaceae | Pseudoalteromonas | Pseudoalteromonas arctica |
| Z1 | 262 | 20 | 0.03 | 163547 | NCBI-3-16S@AF385563.1 | Eubacterium sp. oral clone BU014 | Bacteria | Bacillota | Clostridia | Eubacteriales | Eubacteriaceae | Eubacterium | Eubacterium sp. oral clone BU014 |
| Z1 | 263 | 20 | 0.03 | 2899121 | NCBI-3-16S@NR_179945.1 | Tannockella kyphosi | Bacteria | Bacillota | Erysipelotrichia | Erysipelotrichales | Coprobacillaceae | Tannockella | Tannockella kyphosi |
| Z1 | 264 | 20 | 0.03 | 2763655 | NCBI-3-16S@NR_181386.1 | Gehongia tenuis | Bacteria | Bacillota | Clostridia | Christensenellales | Christensenellaceae | Gehongia | Gehongia tenuis |
| Z1 | 265 | 20 | 0.03 | 270498 | NCBI-3-16S@ON705239.1 | Christensenella hongkongensis | Bacteria | Bacillota | Clostridia | Christensenellales | Christensenellaceae | Christensenella | Christensenella hongkongensis |
| Z1 | 266 | 20 | 0.03 | 676002 | NCBI-3-16S@GQ503878.1 | bacterium enrichment culture clone Ecwsrb041 | Bacteria |  |  |  |  |  | bacterium enrichment culture clone Ecwsrb041 |
| Z1 | 267 | 20 | 0.03 | 437897 | NCBI-3-16S@MN055955.1 | Megamonas funiformis | Bacteria | Bacillota | Negativicutes | Selenomonadales | Selenomonadaceae | Megamonas | Megamonas funiformis |
| Z1 | 268 | 20 | 0.03 | 2758411 | NCBI-3-16S@MT799857.1 | Mailhella sp. Marseille-Q3435 | Bacteria | Thermodesulfobacteriota | Desulfovibrionia | Desulfovibrionales | Desulfovibrionaceae | Mailhella | Mailhella sp. Marseille-Q3435 |
| Z1 | 269 | 20 | 0.03 | 1470347 | NCBI-3-16S@NR_144706.1 | Alistipes ihumii | Bacteria | Bacteroidota | Bacteroidia | Bacteroidales | Rikenellaceae | Alistipes | Alistipes ihumii |
| Z1 | 270 | 20 | 0.03 | 47678 | NCBI-3-16S@OR945919.1 | Bacteroides caccae | Bacteria | Bacteroidota | Bacteroidia | Bacteroidales | Bacteroidaceae | Bacteroides | Bacteroides caccae |
| Z1 | 271 | 20 | 0.03 | 1151620 | NCBI-3-16S@JN713225.1 | Clostridiales bacterium canine oral taxon 061 | Bacteria | Bacillota | Clostridia | Eubacteriales |  |  | Clostridiales bacterium canine oral taxon 061 |
| Z1 | 272 | 20 | 0.03 | 39491 | NCBI-3-16S@ON815083.1 | [Eubacterium] rectale | Bacteria | Bacillota | Clostridia | Lachnospirales | Lachnospiraceae | Agathobacter | Agathobacter rectalis |
| Z1 | 273 | 19 | 0.03 | 1168035 | NCBI-3-16S@OQ348135.1 | Tangfeifania diversioriginum | Bacteria | Bacteroidota | Bacteroidia | Marinilabiliales | Prolixibacteraceae | Tangfeifania | Tangfeifania diversioriginum |
| Z1 | 274 | 19 | 0.03 | 1647705 | NCBI-3-16S@KR232858.1 | Parabacteroides sp. S229 | Bacteria | Bacteroidota | Bacteroidia | Bacteroidales | Tannerellaceae | Parabacteroides | Parabacteroides sp. S229 |
| Z1 | 275 | 19 | 0.03 | 689775 | NCBI-3-16S@GU124473.1 | Bacteroides sp. WA1 | Bacteria | Bacteroidota | Bacteroidia | Bacteroidales | Bacteroidaceae | Bacteroides | Bacteroides sp. WA1 |
| Z1 | 276 | 19 | 0.03 | 2479840 | NCBI-3-16S@NR_179449.1 | Prevotella marseillensis | Bacteria | Bacteroidota | Bacteroidia | Bacteroidales | Prevotellaceae | Prevotella | Prevotella marseillensis |
| Z1 | 277 | 19 | 0.03 | 168384 | NCBI-3-16S@NR_114807.1 | Marvinbryantia formatexigens | Bacteria | Bacillota | Clostridia | Lachnospirales | Lachnospiraceae | Marvinbryantia | Marvinbryantia formatexigens |
| Z1 | 278 | 19 | 0.03 | 89153 | NCBI-3-16S@LC480797.1 | [Clostridium] hylemonae | Bacteria | Bacillota | Clostridia | Lachnospirales | Lachnospiraceae | Lachnoclostridium | [Clostridium] hylemonae |
| Z1 | 279 | 19 | 0.03 | 1622075 | NCBI-3-16S@NR_173694.1 | Olsenella massiliensis | Bacteria | Actinomycetota | Coriobacteriia | Coriobacteriales | Atopobiaceae | Olsenella | Olsenella massiliensis |
| Z1 | 280 | 19 | 0.03 | 1720200 | NCBI-3-16S@NR_147398.1 | Anaerotruncus rubiinfantis | Bacteria | Bacillota | Clostridia | Eubacteriales | Oscillospiraceae | Anaerotruncus | Anaerotruncus rubiinfantis |
| Z1 | 281 | 19 | 0.03 | 1222638 | NCBI-3-16S@JF946843.1 | bacterium enrichment culture clone L35B_101 | Bacteria |  |  |  |  |  | bacterium enrichment culture clone L35B_101 |
| Z1 | 282 | 19 | 0.03 | 1871018 | NCBI-3-16S@NR_179559.1 | Angelakisella massiliensis | Bacteria | Bacillota | Clostridia | Eubacteriales | Oscillospiraceae | Angelakisella | Angelakisella massiliensis |
| Z1 | 283 | 18 | 0.03 | 1816678 | NCBI-3-16S@NR_144743.1 | Christensenella timonensis | Bacteria | Bacillota | Clostridia | Christensenellales | Christensenellaceae | Christensenella | Christensenella timonensis |
| Z1 | 284 | 18 | 0.03 | 29830 | NCBI-3-18S@AY730358.1 | Starmerella apicola | Eukaryota | Ascomycota | Dipodascomycetes | Dipodascales | Trichomonascaceae | Starmerella | Starmerella apicola |
| Z1 | 285 | 18 | 0.03 | 2496531 | NCBI-3-16S@MK287734.1 | Murimonas sp. | Bacteria | Bacillota | Clostridia | Lachnospirales | Lachnospiraceae | Murimonas | Murimonas sp. |
| Z1 | 286 | 18 | 0.03 | 2714355 | NCBI-3-16S@NR_179330.1 | Vescimonas coprocola | Bacteria | Bacillota | Clostridia | Eubacteriales | Oscillospiraceae | Vescimonas | Vescimonas coprocola |
| Z1 | 287 | 18 | 0.03 | 446660 | NCBI-3-16S@OK510307.1 | Adlercreutzia equolifaciens | Bacteria | Actinomycetota | Coriobacteriia | Eggerthellales | Eggerthellaceae | Adlercreutzia | Adlercreutzia equolifaciens |
| Z1 | 288 | 18 | 0.03 | 2033407 | NCBI-3-16S@MH394435.1 | Barnesiella sp. | Bacteria | Bacteroidota | Bacteroidia | Bacteroidales | Barnesiellaceae | Barnesiella | Barnesiella sp. |
| Z1 | 289 | 18 | 0.03 | 1837320 | NCBI-3-16S@KX009918.1 | Lachnospiraceae bacterium DW17 | Bacteria | Bacillota | Clostridia | Lachnospirales | Lachnospiraceae |  | Lachnospiraceae bacterium DW17 |
| Z1 | 290 | 18 | 0.03 | 3046383 | NCBI-3-16S@KF698430.1 | Aristaeella lactis | Bacteria | Bacillota | Clostridia | Eubacteriales | Aristaeellaceae | Aristaeella | Aristaeella lactis |
| Z1 | 291 | 18 | 0.03 | 1796616 | NCBI-3-16S@NR_178840.1 | Blautia pseudococcoides | Bacteria | Bacillota | Clostridia | Lachnospirales | Lachnospiraceae | Blautia | Blautia pseudococcoides |
| Z1 | 292 | 18 | 0.03 | 936073 | NCBI-3-16S@AB596889.1 | Acetivibrio sp. 6-13 | Bacteria | Bacillota | Clostridia | Eubacteriales | Oscillospiraceae | Acetivibrio | Acetivibrio sp. 6-13 |
| Z1 | 293 | 18 | 0.03 | 1841856 | NCBI-3-16S@NR_144744.1 | Bacteroides mediterraneensis | Bacteria | Bacteroidota | Bacteroidia | Bacteroidales | Bacteroidaceae | Bacteroides | Bacteroides mediterraneensis |
| Z1 | 294 | 18 | 0.03 | 187979 | NCBI-3-16S@OR673703.1 | Mitsuokella jalaludinii | Bacteria | Bacillota | Negativicutes | Selenomonadales | Selenomonadaceae | Mitsuokella | Mitsuokella jalaludinii |
| Z1 | 295 | 18 | 0.03 | 1870991 | NCBI-3-16S@NR_179547.1 | Massilioclostridium coli | Bacteria | Bacillota | Clostridia | Eubacteriales | Clostridiaceae | Massilioclostridium | Massilioclostridium coli |
| Z1 | 296 | 18 | 0.03 | 393755 | NCBI-3-16S@DQ676999.1 | iron-reducing enrichment clone Cl-A7 | Bacteria |  |  |  |  |  | iron-reducing enrichment clone Cl-A7 |
| Z1 | 297 | 17 | 0.03 | 487175 | NCBI-3-16S@LT558827.1 | Parasutterella excrementihominis | Bacteria | Pseudomonadota | Betaproteobacteria | Burkholderiales | Sutterellaceae | Parasutterella | Parasutterella excrementihominis |
| Z1 | 298 | 17 | 0.03 | 1151624 | NCBI-3-16S@JN713290.1 | Clostridiales bacterium canine oral taxon 123 | Bacteria | Bacillota | Clostridia | Eubacteriales |  |  | Clostridiales bacterium canine oral taxon 123 |
| Z1 | 299 | 17 | 0.03 | 1720065 | NCBI-3-16S@LN881570.1 | Tepidimicrobium sp. GRM1 | Bacteria | Bacillota | Tissierellia | Tissierellales | Tepidimicrobiaceae | Tepidimicrobium | Tepidimicrobium sp. GRM1 |
| Z1 | 300 | 17 | 0.03 | 1200987 | NCBI-3-16S@HE862234.1 | Clostridium sp. K13-19 | Bacteria | Bacillota | Clostridia | Eubacteriales | Clostridiaceae | Clostridium | Clostridium sp. K13-19 |
| Z1 | 301 | 17 | 0.03 | 1469948 | NCBI-3-16S@NR_156077.1 | Kineothrix alysoides | Bacteria | Bacillota | Clostridia | Lachnospirales | Lachnospiraceae | Kineothrix | Kineothrix alysoides |
| Z1 | 302 | 17 | 0.03 | 1382 | NCBI-3-16S@X67150.1 | Lancefieldella parvula | Bacteria | Actinomycetota | Coriobacteriia | Coriobacteriales | Atopobiaceae | Lancefieldella | Lancefieldella parvula |
| Z1 | 303 | 17 | 0.03 | 1159224 | NCBI-3-16S@AB702938.1 | Clostridiales bacterium CIEAF 022 | Bacteria | Bacillota | Clostridia | Eubacteriales |  |  | Clostridiales bacterium CIEAF 022 |
| Z1 | 304 | 17 | 0.03 | 2564099 | NCBI-3-16S@NR_180520.1 | Ruminococcus bovis | Bacteria | Bacillota | Clostridia | Eubacteriales | Oscillospiraceae | Ruminococcus | Ruminococcus bovis |
| Z1 | 305 | 17 | 0.03 | 265178 | NCBI-3-16S@NR_146687.1 | Breznakia pachnodae | Bacteria | Bacillota | Erysipelotrichia | Erysipelotrichales | Erysipelotrichaceae | Breznakia | Breznakia pachnodae |
| Z1 | 306 | 17 | 0.03 | 1521 | NCBI-3-16S@OK626620.1 | Ruminiclostridium cellulolyticum | Bacteria | Bacillota | Clostridia | Eubacteriales | Oscillospiraceae | Ruminiclostridium | Ruminiclostridium cellulolyticum |
| Z1 | 307 | 17 | 0.03 | 2041843 | NCBI-3-16S@LT934455.1 | Anaeromassilibacillus sp. Marseille-P4683 | Bacteria | Bacillota | Clostridia | Eubacteriales | Acutalibacteraceae | Anaeromassilibacillus | Anaeromassilibacillus sp. Marseille-P4683 |
| Z1 | 308 | 17 | 0.03 | 28112 | NCBI-3-16S@ON222751.1 | Tannerella forsythia | Bacteria | Bacteroidota | Bacteroidia | Bacteroidales | Tannerellaceae | Tannerella | Tannerella forsythia |
| Z1 | 309 | 17 | 0.03 | 1647673 | NCBI-3-16S@KR232914.1 | Barnesiella sp. S496 | Bacteria | Bacteroidota | Bacteroidia | Bacteroidales | Barnesiellaceae | Barnesiella | Barnesiella sp. S496 |
| Z1 | 310 | 17 | 0.03 | 213810 | NCBI-3-16S@NR_102884.1 | Ruminococcus champanellensis 18P13 = JCM 17042 | Bacteria | Bacillota | Clostridia | Eubacteriales | Oscillospiraceae | Ruminococcus | Ruminococcus champanellensis |
| Z1 | 311 | 17 | 0.03 | 1720 | NCBI-3-16S@OR890443.1 | Corynebacterium sp. | Bacteria | Actinomycetota | Actinomycetes | Mycobacteriales | Corynebacteriaceae | Corynebacterium | Corynebacterium sp. |
| Z1 | 312 | 17 | 0.03 | 2290935 | NCBI-3-16S@NR_180156.1 | Parabacteroides acidifaciens | Bacteria | Bacteroidota | Bacteroidia | Bacteroidales | Tannerellaceae | Parabacteroides | Parabacteroides acidifaciens |
| Z1 | 313 | 17 | 0.03 | 2841509 | NCBI-3-16S@NR_181761.1 | Butyricicoccus intestinisimiae | Bacteria | Bacillota | Clostridia | Eubacteriales | Butyricicoccaceae | Butyricicoccus | Butyricicoccus intestinisimiae |
| Z1 | 314 | 17 | 0.03 | 487174 | NCBI-3-16S@NR_113073.1 | Barnesiella intestinihominis | Bacteria | Bacteroidota | Bacteroidia | Bacteroidales | Barnesiellaceae | Barnesiella | Barnesiella intestinihominis |
| Z1 | 315 | 17 | 0.03 | 2518971 | NCBI-3-16S@NR_170508.1 | Duncaniella dubosii | Bacteria | Bacteroidota | Bacteroidia | Bacteroidales | Muribaculaceae | Duncaniella | Duncaniella dubosii |
| Z1 | 316 | 17 | 0.03 | 1211843 | NCBI-3-16S@JX101688.1 | Candidatus Soleaferrea massiliensis AP7 | Bacteria | Bacillota | Clostridia | Eubacteriales |  | Candidatus Soleaferrea | Candidatus Soleaferrea massiliensis |
| Z1 | 317 | 17 | 0.03 | 3046383 | NCBI-3-16S@ON706276.1 | Aristaeella lactis | Bacteria | Bacillota | Clostridia | Eubacteriales | Aristaeellaceae | Aristaeella | Aristaeella lactis |
| Z1 | 318 | 16 | 0.03 | 49283 | NCBI-3-16S@HQ703936.1 | Paenibacillus thiaminolyticus | Bacteria | Bacillota | Bacilli | Bacillales | Paenibacillaceae | Paenibacillus | Paenibacillus thiaminolyticus |
| Z1 | 319 | 16 | 0.03 | 54199 | NCBI-3-18S@JQ698886.1 | Cyniclomyces guttulatus | Eukaryota | Ascomycota | Saccharomycetes | Saccharomycetales | Saccharomycetaceae | Cyniclomyces | Cyniclomyces guttulatus |
| Z1 | 320 | 16 | 0.03 | 2049041 | NCBI-3-16S@MT065897.1 | Slackia sp. | Bacteria | Actinomycetota | Coriobacteriia | Eggerthellales | Eggerthellaceae | Slackia | Slackia sp. |
| Z1 | 321 | 16 | 0.03 | 1742386 | NCBI-3-16S@KR822442.1 | Bacteroides sp. PFB2-14 | Bacteria | Bacteroidota | Bacteroidia | Bacteroidales | Bacteroidaceae | Bacteroides | Bacteroides sp. PFB2-14 |
| Z1 | 322 | 16 | 0.03 | 29323 | NCBI-3-16S@NR_117608.1 | Thermoanaerobacter brockii | Bacteria | Bacillota | Clostridia | Thermoanaerobacterales | Thermoanaerobacteraceae | Thermoanaerobacter | Thermoanaerobacter brockii |
| Z1 | 323 | 16 | 0.03 | 671233 | NCBI-3-16S@GQ422717.1 | Veillonellaceae bacterium oral taxon 129 | Bacteria | Bacillota | Negativicutes | Veillonellales | Veillonellaceae |  | Veillonellaceae bacterium oral taxon 129 |
| Z1 | 324 | 16 | 0.03 | 519017 | NCBI-3-16S@EU592964.1 | Olsenella sp. F0004 | Bacteria | Actinomycetota | Coriobacteriia | Coriobacteriales | Atopobiaceae | Olsenella | Olsenella sp. F0004 |
| Z1 | 325 | 16 | 0.03 | 2764572 | NCBI-3-16S@MT905216.1 | Caproiciproducens sp. NSJ-41 | Bacteria | Bacillota | Clostridia | Eubacteriales | Acutalibacteraceae | Caproiciproducens | Caproiciproducens sp. NSJ-41 |
| Z1 | 326 | 16 | 0.03 | 2687240 | NCBI-3-16S@NR_165703.1 | Zunongwangia flava | Bacteria | Bacteroidota | Flavobacteriia | Flavobacteriales | Flavobacteriaceae | Zunongwangia | Zunongwangia flava |
| Z1 | 327 | 16 | 0.03 | 1964394 | NCBI-3-16S@LT797540.1 | Anaerofilum sp. Marseille-P3374 | Bacteria | Bacillota | Clostridia | Eubacteriales | Oscillospiraceae | Anaerofilum | Anaerofilum sp. Marseille-P3374 |
| Z1 | 328 | 16 | 0.03 | 1577308 | NCBI-3-16S@KM462079.1 | Lachnospiraceae bacterium feline oral taxon 003 | Bacteria | Bacillota | Clostridia | Lachnospirales | Lachnospiraceae |  | Lachnospiraceae bacterium feline oral taxon 003 |
| Z1 | 329 | 16 | 0.03 | 213810 | NCBI-3-16S@NR_114889.1 | Ruminococcus champanellensis 18P13 = JCM 17042 | Bacteria | Bacillota | Clostridia | Eubacteriales | Oscillospiraceae | Ruminococcus | Ruminococcus champanellensis |
| Z1 | 330 | 16 | 0.03 | 1872531 | NCBI-3-16S@MN081629.1 | Anaerotruncus sp. | Bacteria | Bacillota | Clostridia | Eubacteriales | Oscillospiraceae | Anaerotruncus | Anaerotruncus sp. |
| Z1 | 331 | 16 | 0.03 | 1333338 | NCBI-3-16S@KF020721.1 | bacterium enrichment culture clone LDC-11 | Bacteria |  |  |  |  |  | bacterium enrichment culture clone LDC-11 |
| Z1 | 332 | 16 | 0.03 | 691816 | NCBI-3-16S@NR_113072.1 | Bacteroides rodentium | Bacteria | Bacteroidota | Bacteroidia | Bacteroidales | Bacteroidaceae | Bacteroides | Bacteroides rodentium |
| Z1 | 333 | 16 | 0.03 | 745368 | NCBI-3-16S@OK510296.1 | Gemmiger formicilis | Bacteria | Bacillota | Clostridia | Eubacteriales |  | Gemmiger | Gemmiger formicilis |
| Z1 | 334 | 15 | 0.03 | 1030129 | NCBI-3-16S@JF813176.1 | Bacteroides sp. dnLKV7 | Bacteria | Bacteroidota | Bacteroidia | Bacteroidales | Bacteroidaceae | Bacteroides | Bacteroides sp. dnLKV7 |
| Z1 | 335 | 15 | 0.03 | 631044 | NCBI-3-16S@FJ848548.1 | Prevotella sp. 326-8 | Bacteria | Bacteroidota | Bacteroidia | Bacteroidales | Prevotellaceae | Prevotella | Prevotella sp. 326-8 |
| Z1 | 336 | 15 | 0.03 | 35830 | NCBI-3-16S@NR_025917.1 | Acetivibrio cellulolyticus | Bacteria | Bacillota | Clostridia | Eubacteriales | Oscillospiraceae | Acetivibrio | Acetivibrio cellulolyticus |
| Z1 | 337 | 15 | 0.03 | 689779 | NCBI-3-16S@GU124470.1 | Eubacterium sp. SA11 | Bacteria | Bacillota | Clostridia | Eubacteriales | Eubacteriaceae | Eubacterium | Eubacterium sp. SA11 |
| Z1 | 338 | 15 | 0.03 | 626937 | NCBI-3-16S@ON705242.1 | Christensenella minuta | Bacteria | Bacillota | Clostridia | Christensenellales | Christensenellaceae | Christensenella | Christensenella minuta |
| Z1 | 339 | 15 | 0.03 | 1220747 | NCBI-3-16S@JQ771483.1 | bacterium enrichment culture clone E34 | Bacteria |  |  |  |  |  | bacterium enrichment culture clone E34 |
| Z1 | 340 | 15 | 0.03 | 1479757 | NCBI-3-16S@NR_148260.1 | Flavimarina pacifica | Bacteria | Bacteroidota | Flavobacteriia | Flavobacteriales | Flavobacteriaceae | Flavimarina | Flavimarina pacifica |
| Z1 | 341 | 15 | 0.03 | 1007096 | NCBI-3-16S@NR_118156.1 | Oscillibacter ruminantium GH1 | Bacteria | Bacillota | Clostridia | Eubacteriales | Oscillospiraceae | Oscillibacter | Oscillibacter ruminantium |
| Z1 | 342 | 15 | 0.03 | 936069 | NCBI-3-16S@AB596885.1 | Clostridium sp. 6-44 | Bacteria | Bacillota | Clostridia | Eubacteriales | Clostridiaceae | Clostridium | Clostridium sp. 6-44 |
| Z1 | 343 | 15 | 0.03 | 1837337 | NCBI-3-16S@KX009915.1 | Lachnospiraceae bacterium DW8 | Bacteria | Bacillota | Clostridia | Lachnospirales | Lachnospiraceae |  | Lachnospiraceae bacterium DW8 |
| Z1 | 344 | 15 | 0.03 | 2986072 | NCBI-3-16S@OX352005.1 | Candidatus Minimicrobia sp. IHU4 | Bacteria | Candidatus Saccharibacteria |  |  |  | Candidatus Minimicrobia | Candidatus Minimicrobia sp. IHU4 |
| Z1 | 345 | 15 | 0.03 | 1101375 | NCBI-3-16S@JN874874.1 | Clostridium sp. AUH-JLC140 | Bacteria | Bacillota | Clostridia | Eubacteriales | Clostridiaceae | Clostridium | Clostridium sp. AUH-JLC140 |
| Z1 | 346 | 15 | 0.03 | 1506 | NCBI-3-16S@LC515613.1 | Clostridium sp. | Bacteria | Bacillota | Clostridia | Eubacteriales | Clostridiaceae | Clostridium | Clostridium sp. |
| Z1 | 347 | 14 | 0.02 | 1796610 | NCBI-3-16S@NR_144609.1 | Adlercreutzia muris | Bacteria | Actinomycetota | Coriobacteriia | Eggerthellales | Eggerthellaceae | Adlercreutzia | Adlercreutzia muris |
| Z1 | 348 | 14 | 0.02 | 2042683 | NCBI-3-16S@MT323094.2 | Olsenella sp. | Bacteria | Actinomycetota | Coriobacteriia | Coriobacteriales | Atopobiaceae | Olsenella | Olsenella sp. |
| Z1 | 349 | 14 | 0.02 | 1344447 | NCBI-3-16S@KC404041.1 | Ruminococcaceae bacterium enrichment culture clone MRHull-S-07B | Bacteria | Bacillota | Clostridia | Eubacteriales | Oscillospiraceae |  | Ruminococcaceae bacterium enrichment culture clone MRHull-S-07B |
| Z1 | 350 | 14 | 0.02 | 2937417 | NCBI-3-16S@ON361133.1 | Bacteroides muris (ex Fokt et al. 2023) | Bacteria | Bacteroidota | Bacteroidia | Bacteroidales | Bacteroidaceae | Bacteroides | Bacteroides muris (ex Fokt et al. 2023) |
| Z1 | 351 | 14 | 0.02 | 608506 | NCBI-3-16S@NR_117295.1 | Caldicellulosiruptor obsidiansis OB47 | Bacteria | Bacillota |  | Caldicellulosiruptorales | Caldicellulosiruptoraceae | Caldicellulosiruptor | Caldicellulosiruptor obsidiansis |
| Z1 | 352 | 14 | 0.02 | 1349822 | NCBI-3-16S@NR_118316.1 | Coprobacter fastidiosus NSB1 = JCM 33896 | Bacteria | Bacteroidota | Bacteroidia | Bacteroidales | Barnesiellaceae | Coprobacter | Coprobacter fastidiosus |
| Z1 | 353 | 14 | 0.02 | 1776382 | NCBI-3-16S@LN998059.1 | Neglectibacter timonensis | Bacteria | Bacillota | Clostridia | Eubacteriales | Oscillospiraceae | Neglectibacter | Neglectibacter timonensis |
| Z1 | 354 | 14 | 0.02 | 3046382 | NCBI-3-16S@ON706270.1 | Aristaeella hokkaidonensis | Bacteria | Bacillota | Clostridia | Eubacteriales | Aristaeellaceae | Aristaeella | Aristaeella hokkaidonensis |
| Z1 | 355 | 14 | 0.02 | 763034 | NCBI-3-16S@NR_113068.1 | Bacteroides fluxus YIT 12057 | Bacteria | Bacteroidota | Bacteroidia | Bacteroidales | Bacteroidaceae | Bacteroides | Bacteroides fluxus |
| Z1 | 356 | 14 | 0.02 | 1987504 | NCBI-3-16S@LT854615.1 | Butyricimonas sp. Marseille-P3923 | Bacteria | Bacteroidota | Bacteroidia | Bacteroidales | Odoribacteraceae | Butyricimonas | Butyricimonas sp. Marseille-P3923 |
| Z1 | 357 | 14 | 0.02 | 397865 | NCBI-3-16S@NR_121773.2 | Barnesiella viscericola | Bacteria | Bacteroidota | Bacteroidia | Bacteroidales | Barnesiellaceae | Barnesiella | Barnesiella viscericola |
| Z1 | 358 | 14 | 0.02 | 2753162 | NCBI-3-16S@NR_181277.1 | Parabacteroides pekinense | Bacteria | Bacteroidota | Bacteroidia | Bacteroidales | Tannerellaceae | Parabacteroides | Parabacteroides pekinense |
| Z1 | 359 | 13 | 0.02 | 501571 | NCBI-3-16S@NR_044490.1 | Butyricicoccus pullicaecorum | Bacteria | Bacillota | Clostridia | Eubacteriales | Butyricicoccaceae | Butyricicoccus | Butyricicoccus pullicaecorum |
| Z1 | 360 | 13 | 0.02 | 35761 | NCBI-3-16S@MT527593.2 | Nocardioides sp. | Bacteria | Actinomycetota | Actinomycetes | Propionibacteriales | Nocardioidaceae | Nocardioides | Nocardioides sp. |
| Z1 | 361 | 13 | 0.02 | 1217283 | NCBI-3-16S@AB739698.1 | Clostridium sp. 619 | Bacteria | Bacillota | Clostridia | Eubacteriales | Clostridiaceae | Clostridium | Clostridium sp. 619 |
| Z1 | 362 | 13 | 0.02 | 1639600 | NCBI-3-16S@KP980802.1 | Salinimicrobium sp. Bac140 | Bacteria | Bacteroidota | Flavobacteriia | Flavobacteriales | Flavobacteriaceae | Salinimicrobium | Salinimicrobium sp. Bac140 |
| Z1 | 363 | 13 | 0.02 | 1624 | NCBI-3-16S@OR793150.1 | Ligilactobacillus salivarius | Bacteria | Bacillota | Bacilli | Lactobacillales | Lactobacillaceae | Ligilactobacillus | Ligilactobacillus salivarius |
| Z1 | 364 | 13 | 0.02 | 1510 | NCBI-3-16S@NR_114799.1 | Thermoclostridium stercorarium | Bacteria | Bacillota | Clostridia | Eubacteriales | Oscillospiraceae | Thermoclostridium | Thermoclostridium stercorarium |
| Z1 | 365 | 13 | 0.02 | 210830 | NCBI-3-16S@AB093546.1 | Clostridium sp. JC3 | Bacteria | Bacillota | Clostridia | Eubacteriales | Clostridiaceae | Clostridium | Clostridium sp. JC3 |
| Z1 | 366 | 13 | 0.02 | 504444 | NCBI-3-16S@EU432052.1 | Pseudoalteromonas sp. SWI24 | Bacteria | Pseudomonadota | Gammaproteobacteria | Alteromonadales | Pseudoalteromonadaceae | Pseudoalteromonas | Pseudoalteromonas sp. SWI24 |
| Z1 | 367 | 13 | 0.02 | 1220746 | NCBI-3-16S@JQ771482.1 | bacterium enrichment culture clone E33_A | Bacteria |  |  |  |  |  | bacterium enrichment culture clone E33_A |
| Z1 | 368 | 13 | 0.02 | 1852369 | NCBI-3-16S@NR_179531.1 | Marseillibacter massiliensis | Bacteria | Bacillota | Clostridia | Eubacteriales | Oscillospiraceae | Marseillibacter | Marseillibacter massiliensis |
| Z1 | 369 | 13 | 0.02 | 1980694 | NCBI-3-16S@MK287667.1 | Negativibacillus sp. | Bacteria | Bacillota |  |  |  | Negativibacillus | Negativibacillus sp. |
| Z1 | 370 | 13 | 0.02 | 376806 | NCBI-3-16S@NR_041448.1 | Bacteroides gallinarum | Bacteria | Bacteroidota | Bacteroidia | Bacteroidales | Bacteroidaceae | Bacteroides | Bacteroides gallinarum |
| Z1 | 371 | 13 | 0.02 | 880447 | NCBI-3-16S@U26054.1 | Mycoplasma leachii PG50 | Bacteria | Mycoplasmatota | Mollicutes | Mycoplasmatales | Mycoplasmataceae | Mycoplasma | Mycoplasma leachii |
| Z1 | 372 | 13 | 0.02 | 35830 | NCBI-3-16S@KC854859.1 | Acetivibrio cellulolyticus | Bacteria | Bacillota | Clostridia | Eubacteriales | Oscillospiraceae | Acetivibrio | Acetivibrio cellulolyticus |
| Z1 | 373 | 13 | 0.02 | 525256 | NCBI-3-16S@NR_117757.1 | Fannyhessea vaginae DSM 15829 | Bacteria | Actinomycetota | Coriobacteriia | Coriobacteriales | Atopobiaceae | Fannyhessea | Fannyhessea vaginae |
| Z1 | 374 | 13 | 0.02 | 2739389 | NCBI-3-16S@NR_175548.1 | Phocaeicola faecicola | Bacteria | Bacteroidota | Bacteroidia | Bacteroidales | Bacteroidaceae | Phocaeicola | Phocaeicola faecicola |
| Z1 | 375 | 13 | 0.02 | 239 | NCBI-3-16S@OP990481.1 | Flavobacterium sp. | Bacteria | Bacteroidota | Flavobacteriia | Flavobacteriales | Flavobacteriaceae | Flavobacterium | Flavobacterium sp. |
| Z1 | 376 | 13 | 0.02 | 46503 | NCBI-3-16S@NR_041343.1 | Parabacteroides merdae | Bacteria | Bacteroidota | Bacteroidia | Bacteroidales | Tannerellaceae | Parabacteroides | Parabacteroides merdae |
| Z1 | 377 | 13 | 0.02 | 1972642 | NCBI-3-16S@MH456879.2 | Sphaerochaeta sp. | Bacteria | Spirochaetota | Spirochaetia | Spirochaetales | Sphaerochaetaceae | Sphaerochaeta | Sphaerochaeta sp. |
| Z1 | 378 | 13 | 0.02 | 2530391 | NCBI-3-16S@OK626601.1 | Duncaniella freteri | Bacteria | Bacteroidota | Bacteroidia | Bacteroidales | Muribaculaceae | Duncaniella | Duncaniella freteri |
| Z1 | 379 | 12 | 0.02 | 2897707 | NCBI-3-16S@OL617409.1 | Merdimmobilis hominis | Bacteria | Bacillota | Clostridia | Eubacteriales | Oscillospiraceae | Merdimmobilis | Merdimmobilis hominis |
| Z1 | 380 | 12 | 0.02 | 1935934 | NCBI-3-16S@MZ310609.1 | Christensenella sp. | Bacteria | Bacillota | Clostridia | Christensenellales | Christensenellaceae | Christensenella | Christensenella sp. |
| Z1 | 381 | 12 | 0.02 | 41211 | NCBI-3-16S@Y11577.1 | Desulfotomaculum sp. | Bacteria | Bacillota | Clostridia | Eubacteriales | Desulfotomaculaceae | Desulfotomaculum | Desulfotomaculum sp. |
| Z1 | 382 | 12 | 0.02 | 1965325 | NCBI-3-16S@OQ874625.1 | Zunongwangia sp. | Bacteria | Bacteroidota | Flavobacteriia | Flavobacteriales | Flavobacteriaceae | Zunongwangia | Zunongwangia sp. |
| Z1 | 383 | 12 | 0.02 | 45547 | NCBI-3-18S@AB628062.1 | Starmerella etchellsii | Eukaryota | Ascomycota | Dipodascomycetes | Dipodascales | Trichomonascaceae | Starmerella | Starmerella etchellsii |
| Z1 | 384 | 12 | 0.02 | 1161950 | NCBI-3-16S@JQ773354.1 | bacterium enrichment culture clone PKS4 | Bacteria |  |  |  |  |  | bacterium enrichment culture clone PKS4 |
| Z1 | 385 | 12 | 0.02 | 1155409 | NCBI-3-16S@JN688032.1 | bacterium enrichment culture clone M137 | Bacteria |  |  |  |  |  | bacterium enrichment culture clone M137 |
| Z1 | 386 | 12 | 0.02 | 1133056 | NCBI-3-16S@JN585291.1 | Synergistetes bacterium EF1 | Bacteria | Synergistota |  |  |  |  | Synergistetes bacterium EF1 |
| Z1 | 387 | 12 | 0.02 | 150 | NCBI-3-16S@NR_117137.1 | Spirochaeta isovalerica | Bacteria | Spirochaetota | Spirochaetia | Spirochaetales | Spirochaetaceae | Spirochaeta | Spirochaeta isovalerica |
| Z1 | 388 | 12 | 0.02 | 915028 | NCBI-3-16S@HM635213.1 | Acetivibrio sp. enrichment culture clone WSC-3 | Bacteria | Bacillota | Clostridia | Eubacteriales | Oscillospiraceae | Acetivibrio | Acetivibrio sp. enrichment culture clone WSC-3 |
| Z1 | 389 | 12 | 0.02 | 446660 | NCBI-3-16S@NR_121696.1 | Adlercreutzia equolifaciens | Bacteria | Actinomycetota | Coriobacteriia | Eggerthellales | Eggerthellaceae | Adlercreutzia | Adlercreutzia equolifaciens |
| Z1 | 390 | 12 | 0.02 | 630787 | NCBI-3-16S@FJ799122.1 | bacterium enrichment culture clone BA212 | Bacteria |  |  |  |  |  | bacterium enrichment culture clone BA212 |
| Z1 | 391 | 12 | 0.02 | 2841504 | NCBI-3-16S@NR_181757.1 | Dysosmobacter acutus | Bacteria | Bacillota | Clostridia | Eubacteriales | Oscillospiraceae | Dysosmobacter | Dysosmobacter acutus |
| Z1 | 392 | 12 | 0.02 | 93974 | NCBI-3-16S@AF139524.1 | Bacteroides sp. AR20 | Bacteria | Bacteroidota | Bacteroidia | Bacteroidales | Bacteroidaceae | Bacteroides | Bacteroides sp. AR20 |
| Z1 | 393 | 12 | 0.02 | 675978 | NCBI-3-16S@GQ503823.1 | bacterium enrichment culture clone Ecwsrb007 | Bacteria |  |  |  |  |  | bacterium enrichment culture clone Ecwsrb007 |
| Z1 | 394 | 12 | 0.02 | 1394756 | NCBI-3-16S@AB849338.1 | Ruminococcaceae bacterium CG7 | Bacteria | Bacillota | Clostridia | Eubacteriales | Oscillospiraceae |  | Ruminococcaceae bacterium CG7 |
| Z1 | 395 | 12 | 0.02 | 58172 | NCBI-3-16S@ON692827.1 | Paenibacillus sp. | Bacteria | Bacillota | Bacilli | Bacillales | Paenibacillaceae | Paenibacillus | Paenibacillus sp. |
| Z1 | 396 | 12 | 0.02 | 1156029 | NCBI-3-16S@AB700360.1 | Lachnospiraceae bacterium 538 | Bacteria | Bacillota | Clostridia | Lachnospirales | Lachnospiraceae |  | Lachnospiraceae bacterium 538 |
| Z1 | 397 | 12 | 0.02 | 1155397 | NCBI-3-16S@JN688021.1 | bacterium enrichment culture clone M02 | Bacteria |  |  |  |  |  | bacterium enrichment culture clone M02 |
| Z1 | 398 | 12 | 0.02 | 1121430 | NCBI-3-16S@NR_117747.2 | Desulfofundulus thermocisternus DSM 10259 | Bacteria | Bacillota | Clostridia | Eubacteriales | Peptococcaceae | Desulfofundulus | Desulfofundulus thermocisternus |
| Z1 | 399 | 12 | 0.02 | 35830 | NCBI-3-16S@KM036187.1 | Acetivibrio cellulolyticus | Bacteria | Bacillota | Clostridia | Eubacteriales | Oscillospiraceae | Acetivibrio | Acetivibrio cellulolyticus |
| Z1 | 400 | 12 | 0.02 | 1647705 | NCBI-3-16S@KR232858.1 | Parabacteroides sp. S229 | Bacteria | Bacteroidota | Bacteroidia | Bacteroidales | Tannerellaceae | Parabacteroides | Parabacteroides sp. S229 |
| Z1 | 401 | 12 | 0.02 | 604330 | NCBI-3-16S@OR673709.1 | Parafannyhessea umbonata | Bacteria | Actinomycetota | Coriobacteriia | Coriobacteriales | Atopobiaceae | Parafannyhessea | Parafannyhessea umbonata |
| Z1 | 402 | 11 | 0.02 | 57664 | NCBI-3-16S@Y11560.1 | Desulfuromonas thiophila | Bacteria | Thermodesulfobacteriota | Desulfuromonadia | Desulfuromonadales | Desulfuromonadaceae | Desulfuromonas | Desulfuromonas thiophila |
| Z1 | 403 | 11 | 0.02 | 1333339 | NCBI-3-16S@KF020722.1 | bacterium enrichment culture clone LDC-12 | Bacteria |  |  |  |  |  | bacterium enrichment culture clone LDC-12 |
| Z1 | 404 | 11 | 0.02 | 1720195 | NCBI-3-16S@NR_146820.1 | Gabonibacter massiliensis | Bacteria | Bacteroidota | Bacteroidia | Bacteroidales | Porphyromonadaceae | Gabonibacter | Gabonibacter massiliensis |
| Z1 | 405 | 11 | 0.02 | 1394758 | NCBI-3-16S@AB849344.1 | Firmicutes bacterium CA7 | Bacteria | Bacillota |  |  |  |  | Firmicutes bacterium CA7 |
| Z1 | 406 | 11 | 0.02 | 241556 | NCBI-3-16S@AY341819.1 | Bacteroidales genomosp. P1 | Bacteria | Bacteroidota | Bacteroidia | Bacteroidales |  |  | Bacteroidales genomosp. P1 |
| Z1 | 407 | 11 | 0.02 | 1156034 | NCBI-3-16S@AB700365.1 | Lachnospiraceae bacterium 607 | Bacteria | Bacillota | Clostridia | Lachnospirales | Lachnospiraceae |  | Lachnospiraceae bacterium 607 |
| Z1 | 408 | 11 | 0.02 | 411923 | NCBI-3-16S@NR_125518.1 | Moorella perchloratireducens | Bacteria | Bacillota | Clostridia | Moorellales | Moorellaceae | Moorella | Moorella perchloratireducens |
| Z1 | 409 | 11 | 0.02 | 1479757 | NCBI-3-16S@OP343248.1 | Flavimarina pacifica | Bacteria | Bacteroidota | Flavobacteriia | Flavobacteriales | Flavobacteriaceae | Flavimarina | Flavimarina pacifica |
| Z1 | 410 | 11 | 0.02 | 1099853 | NCBI-3-16S@NR_118316.1 | Coprobacter fastidiosus | Bacteria | Bacteroidota | Bacteroidia | Bacteroidales | Barnesiellaceae | Coprobacter | Coprobacter fastidiosus |
| Z1 | 411 | 11 | 0.02 | 253314 | NCBI-3-16S@NR_024829.1 | Acetivibrio straminisolvens | Bacteria | Bacillota | Clostridia | Eubacteriales | Oscillospiraceae | Acetivibrio | Acetivibrio straminisolvens |
| Z1 | 412 | 11 | 0.02 | 2666138 | NCBI-3-16S@OQ134118.1 | Caproicibacterium lactatifermentans | Bacteria | Bacillota | Clostridia | Eubacteriales | Oscillospiraceae | Caproicibacterium | Caproicibacterium lactatifermentans |
| Z1 | 413 | 11 | 0.02 | 78329 | NCBI-3-16S@Y17612.1 | Coenonia anatina | Bacteria | Bacteroidota | Flavobacteriia | Flavobacteriales | Flavobacteriaceae | Allocoenonia | Allocoenonia anatina |
| Z1 | 414 | 11 | 0.02 | 590931 | NCBI-3-16S@AB477432.1 | Clostridiales bacterium SY8526 | Bacteria | Bacillota | Clostridia | Eubacteriales |  |  | Clostridiales bacterium SY8526 |
| Z1 | 415 | 11 | 0.02 | 2897707 | NCBI-3-16S@MT903147.1 | Merdimmobilis hominis | Bacteria | Bacillota | Clostridia | Eubacteriales | Oscillospiraceae | Merdimmobilis | Merdimmobilis hominis |
| Z1 | 416 | 11 | 0.02 | 2773926 | NCBI-3-16S@OP762691.2 | Phocaeicola sp. | Bacteria | Bacteroidota | Bacteroidia | Bacteroidales | Bacteroidaceae | Phocaeicola | Phocaeicola sp. |
| Z1 | 417 | 11 | 0.02 | 861110 | NCBI-3-16S@NR_108994.1 | Mesonia ostreae | Bacteria | Bacteroidota | Flavobacteriia | Flavobacteriales | Flavobacteriaceae | Mesonia | Mesonia ostreae |
| Z1 | 418 | 11 | 0.02 | 696072 | NCBI-3-16S@NR_108707.1 | Cohnella suwonensis | Bacteria | Bacillota | Bacilli | Bacillales | Paenibacillaceae | Cohnella | Cohnella suwonensis |
| Z1 | 419 | 11 | 0.02 | 1188752 | NCBI-3-16S@JQ670706.1 | bacterium enrichment culture clone KSE55-29 | Bacteria |  |  |  |  |  | bacterium enrichment culture clone KSE55-29 |
| Z1 | 420 | 11 | 0.02 | 818 | NCBI-3-16S@MW504858.1 | Bacteroides thetaiotaomicron | Bacteria | Bacteroidota | Bacteroidia | Bacteroidales | Bacteroidaceae | Bacteroides | Bacteroides thetaiotaomicron |
| Z1 | 421 | 11 | 0.02 | 341220 | NCBI-3-16S@NR_043551.1 | Lactonifactor longoviformis | Bacteria | Bacillota | Clostridia | Eubacteriales | Clostridiaceae | Lactonifactor | Lactonifactor longoviformis |
| Z1 | 422 | 11 | 0.02 | 28113 | NCBI-3-16S@KP334251.1 | Bacteroides heparinolyticus | Bacteria | Bacteroidota | Bacteroidia | Bacteroidales | Bacteroidaceae | Bacteroides | Bacteroides heparinolyticus |
| Z1 | 423 | 11 | 0.02 | 697329 | NCBI-3-16S@NR_074399.1 | Ruminococcus albus 7 = DSM 20455 | Bacteria | Bacillota | Clostridia | Eubacteriales | Oscillospiraceae | Ruminococcus | Ruminococcus albus |
| Z1 | 424 | 11 | 0.02 | 930114 | NCBI-3-16S@OK299086.1 | Saliterribacillus persicus | Bacteria | Bacillota | Bacilli | Bacillales | Bacillaceae | Saliterribacillus | Saliterribacillus persicus |
| Z1 | 425 | 11 | 0.02 | 2093856 | NCBI-3-16S@ON705229.1 | Butyricimonas faecalis | Bacteria | Bacteroidota | Bacteroidia | Bacteroidales | Odoribacteraceae | Butyricimonas | Butyricimonas faecalis |
| Z1 | 426 | 11 | 0.02 | 2048138 | NCBI-3-16S@MK287663.1 | Agathobaculum sp. | Bacteria | Bacillota | Clostridia | Eubacteriales | Butyricicoccaceae | Agathobaculum | Agathobaculum sp. |
| Z1 | 427 | 10 | 0.02 | 1837333 | NCBI-3-16S@KX009930.1 | Lachnospiraceae bacterium DW67 | Bacteria | Bacillota | Clostridia | Lachnospirales | Lachnospiraceae |  | Lachnospiraceae bacterium DW67 |
| Z1 | 428 | 10 | 0.02 | 328814 | NCBI-3-16S@NR_113153.1 | Alistipes shahii | Bacteria | Bacteroidota | Bacteroidia | Bacteroidales | Rikenellaceae | Alistipes | Alistipes shahii |
| Z1 | 429 | 10 | 0.02 | 310298 | NCBI-3-16S@AB200225.1 | Phocaeicola coprocola | Bacteria | Bacteroidota | Bacteroidia | Bacteroidales | Bacteroidaceae | Phocaeicola | Phocaeicola coprocola |
| Z1 | 430 | 10 | 0.02 | 1841868 | NCBI-3-16S@NR_179527.1 | Provencibacterium massiliense | Bacteria | Bacillota | Clostridia | Eubacteriales | Oscillospiraceae | Provencibacterium | Provencibacterium massiliense |
| Z1 | 431 | 10 | 0.02 | 371601 | NCBI-3-16S@OR945912.1 | Bacteroides xylanisolvens | Bacteria | Bacteroidota | Bacteroidia | Bacteroidales | Bacteroidaceae | Bacteroides | Bacteroides xylanisolvens |
| Z1 | 432 | 10 | 0.02 | 2608683 | NCBI-3-16S@MN420928.1 | Paenibacillus tepidiphilus | Bacteria | Bacillota | Bacilli | Bacillales | Paenibacillaceae | Paenibacillus | Paenibacillus tepidiphilus |
| Z1 | 433 | 10 | 0.02 | 225198 | NCBI-3-16S@AY187309.1 | Desulfuromusa sp. S1 | Bacteria | Thermodesulfobacteriota | Desulfuromonadia | Desulfuromonadales | Geopsychrobacteraceae | Desulfuromusa | Desulfuromusa sp. S1 |
| Z1 | 434 | 10 | 0.02 | 2024197 | NCBI-3-16S@NR_179635.1 | Bacteroides cutis | Bacteria | Bacteroidota | Bacteroidia | Bacteroidales | Bacteroidaceae | Bacteroides | Bacteroides cutis |
| Z1 | 435 | 10 | 0.02 | 29323 | NCBI-3-16S@NR_027577.1 | Thermoanaerobacter brockii | Bacteria | Bacillota | Clostridia | Thermoanaerobacterales | Thermoanaerobacteraceae | Thermoanaerobacter | Thermoanaerobacter brockii |
| Z1 | 436 | 10 | 0.02 | 1936999 | NCBI-3-16S@LT722679.1 | Lactonifactor sp. Marseille-P3743 | Bacteria | Bacillota | Clostridia | Eubacteriales | Clostridiaceae | Lactonifactor | Lactonifactor sp. Marseille-P3743 |
| Z1 | 437 | 10 | 0.02 | 1958780 | NCBI-3-16S@NR_147400.1 | Clostridium merdae | Bacteria | Bacillota | Clostridia | Eubacteriales | Clostridiaceae | Clostridium | Clostridium merdae |
| Z1 | 438 | 10 | 0.02 | 1469948 | NCBI-3-16S@NR_156081.1 | Kineothrix alysoides | Bacteria | Bacillota | Clostridia | Lachnospirales | Lachnospiraceae | Kineothrix | Kineothrix alysoides |
| Z1 | 439 | 10 | 0.02 | 1287018 | NCBI-3-16S@KC311748.1 | Atopobium sp. S7MSR3 | Bacteria | Actinomycetota | Coriobacteriia | Coriobacteriales | Atopobiaceae | Atopobium | Atopobium sp. S7MSR3 |
| Z1 | 440 | 10 | 0.02 | 2487273 | NCBI-3-16S@NR_180309.1 | Acetivibrio mesophilus | Bacteria | Bacillota | Clostridia | Eubacteriales | Oscillospiraceae | Acetivibrio | Acetivibrio mesophilus |
| Z1 | 441 | 10 | 0.02 | 1577264 | NCBI-3-16S@KM462055.1 | Clostridiales bacterium feline oral taxon 118 | Bacteria | Bacillota | Clostridia | Eubacteriales |  |  | Clostridiales bacterium feline oral taxon 118 |
| Z1 | 442 | 10 | 0.02 | 525256 | NCBI-3-16S@Y17195.1 | Fannyhessea vaginae DSM 15829 | Bacteria | Actinomycetota | Coriobacteriia | Coriobacteriales | Atopobiaceae | Fannyhessea | Fannyhessea vaginae |
| Z1 | 443 | 10 | 0.02 | 2358141 | NCBI-3-16S@NR_165727.1 | Anaerotignum faecicola | Bacteria | Bacillota | Clostridia | Lachnospirales | Anaerotignaceae | Anaerotignum | Anaerotignum faecicola |
| Z1 | 444 | 10 | 0.02 | 1918611 | NCBI-3-16S@MK287701.1 | Muribaculum sp. | Bacteria | Bacteroidota | Bacteroidia | Bacteroidales | Muribaculaceae | Muribaculum | Muribaculum sp. |
| Z1 | 445 | 10 | 0.02 | 1640376 | NCBI-3-16S@KP114244.1 | Ruminococcus sp. FSAA-19 | Bacteria | Bacillota | Clostridia | Eubacteriales | Oscillospiraceae | Ruminococcus | Ruminococcus sp. FSAA-19 |
| Z1 | 446 | 10 | 0.02 | 990 | NCBI-3-16S@NR_117112.1 | Breznakibacter xylanolyticus | Bacteria | Bacteroidota | Bacteroidia | Marinilabiliales | Marinilabiliaceae | Breznakibacter | Breznakibacter xylanolyticus |
| Z1 | 447 | 10 | 0.02 | 2763678 | NCBI-3-16S@MT905149.1 | Youxingia wuxianensis | Bacteria | Bacillota | Clostridia | Eubacteriales | Oscillospiraceae | Youxingia | Youxingia wuxianensis |
| Z1 | 448 | 10 | 0.02 | 575333 | NCBI-3-16S@AB470321.1 | Bacteroides sp. S-18 | Bacteria | Bacteroidota | Bacteroidia | Bacteroidales | Bacteroidaceae | Bacteroides | Bacteroides sp. S-18 |
| Z1 | 449 | 10 | 0.02 | 747601 | NCBI-3-16S@AB551424.1 | Clostridium sp. TG60-1 | Bacteria | Bacillota | Clostridia | Eubacteriales | Clostridiaceae | Clostridium | Clostridium sp. TG60-1 |
| Z1 | 450 | 10 | 0.02 | 1151631 | NCBI-3-16S@JN713380.1 | Clostridiales bacterium canine oral taxon 217 | Bacteria | Bacillota | Clostridia | Eubacteriales |  |  | Clostridiales bacterium canine oral taxon 217 |
| Z1 | 451 | 10 | 0.02 | 1912896 | NCBI-3-16S@MN621536.1 | Bacteroides koreensis | Bacteria | Bacteroidota | Bacteroidia | Bacteroidales | Bacteroidaceae | Bacteroides | Bacteroides koreensis |
| Z1 | 452 | 10 | 0.02 | 2028282 | NCBI-3-16S@MK287758.1 | Lachnoclostridium sp. | Bacteria | Bacillota | Clostridia | Lachnospirales | Lachnospiraceae | Lachnoclostridium | Lachnoclostridium sp. |
| Z1 | 453 | 10 | 0.02 | 1197717 | NCBI-3-16S@NR_109636.1 | Cloacibacillus porcorum | Bacteria | Synergistota | Synergistia | Synergistales | Synergistaceae | Cloacibacillus | Cloacibacillus porcorum |
| Z1 | 454 | 10 | 0.02 | 2719315 | NCBI-3-16S@OP753730.2 | Enterocloster sp. | Bacteria | Bacillota | Clostridia | Lachnospirales | Lachnospiraceae | Enterocloster | Enterocloster sp. |
| Z1 | 455 | 10 | 0.02 | 1168035 | NCBI-3-16S@OQ348135.1 | Tangfeifania diversioriginum | Bacteria | Bacteroidota | Bacteroidia | Marinilabiliales | Prolixibacteraceae | Tangfeifania | Tangfeifania diversioriginum |
| Z1 | 456 | 10 | 0.02 | 1965233 | NCBI-3-16S@MK287741.1 | Odoribacter sp. | Bacteria | Bacteroidota | Bacteroidia | Bacteroidales | Odoribacteraceae | Odoribacter | Odoribacter sp. |
| Z1 | 457 | 10 | 0.02 | 213811 | NCBI-3-16S@AJ515914.1 | Ruminococcus sp. 7L75 | Bacteria | Bacillota | Clostridia | Eubacteriales | Oscillospiraceae | Ruminococcus | Ruminococcus sp. 7L75 |
| Z2 | 1 | 6911 | 16.50 | 1151583 | NCBI-3-16S@JN713550.1 | Spirochaeta sp. canine oral taxon 379 | Bacteria | Spirochaetota | Spirochaetia | Spirochaetales | Spirochaetaceae | Spirochaeta | Spirochaeta sp. canine oral taxon 379 |
| Z2 | 2 | 2130 | 5.09 | 1879010 | NCBI-3-16S@MW741711.1 | Bacillota bacterium | Bacteria | Bacillota |  |  |  |  | Bacillota bacterium |
| Z2 | 3 | 1775 | 4.24 | 1965233 | NCBI-3-16S@MK287741.1 | Odoribacter sp. | Bacteria | Bacteroidota | Bacteroidia | Bacteroidales | Odoribacteraceae | Odoribacter | Odoribacter sp. |
| Z2 | 4 | 1169 | 2.79 | 821 | NCBI-3-16S@MT902980.1 | Phocaeicola vulgatus | Bacteria | Bacteroidota | Bacteroidia | Bacteroidales | Bacteroidaceae | Phocaeicola | Phocaeicola vulgatus |
| Z2 | 5 | 1157 | 2.76 | 820 | NCBI-3-16S@OR945929.1 | Bacteroides uniformis | Bacteria | Bacteroidota | Bacteroidia | Bacteroidales | Bacteroidaceae | Bacteroides | Bacteroides uniformis |
| Z2 | 6 | 825 | 1.97 | 666483 | NCBI-3-16S@GQ377128.1 | bacterium enrichment culture clone DPF25 | Bacteria |  |  |  |  |  | bacterium enrichment culture clone DPF25 |
| Z2 | 7 | 815 | 1.95 | 214819 | NCBI-3-16S@AF550610.1 | Lachnospiraceae bacterium 19gly4 | Bacteria | Bacillota | Clostridia | Lachnospirales | Lachnospiraceae |  | Lachnospiraceae bacterium 19gly4 |
| Z2 | 8 | 729 | 1.74 | 454154 | NCBI-3-16S@NR_113077.1 | Paraprevotella clara | Bacteria | Bacteroidota | Bacteroidia | Bacteroidales | Prevotellaceae | Paraprevotella | Paraprevotella clara |
| Z2 | 9 | 633 | 1.51 | 862466 | NCBI-3-16S@HM231145.1 | Clostridiales bacterium KM2 | Bacteria | Bacillota | Clostridia | Eubacteriales |  |  | Clostridiales bacterium KM2 |
| Z2 | 10 | 541 | 1.29 | 1917882 | NCBI-3-16S@NR_179582.1 | Gabonibacter timonensis | Bacteria | Bacteroidota | Bacteroidia | Bacteroidales | Porphyromonadaceae | Gabonibacter | Gabonibacter timonensis |
| Z2 | 11 | 511 | 1.22 | 28117 | NCBI-3-16S@NR_113152.1 | Alistipes putredinis | Bacteria | Bacteroidota | Bacteroidia | Bacteroidales | Rikenellaceae | Alistipes | Alistipes putredinis |
| Z2 | 12 | 491 | 1.17 | 1981510 | NCBI-3-16S@NR_159227.1 | Monoglobus pectinilyticus | Bacteria | Bacillota | Clostridia | Monoglobales | Monoglobaceae | Monoglobus | Monoglobus pectinilyticus |
| Z2 | 13 | 462 | 1.10 | 1796646 | NCBI-3-16S@OK626632.1 | Muribaculum intestinale | Bacteria | Bacteroidota | Bacteroidia | Bacteroidales | Muribaculaceae | Muribaculum | Muribaculum intestinale |
| Z2 | 14 | 461 | 1.10 | 544645 | NCBI-3-16S@KT288267.1 | Butyricimonas virosa | Bacteria | Bacteroidota | Bacteroidia | Bacteroidales | Odoribacteraceae | Butyricimonas | Butyricimonas virosa |
| Z2 | 15 | 448 | 1.07 | 393755 | NCBI-3-16S@DQ676999.1 | iron-reducing enrichment clone Cl-A7 | Bacteria |  |  |  |  |  | iron-reducing enrichment clone Cl-A7 |
| Z2 | 16 | 438 | 1.05 | 519019 | NCBI-3-16S@EU592966.1 | Atopobium sp. F0209 | Bacteria | Actinomycetota | Coriobacteriia | Coriobacteriales | Atopobiaceae | Atopobium | Atopobium sp. F0209 |
| Z2 | 17 | 394 | 0.94 | 1172158 | NCBI-3-16S@JQ735967.1 | bacterium enrichment culture clone AK10 | Bacteria |  |  |  |  |  | bacterium enrichment culture clone AK10 |
| Z2 | 18 | 377 | 0.90 | 1965293 | NCBI-3-16S@MG321614.1 | Intestinimonas sp. | Bacteria | Bacillota | Clostridia | Eubacteriales |  | Intestinimonas | Intestinimonas sp. |
| Z2 | 19 | 356 | 0.85 | 2564099 | NCBI-3-16S@NR_180520.1 | Ruminococcus bovis | Bacteria | Bacillota | Clostridia | Eubacteriales | Oscillospiraceae | Ruminococcus | Ruminococcus bovis |
| Z2 | 20 | 347 | 0.83 | 2779355 | NCBI-3-16S@NR_180617.1 | Ructibacterium gallinarum | Bacteria | Bacillota | Clostridia | Eubacteriales | Oscillospiraceae | Ructibacterium | Ructibacterium gallinarum |
| Z2 | 21 | 313 | 0.75 | 41978 | NCBI-3-16S@MG859247.1 | Ruminococcus sp. | Bacteria | Bacillota | Clostridia | Eubacteriales | Oscillospiraceae | Ruminococcus | Ruminococcus sp. |
| Z2 | 22 | 306 | 0.73 | 213810 | NCBI-3-16S@NR_102884.1 | Ruminococcus champanellensis 18P13 = JCM 17042 | Bacteria | Bacillota | Clostridia | Eubacteriales | Oscillospiraceae | Ruminococcus | Ruminococcus champanellensis |
| Z2 | 23 | 289 | 0.69 | 1945594 | NCBI-3-16S@MW682303.1 | Sporobacter sp. | Bacteria | Bacillota | Clostridia | Eubacteriales | Oscillospiraceae | Sporobacter | Sporobacter sp. |
| Z2 | 24 | 282 | 0.67 | 1411144 | NCBI-3-16S@NR_145844.1 | Bacteroides caecigallinarum | Bacteria | Bacteroidota | Bacteroidia | Bacteroidales | Bacteroidaceae | Bacteroides | Bacteroides caecigallinarum |
| Z2 | 25 | 281 | 0.67 | 2986072 | NCBI-3-16S@OX352005.1 | Candidatus Minimicrobia sp. IHU4 | Bacteria | Candidatus Saccharibacteria |  |  |  | Candidatus Minimicrobia | Candidatus Minimicrobia sp. IHU4 |
| Z2 | 26 | 234 | 0.56 | 1796610 | NCBI-3-16S@OM658549.1 | Adlercreutzia muris | Bacteria | Actinomycetota | Coriobacteriia | Eggerthellales | Eggerthellaceae | Adlercreutzia | Adlercreutzia muris |
| Z2 | 27 | 234 | 0.56 | 1776381 | NCBI-3-16S@NR_146815.1 | Olegusella massiliensis | Bacteria | Actinomycetota | Coriobacteriia | Coriobacteriales | Coriobacteriaceae | Olegusella | Olegusella massiliensis |
| Z2 | 28 | 224 | 0.53 | 502558 | NCBI-3-16S@AB379693.1 | Eggerthella sp. YY7918 | Bacteria | Actinomycetota | Coriobacteriia | Eggerthellales | Eggerthellaceae | Eggerthella | Eggerthella sp. YY7918 |
| Z2 | 29 | 217 | 0.52 | 3032870 | NCBI-3-16S@MK929052.1 | Lepagella muris | Bacteria | Bacteroidota | Bacteroidia | Bacteroidales | Muribaculaceae | Lepagella | Lepagella muris |
| Z2 | 30 | 211 | 0.50 | 1159221 | NCBI-3-16S@AB702935.1 | Clostridiales bacterium CIEAF 013 | Bacteria | Bacillota | Clostridia | Eubacteriales |  |  | Clostridiales bacterium CIEAF 013 |
| Z2 | 31 | 210 | 0.50 | 2872177 | NCBI-3-16S@OM658611.1 | Odoribacteraceae bacterium | Bacteria | Bacteroidota | Bacteroidia | Bacteroidales | Odoribacteraceae |  | Odoribacteraceae bacterium |
| Z2 | 32 | 194 | 0.46 | 28118 | NCBI-3-16S@MW325948.1 | Odoribacter splanchnicus | Bacteria | Bacteroidota | Bacteroidia | Bacteroidales | Odoribacteraceae | Odoribacter | Odoribacter splanchnicus |
| Z2 | 33 | 185 | 0.44 | 1034346 | NCBI-3-16S@NR_125593.1 | Dielma fastidiosa | Bacteria | Bacillota | Erysipelotrichia | Erysipelotrichales | Erysipelotrichaceae | Dielma | Dielma fastidiosa |
| Z2 | 34 | 184 | 0.44 | 2364796 | NCBI-3-16S@LS999998.1 | Ruminococcus sp. Marseille-P6503 | Bacteria | Bacillota | Clostridia | Eubacteriales | Oscillospiraceae | Ruminococcus | Ruminococcus sp. Marseille-P6503 |
| Z2 | 35 | 184 | 0.44 | 40519 | NCBI-3-16S@X85100.1 | Ruminococcus callidus | Bacteria | Bacillota | Clostridia | Eubacteriales | Oscillospiraceae | Ruminococcus | Ruminococcus callidus |
| Z2 | 36 | 181 | 0.43 | 357276 | NCBI-3-16S@OR125614.1 | Phocaeicola dorei | Bacteria | Bacteroidota | Bacteroidia | Bacteroidales | Bacteroidaceae | Phocaeicola | Phocaeicola dorei |
| Z2 | 37 | 170 | 0.41 | 1131707 | NCBI-3-16S@NR_102964.1 | Sphaerochaeta pleomorpha | Bacteria | Spirochaetota | Spirochaetia | Spirochaetales | Sphaerochaetaceae | Sphaerochaeta | Sphaerochaeta pleomorpha |
| Z2 | 38 | 160 | 0.38 | 1329 | NCBI-3-16S@MT492079.1 | Streptococcus canis | Bacteria | Bacillota | Bacilli | Lactobacillales | Streptococcaceae | Streptococcus | Streptococcus canis |
| Z2 | 39 | 155 | 0.37 | 697329 | NCBI-3-16S@NR_115230.1 | Ruminococcus albus 7 = DSM 20455 | Bacteria | Bacillota | Clostridia | Eubacteriales | Oscillospiraceae | Ruminococcus | Ruminococcus albus |
| Z2 | 40 | 154 | 0.37 | 28111 | NCBI-3-16S@MT902967.1 | Bacteroides eggerthii | Bacteria | Bacteroidota | Bacteroidia | Bacteroidales | Bacteroidaceae | Bacteroides | Bacteroides eggerthii |
| Z2 | 41 | 151 | 0.36 | 1792311 | NCBI-3-16S@NR_178871.1 | Petroclostridium xylanilyticum | Bacteria | Bacillota | Clostridia | Eubacteriales | Oscillospiraceae | Petroclostridium | Petroclostridium xylanilyticum |
| Z2 | 42 | 148 | 0.35 | 1898203 | NCBI-3-16S@MH699361.1 | Lachnospiraceae bacterium | Bacteria | Bacillota | Clostridia | Lachnospirales | Lachnospiraceae |  | Lachnospiraceae bacterium |
| Z2 | 43 | 148 | 0.35 | 1954376 | NCBI-3-16S@MZ310618.1 | Caproiciproducens sp. | Bacteria | Bacillota | Clostridia | Eubacteriales | Acutalibacteraceae | Caproiciproducens | Caproiciproducens sp. |
| Z2 | 44 | 144 | 0.34 | 1870986 | NCBI-3-16S@NR_147375.1 | Colidextribacter massiliensis | Bacteria | Bacillota | Clostridia | Eubacteriales |  | Colidextribacter | Colidextribacter massiliensis |
| Z2 | 45 | 143 | 0.34 | 394503 | NCBI-3-16S@OK626620.1 | Ruminiclostridium cellulolyticum H10 | Bacteria | Bacillota | Clostridia | Eubacteriales | Oscillospiraceae | Ruminiclostridium | Ruminiclostridium cellulolyticum |
| Z2 | 46 | 140 | 0.33 | 411317 | NCBI-3-16S@EF088328.1 | Clostridium islandicum | Bacteria | Bacillota | Clostridia | Eubacteriales | Clostridiaceae | Clostridium | Clostridium islandicum |
| Z2 | 47 | 132 | 0.32 | 1852363 | NCBI-3-16S@LT576387.1 | Clostridiales bacterium Marseille-P2846 | Bacteria | Bacillota | Clostridia | Eubacteriales | Beduinellaceae | Beduinella | Beduinella massiliensis |
| Z2 | 48 | 131 | 0.31 | 55507 | NCBI-3-16S@Y09434.1 | Schwartzia succinivorans | Bacteria | Bacillota | Negativicutes | Selenomonadales | Selenomonadaceae | Schwartzia | Schwartzia succinivorans |
| Z2 | 49 | 127 | 0.30 | 2779352 | NCBI-3-16S@NR_180619.1 | Pseudoflavonifractor gallinarum | Bacteria | Bacillota | Clostridia | Eubacteriales | Oscillospiraceae | Pseudoflavonifractor | Pseudoflavonifractor gallinarum |
| Z2 | 50 | 127 | 0.30 | 2094145 | NCBI-3-16S@NR_179663.1 | Atopobium massiliense | Bacteria | Actinomycetota | Coriobacteriia | Coriobacteriales | Atopobiaceae | Atopobium | Atopobium massiliense |
| Z2 | 51 | 124 | 0.30 | 1872387 | NCBI-3-16S@PP065737.1 | Adlercreutzia sp. | Bacteria | Actinomycetota | Coriobacteriia | Eggerthellales | Eggerthellaceae | Adlercreutzia | Adlercreutzia sp. |
| Z2 | 52 | 122 | 0.29 | 544645 | NCBI-3-16S@NR_041691.1 | Butyricimonas virosa | Bacteria | Bacteroidota | Bacteroidia | Bacteroidales | Odoribacteraceae | Butyricimonas | Butyricimonas virosa |
| Z2 | 53 | 121 | 0.29 | 54199 | NCBI-3-18S@JQ698886.1 | Cyniclomyces guttulatus | Eukaryota | Ascomycota | Saccharomycetes | Saccharomycetales | Saccharomycetaceae | Cyniclomyces | Cyniclomyces guttulatus |
| Z2 | 54 | 118 | 0.28 | 1869337 | NCBI-3-16S@MZ350190.1 | Parabacteroides sp. | Bacteria | Bacteroidota | Bacteroidia | Bacteroidales | Tannerellaceae | Parabacteroides | Parabacteroides sp. |
| Z2 | 55 | 117 | 0.28 | 2894156 | NCBI-3-16S@NR_184626.1 | Leptogranulimonas caecicola | Bacteria | Actinomycetota | Coriobacteriia | Coriobacteriales | Kribbibacteriaceae | Leptogranulimonas | Leptogranulimonas caecicola |
| Z2 | 56 | 116 | 0.28 | 2763676 | NCBI-3-16S@MT905150.1 | Qingrenia yutianensis | Bacteria | Bacillota | Clostridia | Eubacteriales | Oscillospiraceae | Qingrenia | Qingrenia yutianensis |
| Z2 | 57 | 113 | 0.27 | 2939460 | NCBI-3-16S@OP389241.1 | Parvivirga hydrogeniphila | Bacteria | Actinomycetota | Coriobacteriia | Anaerosomatales | Anaerosomataceae | Parvivirga | Parvivirga hydrogeniphila |
| Z2 | 58 | 113 | 0.27 | 1872444 | NCBI-3-16S@MN611110.1 | Alistipes sp. | Bacteria | Bacteroidota | Bacteroidia | Bacteroidales | Rikenellaceae | Alistipes | Alistipes sp. |
| Z2 | 59 | 111 | 0.26 | 1151617 | NCBI-3-16S@JN713189.1 | Clostridiales bacterium canine oral taxon 027 | Bacteria | Bacillota | Clostridia | Eubacteriales |  |  | Clostridiales bacterium canine oral taxon 027 |
| Z2 | 60 | 110 | 0.26 | 1330740 | NCBI-3-16S@KC853480.1 | bacterium enrichment culture clone aHCH2_E12 | Bacteria |  |  |  |  |  | bacterium enrichment culture clone aHCH2_E12 |
| Z2 | 61 | 110 | 0.26 | 1188760 | NCBI-3-16S@JQ670725.1 | bacterium enrichment culture clone KWE55-24 | Bacteria |  |  |  |  |  | bacterium enrichment culture clone KWE55-24 |
| Z2 | 62 | 105 | 0.25 | 84026 | NCBI-3-16S@MT903092.1 | [Clostridium] methylpentosum | Bacteria | Bacillota | Clostridia | Eubacteriales | Oscillospiraceae |  | [Clostridium] methylpentosum |
| Z2 | 63 | 104 | 0.25 | 2164149 | NCBI-3-16S@NR_169458.1 | Tepidibaculum saccharolyticum | Bacteria | Bacillota | Clostridia | Eubacteriales | Oscillospiraceae | Tepidibaculum | Tepidibaculum saccharolyticum |
| Z2 | 64 | 103 | 0.25 | 1870988 | NCBI-3-16S@OK626623.1 | Pseudoflavonifractor phocaeensis | Bacteria | Bacillota | Clostridia | Eubacteriales | Oscillospiraceae | Pseudoflavonifractor | Pseudoflavonifractor phocaeensis |
| Z2 | 65 | 103 | 0.25 | 1980281 | NCBI-3-16S@MN081672.1 | Pseudoflavonifractor sp. | Bacteria | Bacillota | Clostridia | Eubacteriales | Oscillospiraceae | Pseudoflavonifractor | Pseudoflavonifractor sp. |
| Z2 | 66 | 103 | 0.25 | 823 | NCBI-3-16S@OR945889.1 | Parabacteroides distasonis | Bacteria | Bacteroidota | Bacteroidia | Bacteroidales | Tannerellaceae | Parabacteroides | Parabacteroides distasonis |
| Z2 | 67 | 102 | 0.24 | 39490 | NCBI-3-16S@MW398077.1 | Eubacterium ramulus | Bacteria | Bacillota | Clostridia | Eubacteriales | Eubacteriaceae | Eubacterium | Eubacterium ramulus |
| Z2 | 68 | 101 | 0.24 | 641768 | NCBI-3-16S@FJ889653.1 | Ruminococcus sp. ZS2-15 | Bacteria | Bacillota | Clostridia | Eubacteriales | Oscillospiraceae | Ruminococcus | Ruminococcus sp. ZS2-15 |
| Z2 | 69 | 99 | 0.24 | 544645 | NCBI-3-16S@MT902988.1 | Butyricimonas virosa | Bacteria | Bacteroidota | Bacteroidia | Bacteroidales | Odoribacteraceae | Butyricimonas | Butyricimonas virosa |
| Z2 | 70 | 98 | 0.23 | 283734 | NCBI-3-16S@ON497037.1 | Staphylococcus pseudintermedius | Bacteria | Bacillota | Bacilli | Bacillales | Staphylococcaceae | Staphylococcus | Staphylococcus pseudintermedius |
| Z2 | 71 | 98 | 0.23 | 2584469 | NCBI-3-16S@NR_180580.1 | Olsenella lakotia | Bacteria | Actinomycetota | Coriobacteriia | Coriobacteriales | Atopobiaceae | Olsenella | Olsenella lakotia |
| Z2 | 72 | 93 | 0.22 | 1796613 | NCBI-3-16S@OK626630.1 | Bacteroides caecimuris | Bacteria | Bacteroidota | Bacteroidia | Bacteroidales | Bacteroidaceae | Bacteroides | Bacteroides caecimuris |
| Z2 | 73 | 92 | 0.22 | 40518 | NCBI-3-16S@OK510346.1 | Ruminococcus bromii | Bacteria | Bacillota | Clostridia | Eubacteriales | Oscillospiraceae | Ruminococcus | Ruminococcus bromii |
| Z2 | 74 | 91 | 0.22 | 2763659 | NCBI-3-16S@NR_181390.1 | Fumia xinanensis | Bacteria | Bacillota | Clostridia | Eubacteriales | Oscillospiraceae | Fumia | Fumia xinanensis |
| Z2 | 75 | 90 | 0.21 | 666493 | NCBI-3-16S@GQ377117.1 | bacterium enrichment culture clone DPHB07 | Bacteria |  |  |  |  |  | bacterium enrichment culture clone DPHB07 |
| Z2 | 76 | 88 | 0.21 | 2606626 | NCBI-3-16S@NR_180830.1 | Sodaliphilus pleomorphus | Bacteria | Bacteroidota | Bacteroidia | Bacteroidales | Muribaculaceae | Sodaliphilus | Sodaliphilus pleomorphus |
| Z2 | 77 | 87 | 0.21 | 747645 | NCBI-3-16S@NR_117374.1 | Parvibacter caecicola | Bacteria | Actinomycetota | Coriobacteriia | Coriobacteriales | Coriobacteriaceae | Parvibacter | Parvibacter caecicola |
| Z2 | 78 | 86 | 0.21 | 2030927 | NCBI-3-16S@MK929082.1 | Bacteroidales bacterium | Bacteria | Bacteroidota | Bacteroidia | Bacteroidales |  |  | Bacteroidales bacterium |
| Z2 | 79 | 84 | 0.20 | 29523 | NCBI-3-16S@AM117579.1 | Bacteroides sp. | Bacteria | Bacteroidota | Bacteroidia | Bacteroidales | Bacteroidaceae | Bacteroides | Bacteroides sp. |
| Z2 | 80 | 83 | 0.20 | 1929886 | NCBI-3-16S@MK287687.1 | Eggerthella sp. | Bacteria | Actinomycetota | Coriobacteriia | Eggerthellales | Eggerthellaceae | Eggerthella | Eggerthella sp. |
| Z2 | 81 | 83 | 0.20 | 1870985 | NCBI-3-16S@NR_179544.1 | Arabiibacter massiliensis | Bacteria | Actinomycetota | Coriobacteriia | Eggerthellales | Eggerthellaceae | Arabiibacter | Arabiibacter massiliensis |
| Z2 | 82 | 83 | 0.20 | 2086579 | NCBI-3-16S@LT985388.1 | Bacteroides sp. Marseille-P3684 | Bacteria | Bacteroidota | Bacteroidia | Bacteroidales | Bacteroidaceae | Bacteroides | Bacteroides sp. Marseille-P3684 |
| Z2 | 83 | 79 | 0.19 | 1297424 | NCBI-3-16S@NR_125464.1 | Anaerobacterium chartisolvens | Bacteria | Bacillota | Clostridia | Eubacteriales | Oscillospiraceae | Anaerobacterium | Anaerobacterium chartisolvens |
| Z2 | 84 | 79 | 0.19 | 1969738 | NCBI-3-16S@LC259309.1 | Butyricimonas sp. | Bacteria | Bacteroidota | Bacteroidia | Bacteroidales | Odoribacteraceae | Butyricimonas | Butyricimonas sp. |
| Z2 | 85 | 78 | 0.19 | 2485925 | NCBI-3-16S@MN913813.1 | Oscillospiraceae bacterium | Bacteria | Bacillota | Clostridia | Eubacteriales | Oscillospiraceae |  | Oscillospiraceae bacterium |
| Z2 | 86 | 76 | 0.18 | 2981770 | NCBI-3-16S@OK510325.1 | Gallintestinimicrobium propionicum | Bacteria | Bacillota | Clostridia | Lachnospirales | Lachnospiraceae | Gallintestinimicrobium | Gallintestinimicrobium propionicum |
| Z2 | 87 | 73 | 0.17 | 258132 | NCBI-3-16S@AY466715.1 | Clostridiales bacterium NS5-2 | Bacteria | Bacillota | Clostridia | Eubacteriales |  |  | Clostridiales bacterium NS5-2 |
| Z2 | 88 | 71 | 0.17 | 671267 | NCBI-3-16S@NR_113195.1 | Phocaeicola sartorii | Bacteria | Bacteroidota | Bacteroidia | Bacteroidales | Bacteroidaceae | Phocaeicola | Phocaeicola sartorii |
| Z2 | 89 | 71 | 0.17 | 371601 | NCBI-3-16S@OR945801.1 | Bacteroides xylanisolvens | Bacteria | Bacteroidota | Bacteroidia | Bacteroidales | Bacteroidaceae | Bacteroides | Bacteroides xylanisolvens |
| Z2 | 90 | 70 | 0.17 | 9906 | NCBI-3-18S@XR_009732643.1 | Bos javanicus | Eukaryota | Chordata | Mammalia | Artiodactyla | Bovidae | Bos | Bos javanicus |
| Z2 | 91 | 70 | 0.17 | 2763052 | NCBI-3-16S@MT905207.1 | Lawsonibacter faecis | Bacteria | Bacillota | Clostridia | Eubacteriales | Oscillospiraceae | Lawsonibacter | Lawsonibacter faecis |
| Z2 | 92 | 68 | 0.16 | 537375 | NCBI-3-16S@EU728793.1 | Clostridiaceae bacterium DJF_VR76 | Bacteria | Bacillota | Clostridia | Eubacteriales | Clostridiaceae |  | Clostridiaceae bacterium DJF_VR76 |
| Z2 | 93 | 68 | 0.16 | 1030129 | NCBI-3-16S@JF813176.1 | Bacteroides sp. dnLKV7 | Bacteria | Bacteroidota | Bacteroidia | Bacteroidales | Bacteroidaceae | Bacteroides | Bacteroides sp. dnLKV7 |
| Z2 | 94 | 67 | 0.16 | 246199 | NCBI-3-16S@AY445594.1 | Ruminococcus albus 8 | Bacteria | Bacillota | Clostridia | Eubacteriales | Oscillospiraceae | Ruminococcus | Ruminococcus albus |
| Z2 | 95 | 66 | 0.16 | 2834112 | NCBI-3-16S@NR_181731.1 | Bacteroides propionicigenes | Bacteria | Bacteroidota | Bacteroidia | Bacteroidales | Bacteroidaceae | Bacteroides | Bacteroides propionicigenes |
| Z2 | 96 | 66 | 0.16 | 28113 | NCBI-3-16S@JN713478.1 | Bacteroides heparinolyticus | Bacteria | Bacteroidota | Bacteroidia | Bacteroidales | Bacteroidaceae | Bacteroides | Bacteroides heparinolyticus |
| Z2 | 97 | 66 | 0.16 | 1972561 | NCBI-3-16S@OM368626.1 | Eggerthellaceae bacterium | Bacteria | Actinomycetota | Coriobacteriia | Eggerthellales | Eggerthellaceae |  | Eggerthellaceae bacterium |
| Z2 | 98 | 65 | 0.16 | 1926877 | NCBI-3-16S@MG551268.2 | Proteiniphilum sp. | Bacteria | Bacteroidota | Bacteroidia | Bacteroidales | Dysgonomonadaceae | Proteiniphilum | Proteiniphilum sp. |
| Z2 | 99 | 65 | 0.16 | 2049040 | NCBI-3-16S@MK287624.1 | Roseburia sp. | Bacteria | Bacillota | Clostridia | Lachnospirales | Lachnospiraceae | Roseburia | Roseburia sp. |
| Z2 | 100 | 64 | 0.15 | 569483 | NCBI-3-16S@FJ269072.1 | iron-reducing bacterium enrichment culture clone HN109 | Bacteria |  |  |  |  |  | iron-reducing bacterium enrichment culture clone HN109 |
| Z2 | 101 | 62 | 0.15 | 1945593 | NCBI-3-16S@MZ310617.1 | Oscillibacter sp. | Bacteria | Bacillota | Clostridia | Eubacteriales | Oscillospiraceae | Oscillibacter | Oscillibacter sp. |
| Z2 | 102 | 62 | 0.15 | 1155412 | NCBI-3-16S@JN688035.1 | bacterium enrichment culture clone M153 | Bacteria |  |  |  |  |  | bacterium enrichment culture clone M153 |
| Z2 | 103 | 61 | 0.15 | 1852385 | NCBI-3-16S@NR_173693.1 | Olsenella phocaeensis | Bacteria | Actinomycetota | Coriobacteriia | Coriobacteriales | Atopobiaceae | Olsenella | Olsenella phocaeensis |
| Z2 | 104 | 61 | 0.15 | 1295 | NCBI-3-16S@MF678906.1 | Staphylococcus schleiferi | Bacteria | Bacillota | Bacilli | Bacillales | Staphylococcaceae | Staphylococcus | Staphylococcus schleiferi |
| Z2 | 105 | 60 | 0.14 | 1197717 | NCBI-3-16S@MN537495.1 | Cloacibacillus porcorum | Bacteria | Synergistota | Synergistia | Synergistales | Synergistaceae | Cloacibacillus | Cloacibacillus porcorum |
| Z2 | 106 | 60 | 0.14 | 1159223 | NCBI-3-16S@AB702937.1 | Clostridiales bacterium CIEAF 021 | Bacteria | Bacillota | Clostridia | Eubacteriales |  |  | Clostridiales bacterium CIEAF 021 |
| Z2 | 107 | 59 | 0.14 | 1588753 | NCBI-3-16S@KP192306.1 | Coriobacteriales bacterium DNF00809 | Bacteria | Actinomycetota | Coriobacteriia | Coriobacteriales |  |  | Coriobacteriales bacterium DNF00809 |
| Z2 | 108 | 59 | 0.14 | 2763654 | NCBI-3-16S@NR_181383.1 | Luoshenia tenuis | Bacteria | Bacillota | Clostridia | Christensenellales | Christensenellaceae | Luoshenia | Luoshenia tenuis |
| Z2 | 109 | 52 | 0.12 | 1918636 | NCBI-3-16S@MN081649.1 | Acutalibacter sp. | Bacteria | Bacillota | Clostridia | Eubacteriales | Acutalibacteraceae | Acutalibacter | Acutalibacter sp. |
| Z2 | 110 | 52 | 0.12 | 1556 | NCBI-3-16S@NR_117601.1 | Gottschalkia acidurici | Bacteria | Bacillota | Tissierellia | Tissierellales | Gottschalkiaceae | Gottschalkia | Gottschalkia acidurici |
| Z2 | 111 | 51 | 0.12 | 712955 | NCBI-3-16S@HM099644.1 | Clostridiales bacterium oral taxon F32 | Bacteria | Bacillota | Clostridia | Eubacteriales |  |  | Clostridiales bacterium oral taxon F32 |
| Z2 | 112 | 50 | 0.12 | 915171 | NCBI-3-16S@HQ452853.1 | Clostridiales bacterium 30-4c | Bacteria | Bacillota | Clostridia | Eubacteriales |  |  | Clostridiales bacterium 30-4c |
| Z2 | 113 | 49 | 0.12 | 2585119 | NCBI-3-16S@NR_179296.1 | Alistipes dispar | Bacteria | Bacteroidota | Bacteroidia | Bacteroidales | Rikenellaceae | Alistipes | Alistipes dispar |
| Z2 | 114 | 48 | 0.11 | 1972642 | NCBI-3-16S@MW599793.1 | Sphaerochaeta sp. | Bacteria | Spirochaetota | Spirochaetia | Spirochaetales | Sphaerochaetaceae | Sphaerochaeta | Sphaerochaeta sp. |
| Z2 | 115 | 48 | 0.11 | 1515 | NCBI-3-16S@NR_113157.1 | Acetivibrio thermocellus | Bacteria | Bacillota | Clostridia | Eubacteriales | Oscillospiraceae | Acetivibrio | Acetivibrio thermocellus |
| Z2 | 116 | 47 | 0.11 | 292800 | NCBI-3-16S@MN055958.1 | Flavonifractor plautii | Bacteria | Bacillota | Clostridia | Eubacteriales | Oscillospiraceae | Flavonifractor | Flavonifractor plautii |
| Z2 | 117 | 47 | 0.11 | 93974 | NCBI-3-16S@AF139524.1 | Bacteroides sp. AR20 | Bacteria | Bacteroidota | Bacteroidia | Bacteroidales | Bacteroidaceae | Bacteroides | Bacteroides sp. AR20 |
| Z2 | 118 | 47 | 0.11 | 376804 | NCBI-3-16S@NR_041446.1 | Phocaeicola barnesiae | Bacteria | Bacteroidota | Bacteroidia | Bacteroidales | Bacteroidaceae | Phocaeicola | Phocaeicola barnesiae |
| Z2 | 119 | 47 | 0.11 | 1236512 | NCBI-3-16S@NR_113072.1 | Bacteroides rodentium JCM 16496 | Bacteria | Bacteroidota | Bacteroidia | Bacteroidales | Bacteroidaceae | Bacteroides | Bacteroides rodentium |
| Z2 | 120 | 46 | 0.11 | 1288121 | NCBI-3-16S@NR_118219.1 | Alistipes senegalensis | Bacteria | Bacteroidota | Bacteroidia | Bacteroidales | Rikenellaceae | Alistipes | Alistipes senegalensis |
| Z2 | 121 | 46 | 0.11 | 915170 | NCBI-3-16S@HQ452852.1 | Clostridiales bacterium 24-4c | Bacteria | Bacillota | Clostridia | Eubacteriales |  |  | Clostridiales bacterium 24-4c |
| Z2 | 122 | 46 | 0.11 | 454155 | NCBI-3-16S@NR_113078.1 | Paraprevotella xylaniphila | Bacteria | Bacteroidota | Bacteroidia | Bacteroidales | Prevotellaceae | Paraprevotella | Paraprevotella xylaniphila |
| Z2 | 123 | 45 | 0.11 | 2137881 | NCBI-3-16S@MK287767.1 | Coprobacillus sp. | Bacteria | Bacillota | Erysipelotrichia | Erysipelotrichales | Coprobacillaceae | Coprobacillus | Coprobacillus sp. |
| Z2 | 124 | 45 | 0.11 | 742727 | NCBI-3-16S@NR_113070.1 | Bacteroides oleiciplenus YIT 12058 | Bacteria | Bacteroidota | Bacteroidia | Bacteroidales | Bacteroidaceae | Bacteroides | Bacteroides oleiciplenus |
| Z2 | 125 | 45 | 0.11 | 1871023 | NCBI-3-16S@NR_147368.1 | Millionella massiliensis | Bacteria | Bacteroidota | Bacteroidia | Bacteroidales | Rikenellaceae | Millionella | Millionella massiliensis |
| Z2 | 126 | 44 | 0.11 | 1871015 | NCBI-3-16S@LT598575.1 | Pseudoflavonifractor sp. Marseille-P3106 | Bacteria | Bacillota | Clostridia | Eubacteriales | Oscillospiraceae | Pseudoflavonifractor | Pseudoflavonifractor sp. Marseille-P3106 |
| Z2 | 127 | 43 | 0.10 | 2608714 | NCBI-3-16S@MN428134.1 | Geminicoccaceae bacterium CFH 77886 | Bacteria | Pseudomonadota | Alphaproteobacteria | Geminicoccales | Geminicoccaceae |  | Geminicoccaceae bacterium CFH 77886 |
| Z2 | 128 | 43 | 0.10 | 649764 | NCBI-3-16S@NR_024952.1 | Slackia exigua ATCC 700122 | Bacteria | Actinomycetota | Coriobacteriia | Eggerthellales | Eggerthellaceae | Slackia | Slackia exigua |
| Z2 | 129 | 42 | 0.10 | 925962 | NCBI-3-16S@AB599946.1 | Bacteroides sp. SLC1-38 | Bacteria | Bacteroidota | Bacteroidia | Bacteroidales | Bacteroidaceae | Bacteroides | Bacteroides sp. SLC1-38 |
| Z2 | 130 | 42 | 0.10 | 1647716 | NCBI-3-16S@KR232852.1 | Porphyromonadaceae bacterium S190 | Bacteria | Bacteroidota | Bacteroidia | Bacteroidales | Porphyromonadaceae |  | Porphyromonadaceae bacterium S190 |
| Z2 | 131 | 41 | 0.10 | 569511 | NCBI-3-16S@FJ269048.1 | iron-reducing bacterium enrichment culture clone HN7 | Bacteria |  |  |  |  |  | iron-reducing bacterium enrichment culture clone HN7 |
| Z2 | 132 | 41 | 0.10 | 2086583 | NCBI-3-16S@NR_179661.1 | Pseudoruminococcus massiliensis | Bacteria | Bacillota | Clostridia | Eubacteriales | Acutalibacteraceae | Pseudoruminococcus | Pseudoruminococcus massiliensis |
| Z2 | 133 | 41 | 0.10 | 626930 | NCBI-3-16S@MW558146.1 | Bacteroides fluxus | Bacteria | Bacteroidota | Bacteroidia | Bacteroidales | Bacteroidaceae | Bacteroides | Bacteroides fluxus |
| Z2 | 134 | 41 | 0.10 | 1030127 | NCBI-3-16S@JF813174.1 | Bacteroides sp. dnLKV2 | Bacteria | Bacteroidota | Bacteroidia | Bacteroidales | Bacteroidaceae | Bacteroides | Bacteroides sp. dnLKV2 |
| Z2 | 135 | 40 | 0.10 | 1872444 | NCBI-3-16S@MN611110.1 | Alistipes sp. | Bacteria | Bacteroidota | Bacteroidia | Bacteroidales | Rikenellaceae | Alistipes | Alistipes sp. |
| Z2 | 136 | 40 | 0.10 | 1335613 | NCBI-3-16S@MK544835.1 | Gordonibacter urolithinfaciens | Bacteria | Actinomycetota | Coriobacteriia | Eggerthellales | Eggerthellaceae | Gordonibacter | Gordonibacter urolithinfaciens |
| Z2 | 137 | 40 | 0.10 | 142586 | NCBI-3-16S@OK272455.1 | Eubacterium sp. | Bacteria | Bacillota | Clostridia | Eubacteriales | Eubacteriaceae | Eubacterium | Eubacterium sp. |
| Z2 | 138 | 40 | 0.10 | 1903262 | NCBI-3-16S@NR_179564.1 | Bacteroides ndongoniae | Bacteria | Bacteroidota | Bacteroidia | Bacteroidales | Bacteroidaceae | Bacteroides | Bacteroides ndongoniae |
| Z2 | 139 | 40 | 0.10 | 2841509 | NCBI-3-16S@NR_181761.1 | Butyricicoccus intestinisimiae | Bacteria | Bacillota | Clostridia | Eubacteriales | Butyricicoccaceae | Butyricicoccus | Butyricicoccus intestinisimiae |
| Z2 | 140 | 39 | 0.09 | 2585118 | NCBI-3-16S@ON705225.1 | Alistipes communis | Bacteria | Bacteroidota | Bacteroidia | Bacteroidales | Rikenellaceae | Alistipes | Alistipes communis |
| Z2 | 141 | 39 | 0.09 | 2751153 | NCBI-3-16S@NR_173687.1 | Coprobacter secundus subsp. similis | Bacteria | Bacteroidota | Bacteroidia | Bacteroidales | Barnesiellaceae | Coprobacter | Coprobacter secundus |
| Z2 | 142 | 38 | 0.09 | 393757 | NCBI-3-16S@DQ677001.1 | iron-reducing enrichment clone Cl-A9 | Bacteria |  |  |  |  |  | iron-reducing enrichment clone Cl-A9 |
| Z2 | 143 | 38 | 0.09 | 915176 | NCBI-3-16S@HQ452851.1 | Clostridiales bacterium 53-4c | Bacteria | Bacillota | Clostridia | Eubacteriales |  |  | Clostridiales bacterium 53-4c |
| Z2 | 144 | 38 | 0.09 | 545496 | NCBI-3-16S@EU815223.1 | Ruminococcus sp. NML 00-0124 | Bacteria | Bacillota | Clostridia | Eubacteriales | Oscillospiraceae | Ruminococcus | Ruminococcus sp. NML 00-0124 |
| Z2 | 145 | 37 | 0.09 | 1156034 | NCBI-3-16S@AB700365.1 | Lachnospiraceae bacterium 607 | Bacteria | Bacillota | Clostridia | Lachnospirales | Lachnospiraceae |  | Lachnospiraceae bacterium 607 |
| Z2 | 146 | 37 | 0.09 | 2707299 | NCBI-3-16S@NR_179369.1 | Adlercreutzia hattorii | Bacteria | Actinomycetota | Coriobacteriia | Eggerthellales | Eggerthellaceae | Adlercreutzia | Adlercreutzia hattorii |
| Z2 | 147 | 37 | 0.09 | 915028 | NCBI-3-16S@HM635213.1 | Acetivibrio sp. enrichment culture clone WSC-3 | Bacteria | Bacillota | Clostridia | Eubacteriales | Oscillospiraceae | Acetivibrio | Acetivibrio sp. enrichment culture clone WSC-3 |
| Z2 | 148 | 37 | 0.09 | 747602 | NCBI-3-16S@AB551425.1 | Clostridium sp. TG60-81 | Bacteria | Bacillota | Clostridia | Eubacteriales | Clostridiaceae | Clostridium | Clostridium sp. TG60-81 |
| Z2 | 149 | 37 | 0.09 | 2485926 | NCBI-3-16S@OM658553.1 | Atopobiaceae bacterium | Bacteria | Actinomycetota | Coriobacteriia | Coriobacteriales | Atopobiaceae |  | Atopobiaceae bacterium |
| Z2 | 150 | 36 | 0.09 | 1650661 | NCBI-3-16S@NR_179410.1 | Clostridium phoceensis | Bacteria | Bacillota | Clostridia | Eubacteriales | Clostridiaceae | Clostridium | Clostridium phoceensis |
| Z2 | 151 | 36 | 0.09 | 320502 | NCBI-3-16S@OK626616.1 | Acetivibrio alkalicellulosi | Bacteria | Bacillota | Clostridia | Eubacteriales | Oscillospiraceae | Acetivibrio | Acetivibrio alkalicellulosi |
| Z2 | 152 | 35 | 0.08 | 671233 | NCBI-3-16S@GQ422717.1 | Veillonellaceae bacterium oral taxon 129 | Bacteria | Bacillota | Negativicutes | Veillonellales | Veillonellaceae |  | Veillonellaceae bacterium oral taxon 129 |
| Z2 | 153 | 35 | 0.08 | 1033732 | NCBI-3-16S@NR_118219.1 | Alistipes senegalensis JC50 | Bacteria | Bacteroidota | Bacteroidia | Bacteroidales | Rikenellaceae | Alistipes | Alistipes senegalensis |
| Z2 | 154 | 34 | 0.08 | 1702285 | NCBI-3-16S@NR_179426.1 | Intestinimonas gabonensis | Bacteria | Bacillota | Clostridia | Eubacteriales |  | Intestinimonas | Intestinimonas gabonensis |
| Z2 | 155 | 34 | 0.08 | 818 | NCBI-3-16S@OR945867.1 | Bacteroides thetaiotaomicron | Bacteria | Bacteroidota | Bacteroidia | Bacteroidales | Bacteroidaceae | Bacteroides | Bacteroides thetaiotaomicron |
| Z2 | 156 | 34 | 0.08 | 1647718 | NCBI-3-16S@KR232873.1 | Propionibacterium sp. S342 | Bacteria | Actinomycetota | Actinomycetes | Propionibacteriales | Propionibacteriaceae | Propionibacterium | Propionibacterium sp. S342 |
| Z2 | 157 | 33 | 0.08 | 575333 | NCBI-3-16S@AB470321.1 | Bacteroides sp. S-18 | Bacteria | Bacteroidota | Bacteroidia | Bacteroidales | Bacteroidaceae | Bacteroides | Bacteroides sp. S-18 |
| Z2 | 158 | 33 | 0.08 | 246787 | NCBI-3-16S@OP690569.1 | Bacteroides cellulosilyticus | Bacteria | Bacteroidota | Bacteroidia | Bacteroidales | Bacteroidaceae | Bacteroides | Bacteroides cellulosilyticus |
| Z2 | 159 | 33 | 0.08 | 341220 | NCBI-3-16S@NR_043551.1 | Lactonifactor longoviformis | Bacteria | Bacillota | Clostridia | Eubacteriales | Clostridiaceae | Lactonifactor | Lactonifactor longoviformis |
| Z2 | 160 | 32 | 0.08 | 1872092 | NCBI-3-16S@MZ310614.1 | Acetivibrio sp. | Bacteria | Bacillota | Clostridia | Eubacteriales | Oscillospiraceae | Acetivibrio | Acetivibrio sp. |
| Z2 | 161 | 32 | 0.08 | 2897707 | NCBI-3-16S@NR_144748.1 | Merdimmobilis hominis | Bacteria | Bacillota | Clostridia | Eubacteriales | Oscillospiraceae | Merdimmobilis | Merdimmobilis hominis |
| Z2 | 162 | 32 | 0.08 | 265178 | NCBI-3-16S@NR_146687.1 | Breznakia pachnodae | Bacteria | Bacillota | Erysipelotrichia | Erysipelotrichales | Erysipelotrichaceae | Breznakia | Breznakia pachnodae |
| Z2 | 163 | 32 | 0.08 | 1352374 | NCBI-3-16S@KF156793.1 | Ruminococcus sp. YE78 | Bacteria | Bacillota | Clostridia | Eubacteriales | Oscillospiraceae | Ruminococcus | Ruminococcus sp. YE78 |
| Z2 | 164 | 30 | 0.07 | 630799 | NCBI-3-16S@FJ799136.1 | bacterium enrichment culture clone EtOH-23 | Bacteria |  |  |  |  |  | bacterium enrichment culture clone EtOH-23 |
| Z2 | 165 | 30 | 0.07 | 1841857 | NCBI-3-16S@NR_144745.1 | Culturomica massiliensis | Bacteria | Bacteroidota | Bacteroidia | Bacteroidales | Odoribacteraceae | Culturomica | Culturomica massiliensis |
| Z2 | 166 | 30 | 0.07 | 1841865 | NCBI-3-16S@NR_144747.1 | Mediterranea massiliensis | Bacteria | Bacteroidota | Bacteroidia | Bacteroidales | Bacteroidaceae | Mediterranea | Mediterranea massiliensis |
| Z2 | 167 | 30 | 0.07 | 1516126 | NCBI-3-16S@NR_178264.1 | Dysgonomonas termitidis | Bacteria | Bacteroidota | Bacteroidia | Bacteroidales | Dysgonomonadaceae | Dysgonomonas | Dysgonomonas termitidis |
| Z2 | 168 | 30 | 0.07 | 47678 | NCBI-3-16S@OR945817.1 | Bacteroides caccae | Bacteria | Bacteroidota | Bacteroidia | Bacteroidales | Bacteroidaceae | Bacteroides | Bacteroides caccae |
| Z2 | 169 | 30 | 0.07 | 720554 | NCBI-3-16S@NR_102987.1 | Acetivibrio clariflavus DSM 19732 | Bacteria | Bacillota | Clostridia | Eubacteriales | Oscillospiraceae | Acetivibrio | Acetivibrio clariflavus |
| Z2 | 170 | 30 | 0.07 | 1547597 | NCBI-3-16S@NR_178727.1 | Sanguibacteroides justesenii | Bacteria | Bacteroidota | Bacteroidia | Bacteroidales | Porphyromonadaceae | Sanguibacteroides | Sanguibacteroides justesenii |
| Z2 | 171 | 29 | 0.07 | 2070686 | NCBI-3-16S@NR_179114.1 | Enteroscipio rubneri | Bacteria | Actinomycetota | Coriobacteriia | Eggerthellales | Eggerthellaceae | Enteroscipio | Enteroscipio rubneri |
| Z2 | 172 | 29 | 0.07 | 1720203 | NCBI-3-16S@NR_169358.1 | Butyricimonas phoceensis | Bacteria | Bacteroidota | Bacteroidia | Bacteroidales | Odoribacteraceae | Butyricimonas | Butyricimonas phoceensis |
| Z2 | 173 | 29 | 0.07 | 2049025 | NCBI-3-16S@MN913777.1 | Flavonifractor sp. | Bacteria | Bacillota | Clostridia | Eubacteriales | Oscillospiraceae | Flavonifractor | Flavonifractor sp. |
| Z2 | 174 | 29 | 0.07 | 3046383 | NCBI-3-16S@ON706274.1 | Aristaeella lactis | Bacteria | Bacillota | Clostridia | Eubacteriales | Aristaeellaceae | Aristaeella | Aristaeella lactis |
| Z2 | 175 | 28 | 0.07 | 936069 | NCBI-3-16S@AB596885.1 | Clostridium sp. 6-44 | Bacteria | Bacillota | Clostridia | Eubacteriales | Clostridiaceae | Clostridium | Clostridium sp. 6-44 |
| Z2 | 176 | 28 | 0.07 | 2725562 | NCBI-3-16S@NR_181379.1 | Caecibacteroides pullorum | Bacteria | Bacteroidota | Bacteroidia | Bacteroidales | Bacteroidaceae | Caecibacteroides | Caecibacteroides pullorum |
| Z2 | 177 | 28 | 0.07 | 2049021 | NCBI-3-16S@MK287664.1 | Butyricicoccus sp. | Bacteria | Bacillota | Clostridia | Eubacteriales | Butyricicoccaceae | Butyricicoccus | Butyricicoccus sp. |
| Z2 | 178 | 28 | 0.07 | 2211183 | NCBI-3-16S@NR_180148.1 | Evtepia gabavorous | Bacteria | Bacillota | Clostridia | Eubacteriales |  | Evtepia | Evtepia gabavorous |
| Z2 | 179 | 27 | 0.06 | 298183 | NCBI-3-16S@AY756145.2 | anaerobic bacterium Glu3 | Bacteria | Bacillota | Clostridia | Eubacteriales |  |  | anaerobic bacterium Glu3 |
| Z2 | 180 | 27 | 0.06 | 100176 | NCBI-3-16S@NR_025025.1 | Papillibacter cinnamivorans | Bacteria | Bacillota | Clostridia | Eubacteriales | Oscillospiraceae | Papillibacter | Papillibacter cinnamivorans |
| Z2 | 181 | 27 | 0.06 | 446660 | NCBI-3-16S@OK510307.1 | Adlercreutzia equolifaciens | Bacteria | Actinomycetota | Coriobacteriia | Eggerthellales | Eggerthellaceae | Adlercreutzia | Adlercreutzia equolifaciens |
| Z2 | 182 | 27 | 0.06 | 1837333 | NCBI-3-16S@KX009930.1 | Lachnospiraceae bacterium DW67 | Bacteria | Bacillota | Clostridia | Lachnospirales | Lachnospiraceae |  | Lachnospiraceae bacterium DW67 |
| Z2 | 183 | 27 | 0.06 | 399361 | NCBI-3-16S@DQ833401.1 | Sphaerochaeta sp. RCcp2 | Bacteria | Spirochaetota | Spirochaetia | Spirochaetales | Sphaerochaetaceae | Sphaerochaeta | Sphaerochaeta sp. RCcp2 |
| Z2 | 184 | 27 | 0.06 | 172901 | NCBI-3-16S@MN537538.1 | Victivallis vadensis | Bacteria | Lentisphaerota | Lentisphaeria | Victivallales | Victivallaceae | Victivallis | Victivallis vadensis |
| Z2 | 185 | 26 | 0.06 | 2740579 | NCBI-3-16S@OQ989632.1 | Streptococcus vicugnae | Bacteria | Bacillota | Bacilli | Lactobacillales | Streptococcaceae | Streptococcus | Streptococcus vicugnae |
| Z2 | 186 | 26 | 0.06 | 1898207 | NCBI-3-16S@ON714588.1 | Clostridiales bacterium | Bacteria | Bacillota | Clostridia | Eubacteriales |  |  | Clostridiales bacterium |
| Z2 | 187 | 26 | 0.06 | 569452 | NCBI-3-16S@FJ269086.1 | iron-reducing bacterium enrichment culture clone HN-HFO10 | Bacteria |  |  |  |  |  | iron-reducing bacterium enrichment culture clone HN-HFO10 |
| Z2 | 188 | 26 | 0.06 | 1264 | NCBI-3-16S@X85098.1 | Ruminococcus albus | Bacteria | Bacillota | Clostridia | Eubacteriales | Oscillospiraceae | Ruminococcus | Ruminococcus albus |
| Z2 | 189 | 25 | 0.06 | 376806 | NCBI-3-16S@NR_041448.1 | Bacteroides gallinarum | Bacteria | Bacteroidota | Bacteroidia | Bacteroidales | Bacteroidaceae | Bacteroides | Bacteroides gallinarum |
| Z2 | 190 | 25 | 0.06 | 351091 | NCBI-3-16S@MH282444.1 | Oscillibacter valericigenes | Bacteria | Bacillota | Clostridia | Eubacteriales | Oscillospiraceae | Oscillibacter | Oscillibacter valericigenes |
| Z2 | 191 | 25 | 0.06 | 329854 | NCBI-3-16S@NR_041307.1 | Bacteroides intestinalis | Bacteria | Bacteroidota | Bacteroidia | Bacteroidales | Bacteroidaceae | Bacteroides | Bacteroides intestinalis |
| Z2 | 192 | 25 | 0.06 | 1898206 | NCBI-3-16S@OQ150036.1 | Spirochaetaceae bacterium | Bacteria | Spirochaetota | Spirochaetia | Spirochaetales | Spirochaetaceae |  | Spirochaetaceae bacterium |
| Z2 | 193 | 25 | 0.06 | 1156936 | NCBI-3-16S@JQ404436.1 | Clostridium sp. WSC-9-7 | Bacteria | Bacillota | Clostridia | Eubacteriales | Clostridiaceae | Clostridium | Clostridium sp. WSC-9-7 |
| Z2 | 194 | 25 | 0.06 | 742742 | NCBI-3-16S@NR_113273.1 | Collinsella tanakaei YIT 12063 | Bacteria | Actinomycetota | Coriobacteriia | Coriobacteriales | Coriobacteriaceae | Collinsella | Collinsella tanakaei |
| Z2 | 195 | 25 | 0.06 | 1159215 | NCBI-3-16S@AB702928.1 | Clostridiales bacterium CIEAF 020 | Bacteria | Bacillota | Clostridia | Eubacteriales |  |  | Clostridiales bacterium CIEAF 020 |
| Z2 | 196 | 25 | 0.06 | 1841856 | NCBI-3-16S@NR_144744.1 | Bacteroides mediterraneensis | Bacteria | Bacteroidota | Bacteroidia | Bacteroidales | Bacteroidaceae | Bacteroides | Bacteroides mediterraneensis |
| Z2 | 197 | 24 | 0.06 | 2986072 | NCBI-3-16S@OX352005.1 | Candidatus Minimicrobia sp. IHU4 | Bacteria | Candidatus Saccharibacteria |  |  |  | Candidatus Minimicrobia | Candidatus Minimicrobia sp. IHU4 |
| Z2 | 198 | 24 | 0.06 | 74707 | NCBI-3-16S@MF678906.1 | Staphylococcus schleiferi subsp. schleiferi | Bacteria | Bacillota | Bacilli | Bacillales | Staphylococcaceae | Staphylococcus | Staphylococcus schleiferi |
| Z2 | 199 | 24 | 0.06 | 1394756 | NCBI-3-16S@AB849338.1 | Ruminococcaceae bacterium CG7 | Bacteria | Bacillota | Clostridia | Eubacteriales | Oscillospiraceae |  | Ruminococcaceae bacterium CG7 |
| Z2 | 200 | 24 | 0.06 | 1872444 | NCBI-3-16S@OK067667.1 | Alistipes sp. | Bacteria | Bacteroidota | Bacteroidia | Bacteroidales | Rikenellaceae | Alistipes | Alistipes sp. |
| Z2 | 201 | 24 | 0.06 | 1871018 | NCBI-3-16S@NR_179559.1 | Angelakisella massiliensis | Bacteria | Bacillota | Clostridia | Eubacteriales | Oscillospiraceae | Angelakisella | Angelakisella massiliensis |
| Z2 | 202 | 24 | 0.06 | 2496531 | NCBI-3-16S@MK287625.1 | Murimonas sp. | Bacteria | Bacillota | Clostridia | Lachnospirales | Lachnospiraceae | Murimonas | Murimonas sp. |
| Z2 | 203 | 24 | 0.06 | 349096 | NCBI-3-16S@NR_043658.1 | Pectinatus haikarae | Bacteria | Bacillota | Negativicutes | Selenomonadales | Selenomonadaceae | Pectinatus | Pectinatus haikarae |
| Z2 | 204 | 24 | 0.06 | 397286 | NCBI-3-16S@DQ789124.1 | Lachnospiraceae bacterium 14-2 | Bacteria | Bacillota | Clostridia | Lachnospirales | Lachnospiraceae |  | Lachnospiraceae bacterium 14-2 |
| Z2 | 205 | 24 | 0.06 | 1628085 | NCBI-3-16S@NR_151982.1 | Agathobaculum butyriciproducens | Bacteria | Bacillota | Clostridia | Eubacteriales | Butyricicoccaceae | Agathobaculum | Agathobaculum butyriciproducens |
| Z2 | 206 | 23 | 0.05 | 936073 | NCBI-3-16S@AB596889.1 | Acetivibrio sp. 6-13 | Bacteria | Bacillota | Clostridia | Eubacteriales | Oscillospiraceae | Acetivibrio | Acetivibrio sp. 6-13 |
| Z2 | 207 | 23 | 0.05 | 2049037 | NCBI-3-16S@OM658616.1 | Parasutterella sp. | Bacteria | Pseudomonadota | Betaproteobacteria | Burkholderiales | Sutterellaceae | Parasutterella | Parasutterella sp. |
| Z2 | 208 | 23 | 0.05 | 2692629 | NCBI-3-16S@NR_181133.1 | Copranaerobaculum intestinale | Bacteria | Bacillota | Erysipelotrichia | Erysipelotrichales | Erysipelotrichaceae | Copranaerobaculum | Copranaerobaculum intestinale |
| Z2 | 209 | 23 | 0.05 | 1841866 | NCBI-3-16S@NR_179526.1 | Oscillibacter massiliensis | Bacteria | Bacillota | Clostridia | Eubacteriales | Oscillospiraceae | Oscillibacter | Oscillibacter massiliensis |
| Z2 | 210 | 23 | 0.05 | 3046383 | NCBI-3-16S@KF698430.1 | Aristaeella lactis | Bacteria | Bacillota | Clostridia | Eubacteriales | Aristaeellaceae | Aristaeella | Aristaeella lactis |
| Z2 | 211 | 23 | 0.05 | 2606638 | NCBI-3-16S@NR_180814.1 | Bullifex porci | Bacteria | Spirochaetota | Spirochaetia | Spirochaetales | Spirochaetaceae | Bullifex | Bullifex porci |
| Z2 | 212 | 23 | 0.05 | 1968902 | NCBI-3-16S@OK067651.1 | Gordonibacter sp. | Bacteria | Actinomycetota | Coriobacteriia | Eggerthellales | Eggerthellaceae | Gordonibacter | Gordonibacter sp. |
| Z2 | 213 | 22 | 0.05 | 2585119 | NCBI-3-16S@NR_179296.1 | Alistipes dispar | Bacteria | Bacteroidota | Bacteroidia | Bacteroidales | Rikenellaceae | Alistipes | Alistipes dispar |
| Z2 | 214 | 22 | 0.05 | 1240100 | NCBI-3-16S@AB752501.1 | Coriobacteriaceae bacterium SNR48-44 | Bacteria | Actinomycetota | Coriobacteriia | Coriobacteriales | Coriobacteriaceae |  | Coriobacteriaceae bacterium SNR48-44 |
| Z2 | 215 | 22 | 0.05 | 762968 | NCBI-3-16S@NR_041626.1 | Paraprevotella clara YIT 11840 | Bacteria | Bacteroidota | Bacteroidia | Bacteroidales | Prevotellaceae | Paraprevotella | Paraprevotella clara |
| Z2 | 216 | 22 | 0.05 | 2518971 | NCBI-3-16S@NR_170508.1 | Duncaniella dubosii | Bacteria | Bacteroidota | Bacteroidia | Bacteroidales | Muribaculaceae | Duncaniella | Duncaniella dubosii |
| Z2 | 217 | 22 | 0.05 | 889071 | NCBI-3-16S@HQ222293.1 | Clostridium sp. enrichment culture clone VanCtr97 | Bacteria | Bacillota | Clostridia | Eubacteriales | Clostridiaceae | Clostridium | Clostridium sp. enrichment culture clone VanCtr97 |
| Z2 | 218 | 22 | 0.05 | 691816 | NCBI-3-16S@NR_113072.1 | Bacteroides rodentium | Bacteria | Bacteroidota | Bacteroidia | Bacteroidales | Bacteroidaceae | Bacteroides | Bacteroides rodentium |
| Z2 | 219 | 22 | 0.05 | 2764593 | NCBI-3-16S@MT905164.1 | Parabacteroides sp. 426_9 | Bacteria | Bacteroidota | Bacteroidia | Bacteroidales | Tannerellaceae | Parabacteroides | Parabacteroides sp. 426_9 |
| Z2 | 220 | 22 | 0.05 | 692055 | NCBI-3-16S@FJ808609.1 | Clostridium sp. 6-16 | Bacteria | Bacillota | Clostridia | Eubacteriales | Clostridiaceae | Clostridium | Clostridium sp. 6-16 |
| Z2 | 221 | 21 | 0.05 | 2763053 | NCBI-3-16S@MT905206.1 | Lawsonibacter hominis | Bacteria | Bacillota | Clostridia | Eubacteriales | Oscillospiraceae | Lawsonibacter | Lawsonibacter hominis |
| Z2 | 222 | 21 | 0.05 | 1151542 | NCBI-3-16S@JN713451.1 | Prevotella sp. canine oral taxon 284 | Bacteria | Bacteroidota | Bacteroidia | Bacteroidales | Prevotellaceae | Prevotella | Prevotella sp. canine oral taxon 284 |
| Z2 | 223 | 21 | 0.05 | 28116 | NCBI-3-16S@MH719047.1 | Bacteroides ovatus | Bacteria | Bacteroidota | Bacteroidia | Bacteroidales | Bacteroidaceae | Bacteroides | Bacteroides ovatus |
| Z2 | 224 | 21 | 0.05 | 1977263 | NCBI-3-16S@MN081645.1 | Enterorhabdus sp. | Bacteria | Actinomycetota | Coriobacteriia | Eggerthellales | Eggerthellaceae | Enterorhabdus | Enterorhabdus sp. |
| Z2 | 225 | 21 | 0.05 | 1936999 | NCBI-3-16S@LT722679.1 | Lactonifactor sp. Marseille-P3743 | Bacteria | Bacillota | Clostridia | Eubacteriales | Clostridiaceae | Lactonifactor | Lactonifactor sp. Marseille-P3743 |
| Z2 | 226 | 21 | 0.05 | 569465 | NCBI-3-16S@FJ269084.1 | iron-reducing bacterium enrichment culture clone HN-HFO4 | Bacteria |  |  |  |  |  | iron-reducing bacterium enrichment culture clone HN-HFO4 |
| Z2 | 227 | 20 | 0.05 | 2773926 | NCBI-3-16S@OP762691.2 | Phocaeicola sp. | Bacteria | Bacteroidota | Bacteroidia | Bacteroidales | Bacteroidaceae | Phocaeicola | Phocaeicola sp. |
| Z2 | 228 | 20 | 0.05 | 2763660 | NCBI-3-16S@NR_181384.1 | Feifania hominis | Bacteria | Bacillota | Clostridia | Eubacteriales | Feifaniaceae | Feifania | Feifania hominis |
| Z2 | 229 | 20 | 0.05 | 1872444 | NCBI-3-16S@OK067667.1 | Alistipes sp. | Bacteria | Bacteroidota | Bacteroidia | Bacteroidales | Rikenellaceae | Alistipes | Alistipes sp. |
| Z2 | 230 | 20 | 0.05 | 2163169 | NCBI-3-16S@MN081684.1 | Kineothrix sp. | Bacteria | Bacillota | Clostridia | Lachnospirales | Lachnospiraceae | Kineothrix | Kineothrix sp. |
| Z2 | 231 | 20 | 0.05 | 519017 | NCBI-3-16S@EU592964.1 | Olsenella sp. F0004 | Bacteria | Actinomycetota | Coriobacteriia | Coriobacteriales | Atopobiaceae | Olsenella | Olsenella sp. F0004 |
| Z2 | 232 | 20 | 0.05 | 1155414 | NCBI-3-16S@JN688037.1 | bacterium enrichment culture clone M235 | Bacteria |  |  |  |  |  | bacterium enrichment culture clone M235 |
| Z2 | 233 | 20 | 0.05 | 2039241 | NCBI-3-16S@MK287717.1 | Anaerotignum sp. | Bacteria | Bacillota | Clostridia | Lachnospirales | Anaerotignaceae | Anaerotignum | Anaerotignum sp. |
| Z2 | 234 | 20 | 0.05 | 2290935 | NCBI-3-16S@NR_180156.1 | Parabacteroides acidifaciens | Bacteria | Bacteroidota | Bacteroidia | Bacteroidales | Tannerellaceae | Parabacteroides | Parabacteroides acidifaciens |
| Z2 | 235 | 19 | 0.05 | 1159224 | NCBI-3-16S@AB702938.1 | Clostridiales bacterium CIEAF 022 | Bacteria | Bacillota | Clostridia | Eubacteriales |  |  | Clostridiales bacterium CIEAF 022 |
| Z2 | 236 | 19 | 0.05 | 76124 | NCBI-3-16S@U13037.1 | [Eubacterium] minutum | Bacteria | Bacillota | Clostridia | Eubacteriales | Eubacteriales Family XIII. Incertae Sedis |  | [Eubacterium] minutum |
| Z2 | 237 | 19 | 0.05 | 1453586 | NCBI-3-16S@KF809885.1 | Alistipes sp. CC-5826-wt-bac | Bacteria | Bacteroidota | Bacteroidia | Bacteroidales | Rikenellaceae | Alistipes | Alistipes sp. CC-5826-wt-bac |
| Z2 | 238 | 19 | 0.05 | 46506 | NCBI-3-16S@OR554138.1 | Bacteroides stercoris | Bacteria | Bacteroidota | Bacteroidia | Bacteroidales | Bacteroidaceae | Bacteroides | Bacteroides stercoris |
| Z2 | 239 | 19 | 0.05 | 1003345 | NCBI-3-16S@AB622816.1 | Clostridium sp. Culture-23 | Bacteria | Bacillota | Clostridia | Eubacteriales | Clostridiaceae | Clostridium | Clostridium sp. Culture-23 |
| Z2 | 240 | 19 | 0.05 | 2042683 | NCBI-3-16S@MT323094.2 | Olsenella sp. | Bacteria | Actinomycetota | Coriobacteriia | Coriobacteriales | Atopobiaceae | Olsenella | Olsenella sp. |
| Z2 | 241 | 19 | 0.05 | 2850323 | NCBI-3-16S@NR_181407.1 | Diplocloster agilis | Bacteria | Bacillota | Clostridia | Lachnospirales | Lachnospiraceae | Diplocloster | Diplocloster agilis |
| Z2 | 242 | 18 | 0.04 | 2041843 | NCBI-3-16S@LT934455.1 | Anaeromassilibacillus sp. Marseille-P4683 | Bacteria | Bacillota | Clostridia | Eubacteriales | Acutalibacteraceae | Anaeromassilibacillus | Anaeromassilibacillus sp. Marseille-P4683 |
| Z2 | 243 | 18 | 0.04 | 1577241 | NCBI-3-16S@KM461992.1 | Bacteroidia bacterium feline oral taxon 115 | Bacteria | Bacteroidota | Bacteroidia |  |  |  | Bacteroidia bacterium feline oral taxon 115 |
| Z2 | 244 | 18 | 0.04 | 42458 | NCBI-3-18S@HM590655.1 | Lichtheimia corymbifera | Eukaryota | Mucoromycota | Mucoromycetes | Mucorales | Lichtheimiaceae | Lichtheimia | Lichtheimia corymbifera |
| Z2 | 245 | 18 | 0.04 | 241556 | NCBI-3-16S@AY341819.1 | Bacteroidales genomosp. P1 | Bacteria | Bacteroidota | Bacteroidia | Bacteroidales |  |  | Bacteroidales genomosp. P1 |
| Z2 | 246 | 18 | 0.04 | 2108523 | NCBI-3-16S@NR_173697.1 | Lawsonibacter asaccharolyticus | Bacteria | Bacillota | Clostridia | Eubacteriales | Oscillospiraceae | Lawsonibacter | Lawsonibacter asaccharolyticus |
| Z2 | 247 | 18 | 0.04 | 608506 | NCBI-3-16S@NR_117295.1 | Caldicellulosiruptor obsidiansis OB47 | Bacteria | Bacillota |  | Caldicellulosiruptorales | Caldicellulosiruptoraceae | Caldicellulosiruptor | Caldicellulosiruptor obsidiansis |
| Z2 | 248 | 18 | 0.04 | 675999 | NCBI-3-16S@GQ503875.1 | bacterium enrichment culture clone Ecwsrb038 | Bacteria |  |  |  |  |  | bacterium enrichment culture clone Ecwsrb038 |
| Z2 | 249 | 18 | 0.04 | 2687240 | NCBI-3-16S@NR_165703.1 | Zunongwangia flava | Bacteria | Bacteroidota | Flavobacteriia | Flavobacteriales | Flavobacteriaceae | Zunongwangia | Zunongwangia flava |
| Z2 | 250 | 18 | 0.04 | 569500 | NCBI-3-16S@FJ269045.1 | iron-reducing bacterium enrichment culture clone HN3 | Bacteria |  |  |  |  |  | iron-reducing bacterium enrichment culture clone HN3 |
| Z2 | 251 | 18 | 0.04 | 1907662 | NCBI-3-16S@NR_148574.1 | Raoultibacter timonensis | Bacteria | Actinomycetota | Coriobacteriia | Eggerthellales | Eggerthellaceae | Raoultibacter | Raoultibacter timonensis |
| Z2 | 252 | 18 | 0.04 | 4522 | NCBI-3-18S@XR_007879504.1 | Lolium perenne | Eukaryota | Streptophyta | Magnoliopsida | Poales | Poaceae | Lolium | Lolium perenne |
| Z2 | 253 | 18 | 0.04 | 1309939 | NCBI-3-16S@KC441622.1 | bacterium enrichment culture clone ZZ_F11b | Bacteria |  |  |  |  |  | bacterium enrichment culture clone ZZ_F11b |
| Z2 | 254 | 17 | 0.04 | 1211843 | NCBI-3-16S@JX101688.1 | Candidatus Soleaferrea massiliensis AP7 | Bacteria | Bacillota | Clostridia | Eubacteriales |  | Candidatus Soleaferrea | Candidatus Soleaferrea massiliensis |
| Z2 | 255 | 17 | 0.04 | 763034 | NCBI-3-16S@NR_113068.1 | Bacteroides fluxus YIT 12057 | Bacteria | Bacteroidota | Bacteroidia | Bacteroidales | Bacteroidaceae | Bacteroides | Bacteroides fluxus |
| Z2 | 256 | 17 | 0.04 | 446660 | NCBI-3-16S@NR_121696.1 | Adlercreutzia equolifaciens | Bacteria | Actinomycetota | Coriobacteriia | Eggerthellales | Eggerthellaceae | Adlercreutzia | Adlercreutzia equolifaciens |
| Z2 | 257 | 17 | 0.04 | 2046249 | NCBI-3-16S@MT312829.1 | Centipeda sp. (in: firmicutes) | Bacteria | Bacillota | Negativicutes | Selenomonadales | Selenomonadaceae | Centipeda | Centipeda sp. (in: firmicutes) |
| Z2 | 258 | 16 | 0.04 | 1414721 | NCBI-3-16S@NR_144697.1 | Clostridium jeddahense | Bacteria | Bacillota | Clostridia | Eubacteriales | Oscillospiraceae | Faecalispora | Faecalispora jeddahensis |
| Z2 | 259 | 16 | 0.04 | 1872444 | NCBI-3-16S@OM658550.1 | Alistipes sp. | Bacteria | Bacteroidota | Bacteroidia | Bacteroidales | Rikenellaceae | Alistipes | Alistipes sp. |
| Z2 | 260 | 16 | 0.04 | 1776382 | NCBI-3-16S@LN998059.1 | Neglectibacter timonensis | Bacteria | Bacillota | Clostridia | Eubacteriales | Oscillospiraceae | Neglectibacter | Neglectibacter timonensis |
| Z2 | 261 | 16 | 0.04 | 880526 | NCBI-3-16S@AB971798.1 | Rikenella microfusus DSM 15922 | Bacteria | Bacteroidota | Bacteroidia | Bacteroidales | Rikenellaceae | Rikenella | Rikenella microfusus |
| Z2 | 262 | 16 | 0.04 | 1159222 | NCBI-3-16S@AB702936.1 | Clostridiales bacterium CIEAF 019 | Bacteria | Bacillota | Clostridia | Eubacteriales |  |  | Clostridiales bacterium CIEAF 019 |
| Z2 | 263 | 16 | 0.04 | 1161950 | NCBI-3-16S@JQ773354.1 | bacterium enrichment culture clone PKS4 | Bacteria |  |  |  |  |  | bacterium enrichment culture clone PKS4 |
| Z2 | 264 | 16 | 0.04 | 1462571 | NCBI-3-16S@NR_148822.1 | Bacteroides gallinaceum | Bacteria | Bacteroidota | Bacteroidia | Bacteroidales | Bacteroidaceae | Bacteroides | Bacteroides gallinaceum |
| Z2 | 265 | 16 | 0.04 | 2530391 | NCBI-3-16S@OK626601.1 | Duncaniella freteri | Bacteria | Bacteroidota | Bacteroidia | Bacteroidales | Muribaculaceae | Duncaniella | Duncaniella freteri |
| Z2 | 266 | 16 | 0.04 | 1972642 | NCBI-3-16S@MG696667.1 | Sphaerochaeta sp. | Bacteria | Spirochaetota | Spirochaetia | Spirochaetales | Sphaerochaetaceae | Sphaerochaeta | Sphaerochaeta sp. |
| Z2 | 267 | 15 | 0.04 | 1030136 | NCBI-3-16S@JF813177.1 | Parabacteroides sp. dnLKV8 | Bacteria | Bacteroidota | Bacteroidia | Bacteroidales | Tannerellaceae | Parabacteroides | Parabacteroides sp. dnLKV8 |
| Z2 | 268 | 15 | 0.04 | 2364795 | NCBI-3-16S@LS999996.1 | Olsenella sp. Marseille-P4559 | Bacteria | Actinomycetota | Coriobacteriia | Coriobacteriales | Atopobiaceae | Olsenella | Olsenella sp. Marseille-P4559 |
| Z2 | 269 | 15 | 0.04 | 2930083 | NCBI-3-16S@OM533390.1 | Olsenella intestinalis | Bacteria | Actinomycetota | Coriobacteriia | Coriobacteriales | Atopobiaceae | Olsenella | Olsenella intestinalis |
| Z2 | 270 | 15 | 0.04 | 1720195 | NCBI-3-16S@NR_146820.1 | Gabonibacter massiliensis | Bacteria | Bacteroidota | Bacteroidia | Bacteroidales | Porphyromonadaceae | Gabonibacter | Gabonibacter massiliensis |
| Z2 | 271 | 15 | 0.04 | 847 | NCBI-3-16S@HG917902.1 | Oxalobacter formigenes | Bacteria | Pseudomonadota | Betaproteobacteria | Burkholderiales | Oxalobacteraceae | Oxalobacter | Oxalobacter formigenes |
| Z2 | 272 | 15 | 0.04 | 437897 | NCBI-3-16S@MN055955.1 | Megamonas funiformis | Bacteria | Bacillota | Negativicutes | Selenomonadales | Selenomonadaceae | Megamonas | Megamonas funiformis |
| Z2 | 273 | 15 | 0.04 | 689779 | NCBI-3-16S@GU124470.1 | Eubacterium sp. SA11 | Bacteria | Bacillota | Clostridia | Eubacteriales | Eubacteriaceae | Eubacterium | Eubacterium sp. SA11 |
| Z2 | 274 | 15 | 0.04 | 1515 | NCBI-3-16S@FN555230.1 | Acetivibrio thermocellus | Bacteria | Bacillota | Clostridia | Eubacteriales | Oscillospiraceae | Acetivibrio | Acetivibrio thermocellus |
| Z2 | 275 | 15 | 0.04 | 2779354 | NCBI-3-16S@NR_180622.1 | Gemmiger gallinarum | Bacteria | Bacillota | Clostridia | Eubacteriales |  | Gemmiger | Gemmiger gallinarum |
| Z2 | 276 | 15 | 0.04 | 1211813 | NCBI-3-16S@NR_144706.1 | Alistipes ihumii AP11 | Bacteria | Bacteroidota | Bacteroidia | Bacteroidales | Rikenellaceae | Alistipes | Alistipes ihumii |
| Z2 | 277 | 15 | 0.04 | 2011094 | NCBI-3-16S@MN055926.1 | Coriobacteriaceae bacterium | Bacteria | Actinomycetota | Coriobacteriia | Coriobacteriales | Coriobacteriaceae |  | Coriobacteriaceae bacterium |
| Z2 | 278 | 15 | 0.04 | 2763656 | NCBI-3-16S@NR_181387.1 | Guopingia tenuis | Bacteria | Bacillota | Clostridia | Christensenellales | Christensenellaceae | Guopingia | Guopingia tenuis |
| Z2 | 279 | 15 | 0.04 | 997349 | NCBI-3-16S@NR_116939.1 | Olsenella profusa DSM 13989 | Bacteria | Actinomycetota | Coriobacteriia | Coriobacteriales | Atopobiaceae | Olsenella | Olsenella profusa |
| Z2 | 280 | 15 | 0.04 | 1003374 | NCBI-3-16S@AB622845.1 | Clostridium sp. Clone-44 | Bacteria | Bacillota | Clostridia | Eubacteriales | Clostridiaceae | Clostridium | Clostridium sp. Clone-44 |
| Z2 | 281 | 15 | 0.04 | 1898207 | NCBI-3-16S@MK170164.1 | Clostridiales bacterium | Bacteria | Bacillota | Clostridia | Eubacteriales |  |  | Clostridiales bacterium |
| Z2 | 282 | 15 | 0.04 | 590931 | NCBI-3-16S@AB477432.1 | Clostridiales bacterium SY8526 | Bacteria | Bacillota | Clostridia | Eubacteriales |  |  | Clostridiales bacterium SY8526 |
| Z2 | 283 | 15 | 0.04 | 1796620 | NCBI-3-16S@NR_144605.1 | Acutalibacter muris | Bacteria | Bacillota | Clostridia | Eubacteriales | Acutalibacteraceae | Acutalibacter | Acutalibacter muris |
| Z2 | 284 | 14 | 0.03 | 1647673 | NCBI-3-16S@KR232914.1 | Barnesiella sp. S496 | Bacteria | Bacteroidota | Bacteroidia | Bacteroidales | Barnesiellaceae | Barnesiella | Barnesiella sp. S496 |
| Z2 | 285 | 14 | 0.03 | 742818 | NCBI-3-16S@NR_113272.1 | Slackia piriformis YIT 12062 | Bacteria | Actinomycetota | Coriobacteriia | Eggerthellales | Eggerthellaceae | Slackia | Slackia piriformis |
| Z2 | 286 | 14 | 0.03 | 2786956 | NCBI-3-16S@NR_181010.1 | Phocaeicola faecalis | Bacteria | Bacteroidota | Bacteroidia | Bacteroidales | Bacteroidaceae | Phocaeicola | Phocaeicola faecalis |
| Z2 | 287 | 14 | 0.03 | 1977263 | NCBI-3-16S@MK929053.1 | Enterorhabdus sp. | Bacteria | Actinomycetota | Coriobacteriia | Eggerthellales | Eggerthellaceae | Enterorhabdus | Enterorhabdus sp. |
| Z2 | 288 | 14 | 0.03 | 1965314 | NCBI-3-16S@MT829551.1 | Roseivivax sp. | Bacteria | Pseudomonadota | Alphaproteobacteria | Rhodobacterales | Roseobacteraceae | Roseivivax | Roseivivax sp. |
| Z2 | 289 | 14 | 0.03 | 1472417 | NCBI-3-16S@NR_126195.1 | Butyricimonas paravirosa | Bacteria | Bacteroidota | Bacteroidia | Bacteroidales | Odoribacteraceae | Butyricimonas | Butyricimonas paravirosa |
| Z2 | 290 | 14 | 0.03 | 29323 | NCBI-3-16S@NR_117608.1 | Thermoanaerobacter brockii | Bacteria | Bacillota | Clostridia | Thermoanaerobacterales | Thermoanaerobacteraceae | Thermoanaerobacter | Thermoanaerobacter brockii |
| Z2 | 291 | 14 | 0.03 | 2049040 | NCBI-3-16S@MZ310605.1 | Roseburia sp. | Bacteria | Bacillota | Clostridia | Lachnospirales | Lachnospiraceae | Roseburia | Roseburia sp. |
| Z2 | 292 | 14 | 0.03 | 1965293 | NCBI-3-16S@MK287656.1 | Intestinimonas sp. | Bacteria | Bacillota | Clostridia | Eubacteriales |  | Intestinimonas | Intestinimonas sp. |
| Z2 | 293 | 14 | 0.03 | 35830 | NCBI-3-16S@JQ820024.1 | Acetivibrio cellulolyticus | Bacteria | Bacillota | Clostridia | Eubacteriales | Oscillospiraceae | Acetivibrio | Acetivibrio cellulolyticus |
| Z2 | 294 | 14 | 0.03 | 2899121 | NCBI-3-16S@NR_179945.1 | Tannockella kyphosi | Bacteria | Bacillota | Erysipelotrichia | Erysipelotrichales | Coprobacillaceae | Tannockella | Tannockella kyphosi |
| Z2 | 295 | 14 | 0.03 | 35830 | NCBI-3-16S@KM036187.1 | Acetivibrio cellulolyticus | Bacteria | Bacillota | Clostridia | Eubacteriales | Oscillospiraceae | Acetivibrio | Acetivibrio cellulolyticus |
| Z2 | 296 | 14 | 0.03 | 1297617 | NCBI-3-16S@MZ310684.1 | Intestinimonas butyriciproducens | Bacteria | Bacillota | Clostridia | Eubacteriales |  | Intestinimonas | Intestinimonas butyriciproducens |
| Z2 | 297 | 14 | 0.03 | 1769629 | NCBI-3-18S@KR025407.1 | Entamoeba sp. RL9 | Eukaryota | Evosea |  | Mastigamoebida | Entamoebidae | Entamoeba | Entamoeba sp. RL9 |
| Z2 | 298 | 13 | 0.03 | 871324 | NCBI-3-16S@NR_113207.1 | Bacteroides stercorirosoris | Bacteria | Bacteroidota | Bacteroidia | Bacteroidales | Bacteroidaceae | Bacteroides | Bacteroides stercorirosoris |
| Z2 | 299 | 13 | 0.03 | 340101 | NCBI-3-16S@NR_118352.1 | Victivallis vadensis ATCC BAA-548 | Bacteria | Lentisphaerota | Lentisphaeria | Victivallales | Victivallaceae | Victivallis | Victivallis vadensis |
| Z2 | 300 | 13 | 0.03 | 2086585 | NCBI-3-16S@NR_189199.1 | Maliibacterium massiliense | Bacteria | Bacillota | Clostridia | Eubacteriales | Maliibacteriaceae | Maliibacterium | Maliibacterium massiliense |
| Z2 | 301 | 13 | 0.03 | 168384 | NCBI-3-16S@NR_114807.1 | Marvinbryantia formatexigens | Bacteria | Bacillota | Clostridia | Lachnospirales | Lachnospiraceae | Marvinbryantia | Marvinbryantia formatexigens |
| Z2 | 302 | 13 | 0.03 | 1907658 | NCBI-3-16S@NR_179567.1 | Bacteroides ilei | Bacteria | Bacteroidota | Bacteroidia | Bacteroidales | Bacteroidaceae | Bacteroides | Bacteroides ilei |
| Z2 | 303 | 13 | 0.03 | 697329 | NCBI-3-16S@NR_074399.1 | Ruminococcus albus 7 = DSM 20455 | Bacteria | Bacillota | Clostridia | Eubacteriales | Oscillospiraceae | Ruminococcus | Ruminococcus albus |
| Z2 | 304 | 13 | 0.03 | 1945634 | NCBI-3-16S@NR_179713.1 | Butyricicoccus porcorum | Bacteria | Bacillota | Clostridia | Eubacteriales | Butyricicoccaceae | Butyricicoccus | Butyricicoccus porcorum |
| Z2 | 305 | 13 | 0.03 | 1155397 | NCBI-3-16S@JN688021.1 | bacterium enrichment culture clone M02 | Bacteria |  |  |  |  |  | bacterium enrichment culture clone M02 |
| Z2 | 306 | 13 | 0.03 | 2981726 | NCBI-3-16S@OK510342.1 | Hominimerdicola aceti | Bacteria | Bacillota | Clostridia | Eubacteriales | Oscillospiraceae | Hominimerdicola | Hominimerdicola aceti |
| Z2 | 307 | 13 | 0.03 | 310298 | NCBI-3-16S@AB200225.1 | Phocaeicola coprocola | Bacteria | Bacteroidota | Bacteroidia | Bacteroidales | Bacteroidaceae | Phocaeicola | Phocaeicola coprocola |
| Z2 | 308 | 13 | 0.03 | 2763655 | NCBI-3-16S@NR_181386.1 | Gehongia tenuis | Bacteria | Bacillota | Clostridia | Christensenellales | Christensenellaceae | Gehongia | Gehongia tenuis |
| Z2 | 309 | 13 | 0.03 | 163547 | NCBI-3-16S@AF385563.1 | Eubacterium sp. oral clone BU014 | Bacteria | Bacillota | Clostridia | Eubacteriales | Eubacteriaceae | Eubacterium | Eubacterium sp. oral clone BU014 |
| Z2 | 310 | 13 | 0.03 | 393752 | NCBI-3-16S@DQ676996.2 | iron-reducing enrichment clone Cl-A4 | Bacteria |  |  |  |  |  | iron-reducing enrichment clone Cl-A4 |
| Z2 | 311 | 13 | 0.03 | 2024197 | NCBI-3-16S@NR_179635.1 | Bacteroides cutis | Bacteria | Bacteroidota | Bacteroidia | Bacteroidales | Bacteroidaceae | Bacteroides | Bacteroides cutis |
| Z2 | 312 | 13 | 0.03 | 2764584 | NCBI-3-16S@MT905190.1 | Lactobacillus sp. NSJ-43 | Bacteria | Bacillota | Bacilli | Lactobacillales | Lactobacillaceae | Lactobacillus | Lactobacillus sp. NSJ-43 |
| Z2 | 313 | 13 | 0.03 | 471189 | NCBI-3-16S@OM760840.1 | Gordonibacter pamelaeae | Bacteria | Actinomycetota | Coriobacteriia | Eggerthellales | Eggerthellaceae | Gordonibacter | Gordonibacter pamelaeae |
| Z2 | 314 | 13 | 0.03 | 1837330 | NCBI-3-16S@KX009927.1 | Lachnospiraceae bacterium DW59 | Bacteria | Bacillota | Clostridia | Lachnospirales | Lachnospiraceae |  | Lachnospiraceae bacterium DW59 |
| Z2 | 315 | 13 | 0.03 | 626937 | NCBI-3-16S@ON705242.1 | Christensenella minuta | Bacteria | Bacillota | Clostridia | Christensenellales | Christensenellaceae | Christensenella | Christensenella minuta |
| Z2 | 316 | 13 | 0.03 | 1470347 | NCBI-3-16S@NR_144706.1 | Alistipes ihumii | Bacteria | Bacteroidota | Bacteroidia | Bacteroidales | Rikenellaceae | Alistipes | Alistipes ihumii |
| Z2 | 317 | 12 | 0.03 | 1510 | NCBI-3-16S@NR_114799.1 | Thermoclostridium stercorarium | Bacteria | Bacillota | Clostridia | Eubacteriales | Oscillospiraceae | Thermoclostridium | Thermoclostridium stercorarium |
| Z2 | 318 | 12 | 0.03 | 2758411 | NCBI-3-16S@MT799857.1 | Mailhella sp. Marseille-Q3435 | Bacteria | Thermodesulfobacteriota | Desulfovibrionia | Desulfovibrionales | Desulfovibrionaceae | Mailhella | Mailhella sp. Marseille-Q3435 |
| Z2 | 319 | 12 | 0.03 | 2773926 | NCBI-3-16S@OK067645.1 | Phocaeicola sp. | Bacteria | Bacteroidota | Bacteroidia | Bacteroidales | Bacteroidaceae | Phocaeicola | Phocaeicola sp. |
| Z2 | 320 | 12 | 0.03 | 433293 | NCBI-3-16S@NR_151894.1 | Anaerotaenia torta | Bacteria | Bacillota | Clostridia | Lachnospirales | Lachnospiraceae | Anaerotaenia | Anaerotaenia torta |
| Z2 | 321 | 12 | 0.03 | 1898207 | NCBI-3-16S@ON714563.1 | Clostridiales bacterium | Bacteria | Bacillota | Clostridia | Eubacteriales |  |  | Clostridiales bacterium |
| Z2 | 322 | 12 | 0.03 | 399354 | NCBI-3-16S@DQ833395.1 | Sphaerochaeta sp. 'Grapes TMA14' | Bacteria | Spirochaetota | Spirochaetia | Spirochaetales | Sphaerochaetaceae | Sphaerochaeta | Sphaerochaeta sp. 'Grapes TMA14' |
| Z2 | 323 | 12 | 0.03 | 1155415 | NCBI-3-16S@JN688038.1 | bacterium enrichment culture clone M244 | Bacteria |  |  |  |  |  | bacterium enrichment culture clone M244 |
| Z2 | 324 | 12 | 0.03 | 2764572 | NCBI-3-16S@MT905216.1 | Caproiciproducens sp. NSJ-41 | Bacteria | Bacillota | Clostridia | Eubacteriales | Acutalibacteraceae | Caproiciproducens | Caproiciproducens sp. NSJ-41 |
| Z2 | 325 | 12 | 0.03 | 1535 | NCBI-3-16S@NR_114789.1 | [Clostridium] leptum | Bacteria | Bacillota | Clostridia | Eubacteriales | Oscillospiraceae |  | [Clostridium] leptum |
| Z2 | 326 | 11 | 0.03 | 1870991 | NCBI-3-16S@NR_179547.1 | Massilioclostridium coli | Bacteria | Bacillota | Clostridia | Eubacteriales | Clostridiaceae | Massilioclostridium | Massilioclostridium coli |
| Z2 | 327 | 11 | 0.03 | 1151611 | NCBI-3-16S@JN713207.1 | Bacteroidia bacterium canine oral taxon 041 | Bacteria | Bacteroidota | Bacteroidia |  |  |  | Bacteroidia bacterium canine oral taxon 041 |
| Z2 | 328 | 11 | 0.03 | 46503 | NCBI-3-16S@OK626625.1 | Parabacteroides merdae | Bacteria | Bacteroidota | Bacteroidia | Bacteroidales | Tannerellaceae | Parabacteroides | Parabacteroides merdae |
| Z2 | 329 | 11 | 0.03 | 1577308 | NCBI-3-16S@KM462079.1 | Lachnospiraceae bacterium feline oral taxon 003 | Bacteria | Bacillota | Clostridia | Lachnospirales | Lachnospiraceae |  | Lachnospiraceae bacterium feline oral taxon 003 |
| Z2 | 330 | 11 | 0.03 | 1155409 | NCBI-3-16S@JN688032.1 | bacterium enrichment culture clone M137 | Bacteria |  |  |  |  |  | bacterium enrichment culture clone M137 |
| Z2 | 331 | 11 | 0.03 | 569505 | NCBI-3-16S@FJ269060.1 | iron-reducing bacterium enrichment culture clone HN51 | Bacteria |  |  |  |  |  | iron-reducing bacterium enrichment culture clone HN51 |
| Z2 | 332 | 11 | 0.03 | 31899 | NCBI-3-16S@L09180.1 | Caldicellulosiruptor bescii | Bacteria | Bacillota |  | Caldicellulosiruptorales | Caldicellulosiruptoraceae | Caldicellulosiruptor | Caldicellulosiruptor bescii |
| Z2 | 333 | 11 | 0.03 | 1874115 | NCBI-3-16S@OR863769.1 | Sphingobacterium alkalisoli | Bacteria | Bacteroidota | Sphingobacteriia | Sphingobacteriales | Sphingobacteriaceae | Sphingobacterium | Sphingobacterium alkalisoli |
| Z2 | 334 | 11 | 0.03 | 2042309 | NCBI-3-16S@NR_179642.1 | Butyricimonas vaginalis | Bacteria | Bacteroidota | Bacteroidia | Bacteroidales | Odoribacteraceae | Butyricimonas | Butyricimonas vaginalis |
| Z2 | 335 | 11 | 0.03 | 626929 | NCBI-3-16S@ON705228.1 | Bacteroides clarus | Bacteria | Bacteroidota | Bacteroidia | Bacteroidales | Bacteroidaceae | Bacteroides | Bacteroides clarus |
| Z2 | 336 | 11 | 0.03 | 2304688 | NCBI-3-16S@OQ921830.1 | Petroclostridium sp. | Bacteria | Bacillota | Clostridia | Eubacteriales | Oscillospiraceae | Petroclostridium | Petroclostridium sp. |
| Z2 | 337 | 11 | 0.03 | 3046383 | NCBI-3-16S@ON706276.1 | Aristaeella lactis | Bacteria | Bacillota | Clostridia | Eubacteriales | Aristaeellaceae | Aristaeella | Aristaeella lactis |
| Z2 | 338 | 11 | 0.03 | 1220747 | NCBI-3-16S@JQ771483.1 | bacterium enrichment culture clone E34 | Bacteria |  |  |  |  |  | bacterium enrichment culture clone E34 |
| Z2 | 339 | 11 | 0.03 | 2779357 | NCBI-3-16S@NR_180618.1 | Thermophilibacter gallinarum | Bacteria | Actinomycetota | Coriobacteriia | Coriobacteriales | Atopobiaceae | Thermophilibacter | Thermophilibacter gallinarum |
| Z2 | 340 | 11 | 0.03 | 2495406 | NCBI-3-16S@LR135780.1 | Alistipes sp. Marseille-P8752 | Bacteria | Bacteroidota | Bacteroidia | Bacteroidales | Rikenellaceae | Alistipes | Alistipes sp. Marseille-P8752 |
| Z2 | 341 | 11 | 0.03 | 28112 | NCBI-3-16S@ON222751.1 | Tannerella forsythia | Bacteria | Bacteroidota | Bacteroidia | Bacteroidales | Tannerellaceae | Tannerella | Tannerella forsythia |
| Z2 | 342 | 11 | 0.03 | 1964384 | NCBI-3-16S@NR_179615.1 | Olsenella congonensis | Bacteria | Actinomycetota | Coriobacteriia | Coriobacteriales | Atopobiaceae | Olsenella | Olsenella congonensis |
| Z2 | 343 | 11 | 0.03 | 182096 | NCBI-3-18S@XR_006253248.1 | Aspergillus chevalieri | Eukaryota | Ascomycota | Eurotiomycetes | Eurotiales | Aspergillaceae | Aspergillus | Aspergillus chevalieri |
| Z2 | 344 | 11 | 0.03 | 537365 | NCBI-3-16S@EU728720.1 | Bacteroidaceae bacterium DJF_B220 | Bacteria | Bacteroidota | Bacteroidia | Bacteroidales | Bacteroidaceae |  | Bacteroidaceae bacterium DJF_B220 |
| Z2 | 345 | 11 | 0.03 | 3046382 | NCBI-3-16S@ON706269.1 | Aristaeella hokkaidonensis | Bacteria | Bacillota | Clostridia | Eubacteriales | Aristaeellaceae | Aristaeella | Aristaeella hokkaidonensis |
| Z2 | 346 | 11 | 0.03 | 2028282 | NCBI-3-16S@MK287761.1 | Lachnoclostridium sp. | Bacteria | Bacillota | Clostridia | Lachnospirales | Lachnospiraceae | Lachnoclostridium | Lachnoclostridium sp. |
| Z2 | 347 | 11 | 0.03 | 871327 | NCBI-3-16S@NR_113208.1 | Parabacteroides chinchillae | Bacteria | Bacteroidota | Bacteroidia | Bacteroidales | Tannerellaceae | Parabacteroides | Parabacteroides chinchillae |
| Z2 | 348 | 11 | 0.03 | 387661 | NCBI-3-16S@MK743930.1 | Parabacteroides johnsonii | Bacteria | Bacteroidota | Bacteroidia | Bacteroidales | Tannerellaceae | Parabacteroides | Parabacteroides johnsonii |
| Z2 | 349 | 11 | 0.03 | 1344447 | NCBI-3-16S@KC404041.1 | Ruminococcaceae bacterium enrichment culture clone MRHull-S-07B | Bacteria | Bacillota | Clostridia | Eubacteriales | Oscillospiraceae |  | Ruminococcaceae bacterium enrichment culture clone MRHull-S-07B |
| Z2 | 350 | 11 | 0.03 | 1647705 | NCBI-3-16S@KR232858.1 | Parabacteroides sp. S229 | Bacteria | Bacteroidota | Bacteroidia | Bacteroidales | Tannerellaceae | Parabacteroides | Parabacteroides sp. S229 |
| Z2 | 351 | 10 | 0.02 | 2763675 | NCBI-3-16S@MT905151.1 | Jilunia laotingensis | Bacteria | Bacteroidota | Bacteroidia | Bacteroidales | Bacteroidaceae | Jilunia | Jilunia laotingensis |
| Z2 | 352 | 10 | 0.02 | 575334 | NCBI-3-16S@AB470322.1 | Bacteroides sp. F-4 | Bacteria | Bacteroidota | Bacteroidia | Bacteroidales | Bacteroidaceae | Bacteroides | Bacteroides sp. F-4 |
| Z2 | 353 | 10 | 0.02 | 2053611 | NCBI-3-16S@MW714943.1 | Selenomonas sp. | Bacteria | Bacillota | Negativicutes | Selenomonadales | Selenomonadaceae | Selenomonas | Selenomonas sp. |
| Z2 | 354 | 10 | 0.02 | 1622075 | NCBI-3-16S@NR_173694.1 | Olsenella massiliensis | Bacteria | Actinomycetota | Coriobacteriia | Coriobacteriales | Atopobiaceae | Olsenella | Olsenella massiliensis |
| Z2 | 355 | 10 | 0.02 | 487174 | NCBI-3-16S@NR_113073.1 | Barnesiella intestinihominis | Bacteria | Bacteroidota | Bacteroidia | Bacteroidales | Barnesiellaceae | Barnesiella | Barnesiella intestinihominis |
| Z2 | 356 | 10 | 0.02 | 2448052 | NCBI-3-16S@OR803719.1 | Geminicoccaceae bacterium | Bacteria | Pseudomonadota | Alphaproteobacteria | Geminicoccales | Geminicoccaceae |  | Geminicoccaceae bacterium |
| Z2 | 357 | 10 | 0.02 | 1980694 | NCBI-3-16S@MK287667.1 | Negativibacillus sp. | Bacteria | Bacillota |  |  |  | Negativibacillus | Negativibacillus sp. |
| Z2 | 358 | 10 | 0.02 | 1347151 | NCBI-3-16S@KF030226.1 | Petrimonas sp. canine oral taxon 434 | Bacteria | Bacteroidota | Bacteroidia | Bacteroidales | Dysgonomonadaceae | Petrimonas | Petrimonas sp. canine oral taxon 434 |
| Z2 | 359 | 10 | 0.02 | 2763657 | NCBI-3-16S@NR_181389.1 | Congzhengia minquanensis | Bacteria | Bacillota | Clostridia | Eubacteriales | Oscillospiraceae | Congzhengia | Congzhengia minquanensis |
| Z2 | 360 | 10 | 0.02 | 2033137 | NCBI-3-16S@MN595019.1 | Marinifilum sp. | Bacteria | Bacteroidota | Bacteroidia | Marinilabiliales | Marinifilaceae | Marinifilum | Marinifilum sp. |
| Z2 | 361 | 10 | 0.02 | 1131707 | NCBI-3-16S@NR_114609.1 | Sphaerochaeta pleomorpha | Bacteria | Spirochaetota | Spirochaetia | Spirochaetales | Sphaerochaetaceae | Sphaerochaeta | Sphaerochaeta pleomorpha |
| Z2 | 362 | 10 | 0.02 | 411467 | NCBI-3-16S@NR_025670.1 | Pseudoflavonifractor capillosus ATCC 29799 | Bacteria | Bacillota | Clostridia | Eubacteriales | Oscillospiraceae | Pseudoflavonifractor | Pseudoflavonifractor capillosus |
| Z2 | 363 | 10 | 0.02 | 328813 | NCBI-3-16S@OR945844.1 | Alistipes onderdonkii | Bacteria | Bacteroidota | Bacteroidia | Bacteroidales | Rikenellaceae | Alistipes | Alistipes onderdonkii |
| Z2 | 364 | 10 | 0.02 | 328812 | NCBI-3-16S@MT902993.1 | Parabacteroides goldsteinii | Bacteria | Bacteroidota | Bacteroidia | Bacteroidales | Tannerellaceae | Parabacteroides | Parabacteroides goldsteinii |
| Z2 | 365 | 10 | 0.02 | 1188752 | NCBI-3-16S@JQ670706.1 | bacterium enrichment culture clone KSE55-29 | Bacteria |  |  |  |  |  | bacterium enrichment culture clone KSE55-29 |
| Z2 | 366 | 10 | 0.02 | 2842482 | NCBI-3-16S@LC635344.1 | Butyricicoccus sp. TL06 | Bacteria | Bacillota | Clostridia | Eubacteriales | Butyricicoccaceae | Butyricicoccus | Butyricicoccus sp. TL06 |
| Z2 | 367 | 10 | 0.02 | 1469948 | NCBI-3-16S@NR_156081.1 | Kineothrix alysoides | Bacteria | Bacillota | Clostridia | Lachnospirales | Lachnospiraceae | Kineothrix | Kineothrix alysoides |
| Z2 | 368 | 10 | 0.02 | 74707 | NCBI-3-16S@MF678906.1 | Staphylococcus schleiferi subsp. schleiferi | Bacteria | Bacillota | Bacilli | Bacillales | Staphylococcaceae | Staphylococcus | Staphylococcus schleiferi |
| Z2 | 369 | 10 | 0.02 | 1501392 | NCBI-3-16S@NR_173687.1 | Coprobacter secundus | Bacteria | Bacteroidota | Bacteroidia | Bacteroidales | Barnesiellaceae | Coprobacter | Coprobacter secundus |
| Z2 | 370 | 10 | 0.02 | 3046382 | NCBI-3-16S@ON706270.1 | Aristaeella hokkaidonensis | Bacteria | Bacillota | Clostridia | Eubacteriales | Aristaeellaceae | Aristaeella | Aristaeella hokkaidonensis |
| Z2 | 371 | 10 | 0.02 | 1151644 | NCBI-3-16S@JN713419.1 | Erysipelotrichaceae bacterium canine oral taxon 255 | Bacteria | Bacillota | Erysipelotrichia | Erysipelotrichales | Erysipelotrichaceae |  | Erysipelotrichaceae bacterium canine oral taxon 255 |
| Z2 | 372 | 10 | 0.02 | 1506 | NCBI-3-16S@OP028895.1 | Clostridium sp. | Bacteria | Bacillota | Clostridia | Eubacteriales | Clostridiaceae | Clostridium | Clostridium sp. |
| Z2 | 373 | 10 | 0.02 | 694434 | NCBI-3-16S@NR_115692.1 | Gracilibacter thermotolerans JW/YJL-S1 | Bacteria | Bacillota | Clostridia | Eubacteriales | Gracilibacteraceae | Gracilibacter | Gracilibacter thermotolerans |
| Z2 | 374 | 10 | 0.02 | 915031 | NCBI-3-16S@HM635217.1 | Clostridia bacterium enrichment culture clone WSC-26 | Bacteria | Bacillota | Clostridia |  |  |  | Clostridia bacterium enrichment culture clone WSC-26 |
| Z2 | 375 | 10 | 0.02 | 1034346 | NCBI-3-16S@MT903170.1 | Dielma fastidiosa | Bacteria | Bacillota | Erysipelotrichia | Erysipelotrichales | Erysipelotrichaceae | Dielma | Dielma fastidiosa |
| Z2 | 376 | 10 | 0.02 | 377137 | NCBI-3-16S@AM236337.1 | spirochete endosymbiont of a lucinid bivalve | Bacteria | Spirochaetota | Spirochaetia | Spirochaetales |  |  | spirochete endosymbiont of a lucinid bivalve |
| Z2 | 377 | 10 | 0.02 | 255723 | NCBI-3-16S@NR_042220.1 | Slackia faecicanis | Bacteria | Actinomycetota | Coriobacteriia | Eggerthellales | Eggerthellaceae | Slackia | Slackia faecicanis |
| Z2 | 378 | 10 | 0.02 | 326368 | NCBI-3-16S@DQ003622.1 | Prevotella genomosp. P8 oral clone MB3_P13 | Bacteria | Bacteroidota | Bacteroidia | Bacteroidales | Prevotellaceae | Prevotella | Prevotella genomosp. P8 oral clone MB3_P13 |
| Z2 | 379 | 10 | 0.02 | 720849 | NCBI-3-16S@GU374060.1 | bacterium enrichment culture clone SRC_DSC3 | Bacteria |  |  |  |  |  | bacterium enrichment culture clone SRC_DSC3 |
| Z2 | 380 | 10 | 0.02 | 89153 | NCBI-3-16S@LC480797.1 | [Clostridium] hylemonae | Bacteria | Bacillota | Clostridia | Lachnospirales | Lachnospiraceae | Lachnoclostridium | [Clostridium] hylemonae |
| Z3 | 1 | 7080 | 21.09 | 1151583 | NCBI-3-16S@JN713550.1 | Spirochaeta sp. canine oral taxon 379 | Bacteria | Spirochaetota | Spirochaetia | Spirochaetales | Spirochaetaceae | Spirochaeta | Spirochaeta sp. canine oral taxon 379 |
| Z3 | 2 | 2792 | 8.32 | 1034346 | NCBI-3-16S@NR_125593.1 | Dielma fastidiosa | Bacteria | Bacillota | Erysipelotrichia | Erysipelotrichales | Erysipelotrichaceae | Dielma | Dielma fastidiosa |
| Z3 | 3 | 1127 | 3.36 | 1965233 | NCBI-3-16S@MK287741.1 | Odoribacter sp. | Bacteria | Bacteroidota | Bacteroidia | Bacteroidales | Odoribacteraceae | Odoribacter | Odoribacter sp. |
| Z3 | 4 | 1107 | 3.30 | 666493 | NCBI-3-16S@GQ377117.1 | bacterium enrichment culture clone DPHB07 | Bacteria |  |  |  |  |  | bacterium enrichment culture clone DPHB07 |
| Z3 | 5 | 1078 | 3.21 | 1879010 | NCBI-3-16S@MW741711.1 | Bacillota bacterium | Bacteria | Bacillota |  |  |  |  | Bacillota bacterium |
| Z3 | 6 | 1003 | 2.99 | 454154 | NCBI-3-16S@NR_113077.1 | Paraprevotella clara | Bacteria | Bacteroidota | Bacteroidia | Bacteroidales | Prevotellaceae | Paraprevotella | Paraprevotella clara |
| Z3 | 7 | 784 | 2.34 | 862466 | NCBI-3-16S@HM231145.1 | Clostridiales bacterium KM2 | Bacteria | Bacillota | Clostridia | Eubacteriales |  |  | Clostridiales bacterium KM2 |
| Z3 | 8 | 598 | 1.78 | 821 | NCBI-3-16S@MT902980.1 | Phocaeicola vulgatus | Bacteria | Bacteroidota | Bacteroidia | Bacteroidales | Bacteroidaceae | Phocaeicola | Phocaeicola vulgatus |
| Z3 | 9 | 562 | 1.67 | 213810 | NCBI-3-16S@NR_102884.1 | Ruminococcus champanellensis 18P13 = JCM 17042 | Bacteria | Bacillota | Clostridia | Eubacteriales | Oscillospiraceae | Ruminococcus | Ruminococcus champanellensis |
| Z3 | 10 | 525 | 1.56 | 820 | NCBI-3-16S@OR945929.1 | Bacteroides uniformis | Bacteria | Bacteroidota | Bacteroidia | Bacteroidales | Bacteroidaceae | Bacteroides | Bacteroides uniformis |
| Z3 | 11 | 459 | 1.37 | 41978 | NCBI-3-16S@MN721375.1 | Ruminococcus sp. | Bacteria | Bacillota | Clostridia | Eubacteriales | Oscillospiraceae | Ruminococcus | Ruminococcus sp. |
| Z3 | 12 | 388 | 1.16 | 1776381 | NCBI-3-16S@NR_146815.1 | Olegusella massiliensis | Bacteria | Actinomycetota | Coriobacteriia | Coriobacteriales | Coriobacteriaceae | Olegusella | Olegusella massiliensis |
| Z3 | 13 | 370 | 1.10 | 29523 | NCBI-3-16S@MK028608.1 | Bacteroides sp. | Bacteria | Bacteroidota | Bacteroidia | Bacteroidales | Bacteroidaceae | Bacteroides | Bacteroides sp. |
| Z3 | 14 | 313 | 0.93 | 2137881 | NCBI-3-16S@MK287767.1 | Coprobacillus sp. | Bacteria | Bacillota | Erysipelotrichia | Erysipelotrichales | Coprobacillaceae | Coprobacillus | Coprobacillus sp. |
| Z3 | 15 | 301 | 0.90 | 40519 | NCBI-3-16S@X85100.1 | Ruminococcus callidus | Bacteria | Bacillota | Clostridia | Eubacteriales | Oscillospiraceae | Ruminococcus | Ruminococcus callidus |
| Z3 | 16 | 291 | 0.87 | 671267 | NCBI-3-16S@NR_113195.1 | Phocaeicola sartorii | Bacteria | Bacteroidota | Bacteroidia | Bacteroidales | Bacteroidaceae | Phocaeicola | Phocaeicola sartorii |
| Z3 | 17 | 285 | 0.85 | 519019 | NCBI-3-16S@EU592966.1 | Atopobium sp. F0209 | Bacteria | Actinomycetota | Coriobacteriia | Coriobacteriales | Atopobiaceae | Atopobium | Atopobium sp. F0209 |
| Z3 | 18 | 282 | 0.84 | 666483 | NCBI-3-16S@GQ377128.1 | bacterium enrichment culture clone DPF25 | Bacteria |  |  |  |  |  | bacterium enrichment culture clone DPF25 |
| Z3 | 19 | 236 | 0.70 | 2364796 | NCBI-3-16S@LS999998.1 | Ruminococcus sp. Marseille-P6503 | Bacteria | Bacillota | Clostridia | Eubacteriales | Oscillospiraceae | Ruminococcus | Ruminococcus sp. Marseille-P6503 |
| Z3 | 20 | 214 | 0.64 | 697329 | NCBI-3-16S@NR_115230.1 | Ruminococcus albus 7 = DSM 20455 | Bacteria | Bacillota | Clostridia | Eubacteriales | Oscillospiraceae | Ruminococcus | Ruminococcus albus |
| Z3 | 21 | 212 | 0.63 | 54199 | NCBI-3-18S@JQ698886.1 | Cyniclomyces guttulatus | Eukaryota | Ascomycota | Saccharomycetes | Saccharomycetales | Saccharomycetaceae | Cyniclomyces | Cyniclomyces guttulatus |
| Z3 | 22 | 205 | 0.61 | 265178 | NCBI-3-16S@NR_146687.1 | Breznakia pachnodae | Bacteria | Bacillota | Erysipelotrichia | Erysipelotrichales | Erysipelotrichaceae | Breznakia | Breznakia pachnodae |
| Z3 | 23 | 196 | 0.58 | 1981510 | NCBI-3-16S@MK615117.1 | Monoglobus pectinilyticus | Bacteria | Bacillota | Clostridia | Monoglobales | Monoglobaceae | Monoglobus | Monoglobus pectinilyticus |
| Z3 | 24 | 192 | 0.57 | 84026 | NCBI-3-16S@MT903092.1 | [Clostridium] methylpentosum | Bacteria | Bacillota | Clostridia | Eubacteriales | Oscillospiraceae |  | [Clostridium] methylpentosum |
| Z3 | 25 | 190 | 0.57 | 393755 | NCBI-3-16S@DQ676999.1 | iron-reducing enrichment clone Cl-A7 | Bacteria |  |  |  |  |  | iron-reducing enrichment clone Cl-A7 |
| Z3 | 26 | 183 | 0.55 | 1917882 | NCBI-3-16S@NR_179582.1 | Gabonibacter timonensis | Bacteria | Bacteroidota | Bacteroidia | Bacteroidales | Porphyromonadaceae | Gabonibacter | Gabonibacter timonensis |
| Z3 | 27 | 180 | 0.54 | 1188760 | NCBI-3-16S@JQ670725.1 | bacterium enrichment culture clone KWE55-24 | Bacteria |  |  |  |  |  | bacterium enrichment culture clone KWE55-24 |
| Z3 | 28 | 176 | 0.52 | 28117 | NCBI-3-16S@OK626603.1 | Alistipes putredinis | Bacteria | Bacteroidota | Bacteroidia | Bacteroidales | Rikenellaceae | Alistipes | Alistipes putredinis |
| Z3 | 29 | 165 | 0.49 | 1172158 | NCBI-3-16S@JQ735967.1 | bacterium enrichment culture clone AK10 | Bacteria |  |  |  |  |  | bacterium enrichment culture clone AK10 |
| Z3 | 30 | 164 | 0.49 | 1329 | NCBI-3-16S@JX876612.1 | Streptococcus canis | Bacteria | Bacillota | Bacilli | Lactobacillales | Streptococcaceae | Streptococcus | Streptococcus canis |
| Z3 | 31 | 154 | 0.46 | 1131707 | NCBI-3-16S@NR_102964.1 | Sphaerochaeta pleomorpha | Bacteria | Spirochaetota | Spirochaetia | Spirochaetales | Sphaerochaetaceae | Sphaerochaeta | Sphaerochaeta pleomorpha |
| Z3 | 32 | 154 | 0.46 | 28111 | NCBI-3-16S@MN537524.1 | Bacteroides eggerthii | Bacteria | Bacteroidota | Bacteroidia | Bacteroidales | Bacteroidaceae | Bacteroides | Bacteroides eggerthii |
| Z3 | 33 | 151 | 0.45 | 28118 | NCBI-3-16S@NR_113075.1 | Odoribacter splanchnicus | Bacteria | Bacteroidota | Bacteroidia | Bacteroidales | Odoribacteraceae | Odoribacter | Odoribacter splanchnicus |
| Z3 | 34 | 146 | 0.43 | 544645 | NCBI-3-16S@KT288267.1 | Butyricimonas virosa | Bacteria | Bacteroidota | Bacteroidia | Bacteroidales | Odoribacteraceae | Butyricimonas | Butyricimonas virosa |
| Z3 | 35 | 143 | 0.43 | 2692629 | NCBI-3-16S@NR_181133.1 | Copranaerobaculum intestinale | Bacteria | Bacillota | Erysipelotrichia | Erysipelotrichales | Erysipelotrichaceae | Copranaerobaculum | Copranaerobaculum intestinale |
| Z3 | 36 | 143 | 0.43 | 2872177 | NCBI-3-16S@OM658611.1 | Odoribacteraceae bacterium | Bacteria | Bacteroidota | Bacteroidia | Bacteroidales | Odoribacteraceae |  | Odoribacteraceae bacterium |
| Z3 | 37 | 141 | 0.42 | 2164149 | NCBI-3-16S@NR_169458.1 | Tepidibaculum saccharolyticum | Bacteria | Bacillota | Clostridia | Eubacteriales | Oscillospiraceae | Tepidibaculum | Tepidibaculum saccharolyticum |
| Z3 | 38 | 134 | 0.40 | 411317 | NCBI-3-16S@EF088328.1 | Clostridium islandicum | Bacteria | Bacillota | Clostridia | Eubacteriales | Clostridiaceae | Clostridium | Clostridium islandicum |
| Z3 | 39 | 124 | 0.37 | 214819 | NCBI-3-16S@AF550610.1 | Lachnospiraceae bacterium 19gly4 | Bacteria | Bacillota | Clostridia | Lachnospirales | Lachnospiraceae |  | Lachnospiraceae bacterium 19gly4 |
| Z3 | 40 | 115 | 0.34 | 2725562 | NCBI-3-16S@NR_181379.1 | Caecibacteroides pullorum | Bacteria | Bacteroidota | Bacteroidia | Bacteroidales | Bacteroidaceae | Caecibacteroides | Caecibacteroides pullorum |
| Z3 | 41 | 114 | 0.34 | 357276 | NCBI-3-16S@OR125614.1 | Phocaeicola dorei | Bacteria | Bacteroidota | Bacteroidia | Bacteroidales | Bacteroidaceae | Phocaeicola | Phocaeicola dorei |
| Z3 | 42 | 109 | 0.32 | 1898203 | NCBI-3-16S@MH699316.1 | Lachnospiraceae bacterium | Bacteria | Bacillota | Clostridia | Lachnospirales | Lachnospiraceae |  | Lachnospiraceae bacterium |
| Z3 | 43 | 101 | 0.30 | 1411144 | NCBI-3-16S@AB861982.1 | Bacteroides caecigallinarum | Bacteria | Bacteroidota | Bacteroidia | Bacteroidales | Bacteroidaceae | Bacteroides | Bacteroides caecigallinarum |
| Z3 | 44 | 100 | 0.30 | 2779355 | NCBI-3-16S@NR_180617.1 | Ructibacterium gallinarum | Bacteria | Bacillota | Clostridia | Eubacteriales | Oscillospiraceae | Ructibacterium | Ructibacterium gallinarum |
| Z3 | 45 | 97 | 0.29 | 55507 | NCBI-3-16S@Y09434.1 | Schwartzia succinivorans | Bacteria | Bacillota | Negativicutes | Selenomonadales | Selenomonadaceae | Schwartzia | Schwartzia succinivorans |
| Z3 | 46 | 97 | 0.29 | 258132 | NCBI-3-16S@AY466715.1 | Clostridiales bacterium NS5-2 | Bacteria | Bacillota | Clostridia | Eubacteriales |  |  | Clostridiales bacterium NS5-2 |
| Z3 | 47 | 95 | 0.28 | 283734 | NCBI-3-16S@ON497037.1 | Staphylococcus pseudintermedius | Bacteria | Bacillota | Bacilli | Bacillales | Staphylococcaceae | Staphylococcus | Staphylococcus pseudintermedius |
| Z3 | 48 | 94 | 0.28 | 502558 | NCBI-3-16S@AB379693.1 | Eggerthella sp. YY7918 | Bacteria | Actinomycetota | Coriobacteriia | Eggerthellales | Eggerthellaceae | Eggerthella | Eggerthella sp. YY7918 |
| Z3 | 49 | 91 | 0.27 | 2986072 | NCBI-3-16S@OX352005.1 | Candidatus Minimicrobia sp. IHU4 | Bacteria | Candidatus Saccharibacteria |  |  |  | Candidatus Minimicrobia | Candidatus Minimicrobia sp. IHU4 |
| Z3 | 50 | 87 | 0.26 | 9906 | NCBI-3-18S@XR_009732643.1 | Bos javanicus | Eukaryota | Chordata | Mammalia | Artiodactyla | Bovidae | Bos | Bos javanicus |
| Z3 | 51 | 87 | 0.26 | 2763676 | NCBI-3-16S@MT905150.1 | Qingrenia yutianensis | Bacteria | Bacillota | Clostridia | Eubacteriales | Oscillospiraceae | Qingrenia | Qingrenia yutianensis |
| Z3 | 52 | 81 | 0.24 | 2899121 | NCBI-3-16S@NR_179945.1 | Tannockella kyphosi | Bacteria | Bacillota | Erysipelotrichia | Erysipelotrichales | Coprobacillaceae | Tannockella | Tannockella kyphosi |
| Z3 | 53 | 81 | 0.24 | 818 | NCBI-3-16S@LR999624.1 | Bacteroides thetaiotaomicron | Bacteria | Bacteroidota | Bacteroidia | Bacteroidales | Bacteroidaceae | Bacteroides | Bacteroides thetaiotaomicron |
| Z3 | 54 | 74 | 0.22 | 1330740 | NCBI-3-16S@KC853480.1 | bacterium enrichment culture clone aHCH2_E12 | Bacteria |  |  |  |  |  | bacterium enrichment culture clone aHCH2_E12 |
| Z3 | 55 | 71 | 0.21 | 31971 | NCBI-3-16S@NR_113409.1 | Amedibacillus dolichus | Bacteria | Bacillota | Erysipelotrichia | Erysipelotrichales | Erysipelotrichaceae | Amedibacillus | Amedibacillus dolichus |
| Z3 | 56 | 69 | 0.21 | 1954376 | NCBI-3-16S@MZ310618.1 | Caproiciproducens sp. | Bacteria | Bacillota | Clostridia | Eubacteriales | Acutalibacteraceae | Caproiciproducens | Caproiciproducens sp. |
| Z3 | 57 | 68 | 0.20 | 742727 | NCBI-3-16S@NR_113070.1 | Bacteroides oleiciplenus YIT 12058 | Bacteria | Bacteroidota | Bacteroidia | Bacteroidales | Bacteroidaceae | Bacteroides | Bacteroides oleiciplenus |
| Z3 | 58 | 67 | 0.20 | 2894156 | NCBI-3-16S@NR_184626.1 | Leptogranulimonas caecicola | Bacteria | Actinomycetota | Coriobacteriia | Coriobacteriales | Kribbibacteriaceae | Leptogranulimonas | Leptogranulimonas caecicola |
| Z3 | 59 | 66 | 0.20 | 1352374 | NCBI-3-16S@KF156793.1 | Ruminococcus sp. YE78 | Bacteria | Bacillota | Clostridia | Eubacteriales | Oscillospiraceae | Ruminococcus | Ruminococcus sp. YE78 |
| Z3 | 60 | 65 | 0.19 | 1030127 | NCBI-3-16S@JF813174.1 | Bacteroides sp. dnLKV2 | Bacteria | Bacteroidota | Bacteroidia | Bacteroidales | Bacteroidaceae | Bacteroides | Bacteroides sp. dnLKV2 |
| Z3 | 61 | 63 | 0.19 | 1264 | NCBI-3-16S@NR_115230.1 | Ruminococcus albus | Bacteria | Bacillota | Clostridia | Eubacteriales | Oscillospiraceae | Ruminococcus | Ruminococcus albus |
| Z3 | 62 | 63 | 0.19 | 1297424 | NCBI-3-16S@NR_125464.1 | Anaerobacterium chartisolvens | Bacteria | Bacillota | Clostridia | Eubacteriales | Oscillospiraceae | Anaerobacterium | Anaerobacterium chartisolvens |
| Z3 | 63 | 61 | 0.18 | 39490 | NCBI-3-16S@KT221541.1 | Eubacterium ramulus | Bacteria | Bacillota | Clostridia | Eubacteriales | Eubacteriaceae | Eubacterium | Eubacterium ramulus |
| Z3 | 64 | 61 | 0.18 | 2939460 | NCBI-3-16S@OP389241.1 | Parvivirga hydrogeniphila | Bacteria | Actinomycetota | Coriobacteriia | Anaerosomatales | Anaerosomataceae | Parvivirga | Parvivirga hydrogeniphila |
| Z3 | 65 | 59 | 0.18 | 1295 | NCBI-3-16S@MF678906.1 | Staphylococcus schleiferi | Bacteria | Bacillota | Bacilli | Bacillales | Staphylococcaceae | Staphylococcus | Staphylococcus schleiferi |
| Z3 | 66 | 58 | 0.17 | 39494 | NCBI-3-16S@NR_044648.2 | Absiella tortuosum | Bacteria | Bacillota | Erysipelotrichia | Erysipelotrichales | Erysipelotrichaceae | Amedibacterium | Absiella tortuosum |
| Z3 | 67 | 57 | 0.17 | 544645 | NCBI-3-16S@MT902988.1 | Butyricimonas virosa | Bacteria | Bacteroidota | Bacteroidia | Bacteroidales | Odoribacteraceae | Butyricimonas | Butyricimonas virosa |
| Z3 | 68 | 56 | 0.17 | 1034346 | NCBI-3-16S@MT903170.1 | Dielma fastidiosa | Bacteria | Bacillota | Erysipelotrichia | Erysipelotrichales | Erysipelotrichaceae | Dielma | Dielma fastidiosa |
| Z3 | 69 | 54 | 0.16 | 2606626 | NCBI-3-16S@NR_180830.1 | Sodaliphilus pleomorphus | Bacteria | Bacteroidota | Bacteroidia | Bacteroidales | Muribaculaceae | Sodaliphilus | Sodaliphilus pleomorphus |
| Z3 | 70 | 52 | 0.15 | 762968 | NCBI-3-16S@NR_041626.1 | Paraprevotella clara YIT 11840 | Bacteria | Bacteroidota | Bacteroidia | Bacteroidales | Prevotellaceae | Paraprevotella | Paraprevotella clara |
| Z3 | 71 | 52 | 0.15 | 720554 | NCBI-3-16S@NR_102987.1 | Acetivibrio clariflavus DSM 19732 | Bacteria | Bacillota | Clostridia | Eubacteriales | Oscillospiraceae | Acetivibrio | Acetivibrio clariflavus |
| Z3 | 72 | 52 | 0.15 | 1945594 | NCBI-3-16S@MW682303.1 | Sporobacter sp. | Bacteria | Bacillota | Clostridia | Eubacteriales | Oscillospiraceae | Sporobacter | Sporobacter sp. |
| Z3 | 73 | 51 | 0.15 | 2485925 | NCBI-3-16S@MH699341.1 | Oscillospiraceae bacterium | Bacteria | Bacillota | Clostridia | Eubacteriales | Oscillospiraceae |  | Oscillospiraceae bacterium |
| Z3 | 74 | 48 | 0.14 | 2897707 | NCBI-3-16S@NR_144748.1 | Merdimmobilis hominis | Bacteria | Bacillota | Clostridia | Eubacteriales | Oscillospiraceae | Merdimmobilis | Merdimmobilis hominis |
| Z3 | 75 | 47 | 0.14 | 544645 | NCBI-3-16S@NR_041691.1 | Butyricimonas virosa | Bacteria | Bacteroidota | Bacteroidia | Bacteroidales | Odoribacteraceae | Butyricimonas | Butyricimonas virosa |
| Z3 | 76 | 47 | 0.14 | 142586 | NCBI-3-16S@OK272455.1 | Eubacterium sp. | Bacteria | Bacillota | Clostridia | Eubacteriales | Eubacteriaceae | Eubacterium | Eubacterium sp. |
| Z3 | 77 | 46 | 0.14 | 2086579 | NCBI-3-16S@LT985388.1 | Bacteroides sp. Marseille-P3684 | Bacteria | Bacteroidota | Bacteroidia | Bacteroidales | Bacteroidaceae | Bacteroides | Bacteroides sp. Marseille-P3684 |
| Z3 | 78 | 46 | 0.14 | 2049040 | NCBI-3-16S@MK287624.1 | Roseburia sp. | Bacteria | Bacillota | Clostridia | Lachnospirales | Lachnospiraceae | Roseburia | Roseburia sp. |
| Z3 | 79 | 46 | 0.14 | 1929886 | NCBI-3-16S@MK287687.1 | Eggerthella sp. | Bacteria | Actinomycetota | Coriobacteriia | Eggerthellales | Eggerthellaceae | Eggerthella | Eggerthella sp. |
| Z3 | 80 | 44 | 0.13 | 76124 | NCBI-3-16S@U13037.1 | [Eubacterium] minutum | Bacteria | Bacillota | Clostridia | Eubacteriales | Eubacteriales Family XIII. Incertae Sedis |  | [Eubacterium] minutum |
| Z3 | 81 | 44 | 0.13 | 399361 | NCBI-3-16S@DQ833401.1 | Sphaerochaeta sp. RCcp2 | Bacteria | Spirochaetota | Spirochaetia | Spirochaetales | Sphaerochaetaceae | Sphaerochaeta | Sphaerochaeta sp. RCcp2 |
| Z3 | 82 | 44 | 0.13 | 1917883 | NCBI-3-16S@NR_179583.1 | Bacteroides togonis | Bacteria | Bacteroidota | Bacteroidia | Bacteroidales | Bacteroidaceae | Bacteroides | Bacteroides togonis |
| Z3 | 83 | 44 | 0.13 | 1972561 | NCBI-3-16S@OM368626.1 | Eggerthellaceae bacterium | Bacteria | Actinomycetota | Coriobacteriia | Eggerthellales | Eggerthellaceae |  | Eggerthellaceae bacterium |
| Z3 | 84 | 43 | 0.13 | 329854 | NCBI-3-16S@NR_041307.1 | Bacteroides intestinalis | Bacteria | Bacteroidota | Bacteroidia | Bacteroidales | Bacteroidaceae | Bacteroides | Bacteroides intestinalis |
| Z3 | 85 | 43 | 0.13 | 394503 | NCBI-3-16S@OK626620.1 | Ruminiclostridium cellulolyticum H10 | Bacteria | Bacillota | Clostridia | Eubacteriales | Oscillospiraceae | Ruminiclostridium | Ruminiclostridium cellulolyticum |
| Z3 | 86 | 43 | 0.13 | 2841509 | NCBI-3-16S@NR_181761.1 | Butyricicoccus intestinisimiae | Bacteria | Bacillota | Clostridia | Eubacteriales | Butyricicoccaceae | Butyricicoccus | Butyricicoccus intestinisimiae |
| Z3 | 87 | 42 | 0.13 | 454155 | NCBI-3-16S@NR_113078.1 | Paraprevotella xylaniphila | Bacteria | Bacteroidota | Bacteroidia | Bacteroidales | Prevotellaceae | Paraprevotella | Paraprevotella xylaniphila |
| Z3 | 88 | 42 | 0.13 | 1236515 | NCBI-3-16S@NR_113195.1 | Phocaeicola sartorii JCM 17136 = DSM 21941 | Bacteria | Bacteroidota | Bacteroidia | Bacteroidales | Bacteroidaceae | Phocaeicola | Phocaeicola sartorii |
| Z3 | 89 | 41 | 0.12 | 1796610 | NCBI-3-16S@OM658549.1 | Adlercreutzia muris | Bacteria | Actinomycetota | Coriobacteriia | Eggerthellales | Eggerthellaceae | Adlercreutzia | Adlercreutzia muris |
| Z3 | 90 | 40 | 0.12 | 1872387 | NCBI-3-16S@PP065737.1 | Adlercreutzia sp. | Bacteria | Actinomycetota | Coriobacteriia | Eggerthellales | Eggerthellaceae | Adlercreutzia | Adlercreutzia sp. |
| Z3 | 91 | 40 | 0.12 | 1792311 | NCBI-3-16S@NR_178871.1 | Petroclostridium xylanilyticum | Bacteria | Bacillota | Clostridia | Eubacteriales | Oscillospiraceae | Petroclostridium | Petroclostridium xylanilyticum |
| Z3 | 92 | 39 | 0.12 | 1236512 | NCBI-3-16S@NR_113072.1 | Bacteroides rodentium JCM 16496 | Bacteria | Bacteroidota | Bacteroidia | Bacteroidales | Bacteroidaceae | Bacteroides | Bacteroides rodentium |
| Z3 | 93 | 39 | 0.12 | 1870985 | NCBI-3-16S@NR_179544.1 | Arabiibacter massiliensis | Bacteria | Actinomycetota | Coriobacteriia | Eggerthellales | Eggerthellaceae | Arabiibacter | Arabiibacter massiliensis |
| Z3 | 94 | 39 | 0.12 | 1872387 | NCBI-3-16S@MK287689.1 | Adlercreutzia sp. | Bacteria | Actinomycetota | Coriobacteriia | Eggerthellales | Eggerthellaceae | Adlercreutzia | Adlercreutzia sp. |
| Z3 | 95 | 39 | 0.12 | 2834112 | NCBI-3-16S@NR_181731.1 | Bacteroides propionicigenes | Bacteria | Bacteroidota | Bacteroidia | Bacteroidales | Bacteroidaceae | Bacteroides | Bacteroides propionicigenes |
| Z3 | 96 | 38 | 0.11 | 1515 | NCBI-3-16S@NR_113157.1 | Acetivibrio thermocellus | Bacteria | Bacillota | Clostridia | Eubacteriales | Oscillospiraceae | Acetivibrio | Acetivibrio thermocellus |
| Z3 | 97 | 38 | 0.11 | 1852385 | NCBI-3-16S@NR_173693.1 | Olsenella phocaeensis | Bacteria | Actinomycetota | Coriobacteriia | Coriobacteriales | Atopobiaceae | Olsenella | Olsenella phocaeensis |
| Z3 | 98 | 38 | 0.11 | 1852363 | NCBI-3-16S@LT576387.1 | Clostridiales bacterium Marseille-P2846 | Bacteria | Bacillota | Clostridia | Eubacteriales | Beduinellaceae | Beduinella | Beduinella massiliensis |
| Z3 | 99 | 37 | 0.11 | 1151617 | NCBI-3-16S@JN713189.1 | Clostridiales bacterium canine oral taxon 027 | Bacteria | Bacillota | Clostridia | Eubacteriales |  |  | Clostridiales bacterium canine oral taxon 027 |
| Z3 | 100 | 36 | 0.11 | 1556 | NCBI-3-16S@NR_117601.1 | Gottschalkia acidurici | Bacteria | Bacillota | Tissierellia | Tissierellales | Gottschalkiaceae | Gottschalkia | Gottschalkia acidurici |
| Z3 | 101 | 36 | 0.11 | 1588753 | NCBI-3-16S@KP192306.1 | Coriobacteriales bacterium DNF00809 | Bacteria | Actinomycetota | Coriobacteriia | Coriobacteriales |  |  | Coriobacteriales bacterium DNF00809 |
| Z3 | 102 | 36 | 0.11 | 1926877 | NCBI-3-16S@MG551268.2 | Proteiniphilum sp. | Bacteria | Bacteroidota | Bacteroidia | Bacteroidales | Dysgonomonadaceae | Proteiniphilum | Proteiniphilum sp. |
| Z3 | 103 | 34 | 0.10 | 1898206 | NCBI-3-16S@OQ150036.1 | Spirochaetaceae bacterium | Bacteria | Spirochaetota | Spirochaetia | Spirochaetales | Spirochaetaceae |  | Spirochaetaceae bacterium |
| Z3 | 104 | 34 | 0.10 | 925962 | NCBI-3-16S@AB599946.1 | Bacteroides sp. SLC1-38 | Bacteria | Bacteroidota | Bacteroidia | Bacteroidales | Bacteroidaceae | Bacteroides | Bacteroides sp. SLC1-38 |
| Z3 | 105 | 34 | 0.10 | 1972642 | NCBI-3-16S@MW599793.1 | Sphaerochaeta sp. | Bacteria | Spirochaetota | Spirochaetia | Spirochaetales | Sphaerochaetaceae | Sphaerochaeta | Sphaerochaeta sp. |
| Z3 | 106 | 33 | 0.10 | 1872444 | NCBI-3-16S@MN611110.1 | Alistipes sp. | Bacteria | Bacteroidota | Bacteroidia | Bacteroidales | Rikenellaceae | Alistipes | Alistipes sp. |
| Z3 | 107 | 33 | 0.10 | 2687240 | NCBI-3-16S@NR_165703.1 | Zunongwangia flava | Bacteria | Bacteroidota | Flavobacteriia | Flavobacteriales | Flavobacteriaceae | Zunongwangia | Zunongwangia flava |
| Z3 | 108 | 33 | 0.10 | 1841857 | NCBI-3-16S@NR_144745.1 | Culturomica massiliensis | Bacteria | Bacteroidota | Bacteroidia | Bacteroidales | Odoribacteraceae | Culturomica | Culturomica massiliensis |
| Z3 | 109 | 32 | 0.10 | 1969738 | NCBI-3-16S@LC259309.1 | Butyricimonas sp. | Bacteria | Bacteroidota | Bacteroidia | Bacteroidales | Odoribacteraceae | Butyricimonas | Butyricimonas sp. |
| Z3 | 110 | 32 | 0.10 | 46506 | NCBI-3-16S@OR554138.1 | Bacteroides stercoris | Bacteria | Bacteroidota | Bacteroidia | Bacteroidales | Bacteroidaceae | Bacteroides | Bacteroides stercoris |
| Z3 | 111 | 31 | 0.09 | 1872387 | NCBI-3-16S@MT052692.1 | Adlercreutzia sp. | Bacteria | Actinomycetota | Coriobacteriia | Eggerthellales | Eggerthellaceae | Adlercreutzia | Adlercreutzia sp. |
| Z3 | 112 | 31 | 0.09 | 1796646 | NCBI-3-16S@OK626632.1 | Muribaculum intestinale | Bacteria | Bacteroidota | Bacteroidia | Bacteroidales | Muribaculaceae | Muribaculum | Muribaculum intestinale |
| Z3 | 113 | 31 | 0.09 | 2049040 | NCBI-3-16S@MZ310605.1 | Roseburia sp. | Bacteria | Bacillota | Clostridia | Lachnospirales | Lachnospiraceae | Roseburia | Roseburia sp. |
| Z3 | 114 | 30 | 0.09 | 2086584 | NCBI-3-16S@NR_179658.1 | Massilistercora timonensis | Bacteria | Bacillota | Clostridia | Eubacteriales |  | Massilistercora | Massilistercora timonensis |
| Z3 | 115 | 29 | 0.09 | 545496 | NCBI-3-16S@EU815223.1 | Ruminococcus sp. NML 00-0124 | Bacteria | Bacillota | Clostridia | Eubacteriales | Oscillospiraceae | Ruminococcus | Ruminococcus sp. NML 00-0124 |
| Z3 | 116 | 29 | 0.09 | 298183 | NCBI-3-16S@AY756145.2 | anaerobic bacterium Glu3 | Bacteria | Bacillota | Clostridia | Eubacteriales |  |  | anaerobic bacterium Glu3 |
| Z3 | 117 | 29 | 0.09 | 1156936 | NCBI-3-16S@JQ404436.1 | Clostridium sp. WSC-9-7 | Bacteria | Bacillota | Clostridia | Eubacteriales | Clostridiaceae | Clostridium | Clostridium sp. WSC-9-7 |
| Z3 | 118 | 29 | 0.09 | 1903262 | NCBI-3-16S@NR_179564.1 | Bacteroides ndongoniae | Bacteria | Bacteroidota | Bacteroidia | Bacteroidales | Bacteroidaceae | Bacteroides | Bacteroides ndongoniae |
| Z3 | 119 | 28 | 0.08 | 569511 | NCBI-3-16S@FJ269048.1 | iron-reducing bacterium enrichment culture clone HN7 | Bacteria |  |  |  |  |  | iron-reducing bacterium enrichment culture clone HN7 |
| Z3 | 120 | 28 | 0.08 | 747645 | NCBI-3-16S@NR_117374.1 | Parvibacter caecicola | Bacteria | Actinomycetota | Coriobacteriia | Coriobacteriales | Coriobacteriaceae | Parvibacter | Parvibacter caecicola |
| Z3 | 121 | 27 | 0.08 | 1869337 | NCBI-3-16S@OQ353078.1 | Parabacteroides sp. | Bacteria | Bacteroidota | Bacteroidia | Bacteroidales | Tannerellaceae | Parabacteroides | Parabacteroides sp. |
| Z3 | 122 | 27 | 0.08 | 2606638 | NCBI-3-16S@NR_180814.1 | Bullifex porci | Bacteria | Spirochaetota | Spirochaetia | Spirochaetales | Spirochaetaceae | Bullifex | Bullifex porci |
| Z3 | 123 | 26 | 0.08 | 1585974 | NCBI-3-16S@NR_144718.1 | Beduini massiliensis | Bacteria | Bacillota | Erysipelotrichia | Erysipelotrichales | Erysipelotrichaceae | Beduini | Beduini massiliensis |
| Z3 | 124 | 26 | 0.08 | 2039241 | NCBI-3-16S@MK287717.1 | Anaerotignum sp. | Bacteria | Bacillota | Clostridia | Lachnospirales | Anaerotignaceae | Anaerotignum | Anaerotignum sp. |
| Z3 | 125 | 26 | 0.08 | 630799 | NCBI-3-16S@FJ799136.1 | bacterium enrichment culture clone EtOH-23 | Bacteria |  |  |  |  |  | bacterium enrichment culture clone EtOH-23 |
| Z3 | 126 | 26 | 0.08 | 1870988 | NCBI-3-16S@OK626623.1 | Pseudoflavonifractor phocaeensis | Bacteria | Bacillota | Clostridia | Eubacteriales | Oscillospiraceae | Pseudoflavonifractor | Pseudoflavonifractor phocaeensis |
| Z3 | 127 | 26 | 0.08 | 28113 | NCBI-3-16S@JN713478.1 | Bacteroides heparinolyticus | Bacteria | Bacteroidota | Bacteroidia | Bacteroidales | Bacteroidaceae | Bacteroides | Bacteroides heparinolyticus |
| Z3 | 128 | 25 | 0.07 | 1159221 | NCBI-3-16S@AB702935.1 | Clostridiales bacterium CIEAF 013 | Bacteria | Bacillota | Clostridia | Eubacteriales |  |  | Clostridiales bacterium CIEAF 013 |
| Z3 | 129 | 25 | 0.07 | 1151542 | NCBI-3-16S@JN713451.1 | Prevotella sp. canine oral taxon 284 | Bacteria | Bacteroidota | Bacteroidia | Bacteroidales | Prevotellaceae | Prevotella | Prevotella sp. canine oral taxon 284 |
| Z3 | 130 | 25 | 0.07 | 2850323 | NCBI-3-16S@NR_181407.1 | Diplocloster agilis | Bacteria | Bacillota | Clostridia | Lachnospirales | Lachnospiraceae | Diplocloster | Diplocloster agilis |
| Z3 | 131 | 25 | 0.07 | 2049044 | NCBI-3-16S@OK510351.1 | Erysipelotrichaceae bacterium | Bacteria | Bacillota | Erysipelotrichia | Erysipelotrichales | Erysipelotrichaceae |  | Erysipelotrichaceae bacterium |
| Z3 | 132 | 25 | 0.07 | 393757 | NCBI-3-16S@DQ677001.1 | iron-reducing enrichment clone Cl-A9 | Bacteria |  |  |  |  |  | iron-reducing enrichment clone Cl-A9 |
| Z3 | 133 | 25 | 0.07 | 2661914 | NCBI-3-16S@MN604226.1 | Traorella sp. mt218 | Bacteria | Bacillota | Erysipelotrichia | Erysipelotrichales | Erysipelotrichaceae | Traorella | Traorella sp. mt218 |
| Z3 | 134 | 25 | 0.07 | 2485926 | NCBI-3-16S@OK510363.1 | Atopobiaceae bacterium | Bacteria | Actinomycetota | Coriobacteriia | Coriobacteriales | Atopobiaceae |  | Atopobiaceae bacterium |
| Z3 | 135 | 24 | 0.07 | 2058290 | NCBI-3-16S@LT970864.1 | Eubacterium sp. Marseille-P5640 | Bacteria | Bacillota | Erysipelotrichia | Erysipelotrichales | Erysipelotrichaceae | Amedibacterium | Amedibacterium intestinale |
| Z3 | 136 | 24 | 0.07 | 1577240 | NCBI-3-16S@KM462152.1 | Bacteroides sp. feline oral taxon 308 | Bacteria | Bacteroidota | Bacteroidia | Bacteroidales | Bacteroidaceae | Bacteroides | Bacteroides sp. feline oral taxon 308 |
| Z3 | 137 | 23 | 0.07 | 1647716 | NCBI-3-16S@KR232852.1 | Porphyromonadaceae bacterium S190 | Bacteria | Bacteroidota | Bacteroidia | Bacteroidales | Porphyromonadaceae |  | Porphyromonadaceae bacterium S190 |
| Z3 | 138 | 23 | 0.07 | 871324 | NCBI-3-16S@AB574479.1 | Bacteroides stercorirosoris | Bacteria | Bacteroidota | Bacteroidia | Bacteroidales | Bacteroidaceae | Bacteroides | Bacteroides stercorirosoris |
| Z3 | 139 | 22 | 0.07 | 1972642 | NCBI-3-16S@MG696667.1 | Sphaerochaeta sp. | Bacteria | Spirochaetota | Spirochaetia | Spirochaetales | Sphaerochaetaceae | Sphaerochaeta | Sphaerochaeta sp. |
| Z3 | 140 | 22 | 0.07 | 537365 | NCBI-3-16S@EU728720.1 | Bacteroidaceae bacterium DJF_B220 | Bacteria | Bacteroidota | Bacteroidia | Bacteroidales | Bacteroidaceae |  | Bacteroidaceae bacterium DJF_B220 |
| Z3 | 141 | 22 | 0.07 | 889071 | NCBI-3-16S@HQ222293.1 | Clostridium sp. enrichment culture clone VanCtr97 | Bacteria | Bacillota | Clostridia | Eubacteriales | Clostridiaceae | Clostridium | Clostridium sp. enrichment culture clone VanCtr97 |
| Z3 | 142 | 22 | 0.07 | 1522 | NCBI-3-16S@MT903089.1 | [Clostridium] innocuum | Bacteria | Bacillota | Erysipelotrichia | Erysipelotrichales | Erysipelotrichaceae |  | [Clostridium] innocuum |
| Z3 | 143 | 21 | 0.06 | 823 | NCBI-3-16S@MT902991.1 | Parabacteroides distasonis | Bacteria | Bacteroidota | Bacteroidia | Bacteroidales | Tannerellaceae | Parabacteroides | Parabacteroides distasonis |
| Z3 | 144 | 21 | 0.06 | 569483 | NCBI-3-16S@FJ269072.1 | iron-reducing bacterium enrichment culture clone HN109 | Bacteria |  |  |  |  |  | iron-reducing bacterium enrichment culture clone HN109 |
| Z3 | 145 | 21 | 0.06 | 649764 | NCBI-3-16S@NR_024952.1 | Slackia exigua ATCC 700122 | Bacteria | Actinomycetota | Coriobacteriia | Eggerthellales | Eggerthellaceae | Slackia | Slackia exigua |
| Z3 | 146 | 20 | 0.06 | 376804 | NCBI-3-16S@NR_041446.1 | Phocaeicola barnesiae | Bacteria | Bacteroidota | Bacteroidia | Bacteroidales | Bacteroidaceae | Phocaeicola | Phocaeicola barnesiae |
| Z3 | 147 | 20 | 0.06 | 3046383 | NCBI-3-16S@ON706274.1 | Aristaeella lactis | Bacteria | Bacillota | Clostridia | Eubacteriales | Aristaeellaceae | Aristaeella | Aristaeella lactis |
| Z3 | 148 | 20 | 0.06 | 42458 | NCBI-3-18S@HM590655.1 | Lichtheimia corymbifera | Eukaryota | Mucoromycota | Mucoromycetes | Mucorales | Lichtheimiaceae | Lichtheimia | Lichtheimia corymbifera |
| Z3 | 149 | 19 | 0.06 | 397286 | NCBI-3-16S@DQ789124.1 | Lachnospiraceae bacterium 14-2 | Bacteria | Bacillota | Clostridia | Lachnospirales | Lachnospiraceae |  | Lachnospiraceae bacterium 14-2 |
| Z3 | 150 | 19 | 0.06 | 377137 | NCBI-3-16S@AM236337.1 | spirochete endosymbiont of a lucinid bivalve | Bacteria | Spirochaetota | Spirochaetia | Spirochaetales |  |  | spirochete endosymbiont of a lucinid bivalve |
| Z3 | 151 | 19 | 0.06 | 1156034 | NCBI-3-16S@AB700365.1 | Lachnospiraceae bacterium 607 | Bacteria | Bacillota | Clostridia | Lachnospirales | Lachnospiraceae |  | Lachnospiraceae bacterium 607 |
| Z3 | 152 | 19 | 0.06 | 1841865 | NCBI-3-16S@NR_144747.1 | Mediterranea massiliensis | Bacteria | Bacteroidota | Bacteroidia | Bacteroidales | Bacteroidaceae | Mediterranea | Mediterranea massiliensis |
| Z3 | 153 | 19 | 0.06 | 100176 | NCBI-3-16S@NR_025025.1 | Papillibacter cinnamivorans | Bacteria | Bacillota | Clostridia | Eubacteriales | Oscillospiraceae | Papillibacter | Papillibacter cinnamivorans |
| Z3 | 154 | 19 | 0.06 | 2026735 | NCBI-3-16S@OQ808222.1 | Deltaproteobacteria bacterium | Bacteria | Myxococcota | Myxococcia |  |  |  | Deltaproteobacteria bacterium |
| Z3 | 155 | 19 | 0.06 | 2584469 | NCBI-3-16S@NR_180580.1 | Olsenella lakotia | Bacteria | Actinomycetota | Coriobacteriia | Coriobacteriales | Atopobiaceae | Olsenella | Olsenella lakotia |
| Z3 | 156 | 19 | 0.06 | 626929 | NCBI-3-16S@OQ645443.1 | Bacteroides clarus | Bacteria | Bacteroidota | Bacteroidia | Bacteroidales | Bacteroidaceae | Bacteroides | Bacteroides clarus |
| Z3 | 157 | 19 | 0.06 | 371601 | NCBI-3-16S@OP510057.1 | Bacteroides xylanisolvens | Bacteria | Bacteroidota | Bacteroidia | Bacteroidales | Bacteroidaceae | Bacteroides | Bacteroides xylanisolvens |
| Z3 | 158 | 18 | 0.05 | 747602 | NCBI-3-16S@AB551425.1 | Clostridium sp. TG60-81 | Bacteria | Bacillota | Clostridia | Eubacteriales | Clostridiaceae | Clostridium | Clostridium sp. TG60-81 |
| Z3 | 159 | 18 | 0.05 | 1155412 | NCBI-3-16S@JN688035.1 | bacterium enrichment culture clone M153 | Bacteria |  |  |  |  |  | bacterium enrichment culture clone M153 |
| Z3 | 160 | 18 | 0.05 | 2751153 | NCBI-3-16S@NR_173687.1 | Coprobacter secundus subsp. similis | Bacteria | Bacteroidota | Bacteroidia | Bacteroidales | Barnesiellaceae | Coprobacter | Coprobacter secundus |
| Z3 | 161 | 18 | 0.05 | 2518971 | NCBI-3-16S@NR_170508.1 | Duncaniella dubosii | Bacteria | Bacteroidota | Bacteroidia | Bacteroidales | Muribaculaceae | Duncaniella | Duncaniella dubosii |
| Z3 | 162 | 18 | 0.05 | 1335613 | NCBI-3-16S@MK544835.1 | Gordonibacter urolithinfaciens | Bacteria | Actinomycetota | Coriobacteriia | Eggerthellales | Eggerthellaceae | Gordonibacter | Gordonibacter urolithinfaciens |
| Z3 | 163 | 18 | 0.05 | 1870991 | NCBI-3-16S@NR_179547.1 | Massilioclostridium coli | Bacteria | Bacillota | Clostridia | Eubacteriales | Clostridiaceae | Massilioclostridium | Massilioclostridium coli |
| Z3 | 164 | 18 | 0.05 | 399354 | NCBI-3-16S@DQ833395.1 | Sphaerochaeta sp. 'Grapes TMA14' | Bacteria | Spirochaetota | Spirochaetia | Spirochaetales | Sphaerochaetaceae | Sphaerochaeta | Sphaerochaeta sp. 'Grapes TMA14' |
| Z3 | 165 | 18 | 0.05 | 2585119 | NCBI-3-16S@NR_179296.1 | Alistipes dispar | Bacteria | Bacteroidota | Bacteroidia | Bacteroidales | Rikenellaceae | Alistipes | Alistipes dispar |
| Z3 | 166 | 17 | 0.05 | 2937417 | NCBI-3-16S@ON361133.1 | Bacteroides muris (ex Fokt et al. 2023) | Bacteria | Bacteroidota | Bacteroidia | Bacteroidales | Bacteroidaceae | Bacteroides | Bacteroides muris (ex Fokt et al. 2023) |
| Z3 | 167 | 17 | 0.05 | 1872387 | NCBI-3-16S@MK287694.1 | Adlercreutzia sp. | Bacteria | Actinomycetota | Coriobacteriia | Eggerthellales | Eggerthellaceae | Adlercreutzia | Adlercreutzia sp. |
| Z3 | 168 | 17 | 0.05 | 1628085 | NCBI-3-16S@NR_151982.1 | Agathobaculum butyriciproducens | Bacteria | Bacillota | Clostridia | Eubacteriales | Butyricicoccaceae | Agathobaculum | Agathobaculum butyriciproducens |
| Z3 | 169 | 17 | 0.05 | 569479 | NCBI-3-16S@FJ269050.1 | iron-reducing bacterium enrichment culture clone HN10 | Bacteria |  |  |  |  |  | iron-reducing bacterium enrichment culture clone HN10 |
| Z3 | 170 | 17 | 0.05 | 361365 | NCBI-3-16S@NR_043664.1 | Tindallia texcoconensis | Bacteria | Bacillota | Clostridia | Peptostreptococcales | Tindalliaceae | Tindallia | Tindallia texcoconensis |
| Z3 | 171 | 17 | 0.05 | 697329 | NCBI-3-16S@NR_074399.1 | Ruminococcus albus 7 = DSM 20455 | Bacteria | Bacillota | Clostridia | Eubacteriales | Oscillospiraceae | Ruminococcus | Ruminococcus albus |
| Z3 | 172 | 17 | 0.05 | 915028 | NCBI-3-16S@HM635213.1 | Acetivibrio sp. enrichment culture clone WSC-3 | Bacteria | Bacillota | Clostridia | Eubacteriales | Oscillospiraceae | Acetivibrio | Acetivibrio sp. enrichment culture clone WSC-3 |
| Z3 | 173 | 17 | 0.05 | 1030129 | NCBI-3-16S@JF813176.1 | Bacteroides sp. dnLKV7 | Bacteria | Bacteroidota | Bacteroidia | Bacteroidales | Bacteroidaceae | Bacteroides | Bacteroides sp. dnLKV7 |
| Z3 | 174 | 17 | 0.05 | 2740579 | NCBI-3-16S@OQ989632.1 | Streptococcus vicugnae | Bacteria | Bacillota | Bacilli | Lactobacillales | Streptococcaceae | Streptococcus | Streptococcus vicugnae |
| Z3 | 175 | 17 | 0.05 | 575333 | NCBI-3-16S@AB470321.1 | Bacteroides sp. S-18 | Bacteria | Bacteroidota | Bacteroidia | Bacteroidales | Bacteroidaceae | Bacteroides | Bacteroides sp. S-18 |
| Z3 | 176 | 16 | 0.05 | 31899 | NCBI-3-16S@L09180.1 | Caldicellulosiruptor bescii | Bacteria | Bacillota |  | Caldicellulosiruptorales | Caldicellulosiruptoraceae | Caldicellulosiruptor | Caldicellulosiruptor bescii |
| Z3 | 177 | 16 | 0.05 | 1720203 | NCBI-3-16S@NR_169358.1 | Butyricimonas phoceensis | Bacteria | Bacteroidota | Bacteroidia | Bacteroidales | Odoribacteraceae | Butyricimonas | Butyricimonas phoceensis |
| Z3 | 178 | 16 | 0.05 | 1033732 | NCBI-3-16S@NR_118219.1 | Alistipes senegalensis JC50 | Bacteria | Bacteroidota | Bacteroidia | Bacteroidales | Rikenellaceae | Alistipes | Alistipes senegalensis |
| Z3 | 179 | 16 | 0.05 | 1920505 | NCBI-3-16S@OQ146993.1 | Pleomorphochaeta sp. | Bacteria | Spirochaetota | Spirochaetia | Spirochaetales | Sphaerochaetaceae | Pleomorphochaeta | Pleomorphochaeta sp. |
| Z3 | 180 | 16 | 0.05 | 2070686 | NCBI-3-16S@NR_179114.1 | Enteroscipio rubneri | Bacteria | Actinomycetota | Coriobacteriia | Eggerthellales | Eggerthellaceae | Enteroscipio | Enteroscipio rubneri |
| Z3 | 181 | 16 | 0.05 | 590931 | NCBI-3-16S@AB477432.1 | Clostridiales bacterium SY8526 | Bacteria | Bacillota | Clostridia | Eubacteriales |  |  | Clostridiales bacterium SY8526 |
| Z3 | 182 | 16 | 0.05 | 40518 | NCBI-3-16S@OK510346.1 | Ruminococcus bromii | Bacteria | Bacillota | Clostridia | Eubacteriales | Oscillospiraceae | Ruminococcus | Ruminococcus bromii |
| Z3 | 183 | 16 | 0.05 | 2763654 | NCBI-3-16S@NR_181383.1 | Luoshenia tenuis | Bacteria | Bacillota | Clostridia | Christensenellales | Christensenellaceae | Luoshenia | Luoshenia tenuis |
| Z3 | 184 | 16 | 0.05 | 1240100 | NCBI-3-16S@AB752501.1 | Coriobacteriaceae bacterium SNR48-44 | Bacteria | Actinomycetota | Coriobacteriia | Coriobacteriales | Coriobacteriaceae |  | Coriobacteriaceae bacterium SNR48-44 |
| Z3 | 185 | 16 | 0.05 | 326368 | NCBI-3-16S@DQ003622.1 | Prevotella genomosp. P8 oral clone MB3_P13 | Bacteria | Bacteroidota | Bacteroidia | Bacteroidales | Prevotellaceae | Prevotella | Prevotella genomosp. P8 oral clone MB3_P13 |
| Z3 | 186 | 16 | 0.05 | 1907662 | NCBI-3-16S@NR_148574.1 | Raoultibacter timonensis | Bacteria | Actinomycetota | Coriobacteriia | Eggerthellales | Eggerthellaceae | Raoultibacter | Raoultibacter timonensis |
| Z3 | 187 | 16 | 0.05 | 1965314 | NCBI-3-16S@MT829551.1 | Roseivivax sp. | Bacteria | Pseudomonadota | Alphaproteobacteria | Rhodobacterales | Roseobacteraceae | Roseivivax | Roseivivax sp. |
| Z3 | 188 | 16 | 0.05 | 1871018 | NCBI-3-16S@NR_179559.1 | Angelakisella massiliensis | Bacteria | Bacillota | Clostridia | Eubacteriales | Oscillospiraceae | Angelakisella | Angelakisella massiliensis |
| Z3 | 189 | 15 | 0.04 | 569500 | NCBI-3-16S@FJ269045.1 | iron-reducing bacterium enrichment culture clone HN3 | Bacteria |  |  |  |  |  | iron-reducing bacterium enrichment culture clone HN3 |
| Z3 | 190 | 15 | 0.04 | 1960295 | NCBI-3-16S@KX863562.1 | Sedimentibacter sp. | Bacteria | Bacillota | Tissierellia |  |  | Sedimentibacter | Sedimentibacter sp. |
| Z3 | 191 | 15 | 0.04 | 1796619 | NCBI-3-16S@NR_144613.1 | Irregularibacter muris | Bacteria | Bacillota | Clostridia | Eubacteriales | Eubacteriaceae | Irregularibacter | Irregularibacter muris |
| Z3 | 192 | 15 | 0.04 | 1816678 | NCBI-3-16S@NR_144743.1 | Christensenella timonensis | Bacteria | Bacillota | Clostridia | Christensenellales | Christensenellaceae | Christensenella | Christensenella timonensis |
| Z3 | 193 | 15 | 0.04 | 608506 | NCBI-3-16S@NR_117295.1 | Caldicellulosiruptor obsidiansis OB47 | Bacteria | Bacillota |  | Caldicellulosiruptorales | Caldicellulosiruptoraceae | Caldicellulosiruptor | Caldicellulosiruptor obsidiansis |
| Z3 | 194 | 15 | 0.04 | 1872092 | NCBI-3-16S@MZ310614.1 | Acetivibrio sp. | Bacteria | Bacillota | Clostridia | Eubacteriales | Oscillospiraceae | Acetivibrio | Acetivibrio sp. |
| Z3 | 195 | 15 | 0.04 | 2094145 | NCBI-3-16S@NR_179663.1 | Atopobium massiliense | Bacteria | Actinomycetota | Coriobacteriia | Coriobacteriales | Atopobiaceae | Atopobium | Atopobium massiliense |
| Z3 | 196 | 14 | 0.04 | 2739389 | NCBI-3-16S@NR_175548.1 | Phocaeicola faecicola | Bacteria | Bacteroidota | Bacteroidia | Bacteroidales | Bacteroidaceae | Phocaeicola | Phocaeicola faecicola |
| Z3 | 197 | 14 | 0.04 | 1265 | NCBI-3-16S@AF030449.1 | Ruminococcus flavefaciens | Bacteria | Bacillota | Clostridia | Eubacteriales | Oscillospiraceae | Ruminococcus | Ruminococcus flavefaciens |
| Z3 | 198 | 14 | 0.04 | 2202144 | NCBI-3-16S@ON560939.1 | Spirochaetota bacterium | Bacteria | Spirochaetota |  |  |  |  | Spirochaetota bacterium |
| Z3 | 199 | 14 | 0.04 | 2049025 | NCBI-3-16S@MN913777.1 | Flavonifractor sp. | Bacteria | Bacillota | Clostridia | Eubacteriales | Oscillospiraceae | Flavonifractor | Flavonifractor sp. |
| Z3 | 200 | 14 | 0.04 | 2163169 | NCBI-3-16S@MN081684.1 | Kineothrix sp. | Bacteria | Bacillota | Clostridia | Lachnospirales | Lachnospiraceae | Kineothrix | Kineothrix sp. |
| Z3 | 201 | 14 | 0.04 | 742742 | NCBI-3-16S@NR_113273.1 | Collinsella tanakaei YIT 12063 | Bacteria | Actinomycetota | Coriobacteriia | Coriobacteriales | Coriobacteriaceae | Collinsella | Collinsella tanakaei |
| Z3 | 202 | 14 | 0.04 | 604330 | NCBI-3-16S@OR673709.1 | Parafannyhessea umbonata | Bacteria | Actinomycetota | Coriobacteriia | Coriobacteriales | Atopobiaceae | Parafannyhessea | Parafannyhessea umbonata |
| Z3 | 203 | 14 | 0.04 | 691816 | NCBI-3-16S@NR_113072.1 | Bacteroides rodentium | Bacteria | Bacteroidota | Bacteroidia | Bacteroidales | Bacteroidaceae | Bacteroides | Bacteroides rodentium |
| Z3 | 204 | 14 | 0.04 | 53442 | NCBI-3-16S@X96961.1 | Eubacterium callanderi | Bacteria | Bacillota | Clostridia | Eubacteriales | Eubacteriaceae | Eubacterium | Eubacterium callanderi |
| Z3 | 205 | 14 | 0.04 | 1936999 | NCBI-3-16S@LT722679.1 | Lactonifactor sp. Marseille-P3743 | Bacteria | Bacillota | Clostridia | Eubacteriales | Clostridiaceae | Lactonifactor | Lactonifactor sp. Marseille-P3743 |
| Z3 | 206 | 14 | 0.04 | 74707 | NCBI-3-16S@MF678906.1 | Staphylococcus schleiferi subsp. schleiferi | Bacteria | Bacillota | Bacilli | Bacillales | Staphylococcaceae | Staphylococcus | Staphylococcus schleiferi |
| Z3 | 207 | 14 | 0.04 | 1411150 | NCBI-3-16S@HG531807.1 | Sphaerochaeta sp. DSM 26296 | Bacteria | Spirochaetota | Spirochaetia | Spirochaetales | Sphaerochaetaceae | Sphaerochaeta | Sphaerochaeta sp. DSM 26296 |
| Z3 | 208 | 14 | 0.04 | 763034 | NCBI-3-16S@NR_113068.1 | Bacteroides fluxus YIT 12057 | Bacteria | Bacteroidota | Bacteroidia | Bacteroidales | Bacteroidaceae | Bacteroides | Bacteroides fluxus |
| Z3 | 209 | 14 | 0.04 | 2930083 | NCBI-3-16S@OM533390.1 | Olsenella intestinalis | Bacteria | Actinomycetota | Coriobacteriia | Coriobacteriales | Atopobiaceae | Olsenella | Olsenella intestinalis |
| Z3 | 210 | 14 | 0.04 | 1230735 | NCBI-3-16S@JX262675.1 | Coriobacteriaceae bacterium S5-A3 | Bacteria | Actinomycetota | Coriobacteriia | Coriobacteriales | Coriobacteriaceae |  | Coriobacteriaceae bacterium S5-A3 |
| Z3 | 211 | 14 | 0.04 | 320502 | NCBI-3-16S@OK626616.1 | Acetivibrio alkalicellulosi | Bacteria | Bacillota | Clostridia | Eubacteriales | Oscillospiraceae | Acetivibrio | Acetivibrio alkalicellulosi |
| Z3 | 212 | 13 | 0.04 | 2779352 | NCBI-3-16S@NR_180619.1 | Pseudoflavonifractor gallinarum | Bacteria | Bacillota | Clostridia | Eubacteriales | Oscillospiraceae | Pseudoflavonifractor | Pseudoflavonifractor gallinarum |
| Z3 | 213 | 13 | 0.04 | 2086583 | NCBI-3-16S@NR_179661.1 | Pseudoruminococcus massiliensis | Bacteria | Bacillota | Clostridia | Eubacteriales | Acutalibacteraceae | Pseudoruminococcus | Pseudoruminococcus massiliensis |
| Z3 | 214 | 13 | 0.04 | 246199 | NCBI-3-16S@AY445594.1 | Ruminococcus albus 8 | Bacteria | Bacillota | Clostridia | Eubacteriales | Oscillospiraceae | Ruminococcus | Ruminococcus albus |
| Z3 | 215 | 13 | 0.04 | 1161942 | NCBI-3-16S@MH282446.1 | Ruminococcus champanellensis | Bacteria | Bacillota | Clostridia | Eubacteriales | Oscillospiraceae | Ruminococcus | Ruminococcus champanellensis |
| Z3 | 216 | 13 | 0.04 | 349096 | NCBI-3-16S@NR_043658.1 | Pectinatus haikarae | Bacteria | Bacillota | Negativicutes | Selenomonadales | Selenomonadaceae | Pectinatus | Pectinatus haikarae |
| Z3 | 217 | 13 | 0.04 | 1577308 | NCBI-3-16S@KM462079.1 | Lachnospiraceae bacterium feline oral taxon 003 | Bacteria | Bacillota | Clostridia | Lachnospirales | Lachnospiraceae |  | Lachnospiraceae bacterium feline oral taxon 003 |
| Z3 | 218 | 13 | 0.04 | 1796620 | NCBI-3-16S@NR_144605.1 | Acutalibacter muris | Bacteria | Bacillota | Clostridia | Eubacteriales | Acutalibacteraceae | Acutalibacter | Acutalibacter muris |
| Z3 | 219 | 13 | 0.04 | 2231116 | NCBI-3-16S@OR534223.1 | Mycoplasmatota bacterium | Bacteria | Mycoplasmatota |  |  |  |  | Mycoplasmatota bacterium |
| Z3 | 220 | 13 | 0.04 | 376806 | NCBI-3-16S@NR_041448.1 | Bacteroides gallinarum | Bacteria | Bacteroidota | Bacteroidia | Bacteroidales | Bacteroidaceae | Bacteroides | Bacteroides gallinarum |
| Z3 | 221 | 13 | 0.04 | 1155414 | NCBI-3-16S@JN688037.1 | bacterium enrichment culture clone M235 | Bacteria |  |  |  |  |  | bacterium enrichment culture clone M235 |
| Z3 | 222 | 13 | 0.04 | 1907658 | NCBI-3-16S@NR_179567.1 | Bacteroides ilei | Bacteria | Bacteroidota | Bacteroidia | Bacteroidales | Bacteroidaceae | Bacteroides | Bacteroides ilei |
| Z3 | 223 | 13 | 0.04 | 519017 | NCBI-3-16S@EU592964.1 | Olsenella sp. F0004 | Bacteria | Actinomycetota | Coriobacteriia | Coriobacteriales | Atopobiaceae | Olsenella | Olsenella sp. F0004 |
| Z3 | 224 | 13 | 0.04 | 172901 | NCBI-3-16S@NR_118352.1 | Victivallis vadensis | Bacteria | Lentisphaerota | Lentisphaeria | Victivallales | Victivallaceae | Victivallis | Victivallis vadensis |
| Z3 | 225 | 13 | 0.04 | 1469948 | NCBI-3-16S@NR_156077.1 | Kineothrix alysoides | Bacteria | Bacillota | Clostridia | Lachnospirales | Lachnospiraceae | Kineothrix | Kineothrix alysoides |
| Z3 | 226 | 13 | 0.04 | 1414721 | NCBI-3-16S@OP727734.1 | Clostridium jeddahense | Bacteria | Bacillota | Clostridia | Eubacteriales | Oscillospiraceae | Faecalispora | Faecalispora jeddahensis |
| Z3 | 227 | 13 | 0.04 | 2981726 | NCBI-3-16S@OK510342.1 | Hominimerdicola aceti | Bacteria | Bacillota | Clostridia | Eubacteriales | Oscillospiraceae | Hominimerdicola | Hominimerdicola aceti |
| Z3 | 228 | 13 | 0.04 | 437897 | NCBI-3-16S@MN055955.1 | Megamonas funiformis | Bacteria | Bacillota | Negativicutes | Selenomonadales | Selenomonadaceae | Megamonas | Megamonas funiformis |
| Z3 | 229 | 13 | 0.04 | 1159223 | NCBI-3-16S@AB702937.1 | Clostridiales bacterium CIEAF 021 | Bacteria | Bacillota | Clostridia | Eubacteriales |  |  | Clostridiales bacterium CIEAF 021 |
| Z3 | 230 | 12 | 0.04 | 1220747 | NCBI-3-16S@JQ771483.1 | bacterium enrichment culture clone E34 | Bacteria |  |  |  |  |  | bacterium enrichment culture clone E34 |
| Z3 | 231 | 12 | 0.04 | 1197717 | NCBI-3-16S@MN537495.1 | Cloacibacillus porcorum | Bacteria | Synergistota | Synergistia | Synergistales | Synergistaceae | Cloacibacillus | Cloacibacillus porcorum |
| Z3 | 232 | 12 | 0.04 | 57664 | NCBI-3-16S@Y11560.1 | Desulfuromonas thiophila | Bacteria | Thermodesulfobacteriota | Desulfuromonadia | Desulfuromonadales | Desulfuromonadaceae | Desulfuromonas | Desulfuromonas thiophila |
| Z3 | 233 | 12 | 0.04 | 689779 | NCBI-3-16S@GU124470.1 | Eubacterium sp. SA11 | Bacteria | Bacillota | Clostridia | Eubacteriales | Eubacteriaceae | Eubacterium | Eubacterium sp. SA11 |
| Z3 | 234 | 12 | 0.04 | 880447 | NCBI-3-16S@U26054.1 | Mycoplasma leachii PG50 | Bacteria | Mycoplasmatota | Mollicutes | Mycoplasmatales | Mycoplasmataceae | Mycoplasma | Mycoplasma leachii |
| Z3 | 235 | 12 | 0.04 | 1161950 | NCBI-3-16S@JQ773354.1 | bacterium enrichment culture clone PKS4 | Bacteria |  |  |  |  |  | bacterium enrichment culture clone PKS4 |
| Z3 | 236 | 12 | 0.04 | 2779354 | NCBI-3-16S@NR_180622.1 | Gemmiger gallinarum | Bacteria | Bacillota | Clostridia | Eubacteriales |  | Gemmiger | Gemmiger gallinarum |
| Z3 | 237 | 12 | 0.04 | 241556 | NCBI-3-16S@AY341819.1 | Bacteroidales genomosp. P1 | Bacteria | Bacteroidota | Bacteroidia | Bacteroidales |  |  | Bacteroidales genomosp. P1 |
| Z3 | 238 | 12 | 0.04 | 569465 | NCBI-3-16S@FJ269084.1 | iron-reducing bacterium enrichment culture clone HN-HFO4 | Bacteria |  |  |  |  |  | iron-reducing bacterium enrichment culture clone HN-HFO4 |
| Z3 | 239 | 12 | 0.04 | 1151611 | NCBI-3-16S@JN713207.1 | Bacteroidia bacterium canine oral taxon 041 | Bacteria | Bacteroidota | Bacteroidia |  |  |  | Bacteroidia bacterium canine oral taxon 041 |
| Z3 | 240 | 12 | 0.04 | 2046249 | NCBI-3-16S@MT312829.1 | Centipeda sp. (in: firmicutes) | Bacteria | Bacillota | Negativicutes | Selenomonadales | Selenomonadaceae | Centipeda | Centipeda sp. (in: firmicutes) |
| Z3 | 241 | 12 | 0.04 | 38403 | NCBI-3-16S@MT007981.1 | Erysipelothrix sp. | Bacteria | Bacillota | Erysipelotrichia | Erysipelotrichales | Erysipelotrichaceae | Erysipelothrix | Erysipelothrix sp. |
| Z3 | 242 | 12 | 0.04 | 626930 | NCBI-3-16S@MW558146.1 | Bacteroides fluxus | Bacteria | Bacteroidota | Bacteroidia | Bacteroidales | Bacteroidaceae | Bacteroides | Bacteroides fluxus |
| Z3 | 243 | 12 | 0.04 | 2496531 | NCBI-3-16S@MK287625.1 | Murimonas sp. | Bacteria | Bacillota | Clostridia | Lachnospirales | Lachnospiraceae | Murimonas | Murimonas sp. |
| Z3 | 244 | 12 | 0.04 | 3047475 | NCBI-3-16S@OQ998951.1 | Gordonibacter sp. KGMB12511 | Bacteria | Actinomycetota | Coriobacteriia | Eggerthellales | Eggerthellaceae | Gordonibacter | Gordonibacter faecis |
| Z3 | 245 | 12 | 0.04 | 1630007 | NCBI-3-16S@LC033795.1 | Clostridiales bacterium W23 | Bacteria | Bacillota | Clostridia | Eubacteriales |  |  | Clostridiales bacterium W23 |
| Z3 | 246 | 12 | 0.04 | 2024197 | NCBI-3-16S@NR_179635.1 | Bacteroides cutis | Bacteria | Bacteroidota | Bacteroidia | Bacteroidales | Bacteroidaceae | Bacteroides | Bacteroides cutis |
| Z3 | 247 | 12 | 0.04 | 29323 | NCBI-3-16S@NR_117608.1 | Thermoanaerobacter brockii | Bacteria | Bacillota | Clostridia | Thermoanaerobacterales | Thermoanaerobacteraceae | Thermoanaerobacter | Thermoanaerobacter brockii |
| Z3 | 248 | 12 | 0.04 | 1872444 | NCBI-3-16S@MN611110.1 | Alistipes sp. | Bacteria | Bacteroidota | Bacteroidia | Bacteroidales | Rikenellaceae | Alistipes | Alistipes sp. |
| Z3 | 249 | 11 | 0.03 | 1299440 | NCBI-3-18S@AB795026.1 | Alloiozona trizona | Eukaryota | Ciliophora | Litostomatea | Entodiniomorphida | Buetschliidae | Alloiozona | Alloiozona trizona |
| Z3 | 250 | 11 | 0.03 | 39492 | NCBI-3-16S@MT903124.1 | [Eubacterium] siraeum | Bacteria | Bacillota | Clostridia | Eubacteriales | Oscillospiraceae |  | [Eubacterium] siraeum |
| Z3 | 251 | 11 | 0.03 | 2986072 | NCBI-3-16S@OX352005.1 | Candidatus Minimicrobia sp. IHU4 | Bacteria | Candidatus Saccharibacteria |  |  |  | Candidatus Minimicrobia | Candidatus Minimicrobia sp. IHU4 |
| Z3 | 252 | 11 | 0.03 | 1898207 | NCBI-3-16S@MK170164.1 | Clostridiales bacterium | Bacteria | Bacillota | Clostridia | Eubacteriales |  |  | Clostridiales bacterium |
| Z3 | 253 | 11 | 0.03 | 1347151 | NCBI-3-16S@KF030226.1 | Petrimonas sp. canine oral taxon 434 | Bacteria | Bacteroidota | Bacteroidia | Bacteroidales | Dysgonomonadaceae | Petrimonas | Petrimonas sp. canine oral taxon 434 |
| Z3 | 254 | 11 | 0.03 | 1516126 | NCBI-3-16S@NR_178264.1 | Dysgonomonas termitidis | Bacteria | Bacteroidota | Bacteroidia | Bacteroidales | Dysgonomonadaceae | Dysgonomonas | Dysgonomonas termitidis |
| Z3 | 255 | 11 | 0.03 | 1462571 | NCBI-3-16S@NR_148822.1 | Bacteroides gallinaceum | Bacteria | Bacteroidota | Bacteroidia | Bacteroidales | Bacteroidaceae | Bacteroides | Bacteroides gallinaceum |
| Z3 | 256 | 11 | 0.03 | 2773926 | NCBI-3-16S@OP762691.2 | Phocaeicola sp. | Bacteria | Bacteroidota | Bacteroidia | Bacteroidales | Bacteroidaceae | Phocaeicola | Phocaeicola sp. |
| Z3 | 257 | 11 | 0.03 | 213810 | NCBI-3-16S@NR_114889.1 | Ruminococcus champanellensis 18P13 = JCM 17042 | Bacteria | Bacillota | Clostridia | Eubacteriales | Oscillospiraceae | Ruminococcus | Ruminococcus champanellensis |
| Z3 | 258 | 11 | 0.03 | 915177 | NCBI-3-16S@HQ452859.1 | Clostridiales bacterium 60-7e | Bacteria | Bacillota | Clostridia | Eubacteriales |  |  | Clostridiales bacterium 60-7e |
| Z3 | 259 | 11 | 0.03 | 28113 | NCBI-3-16S@KP334251.1 | Bacteroides heparinolyticus | Bacteria | Bacteroidota | Bacteroidia | Bacteroidales | Bacteroidaceae | Bacteroides | Bacteroides heparinolyticus |
| Z3 | 260 | 11 | 0.03 | 89153 | NCBI-3-16S@LC480797.1 | [Clostridium] hylemonae | Bacteria | Bacillota | Clostridia | Lachnospirales | Lachnospiraceae | Lachnoclostridium | [Clostridium] hylemonae |
| Z3 | 261 | 11 | 0.03 | 1918636 | NCBI-3-16S@MN081649.1 | Acutalibacter sp. | Bacteria | Bacillota | Clostridia | Eubacteriales | Acutalibacteraceae | Acutalibacter | Acutalibacter sp. |
| Z3 | 262 | 11 | 0.03 | 1288121 | NCBI-3-16S@NR_118219.1 | Alistipes senegalensis | Bacteria | Bacteroidota | Bacteroidia | Bacteroidales | Rikenellaceae | Alistipes | Alistipes senegalensis |
| Z3 | 263 | 11 | 0.03 | 1030130 | NCBI-3-16S@JF813178.1 | Bacteroides sp. dnLKV9 | Bacteria | Bacteroidota | Bacteroidia | Bacteroidales | Bacteroidaceae | Bacteroides | Bacteroides sp. dnLKV9 |
| Z3 | 264 | 11 | 0.03 | 1720065 | NCBI-3-16S@LN881570.1 | Tepidimicrobium sp. GRM1 | Bacteria | Bacillota | Tissierellia | Tissierellales | Tepidimicrobiaceae | Tepidimicrobium | Tepidimicrobium sp. GRM1 |
| Z3 | 265 | 10 | 0.03 | 1796613 | NCBI-3-16S@OK626630.1 | Bacteroides caecimuris | Bacteria | Bacteroidota | Bacteroidia | Bacteroidales | Bacteroidaceae | Bacteroides | Bacteroides caecimuris |
| Z3 | 266 | 10 | 0.03 | 2763660 | NCBI-3-16S@NR_181384.1 | Feifania hominis | Bacteria | Bacillota | Clostridia | Eubacteriales | Feifaniaceae | Feifania | Feifania hominis |
| Z3 | 267 | 10 | 0.03 | 1837337 | NCBI-3-16S@KX009915.1 | Lachnospiraceae bacterium DW8 | Bacteria | Bacillota | Clostridia | Lachnospirales | Lachnospiraceae |  | Lachnospiraceae bacterium DW8 |
| Z3 | 268 | 10 | 0.03 | 2042683 | NCBI-3-16S@MT323094.2 | Olsenella sp. | Bacteria | Actinomycetota | Coriobacteriia | Coriobacteriales | Atopobiaceae | Olsenella | Olsenella sp. |
| Z3 | 269 | 10 | 0.03 | 3032870 | NCBI-3-16S@MK929052.1 | Lepagella muris | Bacteria | Bacteroidota | Bacteroidia | Bacteroidales | Muribaculaceae | Lepagella | Lepagella muris |
| Z3 | 270 | 10 | 0.03 | 1379340 | NCBI-3-16S@KF312293.1 | Paenibacillus sp. TWNG08 | Bacteria | Bacillota | Bacilli | Bacillales | Paenibacillaceae | Paenibacillus | Paenibacillus sp. TWNG08 |
| Z3 | 271 | 10 | 0.03 | 2049042 | NCBI-3-16S@MK287738.1 | Turicibacter sp. | Bacteria | Bacillota | Erysipelotrichia | Erysipelotrichales | Turicibacteraceae | Turicibacter | Turicibacter sp. |
| Z3 | 272 | 10 | 0.03 | 888721 | NCBI-3-16S@NR_114689.1 | Eubacterium minutum ATCC 700079 | Bacteria | Bacillota | Clostridia | Eubacteriales | Eubacteriales Family XIII. Incertae Sedis |  | [Eubacterium] minutum |
| Z3 | 273 | 10 | 0.03 | 446660 | NCBI-3-16S@OK510307.1 | Adlercreutzia equolifaciens | Bacteria | Actinomycetota | Coriobacteriia | Eggerthellales | Eggerthellaceae | Adlercreutzia | Adlercreutzia equolifaciens |
| Z3 | 274 | 10 | 0.03 | 1236538 | NCBI-3-16S@NR_113064.1 | Phocaeicola sartorii JCM 16497 | Bacteria | Bacteroidota | Bacteroidia | Bacteroidales | Bacteroidaceae | Phocaeicola | Phocaeicola sartorii |
| Z3 | 275 | 10 | 0.03 | 525256 | NCBI-3-16S@NR_117757.1 | Fannyhessea vaginae DSM 15829 | Bacteria | Actinomycetota | Coriobacteriia | Coriobacteriales | Atopobiaceae | Fannyhessea | Fannyhessea vaginae |
| Z3 | 276 | 10 | 0.03 | 671233 | NCBI-3-16S@GQ422717.1 | Veillonellaceae bacterium oral taxon 129 | Bacteria | Bacillota | Negativicutes | Veillonellales | Veillonellaceae |  | Veillonellaceae bacterium oral taxon 129 |
| Z3 | 277 | 10 | 0.03 | 2972488 | NCBI-3-16S@OP402864.1 | Paenibacillus radicibacter | Bacteria | Bacillota | Bacilli | Bacillales | Paenibacillaceae | Paenibacillus | Paenibacillus radicibacter |
| Z3 | 278 | 10 | 0.03 | 61171 | NCBI-3-16S@LT558818.1 | Holdemania filiformis | Bacteria | Bacillota | Erysipelotrichia | Erysipelotrichales | Erysipelotrichaceae | Holdemania | Holdemania filiformis |
| Z3 | 279 | 10 | 0.03 | 78329 | NCBI-3-16S@Y17612.1 | Coenonia anatina | Bacteria | Bacteroidota | Flavobacteriia | Flavobacteriales | Flavobacteriaceae | Allocoenonia | Allocoenonia anatina |
| Z3 | 280 | 10 | 0.03 | 2585119 | NCBI-3-16S@NR_179296.1 | Alistipes dispar | Bacteria | Bacteroidota | Bacteroidia | Bacteroidales | Rikenellaceae | Alistipes | Alistipes dispar |
| Z3 | 281 | 10 | 0.03 | 246787 | NCBI-3-16S@OP690569.1 | Bacteroides cellulosilyticus | Bacteria | Bacteroidota | Bacteroidia | Bacteroidales | Bacteroidaceae | Bacteroides | Bacteroides cellulosilyticus |
| Z3 | 282 | 10 | 0.03 | 2763658 | NCBI-3-16S@NR_181388.1 | Ligaoa zhengdingensis | Bacteria | Bacillota | Clostridia | Eubacteriales | Oscillospiraceae | Ligaoa | Ligaoa zhengdingensis |
| Z3 | 283 | 10 | 0.03 | 467023 | NCBI-3-18S@EF632074.1 | Tripalmaria dogieli | Eukaryota | Ciliophora | Litostomatea | Entodiniomorphida | Cycloposthiidae | Tripalmaria | Tripalmaria dogieli |
| Z3 | 284 | 10 | 0.03 | 1945593 | NCBI-3-16S@MK287649.1 | Oscillibacter sp. | Bacteria | Bacillota | Clostridia | Eubacteriales | Oscillospiraceae | Oscillibacter | Oscillibacter sp. |
| Z3 | 285 | 10 | 0.03 | 720849 | NCBI-3-16S@GU374060.1 | bacterium enrichment culture clone SRC_DSC3 | Bacteria |  |  |  |  |  | bacterium enrichment culture clone SRC_DSC3 |
| Z3 | 286 | 10 | 0.03 | 2585118 | NCBI-3-16S@ON705225.1 | Alistipes communis | Bacteria | Bacteroidota | Bacteroidia | Bacteroidales | Rikenellaceae | Alistipes | Alistipes communis |
| Z3 | 287 | 10 | 0.03 | 847 | NCBI-3-16S@HG917902.1 | Oxalobacter formigenes | Bacteria | Pseudomonadota | Betaproteobacteria | Burkholderiales | Oxalobacteraceae | Oxalobacter | Oxalobacter formigenes |
