# Supplementary material for: Full‐Length 16S and 18S rRNA Long‐Read Sequencing Reveals Gut Microbiome Diversity in the European Brown Hare ( Lepus europaeus )
Source: Environ Microbiol Rep. 2026 May 25;18(3):e70358. doi: 10.1111/1758-2229.70358 (PMC13239162; doi:10.1111/1758-2229.70358)
Supplement: Supplementary file 2 — Table S2: Quantitative group comparison analysis of detected microbial taxa across hierarchical classification levels at 80% and 95% sequence identity thresholds. This dataset represents the underlying values for Figure 3, detailing the average number of taxa identified per sample, taxa unique to each stringency threshold, and the core set of shared taxa. [file EMI4-18-e70358-s002.docx]

# Data from Supplementary Table 2 to Figure 3

Total entries: 128

Columns: Panel, Taxonomic_Level, Taxon, Threshold, Percentage, Color, Unique_To_Threshold, NCBI_Tax_ID

| **Panel** | **Taxonomic_Level** | **Taxon** | **Threshold** | **Percentage** | **Color** | **Unique_To_Threshold** | **NCBI_Tax_ID** |
| --- | --- | --- | --- | --- | --- | --- | --- |
| A | Phylum | Bacillota | 80% | 39.7 | #e377c2 | False | nan |
| A | Phylum | Bacteroidota | 80% | 25.9 | #9edae5 | False | nan |
| A | Phylum | Spirochaetota | 80% | 25.2 | #bcbd22 | True | nan |
| A | Phylum | Actinomycetota | 80% | 6.6 | #9467bd | False | nan |
| A | Phylum | Other (<1%) | 80% | 2.7 | #1f77b4 | False | nan |
| A | Phylum | Bacteroidota | 95% | 59.6 | #9edae5 | False | nan |
| A | Phylum | Bacillota | 95% | 31.7 | #e377c2 | False | nan |
| A | Phylum | Ascomycota | 95% | 4.3 | #2ca02c | True | nan |
| A | Phylum | Other (<1%) | 95% | 2.9 | #1f77b4 | False | nan |
| A | Phylum | Actinomycetota | 95% | 1.5 | #9467bd | False | nan |
| B | Class | Clostridia | 80% | 29.8 | #1f77b4 | False | nan |
| B | Class | Bacteroidia | 80% | 26.7 | #c7c7c7 | False | nan |
| B | Class | Spirochaetia | 80% | 26.5 | #9edae5 | True | nan |
| B | Class | Coriobacteriia | 80% | 6.6 | #ff9896 | False | nan |
| B | Class | Erysipelotrichia | 80% | 3.9 | #dbdb8d | True | nan |
| B | Class | Other (<1%) | 80% | 3.4 | #ff7f0e | False | nan |
| B | Class | Bacilli | 80% | 2.1 | #e377c2 | False | nan |
| B | Class | Negativicutes | 80% | 1.0 | #8c564b | True | nan |
| B | Class | Bacteroidia | 95% | 61.4 | #c7c7c7 | False | nan |
| B | Class | Clostridia | 95% | 22.7 | #1f77b4 | False | nan |
| B | Class | Bacilli | 95% | 7.3 | #e377c2 | False | nan |
| B | Class | Other (<1%) | 95% | 4.3 | #ff7f0e | False | nan |
| B | Class | Saccharomycetes | 95% | 3.1 | #98df8a | True | nan |
| B | Class | Coriobacteriia | 95% | 1.2 | #ff9896 | False | nan |
| C | Order | Spirochaetales | 80% | 26.6 | #98df8a | True | nan |
| C | Order | Bacteroidales | 80% | 26.6 | #ff9896 | False | nan |
| C | Order | Eubacteriales | 80% | 24.4 | #e377c2 | False | nan |
| C | Order | Other (<1%) | 80% | 6.8 | #aec7e8 | False | nan |
| C | Order | Lachnospirales | 80% | 4.0 | #bcbd22 | False | nan |
| C | Order | Erysipelotrichales | 80% | 3.9 | #8c564b | True | nan |
| C | Order | Coriobacteriales | 80% | 3.4 | #17becf | True | nan |
| C | Order | Eggerthellales | 80% | 3.0 | #c5b0d5 | True | nan |
| C | Order | Bacillales | 80% | 1.3 | #9edae5 | False | nan |
| C | Order | Bacteroidales | 95% | 61.2 | #ff9896 | False | nan |
| C | Order | Eubacteriales | 95% | 16.8 | #e377c2 | False | nan |
| C | Order | Other (<1%) | 95% | 5.8 | #aec7e8 | False | nan |
| C | Order | Lactobacillales | 95% | 4.1 | #7f7f7f | True | nan |
| C | Order | Monoglobales | 95% | 3.8 | #1f77b4 | True | nan |
| C | Order | Bacillales | 95% | 3.1 | #9edae5 | False | nan |
| C | Order | Saccharomycetales | 95% | 3.1 | #ffbb78 | True | nan |
| C | Order | Lachnospirales | 95% | 1.9 | #bcbd22 | False | nan |
| D | Family | Spirochaetaceae | 80% | 26.4 | #d62728 | True | nan |
| D | Family | Oscillospiraceae | 80% | 15.7 | #9edae5 | False | nan |
| D | Family | Other (<1%) | 80% | 14.0 | #1f77b4 | False | nan |
| D | Family | Bacteroidaceae | 80% | 11.2 | #7f7f7f | False | nan |
| D | Family | Odoribacteraceae | 80% | 6.7 | #e377c2 | True | nan |
| D | Family | Lachnospiraceae | 80% | 4.0 | #f7b6d2 | False | nan |
| D | Family | Prevotellaceae | 80% | 3.8 | #c5b0d5 | False | nan |
| D | Family | Erysipelotrichaceae | 80% | 3.6 | #ffbb78 | True | nan |
| D | Family | Eggerthellaceae | 80% | 3.2 | #17becf | True | nan |
| D | Family | Atopobiaceae | 80% | 2.1 | #9edae5 | True | nan |
| D | Family | Rikenellaceae | 80% | 2.0 | #ff7f0e | False | nan |
| D | Family | Clostridiaceae | 80% | 1.9 | #dbdb8d | True | nan |
| D | Family | Sphaerochaetaceae | 80% | 1.7 | #ff9896 | True | nan |
| D | Family | Muribaculaceae | 80% | 1.3 | #aec7e8 | True | nan |
| D | Family | Porphyromonadaceae | 80% | 1.3 | #2ca02c | True | nan |
| D | Family | Coriobacteriaceae | 80% | 1.2 | #c7c7c7 | True | nan |
| D | Family | Bacteroidaceae | 95% | 51.5 | #7f7f7f | False | nan |
| D | Family | Oscillospiraceae | 95% | 13.2 | #9edae5 | False | nan |
| D | Family | Other (<1%) | 95% | 9.9 | #1f77b4 | False | nan |
| D | Family | Prevotellaceae | 95% | 4.5 | #c5b0d5 | False | nan |
| D | Family | Monoglobaceae | 95% | 3.9 | #8c564b | True | nan |
| D | Family | Streptococcaceae | 95% | 3.4 | #c49c94 | True | nan |
| D | Family | Tannerellaceae | 95% | 3.2 | #98df8a | True | nan |
| D | Family | Staphylococcaceae | 95% | 3.2 | #bcbd22 | True | nan |
| D | Family | Saccharomycetaceae | 95% | 3.2 | #9467bd | True | nan |
| D | Family | Rikenellaceae | 95% | 2.0 | #ff7f0e | False | nan |
| D | Family | Lachnospiraceae | 95% | 1.9 | #f7b6d2 | False | nan |
| E | Genus | Other (<1%) | 80% | 32.6 | #aec7e8 | False | nan |
| E | Genus | Spirochaeta | 80% | 26.3 | #8c564b | True | nan |
| E | Genus | Ruminococcus | 80% | 7.7 | #7f7f7f | False | nan |
| E | Genus | Bacteroides | 80% | 7.4 | #1f77b4 | False | nan |
| E | Genus | Odoribacter | 80% | 4.7 | #c5b0d5 | True | nan |
| E | Genus | Phocaeicola | 80% | 3.6 | #e377c2 | False | nan |
| E | Genus | Paraprevotella | 80% | 3.6 | #9edae5 | False | nan |
| E | Genus | Dielma | 80% | 2.9 | #ff9896 | True | nan |
| E | Genus | Alistipes | 80% | 2.0 | #f7b6d2 | False | nan |
| E | Genus | Clostridium | 80% | 1.6 | #c7c7c7 | True | nan |
| E | Genus | Butyricimonas | 80% | 1.5 | #d62728 | True | nan |
| E | Genus | Sphaerochaeta | 80% | 1.4 | #98df8a | True | nan |
| E | Genus | Atopobium | 80% | 1.2 | #9edae5 | True | nan |
| E | Genus | Adlercreutzia | 80% | 1.1 | #ff7f0e | True | nan |
| E | Genus | Gabonibacter | 80% | 1.1 | #9467bd | True | nan |
| E | Genus | Pseudoflavonifractor | 80% | 1.1 | #2ca02c | False | nan |
| E | Genus | Bacteroides | 95% | 26.7 | #1f77b4 | False | nan |
| E | Genus | Phocaeicola | 95% | 26.0 | #e377c2 | False | nan |
| E | Genus | Other (<1%) | 95% | 14.7 | #aec7e8 | False | nan |
| E | Genus | Ruminococcus | 95% | 6.6 | #7f7f7f | False | nan |
| E | Genus | Paraprevotella | 95% | 4.6 | #9edae5 | False | nan |
| E | Genus | Monoglobus | 95% | 4.0 | #17becf | True | nan |
| E | Genus | Streptococcus | 95% | 3.5 | #dbdb8d | True | nan |
| E | Genus | Parabacteroides | 95% | 3.3 | #ffbb78 | True | nan |
| E | Genus | Staphylococcus | 95% | 3.3 | #c49c94 | True | nan |
| E | Genus | Cyniclomyces | 95% | 3.3 | #bcbd22 | True | nan |
| E | Genus | Pseudoflavonifractor | 95% | 2.1 | #2ca02c | False | nan |
| E | Genus | Alistipes | 95% | 2.1 | #f7b6d2 | False | nan |
| F | Species | Other (<1%) | 80% | 52.8 | #c49c94 | False | nan |
| F | Species | Spirochaeta sp. canine oral taxon 379 | 80% | 21.3 | #1f77b4 | True | 1151583.0 |
| F | Species | Bacillota bacterium | 80% | 4.2 | #1f77b4 | False | 1879010.0 |
| F | Species | Odoribacter sp. | 80% | 3.3 | #aec7e8 | True | 1965233.0 |
| F | Species | Paraprevotella clara | 80% | 2.7 | #ff7f0e | False | 454154.0 |
| F | Species | Dielma fastidiosa | 80% | 2.4 | #ffbb78 | True | 1034346.0 |
| F | Species | Bacteroides uniformis | 80% | 1.8 | #ffbb78 | False | 820.0 |
| F | Species | Clostridiales bacterium KM2 | 80% | 1.7 | #2ca02c | True | 862466.0 |
| F | Species | Phocaeicola vulgatus | 80% | 1.6 | #98df8a | False | 821.0 |
| F | Species | Ruminococcus champanellensis | 80% | 1.5 | #d62728 | True | 213810.0 |
| F | Species | Lachnospiraceae bacterium 19gly4 | 80% | 1.5 | #d62728 | True | 214819.0 |
| F | Species | Ruminococcus callidus | 80% | 1.2 | #ff9896 | True | 40519.0 |
| F | Species | bacterium enrichment culture clone DPF25 | 80% | 1.1 | #9467bd | True | 666483.0 |
| F | Species | Ruminococcus sp. | 80% | 1.1 | #c5b0d5 | True | 41978.0 |
| F | Species | Butyricimonas virosa | 80% | 0.9 | #8c564b | True | 544645.0 |
| F | Species | bacterium enrichment culture clone DPHB07 | 80% | 0.9 | #8c564b | True | 666493.0 |
| F | Species | Other (<1%) | 95% | 35.5 | #c49c94 | False | nan |
| F | Species | Phocaeicola vulgatus | 95% | 16.5 | #98df8a | False | 821.0 |
| F | Species | Bacteroides uniformis | 95% | 15.9 | #ffbb78 | False | 820.0 |
| F | Species | Phocaeicola dorei | 95% | 4.2 | #e377c2 | True | 357276.0 |
| F | Species | Paraprevotella clara | 95% | 4.1 | #ff7f0e | False | 454154.0 |
| F | Species | Monoglobus pectinilyticus | 95% | 3.7 | #f7b6d2 | True | 1981510.0 |
| F | Species | Cyniclomyces guttulatus | 95% | 3.0 | #f7b6d2 | True | 54199.0 |
| F | Species | Phocaeicola sartorii | 95% | 2.9 | #7f7f7f | True | 671267.0 |
| F | Species | Streptococcus canis | 95% | 2.5 | #c7c7c7 | True | 1329.0 |
| F | Species | Bacillota bacterium | 95% | 2.4 | #1f77b4 | False | 1879010.0 |
| F | Species | Parabacteroides distasonis | 95% | 1.9 | #bcbd22 | True | 823.0 |
| F | Species | Pseudoflavonifractor sp. | 95% | 1.8 | #bcbd22 | True | 1980281.0 |
| F | Species | Staphylococcus pseudintermedius | 95% | 1.5 | #dbdb8d | True | 283734.0 |
| F | Species | Bacteroides intestinalis | 95% | 1.5 | #17becf | True | 329854.0 |
| F | Species | Oscillospiraceae bacterium | 95% | 1.4 | #9edae5 | True | 2485925.0 |
| F | Species | bacterium enrichment culture clone ZZ_F11b | 95% | 1.1 | #9edae5 | True | 1309939.0 |

## Data by Panel

### Panel: A

Number of entries: 10

Average percentage: 20.01%

Preview:

| **Panel** | **Taxonomic_Level** | **Taxon** | **Threshold** | **Percentage** | **Color** | **Unique_To_Threshold** | **NCBI_Tax_ID** |
| --- | --- | --- | --- | --- | --- | --- | --- |
| A | Phylum | Bacillota | 80% | 39.7 | #e377c2 | False | nan |
| A | Phylum | Bacteroidota | 80% | 25.9 | #9edae5 | False | nan |
| A | Phylum | Spirochaetota | 80% | 25.2 | #bcbd22 | True | nan |
| A | Phylum | Actinomycetota | 80% | 6.6 | #9467bd | False | nan |
| A | Phylum | Other (<1%) | 80% | 2.7 | #1f77b4 | False | nan |

### Panel: B

Number of entries: 14

Average percentage: 14.29%

Preview:

| **Panel** | **Taxonomic_Level** | **Taxon** | **Threshold** | **Percentage** | **Color** | **Unique_To_Threshold** | **NCBI_Tax_ID** |
| --- | --- | --- | --- | --- | --- | --- | --- |
| B | Class | Clostridia | 80% | 29.8 | #1f77b4 | False | nan |
| B | Class | Bacteroidia | 80% | 26.7 | #c7c7c7 | False | nan |
| B | Class | Spirochaetia | 80% | 26.5 | #9edae5 | True | nan |
| B | Class | Coriobacteriia | 80% | 6.6 | #ff9896 | False | nan |
| B | Class | Erysipelotrichia | 80% | 3.9 | #dbdb8d | True | nan |

### Panel: C

Number of entries: 17

Average percentage: 11.75%

Preview:

| **Panel** | **Taxonomic_Level** | **Taxon** | **Threshold** | **Percentage** | **Color** | **Unique_To_Threshold** | **NCBI_Tax_ID** |
| --- | --- | --- | --- | --- | --- | --- | --- |
| C | Order | Spirochaetales | 80% | 26.6 | #98df8a | True | nan |
| C | Order | Bacteroidales | 80% | 26.6 | #ff9896 | False | nan |
| C | Order | Eubacteriales | 80% | 24.4 | #e377c2 | False | nan |
| C | Order | Other (<1%) | 80% | 6.8 | #aec7e8 | False | nan |
| C | Order | Lachnospirales | 80% | 4.0 | #bcbd22 | False | nan |

### Panel: D

Number of entries: 27

Average percentage: 7.41%

Preview:

| **Panel** | **Taxonomic_Level** | **Taxon** | **Threshold** | **Percentage** | **Color** | **Unique_To_Threshold** | **NCBI_Tax_ID** |
| --- | --- | --- | --- | --- | --- | --- | --- |
| D | Family | Spirochaetaceae | 80% | 26.4 | #d62728 | True | nan |
| D | Family | Oscillospiraceae | 80% | 15.7 | #9edae5 | False | nan |
| D | Family | Other (<1%) | 80% | 14.0 | #1f77b4 | False | nan |
| D | Family | Bacteroidaceae | 80% | 11.2 | #7f7f7f | False | nan |
| D | Family | Odoribacteraceae | 80% | 6.7 | #e377c2 | True | nan |

### Panel: E

Number of entries: 28

Average percentage: 7.14%

Preview:

| **Panel** | **Taxonomic_Level** | **Taxon** | **Threshold** | **Percentage** | **Color** | **Unique_To_Threshold** | **NCBI_Tax_ID** |
| --- | --- | --- | --- | --- | --- | --- | --- |
| E | Genus | Other (<1%) | 80% | 32.6 | #aec7e8 | False | nan |
| E | Genus | Spirochaeta | 80% | 26.3 | #8c564b | True | nan |
| E | Genus | Ruminococcus | 80% | 7.7 | #7f7f7f | False | nan |
| E | Genus | Bacteroides | 80% | 7.4 | #1f77b4 | False | nan |
| E | Genus | Odoribacter | 80% | 4.7 | #c5b0d5 | True | nan |

### Panel: F

Number of entries: 32

Average percentage: 6.25%

Preview:

| **Panel** | **Taxonomic_Level** | **Taxon** | **Threshold** | **Percentage** | **Color** | **Unique_To_Threshold** | **NCBI_Tax_ID** |
| --- | --- | --- | --- | --- | --- | --- | --- |
| F | Species | Other (<1%) | 80% | 52.8 | #c49c94 | False | nan |
| F | Species | Spirochaeta sp. canine oral taxon 379 | 80% | 21.3 | #1f77b4 | True | 1151583.0 |
| F | Species | Bacillota bacterium | 80% | 4.2 | #1f77b4 | False | 1879010.0 |
| F | Species | Odoribacter sp. | 80% | 3.3 | #aec7e8 | True | 1965233.0 |
| F | Species | Paraprevotella clara | 80% | 2.7 | #ff7f0e | False | 454154.0 |
