# Supplementary material for: Full‐Length 16S and 18S rRNA Long‐Read Sequencing Reveals Gut Microbiome Diversity in the European Brown Hare ( Lepus europaeus )
Source: Environ Microbiol Rep. 2026 May 25;18(3):e70358. doi: 10.1111/1758-2229.70358 (PMC13239162; doi:10.1111/1758-2229.70358)
Supplement: Supplementary file 3 — Table S3: Comprehensive inventory of microbial taxa reported in previous brown hare (Lepus europaeus) gut microbiome reference studies (Stalder et al. 2019 and Padula et al. 2021) that were absent from the current dataset. Lineages are mapped according to current NCBI taxonomy standards, highlighting both valid entries and those lacking defined database nomenclature. [file EMI4-18-e70358-s003.docx]

# Missing Taxa Report

This report lists taxa found in reference sources but missing from the current study.

| **Level** | **Source** | **NCBI Name** | **Published Name** | **NCBI Tax ID** |
| --- | --- | --- | --- | --- |
| Phylum | Padula et al. 2021 | Armatimonadota | Armatimonadetes | 67819.0 |
| Phylum | Padula et al. 2021 | Unclassified BRC1 | 1,00 BRC |  |
| Phylum | Padula et al. 2021 | Chlamydiota | Chlamydiae | 204428.0 |
| Phylum | Padula et al. 2021 | Unclassifed Chloroflexi (Wrong rank) | Chloroflexi |  |
| Phylum | Padula et al. 2021 | Unclassifed Deferribacteres (Wrong rank) | Deferribacteres |  |
| Phylum | Padula et al. 2021 | Unclassifed Dependentiae (Wrong rank) | Dependentiae |  |
| Phylum | Padula et al. 2021 | Unclassifed Elusimicrobia (Wrong rank) | Elusimicrobia |  |
| Phylum | Padula et al. 2021 | Campylobacterota | Epsilonbacteraeota | 29547.0 |
| Phylum | Padula et al. 2021 | Methanobacteriota | Euryarchaeota | 28890.0 |
| Phylum | Padula et al. 2021 | Unclassified FBP | FBP |  |
| Phylum | Padula et al. 2021 | Unclassified FCPU426 | FCPU426 |  |
| Phylum | Padula et al. 2021 | Unclassifed Firmicutes (Wrong rank) | Firmicutes |  |
| Phylum | Padula et al. 2021 | Gemmatimonadota | Gemmatimonadetes | 142182.0 |
| Phylum | Padula et al. 2021 | Kiritimatiellota | Kiritimatiellaeota | 134625.0 |
| Phylum | Padula et al. 2021 | Nanobdellota | Nanoarchaeota | 192989.0 |
| Phylum | Padula et al. 2021 | Candidatus Omnitrophota | Omnitrophicaeota | 67812.0 |
| Phylum | Padula et al. 2021 | Unclassifed Patescibacteria (Wrong rank) | Patescibacteria |  |
| Phylum | Padula et al. 2021 | Planctomycetota | Planctomycetes | 203682.0 |
| Phylum | Padula et al. 2021 | Unclassified Rokubacteria | Rokubacteria |  |
| Phylum | Padula et al. 2021 | Nitrososphaerota | Thaumarchaeota | 651137.0 |
| Phylum | Padula et al. 2021 | Verrucomicrobiota | Verrucomicrobia | 74201.0 |
| Phylum | Padula et al. 2021 | Unclassified WPS-2 | WPS-2 |  |
| Phylum | Padula et al. 2021 | Unclassified WS2 | WS2 |  |
| Phylum | Padula et al. 2021 | Unclassified | Zixibacteria |  |
| Phylum | Stalder et al. 2019 | Unclassifed Firmicutes (Wrong rank) | Firmicutes |  |
| Family | Stalder et al. 2019 | Unclassified Paraprevotellaceae | Paraprevotellaceae |  |
| Family | Stalder et al. 2019 | Brucellaceae | Brucellaceae | 118882.0 |
| Genus | Padula et al. 2021 | Unclassifed Bacteroidetes (Wrong rank) | Bacteroidetes |  |
| Genus | Padula et al. 2021 | Candidatus Saccharimonas | Candidatus Saccharimonas | 1331051.0 |
| Genus | Padula et al. 2021 | Unclassifed X.Eubacterium..ventriosum group (Wrong rank) | X.Eubacterium..ventriosum group |  |
| Genus | Padula et al. 2021 | Unclassified Fusicatenaibacter | Fusicatenaibacter |  |
| Genus | Padula et al. 2021 | Unclassified CAG.352 (Uncultured group) | CAG.352 |  |
| Genus | Padula et al. 2021 | Unclassified Lachnospiraceae.NK4B4 (Uncultured group) | Lachnospiraceae.NK4B4 |  |
| Genus | Padula et al. 2021 | Unclassified Oscillobacter | Oscillobacter |  |
| Species | Stalder et al. 2019 | Sphingobacterium wenxiniae | Sphingobacterium wenxiniae | 683125.0 |
| Species | Stalder et al. 2019 | Selenomonas dianae | Selenomonas dianae | 135079.0 |
| Species | Stalder et al. 2019 | Kyrpidia tusciae | Kyrpidia tusciae | 33943.0 |
